# Potential Mechanism of Dingji Fumai Decoction Against Atrial Fibrillation Based on Network Pharmacology, Molecular Docking, and Experimental Verification Integration Strategy

**DOI:** 10.3389/fcvm.2021.712398

**Published:** 2021-11-11

**Authors:** Yi Liang, Bo Liang, Wen Chen, Xin-Rui Wu, Wu-Sha Liu-Huo, Li-Zhi Zhao

**Affiliations:** ^1^Southwest Medical University, Luzhou, China; ^2^Nanjing University of Chinese Medicine, Nanjing, China; ^3^The Affiliated Traditional Chinese Medicine Hospital of Southwest Medical University, Luzhou, China

**Keywords:** Dingji Fumai Decoction, traditional Chinese medicine, atrial fibrillation, network pharmacology, molecular docking, experimental verification

## Abstract

**Background:** Dingji Fumai Decoction (DFD), a traditional herbal mixture, has been widely used to treat arrhythmia in clinical practice in China. However, the exploration of the active components and underlying mechanism of DFD in treating atrial fibrillation (AF) is still scarce.

**Methods:** Compounds of DFD were collected from TCMSP, ETCM, and literature. The targets of active compounds were explored using SwissTargetPrediction. Meanwhile, targets of AF were collected from DrugBank, TTD, MalaCards, TCMSP, DisGeNET, and OMIM. Then, the H-C-T-D and PPI networks were constructed using STRING and analyzed using CytoNCA. Meanwhile, VarElect was utilized to detect the correlation between targets and diseases. Next, Metascape was employed for systematic analysis of the mechanism of potential targets and protein complexes in treating AF. AutoDock Vina, Pymol, and Discovery Studio were applied for molecular docking. Finally, the main findings were validated through molecular biology experiments.

**Results:** A total of 168 active compounds and 1,093 targets of DFD were collected, and there were 89 shared targets between DFD and AF. H-C-T-D network showed the relationships among DFD, active compounds, targets, and AF. Three functional protein complexes of DFD were extracted from the PPI network. Further systematic analysis revealed that the regulation of cardiac oxidative stress, cardiac inflammation, and cardiac ion channels were the potential mechanism of DFD in treating AF. Addtionally, molecular docking verified the interactions between active compounds and targets. Finally, we found that DFD significantly increased the level of SIRT1 and reduced the levels of ACE, VCAM-1, and IL-6.

**Conclusions:** DFD could be utilized in treating AF through a complicated mechanism, including interactions between related active compounds and targets, promoting the explanation and understanding of the molecular biological mechanism of DFD in the treatment of AF.

## Introduction

Atrial fibrillation (AF) is a common but fatal clinical tachyarrhythmia and is caused by various factors that lead to the defect of original regular and orderly atrial electrical conduction activities and is showcased by rapid oscillations or fibrillatory waves on the electrocardiogram (1). AF could cause adverse hemodynamic effects, such as the loss of atrial contraction and rapid ventricular rates, leading to the decreasing of cardiac output (2). Especially, in patients with both chronic heart failure and AF, the situation would worsen (3). Besides, a study has pointed out that the incidence of ischemic stroke in patients with AF is seven times that of patients without AF (4). In recent years, the number of patients suffering from cardiovascular diseases, including coronary artery disease, hypertension, and heart failure, has increased year by year, becoming a potentially high-risk group of AF, which has greatly increased the burden of the medical system (5). A review of epidemiological study mentioned that the prevalence of AF increases with aging, which varies from 0.1 to 0.5% among patients <50 years old, to 1–2% in the 50–60 years old range, and 5–7% and higher in patients aged 70 years or older (6). The basic therapy of AF often contains anticoagulation, rate and rhythm control, and invasive treatment options including radiofrequency ablation or cryoablation of pulmonary veins, and surgical ablation (1, 7). However, most drug treatment may be accompanied by adverse and unpredictable events, and invasive treatment could be followed by the recurrence of AF (8), Therefore, we need to actively explore better treatment options.

Dingji Fumai Decoction (DFD), consisting of *Ligusticum chuanxiong* (Chuanxiong), *Ziziphus jujuba Mill* (Dazao), *Poria cocos (Schw.), Wolf* (Fuling), *Cinnamomum cassia (L.), J.Presl* (Guizhi), *Albizia julibrissin Durazz* (Hehuanpi), *Os Draconis* (Longgu), *Crassostrea Gigas* (Muli), *Semen Ziziphi Spinosae* (Suanzaoren), *Polygala tenuifolia Willd* (Yuanzhi), and *Glycyrrhiza uralensis Fisch* (Gancao), is widely used in treating arrhythmia (9). A previous clinical trial showed that after a 12-week follow-up, DFD significantly decreased the traditional Chinese medicine syndrome score and the number of arrhythmia compared with metoprolol with no adverse drug effects and acceptable patient medication compliance (10). Moreover, DFD prolonged the occurrence time and shortened the duration of arrhythmia *in vivo*, decreased the malondialdehyde and increased the superoxide dismutase, and alleviated the activation of Na^+^-K^+^-ATPase and connexin-43 (11). Further, DFD suppressed Nav_1.5_ dose-dependently with an IC_50_ of 24.0 ± 2.4 mg/mL (11). However, the exploration of the active components in DFD and underlying mechanism for treating AF is still scarce.

**Figure 1:**
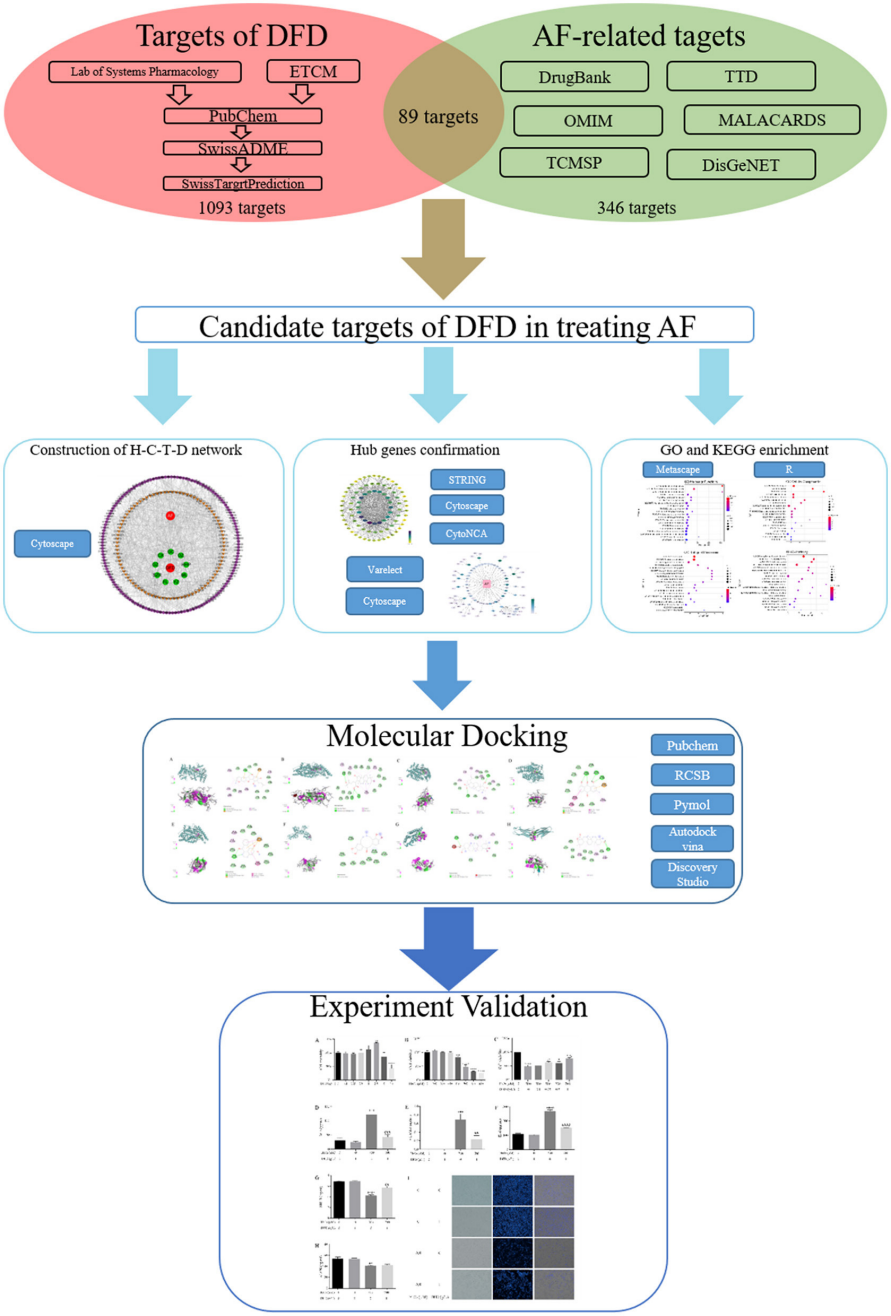
The flow chart of this work.

Network pharmacology is an interdisciplinary subject, its formation and development mainly benefit from artificial intelligence and big data analysis (12). The primary advantage of the network pharmacology is to emphasize the integrity, systemic, and biological network of the research object (13), which promotes the transformation of traditional Chinese medicine research method from the “one target, one drug” to “multiple target and multi-components,” thereby the molecular association between the multiple components and multiple targets could be analyzed. This feature more closely coincides with the concept of traditional Chinese medicine (14), which emphasizes the systemic and integrity in the progress of diagnosis and therapy (15, 16). With the increasing influence and application of network pharmacology, more and more TCM typical prescriptions have been explained at the molecular mechanism level and promoted in clinical practice (17). Here, we explored the potential mechanism of DFD in treating AF using network pharmacology and molecular docking integration strategy (Figure 1) (18, 19). At first, we obtained the bioactive compounds and potential targets of DFD in the treatment of AF. Then, the protein-protein interaction (PPI) of potential targets was constructed. Next, the functional enrichment analysis of potential targets and protein complexes was conducted. Meanwhile, the interactions between the bioactive compounds and key targets were visualized using molecular docking.

## Methods

### Acquisition of Bioactive Compounds and Potential Targets of DFD

At first, the compounds of each herb in DFD were collected from the Traditional Chinese Medicine Systems Pharmacology database and analysis platform (TCMSP) (20), Encyclopedia of Traditional Chinese Medicine (ETCM) (21), and the published literature. Then, the structures of each compound were obtained from PubChem (22). Next, the absorption, distribution, metabolism, and excretion (ADME) of each compound were detected using SwissADME with Gastrointestinal absorption (GA) and drug-likeness (DL) (23, 24), compounds with both high possibility of GA and DL were defined as bioactive compounds (9). At last, bioactive compounds were adopted for potential target screening using SwissTargetPrediction (25).

### Therapy Targets of AF

Taking the “atrial fibrillation” or “Fibrillation, Atrial” as keywords, we surveyed the therapy targets of AF from web-based datasets, including DrugBank (26), Therapeutic Target Database (TTD) (27), MalaCards (28), TCMSP (20), DisGeNET (29), and Online Mendelian Inheritance in Man (OMIM) (30). The species were limited to “human” or “*Homo sapiens*.” Among the results, the format of candidate therapy targets in DrugBank was transformed to a gene symbol using UniProt (31), the targets with determined proofs were adopted for further analysis in MalaCards (32), and the top 10% scores targets were adopted in DisGeNET. After the removal of duplicates, all targets were standardized using UniProt.

### Construction of Herb-Compound-Target-Disease Network

Based on the collected data, we constructed the herb-compound-target-disease (H-C-T-D) network using Cytoscape (version 3.8.0) to showcase the correlation between bioactive compounds and potential targets, moreover, to display the shard targets between DFD and AF (33). The shard targets were applied for further protein interaction exploration and functional enrichment analysis.

### Protein-Protein Interaction Exploration

PPI is one of the cores of cellular processing, which makes the interactions of proteins clear and helps with the functional explanation of potential protein complexes or functional modules (34). In this study, shared targets were applied for PPI exploration using STRING (34), a web database providing online analysis of PPI. The organism was set as “*homo sapiens*,” and the disconnected nodes were hidden. Then, the results were processed using Cytoscape (33), the CytoNCA plugin was used for the hub gene screening (35) and the MCODE plugin was employed for the investigation of functional protein complexes (36). Besides, to survey the hub gene more exactly, we utilized the VarElect dataset, an online tool that could point out the likelihood of genes to be related to certain disorders with scores (37), for the relevance analysis between the gene symbol of shared targets and AF.

### Functional Enrichment Analysis

In this study, functional enrichment analysis mainly includes the analysis for Gene Ontology (GO) resource and Kyoto Encyclopedia of Genes and Genomes (KEGG) dataset (38, 39), which contribute to the functional interpretation of genes and enable discoveries. Using Metascape, a web-based tool providing the analysis of systems-level datasets for GO, KEGG, and other datasets (40), we conducted the functional enrichment analysis of shared targets and the functional protein complexes found in the PPI network, GO terms or KEGG pathways with both *P*-value < 0.01 and enriched with more than 3 genes were considered significantly enrichment analysis. The results were visualized using the *ggplot2* package in R (41).

### Molecular Docking

The intersection of hub genes and high relevance genes were selected as candidate genes for molecular docking. The structures of the receptors were obtained from the RCSB dataset (42), all proteins were removed water molecule using Pymol (43), added polar-hydrogen, and calculated gasteiger using AutodockTools (version 1.5.6) (44). Then, the molecular docking was conducted using Autodock vina (45), and the results were processed with Discovery Studio (version 16.1) (46).

### Experimental Validation

#### Reagents

Hydrogen peroxide (H_2_O_2_) was provided by The Affiliated Traditional Chinese Medicine Hospital of Southwest Medical University (Luzhou, China). DFD was made into freeze-dried powder.

#### Cell Culture

HL-1 cells were obtained from Zhong Qiao Xin Zhou Biotechnology (Shanghai, China) and cultured in dulbecco's modified eagle medium (Gibco, Billings, MT, USA) with 10% fetal bovine serum (Biological Industries). Cells were cultured at 37°C in an atmosphere of 5% CO_2_ for all experiments.

#### Cell Counting KIT-8

HL-1 cells were seeded into 96-well micro-test plates. After incubation for 24 h, HL-1 cells were exposed to H_2_O_2_ or/and DFD. Then, 10 μL CCK-8 solution (Dojindo Molecular Technologies, Inc) were added to each well, and cells were incubated for another 1 h. The absorbance at 450 nm was measured by the SYNERGY microplate reader (BioTek Instruments, Inc, USA).

#### Enzyme-Linked Immunosorbent Assay

HL-1 cells were incubated with H_2_O_2_ for 24 h and then treated with DFD for another 24 h. Supernatants were harvested to determine the levels of ACE, eNOS, SIRT1, VCAM-1, and IL-6 using ELISA kits (Elabscience, Wuhan, China) according to the manufacturers' instructions.

#### Hoechst 33342 Stain

Nuclei were stained with Hoechst 33342 (Beyotime, Shanghai, China) according to the manufacturer's instructions. The cells were then visualized under a fluorescence microscope (EVOS FL Auto, Life Technologies, USA).

## Results

### Bioactive Compounds and Potential Targets

A total of 434 compounds of DFD were surveyed from TCMSP, ETCM, and literature. In detail, we included 92, 76, 73, 70, 38, 36, 19, 18, and 12 compounds in *Glycyrrhiza uralensis Fisch, Ziziphus jujuba Mill, Ligusticum chuanxiong, Albizia julibrissin Durazz., Poria cocos (Schw.)Wolf* , *Semen Ziziphi Spinosae, Cinnamomum cassia (L.) J.Presl, Polygala tenuifolia Willd*, and *Crassostrea Gigas*, respectively. Interestingly, several herbs contain the same compounds, as shown in Table 1. After the screening of ADME of each compound using SwissADME, a total of 168 compounds were validated possessing both high possibilities of GA and DL, details of screening are showcased in Supplementary Table 1. Compounds which were bioactive were applied for potential target prediction using SwissTargetPrediction, after the removal of duplicates, a total of 1,093 targets were collected, as shown in Supplementary Table 2.

**Table 1:** The information of shared compounds.

**Compound**	**PubChem ID**	**Herbal**
Quercetin	5280343	*Albizia julibrissin Durazz, Ziziphus jujuba Mill., Glycyrrhiza uralensis Fisch*
Protocatechuic Acid	72	*Ligusticum chuanxiong, Cinnamomum cassia (L.) J.Presl*
Cetylic Acid	985	*Ligusticum chuanxiong, Poria Cocos (Schw.) Wolf, Ziziphus jujuba Mill*.
3-O-trans ferulylquinic acid	445858	*Ligusticum chuanxiong, Ziziphi Spinosae Semen*
Peroxyergosterol	5351516	*Ligusticum chuanxiong, Poria Cocos (Schw.) Wolf*
(S)-Coclaurine	160487	*Semen Ziziphi Spinosae, Ziziphus jujuba Mill*.
Lysicamine	122691	*Semen Ziziphi Spinosae, Ziziphus jujuba Mill*.

### Identification of Potential Therapy Targets

To identify the therapy-related targets of AF, we surveyed DrugBank, TTD, MalaCards, TCMSP, DisGeNET, and OMIM with the cutoff mentioned above, after the removal of duplicates, a total of 346 targets were obtained. Taking the intersection between potential targets of DFD and therapy targets of AF, 89 shared targets were identified as potential therapeutic targets of DFD in treating AF.

### Construction of H-C-T-D Network

**Figure 2:**
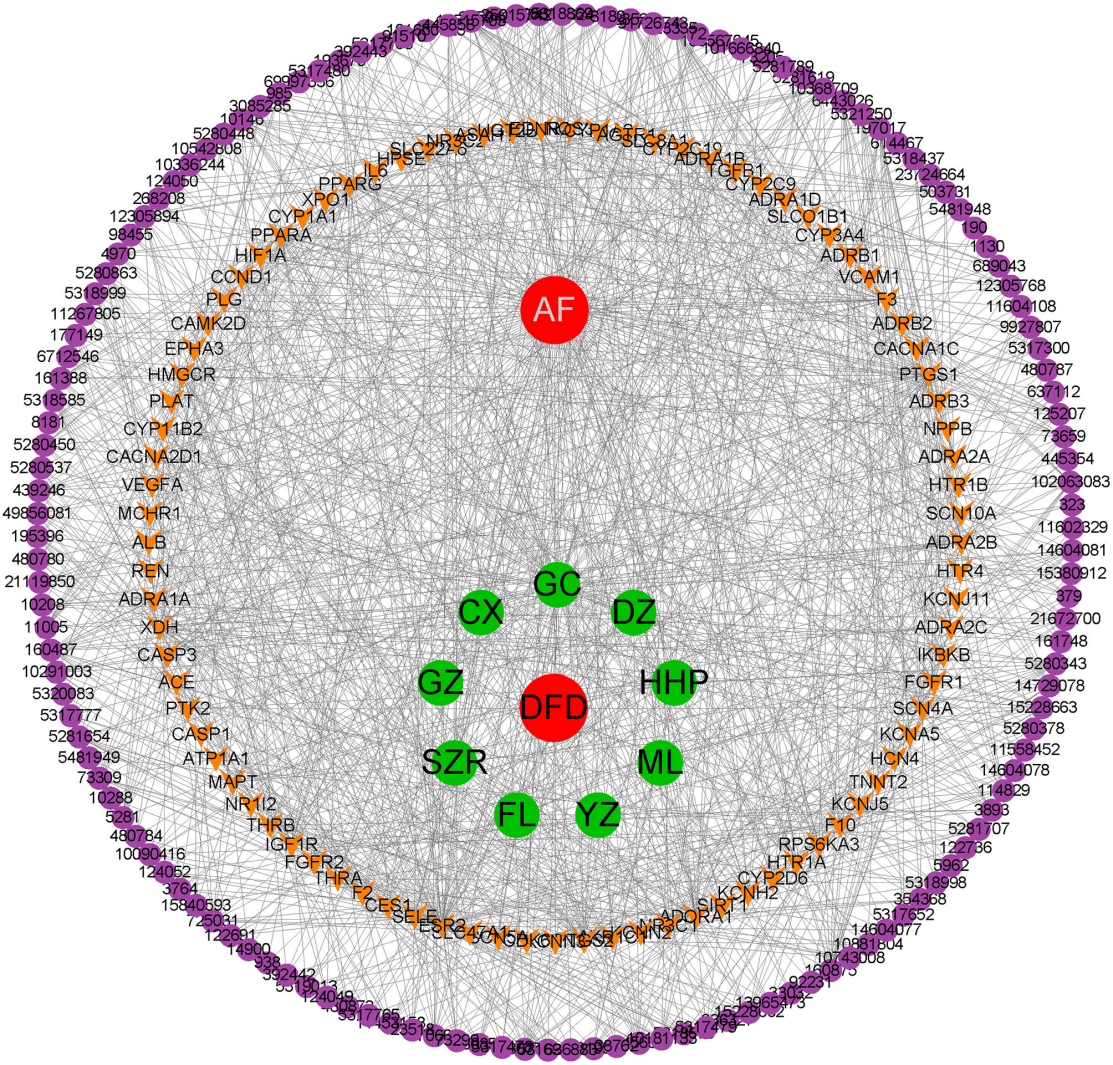
The H-C-T-D network.

Based on the obtained data, we constructed the H-C-T-D network using Cytoscape. The network was composed of 242 nodes, including DFD, VA, 9 herbals, 142 bioactive compounds, and 89 common targets, and 1,237 edges were connected among 242 nodes (Figure 2). The network preliminarily revealed the complex mechanism of multiple compounds and multiple targets in the treatment of AF.

### Identification of Hub Genes

**Figure 3:**
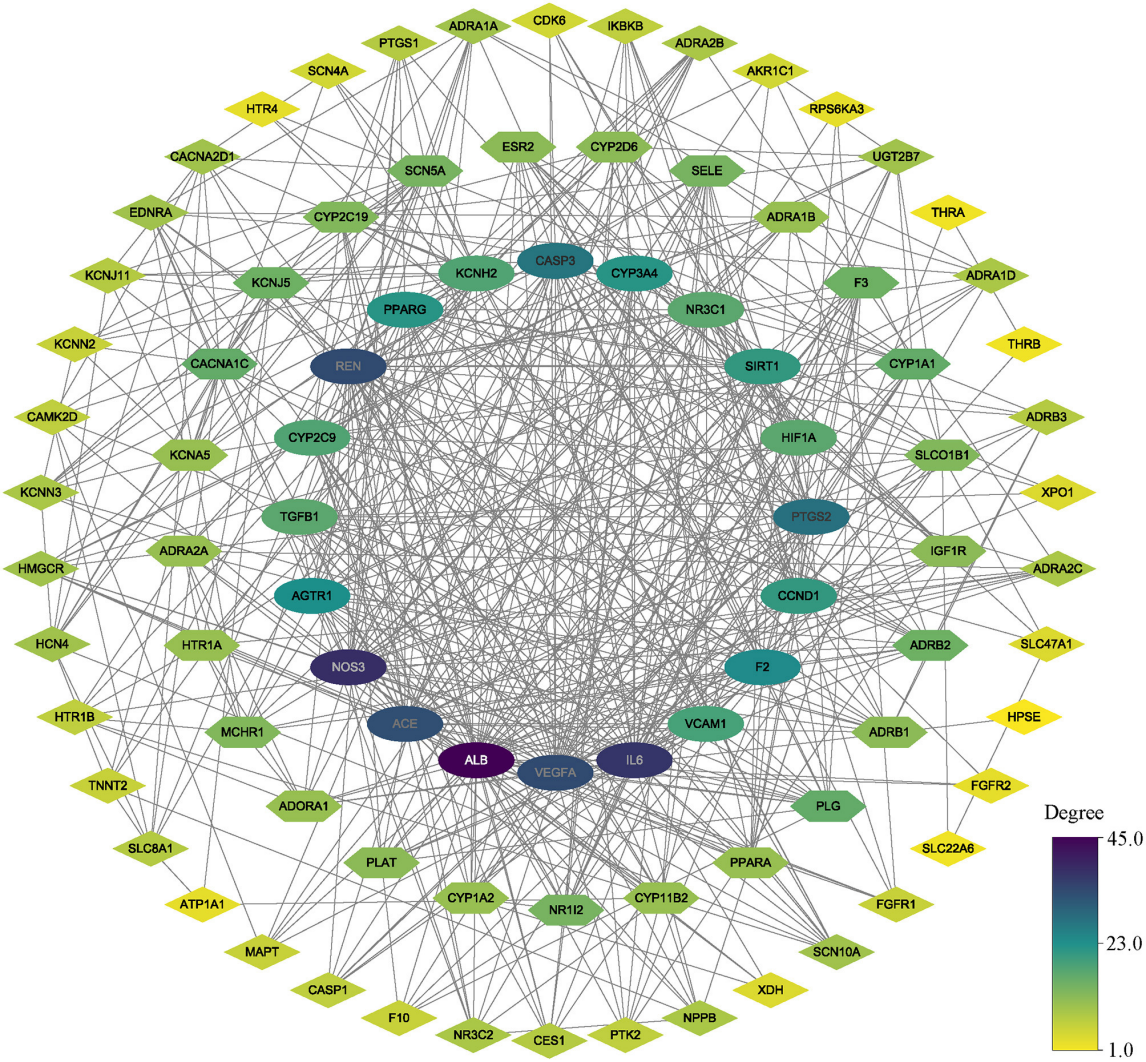
The PPI network. All nodes colored according to the degree value. oval nodes on the inner circle indicate hub targets.

**Figure 4:**
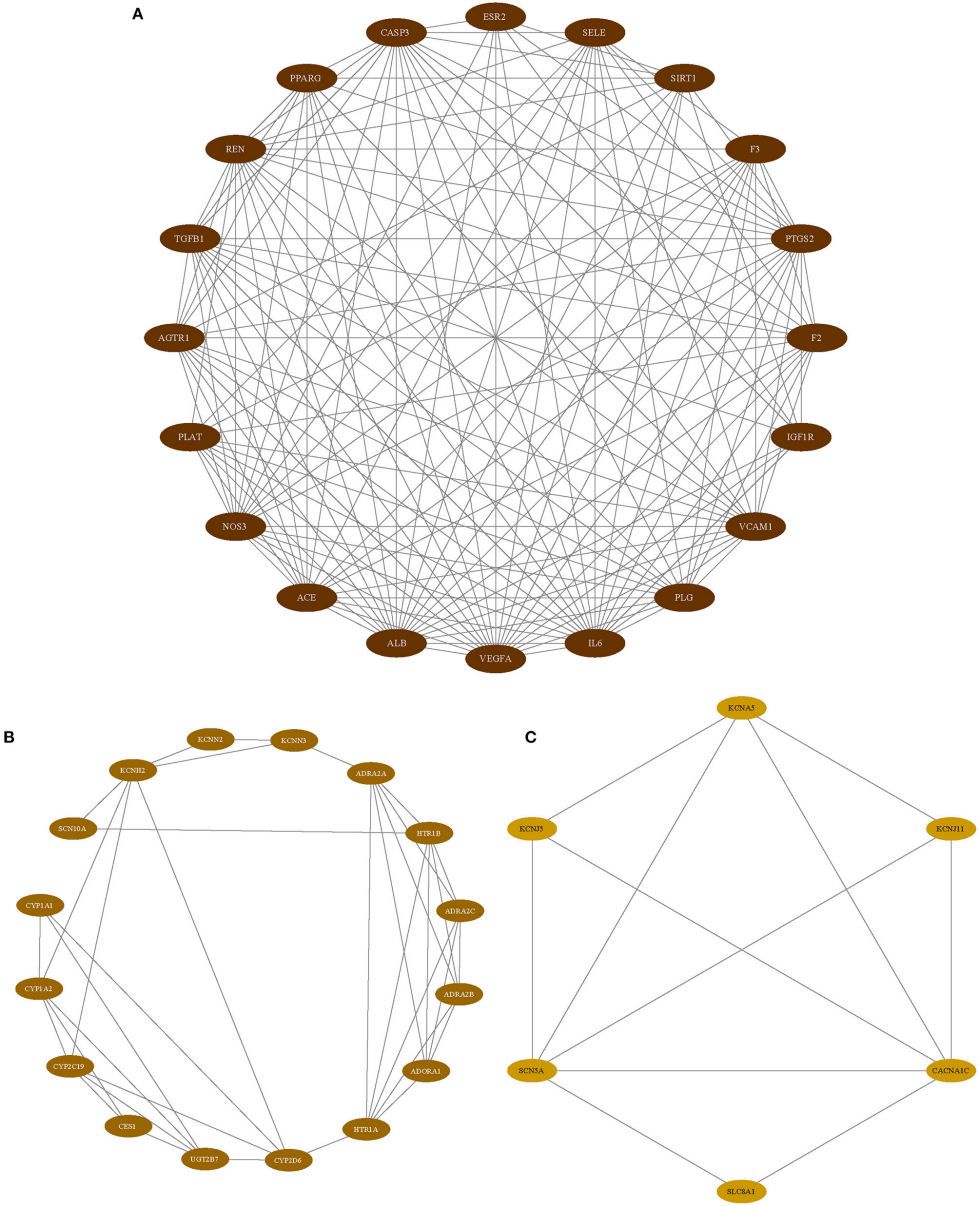
The functional protein complexes. **(A)** Complex A. **(B)** Complex B. **(C)** Complex C.

As we mentioned above, PPI analysis is pivotal to the explaining of the protein interactions and functional analysis of potential protein complexes or functional modules. We analyzed the PPI analysis using STRING, the results were uploaded to Cytoscape for hub gene identification and potential functional protein complexes detection, the hub gene was selected with the value of 75% degree, which was calculated using CytoNCA plugin, the details of degree value were shown in Supplementary Table 3, and the PPI network was reconstructed according to the value of degree (Figure 3). Besides, the MCODE plugin has surveyed three functional protein complexes, which were named as complex A, complex B, and complex C (Figure 4). To determine the hub genes more exactly, we conducted the relevance analysis using VarElect (Figure 4), the results suggested that 47 genes were related to the therapy of AF directly, and 42 genes related to the therapy of AF indirectly (Supplementary Table 4). After taking the intersection of hub genes and high relevance genes and checking related published literature, 6 genes, including NOS3, ACE, SIRT1, IL-6, VCAM-1, and KCNH2, of DFD in treating AF were finally determined.

### Functional Enrichment Analysis

**Figure 5:**
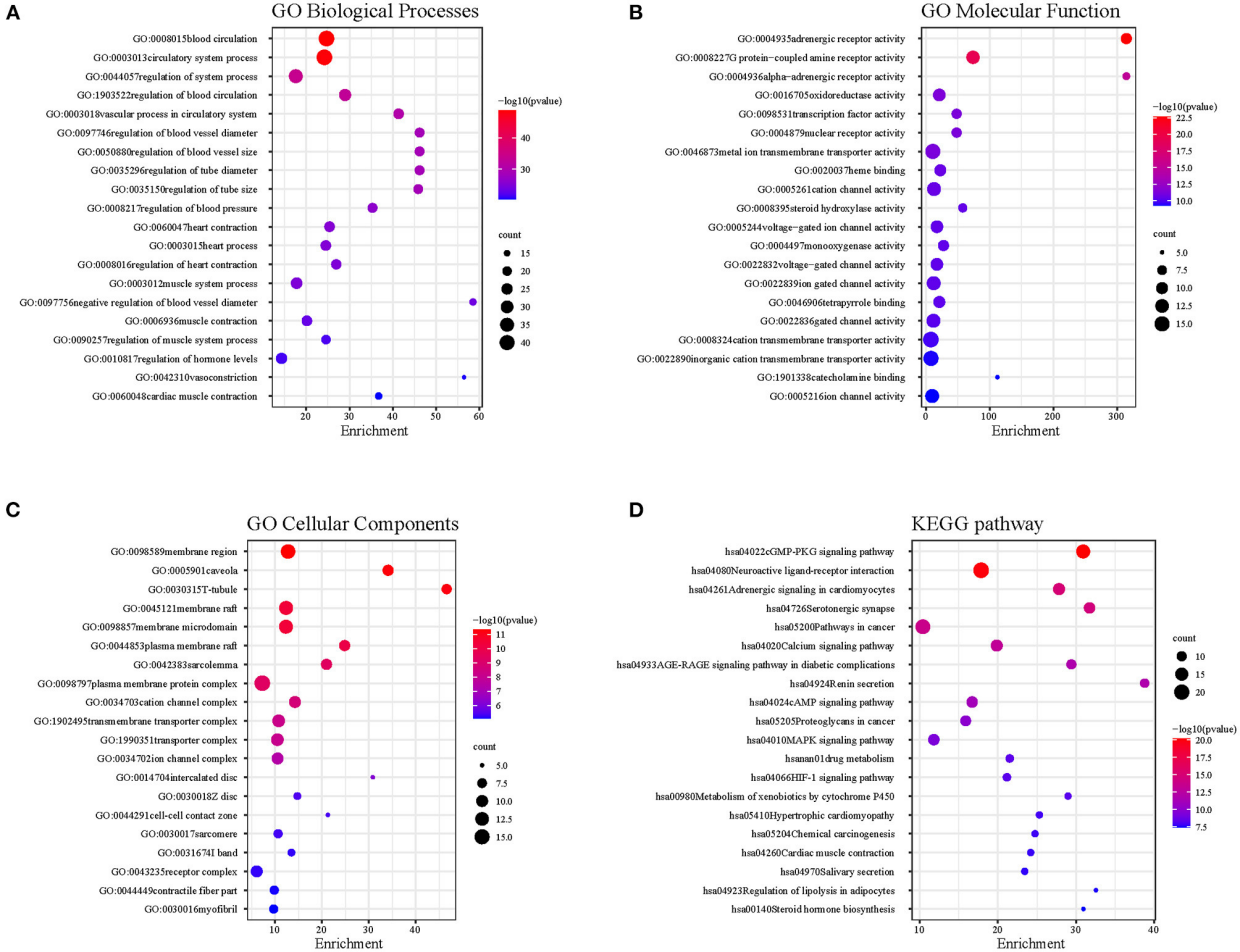
The functional enrichment analysis. **(A)** GO Biological Processes. **(B)** GO Molecular Function. **(C)** GO Cellular Components. **(D)** KEGG pathway.

The common targets between DFD and AF were performed functional enrichment analysis using Metascape, including GO Biological Processes, GO Molecular Function, GO Cellular Components, and KEGG pathway. The results were visualized using the *ggplot* package in R (Figure 5). The results suggested that the shared targets were involved in various GO Biological Processes including regulation of blood circulation, the vascular process in the circulatory system, regulation of blood pressure, heart contraction, heart process, muscle system process, regulation of hormone levels, and cardiac muscle contraction (Figure 5A). numerous GO molecular functions involved adrenergic receptor activity, G protein-coupled amine receptor activity, alpha-adrenergic receptor activity, oxidoreductase activity, reduction of molecular oxygen, transcription factor activity, cation channel activity, and voltage-gated ion channel activity (Figure 5B). Numerous GO cellular components were involved in membrane region, T-tubule, plasma membrane raft, sarcolemma, cation channel complex, ion channel complex, intercalated disc, Z disc, cell-cell contact zone, I band, contractile fiber part, and myofibril (Figure 5C), and numerous KEGG pathways were involved in neuroactive ligand-receptor interaction, cGMP-PKG signaling pathway, adrenergic signaling in cardiomyocytes, serotonergic synapse, calcium signaling pathway, calcium signaling pathway, and cAMP signaling pathway (Figure 5D).

**Figure 6:**
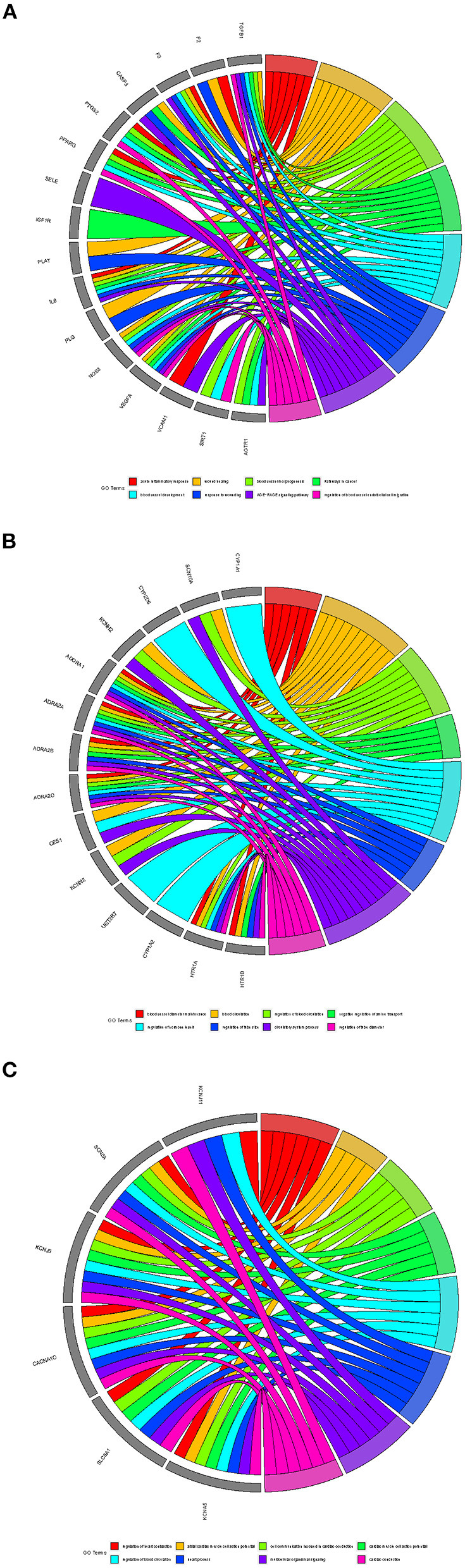
The functional analysis of protein complexes. **(A)** Complex A. **(B)** Complex B. **(C)** Complex C.

Besides, three potential functional protein complexes were applied for functional enrichment analysis, respectively. As showcased in Figure 6. Complex A was involved in the AGE-RAGE signaling pathway, wound healing, acute inflammatory response, pathways in cancer, response to wounding, blood vessel morphogenesis, regulation of blood vessel endothelial cell migration, and blood vessel development. Complex B was involved in blood circulation, circulatory system process, regulation of blood circulation, negative regulation of amine transport, regulation of hormone levels, regulation of tube diameter, blood vessel diameter maintenance, and regulation of tube size. Complex C was involved in cardiac conduction, multicellular organismal signaling, cell communication involved in cardiac conduction, regulation of heart contraction, cardiac muscle cell action potential, heart process, regulation of blood circulation, and atrial cardiac muscle cell action potential.

### Molecular Docking

**Figure 7:**
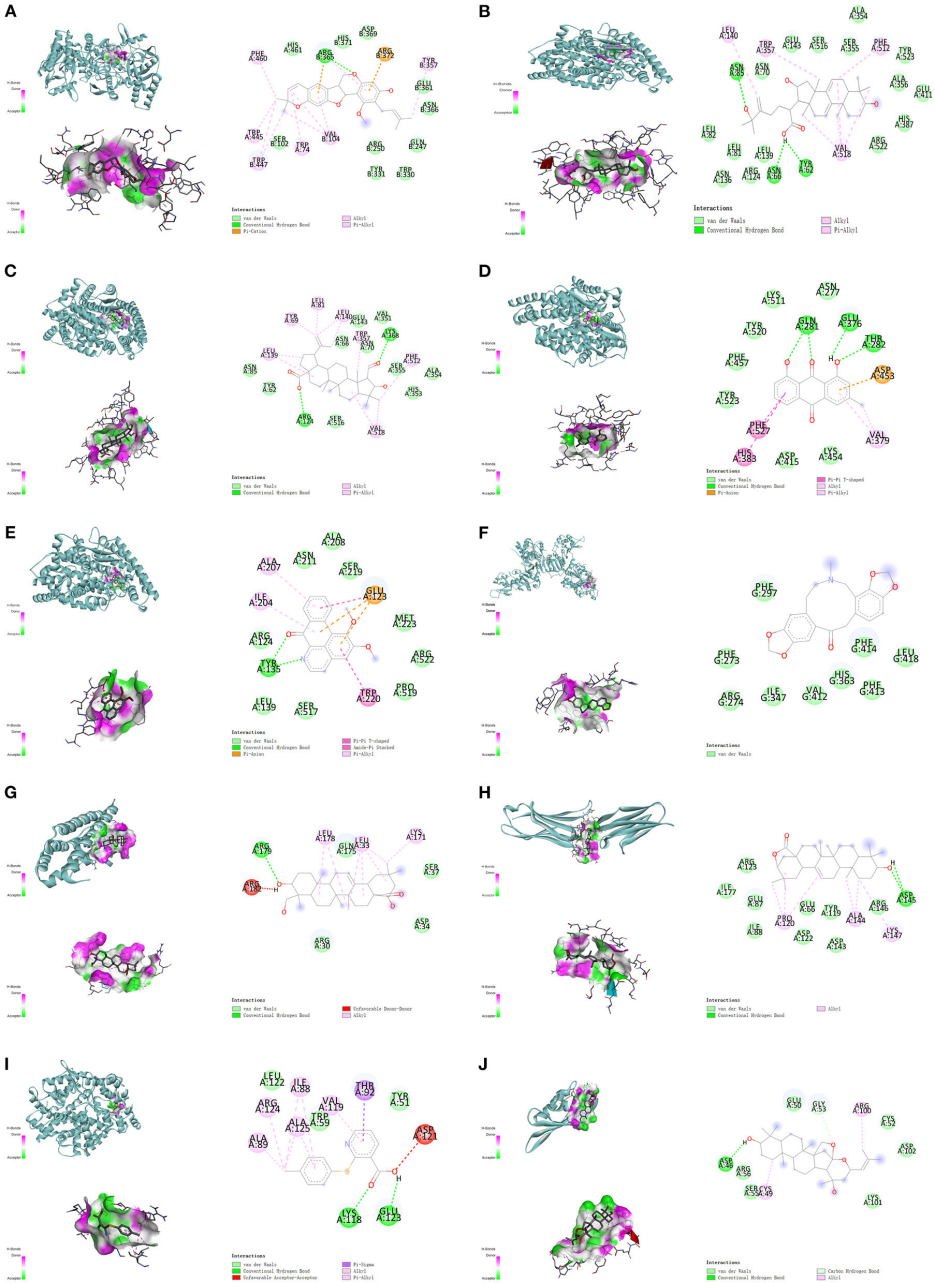
The top 10 binding energy molecular docking models. **(A)** Kanzonol F and NOS3. **(B)** 25-Hydroxy-3-epidehydrotumulosic acid and ACE. **(C)** Colubrinic acid and ACE. **(D)** Chrysophanol and ACE. **(E)** Lysicamine and ACE. **(F)** Fumarine and SIRT1. **(G)** Hederagenin and IL-6. **(H)** Machaerinic acid lactone and VCAM-1. **(I)** JL1 and ACE. **(J)** Jujubogenin and KCNH2.

After the checking of related published literature, 6 proteins contain NOS3, ACE, SIRT1, IL-6, VCAM-1, and KCNH2 were included in the molecular docking. The corresponding structures were obtained from RCSB (Supplementary Table 5). The details of molecular docking are shown in Table 2, the lower the binding energy, the more stable the docking model. The results suggested that the corresponding compounds could interact with the 6 selected proteins against AF, and the lowest 10 binding energy docking models were shown in Figure 7.

**Table 2:** Details of molecular docking.

**Compound**	**PubChem ID**	**Target**	**PDB ID**	**Binding Energy (kcal/mol)**
Kanzonol F	101666840	NOS3	1M9J	−9.9
25-Hydroxy-3-epidehydrotumulosic acid	10368709	ACE	1O86	−9.8
Colubrinic acid	21672700	ACE	1O86	−9.2
Chrysophanol	10208	ACE	1O86	−9
Lysicamine	122691	ACE	1O86	−8.3
Fumarine	4970	SIRT1	4IG9	−7.9
Hederagenin	73299	IL-6	1ALU	−7.6
Machaerinic acid lactone	21594250	VCAM-1	1IJ9	−7.6
JL1	725031	ACE	1O86	−7.2
Jujubogenin	15515703	KCNH2	1BYW	−6.9

*The Binding energy refers to the strength of the binding between the receptor and the ligand, the lower the binding energy, the more stable the docking module*.

As is shown above, the results suggested that Kanzonol F formed binding energy of 9.9 kcal/mol with NOS3, which mainly through the conventional hydrogen bond formed with ARG365, the van der Waals force formed with HIS461, HIS371 ASP369, SER102, and others, the Pi-cation interaction with ARG372 (Figure 7A). 25-Hydroxy-3-epidehydrotumulosic acid formed binding energy of −9.8 kcal/mol with ACE, mainly through the conventional hydrogen bond, Vander Waals forces, and Pi-Alkyl interaction (Figure 7B). Colubrinic acid formed binding energy of −9.2 kcal/mol with ACE, mainly through a conventional hydrogen bond, Vander Waals forces, and Pi-Alkyl interaction (Figure 7C), Chrysophanol formed binding energy of −9.0 kcal/mol with ACE, mainly through a conventional hydrogen bond, van der Waals forces Pi-Pi T-Shaped interaction, and Pi-Alkyl interaction (Figure 7D), Lysicamine formed binding energy of −8.3 kcal/mol with ACE, mainly through a conventional hydrogen bond, Vander Waals forces, Pi-Pi T-Shaped interaction, Amide-Pi Stacked interaction and Pi-Alkyl interaction (Figure 7E). Fumarine formed binding energy of −7.9 kcal/mol with SIRT1, mainly through van der Waals force (Figure 7F). Hederagenin formed binding energy of −7.6 kcal/mol with IL-6, mainly through conventional hydrogen bond and Vander Waals force (Figure 7G). Machaerinic acid lactone formed binding energy of −7.6 kcal/mol with VCAM-1, mainly through conventional hydrogen bond and Vander Waals force (Figure 7H). JL1 formed binding energy of −7.2 kcal/mol with ACE, mainly through a conventional hydrogen bond, Vander Waals forces, Pi-Pi T-Shaped interaction, Amide-Pi Stacked interaction, Pi-anion interaction, and Pi-Alkyl interaction (Figure 7I), and Jujubogenin formed binding energy of −6.9 kcal/mol with KCNH2, mainly through a conventional hydrogen bond, Vander Waals force and carbon-hydrogen bond (Figure 7J).

### Experimental Validation

**Figure 8:**
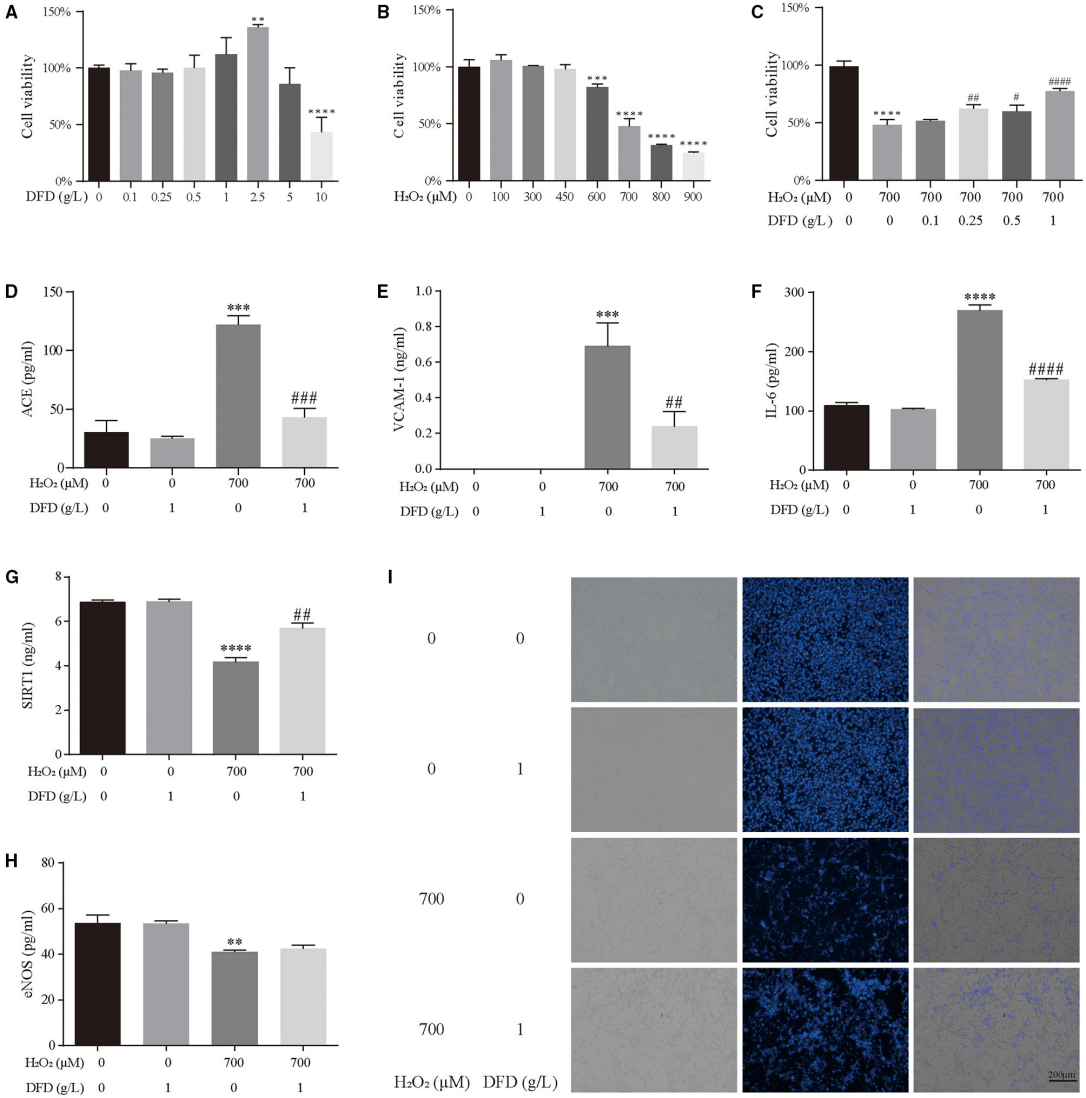
Experimental validation. **(A)** The effect of DFD on the cell viability of HL-1 cells. **(B)** The effect of H_2_O_2_ on the cell viability of HL-1 cells. **(C)** The effect of DFD and H_2_O_2_ on the cell viability of HL-1 cells. **(D)** ACE. **(E)** VCAM-1. **(F)** IL-6. **(G)** SIRT1. **(H)** eNOS. **(I)** Hoechst 33342 stain. **P < 0.01 compared with control group, ***P < 0.001 compared with control group, and ****P < 0.0001 compared with control group; ^#^*P* < 0.05 compared with H_2_O_2_ group, ^##^*P* < 0.01 compared with H_2_O_2_ group, ^###^*P* < 0.001 compared with H_2_O_2_ group, and ^####^*P* < 0.0001 compared with H_2_O_2_ group.

First, we detected the effect of different doses of DFD and H_2_O_2_ on the viability of HL-1 cells using the CCK-8 assay. Compared with the control group, there was no significant changes in the viability of HL-1 cells when 0.1–1.0 g/L DFD was administrated (Figure 8A). Compared with the group, there was 50% decrease in the viability of HL-1 cells when 700 μM H_2_O_2_ was administrated (Figure 8B). Interestingly, 0.25–1.0 g/L DFD could siginificantly reverse the viability of HL-1 treated with 700 μM H_2_O_2_ (Figure 8C). Therefore, we continued to use 1.0 g/L DFD and to 700 μM H_2_O_2_ to further validate the results obtained in the systematic pharmacologic analysis. We found that when 700 μM H_2_O_2_ was given, the levels of ACE, VCAM-1 and IL-6 in HL-1 cells increased, while SIRT1 and eNOS decreased (Figures 8D–H). Interestingly, these changes could be restored after 1.0 g/L DFD (Figures 8D–H). Hoechst 33342 stain indicated that H_2_O_2_ aggravated cell apoptosis, and DFD can play a good protective role (Figure 8I).

## Discussion

AF is the most common arrhythmia among clinical practice, owing to the control of cardiovascular diseases in recent years, AF has declined significantly, but it still brings adverse results to patients and a heavy burden to the medical system. DFD is a traditional Chinese herb mixture, and has been utilized to treat the phenotype of palpitations for a long time, and has good effectiveness (10). Our previous work suggests that using the therapy strategy of DFD combined with metoprolol could benefit ventricular arrhythmia patients more than using metoprolol only (10). Besides, using the whole-cell patch-clamp, we have detected that one mechanism of DFD in treating arrhythmia is suppressing Nav_1.5_ (11), and this work aims to detect the mechanism of DFD in treating AF in network pharmacology and molecular docking integration strategy and to provide a supplement therapy strategy of traditional Chinese medicine for AF.

After screening the related datasets, a total of 168 bioactive compounds, 1,093 potential targets of DFD, and 346 therapy-related targets of AF were collected. Then we constructed the H-C-T-D network, which has demonstrated the highly complex interactions among herbs, targets, and diseases and initially revealed the potential mechanism of DFD in treating AF and determined 89 candidate targets of DFD in treating AF. Next, to analyze the hub gene and potential functional protein complex of DFD in treating AF, the 89 candidate targets were applied to PPI network analysis and relevance analysis, the results filtered out 6 hub genes and 3 functional complexes. In terms of the 6 hub genes, NOS3 could produce nitric oxide, which is involved in the progress of vascular smooth muscle relaxation, coronary vessels angiogenesis, and blood clotting. Besides, NOS3 mediates the stretch dependence of Ca2^+^ release in cardiomyocytes, which is pivotal protection in the prevention of arrhythmia (47, 48). A study has mentioned that a higher expression level of NOS3 in Chinese hamster ovary cells could restore the duration of the plateau phase of action potentials and increase the bioavailability of nitric oxide, which is Impaired in AF patients (49). Besides, a study points out that compared to sheep without AF, the transcript level of NOS3 was attenuated in sheep models of persistent AF, which was probably caused by increased NADPH oxidase activity and a violent oxidative reaction (50). IL-6 has participated in the progress of immune response and anti-inflammatory activity (51). Recently, clinical proofs have highlighted the mechanistic link between the expression level of IL-6 and the development and prognostic of AF (52), the results suggest a declined expression of IL-6 may spur a lower incidence of AF (53), further mechanism studies have illustrated that the mutation of IL-6 and inflammatory reactions related to IL-6 are pivotal in the progress of atrial remodeling, which is one of the cores of the development of AF (54–57). Similarly, recent studies of rats suggested the declined release of IL-6 is related to the reduction of atrial fibrosis and a lower incidence of AF (58, 59). ACE is one of the critical regulators of systemic vascular resistance, blood volume, and cardiovascular homeostasis. Clinical evidence suggested that the treatment of ACE blockers can reduce the incidence and the recurrence of AF (60–62). A review figures out the plasma concentration of IL-6 and ACE has high sensitivity in the diagnosis of AF and could be the ideal candidate biomarkers of AF (63). A study suggested the mutation of ACE is associated with increased cardiac fibrosis, adverse cardiovascular diseases and may cause the failure of anti-AF treatment (64). Mechanism studies suggested that ACE inhibitors could prevent AF by affecting the inflammation reaction, reducing the accumulation of the epicardial adipose tissue, and decrease the incidence of arrhythmia via reverse remodeling (65). KCNH2 is one of the subunits of the voltage-gated inwardly rectifying potassium channel. Which mediates the rapidly activating component of the delayed rectifying potassium current (I_Kr_) in the heart (66, 67). The mutation of KCNH2 is associated with familial AF (68) and is companied by a higher incidence of AF in clinical (69). Meanwhile, KCNH2 is part of the gene network regulated by transcription factor ETV1, the upregulation of which is linked to the development of AF (70). Recently, a gene therapy trial produced an inspiring result, that gene therapy with KCNH2 could eliminate AF via prolonging atrial action potential duration (71). Numerous studies suggest that SIRT1, VCAM, VEGFA, TGFB1, CAP3, and CACNA1C are involved in the progress of the occurrence, development, and treatment of AF (72–76).

In further mechanism exploration, the results of GO analysis suggest that DFD involves the biological processes closely related to AF, including inflammation, heart modeling, adrenergic receptor activity, transportation of cation, and cell-cell contact, which promote the mechanism explaining of DFD in treating AF. The results of the KEGG pathway analysis suggest DFD is involved in the cGMP-PKG signaling pathway via the regulation of ADRA, ADRB, and NOS3. In cardiomyocytes, PKG could suppress Ca^2+^ conductance, NFAT activation, myocardial damage, and myocyte hypertrophic responses (77). Besides, PKG also opens mitochondrial ATP-sensitive K^+^ channels and subsequent release of ROS triggers cardioprotection (77, 78). DFD also involves two linked pathways including adrenergic signaling in cardiomyocytes and the cAMP signaling pathway via the regulation of ADRA1, ADRB, CACNA1, and SCN5A. Pathway of adrenergic signaling in cardiomyocytes mainly participates in the progress of cardiomyocyte hypertrophy and apoptosis (79–81), and the cAMP signaling pathway regulates important physiologic processes including metabolism, gene transcription, calcium homeostasis, and muscle contraction (82, 83). The calcium signaling pathway is another significant pathway related to the occurrence of AF development that DFD involves. It is pivotal in the regulation of muscle contraction, calcium ion binding, transport, and cell metabolism (84, 85). Nevertheless, numerous pathways cooperatively correlated with the initiation, maintenance, and recurrence of AF could be found in the enrichment analysis results. The results of enrichment analysis of functional protein complexes suggested that complex A involves the progress of acute inflammatory response, blood vessel morphogenesis, regulation of blood vessel endothelial cell migration, and blood vessel development, Complex B involves the regulation of blood circulation, circulatory system process, and hormone levels, which means complex A and B have a great potential in the regulation of contractile remodeling and structural remodeling, which are the core mechanisms of AF (86). Complex C involves the progress of cardiac conduction, multicellular organismal signaling, cell communication involved in cardiac conduction, regulation of heart contraction, cardiac muscle cell action potential, and atrial cardiac muscle cell action potential, the results suggest that complex C could regulate the electrical remodeling.

Nevertheless, this study has some limitations. First, although we hope to detect all bioactive compounds, Os Draconis, Crassostrea Gigas, has only several references that reported several compounds, and were excluded for its poor quality of GA and DL, this is inconsistent with the tranquilizing mind function harbored by Os Draconis and Crassostrea Gigas mentioned in traditional Chinese medicine. Moreover, this study was designed to explore the potential mechanism based on network pharmacology and molecular docking integration strategy. Same as other network pharmacology analysis, although taking the intersection of the potential targets of DFD and the therapy-related targets of AF in this study makes the result of virtual screening more reliable, some targets out of the intersection may be pivotal to the therapy or diagnosis of AF has been ignored, which is a common and inevitable issue in virtual screening. Finally, we firstly explored the potential mechanism of DFD against AF based on network pharmacology and molecular docking integration strategy with more solid experimental studies. Although our previous clinical study have proved that DFD is safe and effective in the treatment of arrhythmias (10), and *in vivo* experiments have also verified part of the antiarrhythmic mechanism of DFD (11), more experiments are still needed to verify our findings.

## Conclusions

DFD could be utilized in treating AF through a complicated mechanism, including interactions between related compounds and targets, as predicted by network pharmacology and molecular docking. This work confirmed that DFD could be used for the treatment of AF and promoted the explanation of DFD for AF in the molecular mechanism.

## Data Availability Statement

The datasets presented in this study can be found in online repositories. The names of the repository/repositories and accession number(s) can be found in the article/Supplementary Material.

## Author Contributions

YL, BL, and L-ZZ conceived, designed, and planned the study. BL, YL, and X-RW acquired and analyzed the data. BL and WC interpreted the results. YL and W-SL-H conducted the *in vitro* experiment. YL and BL drafted the manuscript. BL and L-ZZ contributed to the critical revision of the manuscript. All authors contributed to the article and approved the submitted version.

## Funding

This work was funded by General Project of Sichuan Provincial Administration of Traditional Chinese Medicine (2021MS114) and Research and Practice Innovation Plan for Postgraduates of Jiangsu, China (KYCX21_1641).

## Conflict of Interest

The authors declare that the research was conducted in the absence of any commercial or financial relationships that could be construed as a potential conflict of interest.

## Publisher's Note

All claims expressed in this article are solely those of the authors and do not necessarily represent those of their affiliated organizations, or those of the publisher, the editors and the reviewers. Any product that may be evaluated in this article, or claim that may be made by its manufacturer, is not guaranteed or endorsed by the publisher.
