# Supplementary material for: Potential Mechanism of Dingji Fumai Decoction Against Atrial Fibrillation Based on Network Pharmacology, Molecular Docking, and Experimental Verification Integration Strategy
Source: Front Cardiovasc Med. 2021 Nov 11;8:712398. doi: 10.3389/fcvm.2021.712398 (PMC8631917; doi:10.3389/fcvm.2021.712398)
Supplement: Supplementary file 1 [file Table_1.pdf]

Table S1. Details of qualified compounds in various herbs.

PubChem ID	Ingredients	SwissADME					
		GA	DL				
			lipinski	ghose	veber	egan	muegge
5280343	Quercetin	high	√	√	√	√	√
72	Protocatechuic Acid	high	√		√	√	
985	Cetylic Acid	high	√	√		√	
689043	Caffeic Acid	high	√	√	√	√	
5321250	Senkyunolide G	high	√	√	√	√	√
15138552	Senkyunolide N	high	√	√	√	√	√
91726743	Senkyunone	high	√		√	√	
92231	Spathulenol	high	√	√	√	√	
1174	Uracil	high	√		√	√	
1183	Vanillin	high	√		√	√	
122736	Wallichilide	high	√		√	√	√
522266	Spathulenol	high	√	√	√	√	
10288	Chrysophanic Acid	high	√	√	√	√	√
10208	Chrysophanol	high	√	√	√	√	√
14900	Dilinoyl Palmitoyl Glyceride, Glycerol1-Monopalmitate	high	√	√		√	
8762	Ethylpentadecanoate	high	√			√	
8181	Methyl Hexadecanate	high	√			√	
23518	Methyl Pentadecanoate	high	√	√		√	
161748	Myricanone	high	√	√	√	√	√
445354	Retinol	high	√	√	√	√	
445858	3-O-trans ferulylquinic acid	high	√	√	√	√	
98455	Stepharine	high	√	√	√	√	√
5280450	Linoleic Acid	high	√		√		
11005	Myristic Acid	high	√	√		√	
5281	Stearic Acid	high	√		√		
222656	Malic Acid	high	√		√	√	
938	Nicotinic Acid	high	√		√	√	
6267	Asparagine	high	√		√	√	
470606	Alphitolic Acid	high	√		√		
445638	Palmitoleic Acid	high	√	√		√	
160875	Asimilobine	high	√	√	√	√	√
289	Catechol	high	√		√	√	
5281707	coumestrol	high	√	√	√	√	√
21672700	Colubrinic Acid	high	√		√		
73659	2Î‘,3Î’-Dihydroxyolean-12-En-28-Oic Acid	high	√		√		
1130	Vitamin B1	high	√	√	√	√	√
5280462	Vomifoliol	high	√	√	√	√	√
6443026	Mauritine D	high	√			√	
2353	berberine	high	√	√	√	√	√
101650325	Ruvoside	high	√		√		√
6917970	Stepholidine	high	√	√	√	√	√
10146	Nuciferine	high	√	√	√	√	√
4970	Fumarine	high	√	√	√	√	√

5280537	Moupinamide	high	√	√	√	√	√
5351516	Peroxyergosterol	high	√		√		
160487	(S)-Coclaurine	high	√	√	√	√	√
122691	Lysicamine	high	√	√	√	√	
73299	hederagenin	high	√		√		
10181133	Cerevisterol	high	√		√	√	
10743008	(2R)-2-[(3S,5R,10S,13R,14R,16R,17R)-3,16-dihydroxy-4,4,10,13,14-pentamethyl-2,3,5,6,12,15,16,17-octahydro-1H-cyclopenta[a]phenanthren-17-yl]-6-methylhept-5-enoic acid	high	√		√		
3893	Lauric Acid	high	√	√	√	√	√
379	Caprylic Acid	high	√		√	√	
125207	Dodecenoic Acid	high	√	√	√	√	√
190	Adenine	high	√		√	√	
10368709	25-Hydroxy-3-Epidehydrotumulosic Acid	high	√		√	√	√
8180	Undecanoic Acid	high	√	√	√	√	
91510	Inermine	high	√	√	√	√	√
5280448	Calycosin	high	√	√	√	√	√
5280863	kaempferol	high	√	√	√	√	√
439246	naringenin	high	√	√	√	√	√
197678	(2S)-2-[4-hydroxy-3-(3-methylbut-2-enyl)phenyl]-8,8-dimethyl-2,3-dihydropyrano[2,3-f]chromen-4-one	high	√	√	√	√	
10291003	euchrenone	high	√	√	√	√	√
480784	glyasperin B	high	√	√	√	√	√
392442	glyasperin F	high	√	√	√	√	√
480859	Glyasperin C	high	√	√	√	√	√
5318679	Isotrifoliol	high	√	√	√	√	√
10881804	(E)-1-(2,4-dihydroxyphenyl)-3-(2,2-dimethylchromen-6-yl)prop-2-en-1-one	high	√	√	√	√	√
114829	DFV	high	√	√	√	√	√
15380912	kanzonols W	high	√	√	√	√	√
637112	(2S)-6-(2,4-dihydroxyphenyl)-2-(2-hydroxypropan-2-yl)-4-methoxy-2,3-dihydrofuro[3,2-g]chromen-7-one	high	√	√	√	√	√
5481948	Semilicoisoflavone B	high	√	√	√	√	√
5281619	Glepidotin A	high	√	√	√	√	√
442411	Glepidotin B	high	√	√	√	√	√
162412	Phaseolinisoflavan	high	√	√	√	√	√
5317768	Glypallichalcone	high	√	√	√	√	√
10542808	8-(6-hydroxy-2-benzofuranyl)-2,2-dimethyl-5-chromenol	high	√	√	√	√	√
5318999	Licochalcone B	high	√	√	√	√	√
49856081	licochalcone G	high	√	√	√	√	
5320083	Glycyrol	high	√	√	√	√	√
10090416	3-(2,4-dihydroxyphenyl)-8-(1,1-dimethylprop-2-enyl)-7-hydroxy-5-methoxy-coumarin	high	√	√	√	√	√
5319013	Licoricone	high	√	√	√	√	√
5317478	Gancaonin A	high	√	√	√	√	√
5317479	Gancaonin B	high	√	√	√	√	√
14604077	3-(3,4-dihydroxyphenyl)-5,7-dihydroxy-8-(3-methylbut-2-enyl)chromone	high	√	√	√	√	√

14604078	5,7-dihydroxy-3-(4-methoxyphenyl)-8-(3-methylbut-2-enyl)chromone	high	√	√	√	√	√
14604081	2-(3,4-dihydroxyphenyl)-5,7-dihydroxy-6-(3-methylbut-2-enyl)chromone	high	√	√	√	√	√
480787	Glycyrin	high	√	√	√	√	√
503731	Licocoumarone	high	√	√	√	√	√
5281789	Licoisoflavone	high	√	√	√	√	√
5318869	Jaranol	high	√	√	√	√	√
5481234	Licoisoflavone B	high	√	√	√	√	√
392443	licoisoflavanone	high	√	√	√	√	√
10336244	shinpterocarpin	high	√	√	√	√	√
11267805	(E)-3-[3,4-dihydroxy-5-(3-methylbut-2-enyl)phenyl]-1-(2,4-dihydroxyphenyl)prop-2-en-1-one	high	√	√	√	√	√
122851	licopyranocoumarin	high	√	√	√	√	√
195396	3,22-Dihydroxy-11-oxo-delta(12)-oleanene-27-alpha-methoxycarbonyl-29-oic acid	high	√		√	√	√
5317777	Glyzaglabrin	high	√	√	√	√	√
124052	Glabridin	high	√	√	√	√	√
124049	Glabranin	high	√	√	√	√	√
480774	Glabrene	high	√	√	√	√	√
336327	Medicarpin	high	√	√	√	√	√
5317652	Glabrone	high	√	√	√	√	√
11558452	1,3-dihydroxy-9-methoxy-6-benzofurano[3,2-c]chromenone	high	√	√	√	√	√
11602329	1,3-dihydroxy-8,9-dimethoxy-6-benzofurano[3,2-c]chromenone	high	√	√	√	√	√
5317300	Eurycarpin A	high	√	√	√	√	√
23724664	(-)-Medicocarpin	high	√	√	√	√	√
73205	Sigmoidin-B	high	√	√	√	√	√
928837	(2R)-7-hydroxy-2-(4-hydroxyphenyl)chroman-4-one	high	√	√	√	√	√
193679	(2S)-7-hydroxy-2-(4-hydroxyphenyl)-8-(3-methylbut-2-enyl)chroman-4-one	high	√	√	√	√	√
124050	Isoglycyrol	high	√	√	√	√	√
5318585	Isolicoflavonol	high	√	√	√	√	√
5281654	isorhamnetin	high	√	√	√	√	√
3764	HMO	high	√	√	√	√	√
480873	1-Methoxyphaseollidin	high	√	√	√	√	√
5316900	Quercetin der.	high	√	√	√	√	√
15228662	3'-Hydroxy-4'-O-Methylglabridin	high	√	√	√	√	√
5318998	licochalcone a	high	√	√	√	√	√
15228663	3'-Methoxyglabridin	high	√	√	√	√	√
9927807	2-[(3R)-8,8-dimethyl-3,4-dihydro-2H-pyrano[6,5-f]chromen-3-yl]-5-methoxyphenol	high	√	√	√	√	√
5318437	Inflacoumarin A	high	√	√	√	√	√
101666840	Kanzonol F	high	√	√	√	√	
25015742	7,2',4'-trihydroxy − 5-methoxy-3 − arylcoumarin	high	√	√	√	√	√
5317480	Lupiwighteone	high	√	√	√	√	√
268208	7-Acetoxy-2-methylisoflavone	high	√	√	√	√	√
177149	Vestitol	high	√	√	√	√	√
480780	Gancaonin G	high	√	√	√	√	√

5481949	Gancaonin H	high	√	√	√	√	
15840593	Licoagrocarpin	high	√	√	√	√	√
5317765	Glycyrrhiza flavonol A	high	√	√	√	√	√
636883	Licoagroisoflavone	high	√	√	√	√	√
13965473	Odoratin	high	√	√	√	√	√
44257530	Phaseol	high	√	√	√	√	√
14769500	Xambioona	high	√	√	√	√	√
354368	7-Methoxy-2-methyl isoflavone	high	√	√	√	√	√
5280378	formononetin	high	√	√	√	√	√
323	Coumarin	high	√		√	√	
444539	Cinnamic Acid	high	√		√	√	
11604108	(-)-Syringaresinol	High	√	√	√	√	√
614467	5-(2-Methyl-1,3-thiazol-4-yl)thiophene-2-carboxylic acid	high	√	√	√	√	√
2794766	2-[4-(Trifluoromethyl)phenyl]-1,3-thiazole-4-carboxylic Acid	high	√	√	√	√	√
135567045	2-(4-((Pyridin-4-Yl)methyl)piperazin-1-Yl)-3,4,5,6,7,8-Hexahydroquinazolin-4-One	high	√	√	√	√	√
196916	Julibrine I	high	√	√	√		
21594250	Macharinic acid lactone	high	√	√	√	√	√
5281646	Macluraxanthone	high	√	√	√	√	
69997336	Norarmepavine	high	√	√	√	√	√
54670067	Vitamin C	high	√		√	√	
9064	D-Catechin	high	√	√	√	√	√
12305894	acacic acid	high	√		√	√	
6712546	acacic acid lactone	high	√		√	√	
21119850	AP1	high	√			√	
444664	AP3	high	√	√	√		
73309	Echinocystic acid	high	√		√		
725031	2-[(4-Methylphenyl)thio]nicotinic acid	high	√	√	√	√	√
1453158	Keratinocyte Differentiation Inducer	high	√	√	√	√	√
131990	10-(4-Methylpiperazin-1-yl)pyrido(4,3-b)(1,4)benzothiazepine	high	√	√	√	√	√
33032	L-glutamic acid	high	√		√	√	
5960	l-aspartic acid	high	√		√	√	
5962	lysine	high	√		√	√	
205	DL-Threonine	high	√		√	√	
14729078	sanjoinenine	hgih	√		√	√	
102063083	zizyphusine	hgih	√	√	√	√	
12305768	Alphitolic Acid	high	√		√		
197017	N-Methylasimilobine	high	√	√	√	√	
23335	Caaverine	high	√	√	√	√	
15515703	Jujubogenin	high	√		√	√	
3085285	Juzirine	high	√	√	√	√	
161388	Virgaureagenin G	high	√		√	√	
21668841	Onjisaponin A	high	√	√	√	√	√
