# Supplementary material for: Potential Mechanism of Dingji Fumai Decoction Against Atrial Fibrillation Based on Network Pharmacology, Molecular Docking, and Experimental Verification Integration Strategy
Source: Front Cardiovasc Med. 2021 Nov 11;8:712398. doi: 10.3389/fcvm.2021.712398 (PMC8631917; doi:10.3389/fcvm.2021.712398)
Supplement: Supplementary file 2 [file Table_2.pdf]

Table S2. The Correspondence information between compounds and targets.

PubChem ID	Compound	Target
5280343	Quercetin	ABCB1
5280343	Quercetin	ABCC1
5280343	Quercetin	ABCG2
5280343	Quercetin	ACHE
5280343	Quercetin	ADORA1
5280343	Quercetin	ADORA2A
5280343	Quercetin	AHR
5280343	Quercetin	AKR1A1
5280343	Quercetin	AKR1B1
5280343	Quercetin	AKR1B10
5280343	Quercetin	AKR1C1
5280343	Quercetin	AKR1C2
5280343	Quercetin	AKR1C3
5280343	Quercetin	AKR1C4
5280343	Quercetin	AKT1
5280343	Quercetin	ALK
5280343	Quercetin	ALOX12
5280343	Quercetin	ALOX15
5280343	Quercetin	ALOX5
5280343	Quercetin	APEX1
5280343	Quercetin	APP
5280343	Quercetin	ARG1
5280343	Quercetin	AURKB
5280343	Quercetin	AVPR2
5280343	Quercetin	AXL
5280343	Quercetin	BACE1
5280343	Quercetin	CA1
5280343	Quercetin	CA12
5280343	Quercetin	CA13
5280343	Quercetin	CA14
5280343	Quercetin	CA2
5280343	Quercetin	CA3
5280343	Quercetin	CA4
5280343	Quercetin	CA5A
5280343	Quercetin	CA6
5280343	Quercetin	CA7
5280343	Quercetin	CA9
5280343	Quercetin	CAMK2B
5280343	Quercetin	CCNB1
5280343	Quercetin	CCNB2
5280343	Quercetin	CCNB3
5280343	Quercetin	CD38

5280343	Quercetin	CDK1
5280343	Quercetin	CDK2
5280343	Quercetin	CDK5
5280343	Quercetin	CDK5R1
5280343	Quercetin	CDK6
5280343	Quercetin	CSNK2A1
5280343	Quercetin	CXCR1
5280343	Quercetin	CYP19A1
5280343	Quercetin	CYP1B1
5280343	Quercetin	DAPK1
5280343	Quercetin	DRD4
5280343	Quercetin	EGFR
5280343	Quercetin	ESR2
5280343	Quercetin	ESRRA
5280343	Quercetin	F2
5280343	Quercetin	FLT3
5280343	Quercetin	GLO1
5280343	Quercetin	GPR35
5280343	Quercetin	GSK3B
5280343	Quercetin	HSD17B1
5280343	Quercetin	HSD17B2
5280343	Quercetin	IGF1R
5280343	Quercetin	INSR
5280343	Quercetin	KDM4E
5280343	Quercetin	KDR
5280343	Quercetin	MAOA
5280343	Quercetin	MAPT
5280343	Quercetin	MET
5280343	Quercetin	MMP12
5280343	Quercetin	MMP13
5280343	Quercetin	MMP2
5280343	Quercetin	MMP3
5280343	Quercetin	MMP9
5280343	Quercetin	MPG
5280343	Quercetin	MPO
5280343	Quercetin	MYLK
5280343	Quercetin	NEK2
5280343	Quercetin	NEK6
5280343	Quercetin	NOX4
5280343	Quercetin	NUAK1
5280343	Quercetin	PARP1
5280343	Quercetin	PIK3CG
5280343	Quercetin	PIK3R1
5280343	Quercetin	PIM1

5280343	Quercetin	PKN1
5280343	Quercetin	PLA2G1B
5280343	Quercetin	PLK1
5280343	Quercetin	PTK2
5280343	Quercetin	PTPRS
5280343	Quercetin	PYGL
5280343	Quercetin	SLC22A12
5280343	Quercetin	SRC
5280343	Quercetin	SYK
5280343	Quercetin	TERT
5280343	Quercetin	TNKS
5280343	Quercetin	TNKS2
5280343	Quercetin	TOP1
5280343	Quercetin	TOP2A
5280343	Quercetin	TTR
5280343	Quercetin	TYR
5280343	Quercetin	XDH
72	Protocatechuic Acid	AKR1C3
72	Protocatechuic Acid	ALB
72	Protocatechuic Acid	ALK
72	Protocatechuic Acid	BCL2L1
72	Protocatechuic Acid	CA1
72	Protocatechuic Acid	CA12
72	Protocatechuic Acid	CA13
72	Protocatechuic Acid	CA14
72	Protocatechuic Acid	CA2
72	Protocatechuic Acid	CA3
72	Protocatechuic Acid	CA4
72	Protocatechuic Acid	CA5A
72	Protocatechuic Acid	CA5B
72	Protocatechuic Acid	CA6
72	Protocatechuic Acid	CA7
72	Protocatechuic Acid	CA9
72	Protocatechuic Acid	COMT
72	Protocatechuic Acid	ESR2
72	Protocatechuic Acid	FUT7
72	Protocatechuic Acid	GPR35
72	Protocatechuic Acid	IGF1R
72	Protocatechuic Acid	LDHA
72	Protocatechuic Acid	LDHB
72	Protocatechuic Acid	SERPINE1
72	Protocatechuic Acid	SQLE
72	Protocatechuic Acid	TTR
985	Cetylic Acid	ABCB1

985	Cetylic Acid	ABCB1
985	Cetylic Acid	ABCC1
985	Cetylic Acid	AKR1B10
985	Cetylic Acid	ALOX12
985	Cetylic Acid	ALOX15
985	Cetylic Acid	AR
985	Cetylic Acid	CA1
985	Cetylic Acid	CA2
985	Cetylic Acid	CACNA2D1
985	Cetylic Acid	CDC25A
985	Cetylic Acid	CDC45
985	Cetylic Acid	CHRNA7
985	Cetylic Acid	CYP19A1
985	Cetylic Acid	CYP26A1
985	Cetylic Acid	CYP26B1
985	Cetylic Acid	EPHX2
985	Cetylic Acid	FAAH
985	Cetylic Acid	FABP1
985	Cetylic Acid	FABP2
985	Cetylic Acid	FABP3
985	Cetylic Acid	FABP4
985	Cetylic Acid	FABP5
985	Cetylic Acid	FFAR1
985	Cetylic Acid	FFAR4
985	Cetylic Acid	FNTA
985	Cetylic Acid	FNTB
985	Cetylic Acid	G6PD
985	Cetylic Acid	GABBR1
985	Cetylic Acid	GABRA2
985	Cetylic Acid	GABRB2
985	Cetylic Acid	GABRG2
985	Cetylic Acid	GLRA1
985	Cetylic Acid	GPBAR1
985	Cetylic Acid	GRM5
985	Cetylic Acid	GSTK1
985	Cetylic Acid	HAO1
985	Cetylic Acid	HMGCR
985	Cetylic Acid	HNF4A
985	Cetylic Acid	HSD11B1
985	Cetylic Acid	HSD11B2
985	Cetylic Acid	HSD17B3
985	Cetylic Acid	HTR2B
985	Cetylic Acid	KDM2A
985	Cetylic Acid	KDM5C

985	Cetylic Acid	LTA4H
985	Cetylic Acid	MAPK1
985	Cetylic Acid	NPC1L1
985	Cetylic Acid	NR0B2
985	Cetylic Acid	NR1H4
985	Cetylic Acid	PHF8
985	Cetylic Acid	PLA2G4A
985	Cetylic Acid	PLG
985	Cetylic Acid	POLB
985	Cetylic Acid	PPARA
985	Cetylic Acid	PPARD
985	Cetylic Acid	PPARG
985	Cetylic Acid	PTGER2
985	Cetylic Acid	PTGER4
985	Cetylic Acid	PTGES
985	Cetylic Acid	PTGFR
985	Cetylic Acid	PTPN1
985	Cetylic Acid	PTPRC
985	Cetylic Acid	RARA
985	Cetylic Acid	RARB
985	Cetylic Acid	RARG
985	Cetylic Acid	RORA
985	Cetylic Acid	RORB
985	Cetylic Acid	RXRA
985	Cetylic Acid	RXRB
985	Cetylic Acid	RXRG
985	Cetylic Acid	SAE1
985	Cetylic Acid	SCD
985	Cetylic Acid	SERPINA6
985	Cetylic Acid	SHBG
985	Cetylic Acid	SLC16A1
985	Cetylic Acid	SLC22A6
985	Cetylic Acid	TBXA2R
985	Cetylic Acid	TERT
985	Cetylic Acid	UBA2
985	Cetylic Acid	UGT2B7
985	Cetylic Acid	VDR
689043	Caffeic Acid	AKR1B1
689043	Caffeic Acid	AKR1B10
689043	Caffeic Acid	AKR1C2
689043	Caffeic Acid	AKR1C3
689043	Caffeic Acid	AKR1C4
689043	Caffeic Acid	ALOX5
689043	Caffeic Acid	APP

689043	Caffeic Acid	CA1
689043	Caffeic Acid	CA12
689043	Caffeic Acid	CA13
689043	Caffeic Acid	CA14
689043	Caffeic Acid	CA2
689043	Caffeic Acid	CA3
689043	Caffeic Acid	CA4
689043	Caffeic Acid	CA5A
689043	Caffeic Acid	CA5B
689043	Caffeic Acid	CA6
689043	Caffeic Acid	CA7
689043	Caffeic Acid	CA9
689043	Caffeic Acid	CYP1A2
689043	Caffeic Acid	CYP2C19
689043	Caffeic Acid	CYP2C9
689043	Caffeic Acid	CYP3A4
689043	Caffeic Acid	EGFR
689043	Caffeic Acid	ELANE
689043	Caffeic Acid	ERBB2
689043	Caffeic Acid	ESR1
689043	Caffeic Acid	ESR2
689043	Caffeic Acid	F3
689043	Caffeic Acid	FYN
689043	Caffeic Acid	HCAR2
689043	Caffeic Acid	HSD11B1
689043	Caffeic Acid	LCK
689043	Caffeic Acid	MAOB
689043	Caffeic Acid	MAPK1
689043	Caffeic Acid	MIF
689043	Caffeic Acid	MMP1
689043	Caffeic Acid	MMP2
689043	Caffeic Acid	MMP9
689043	Caffeic Acid	NFE2L2
689043	Caffeic Acid	NQO2
689043	Caffeic Acid	PIK3CA
689043	Caffeic Acid	PIK3CB
689043	Caffeic Acid	PTGS1
689043	Caffeic Acid	PTPN1
689043	Caffeic Acid	SLC6A2
689043	Caffeic Acid	STAT3
689043	Caffeic Acid	SYK
689043	Caffeic Acid	TLR4
689043	Caffeic Acid	TTR
5321250	Senkyunolide G	ADAMTS4

5321250	Senkyunolide G	ADAMTS5
5321250	Senkyunolide G	ADORA2A
5321250	Senkyunolide G	ADORA2B
5321250	Senkyunolide G	ADORA3
5321250	Senkyunolide G	ADRA2A
5321250	Senkyunolide G	ADRA2B
5321250	Senkyunolide G	ADRA2C
5321250	Senkyunolide G	CCNB1
5321250	Senkyunolide G	CCNB3
5321250	Senkyunolide G	CCNE1
5321250	Senkyunolide G	CCNE2
5321250	Senkyunolide G	CDK1
5321250	Senkyunolide G	CDK2
5321250	Senkyunolide G	CDK5
5321250	Senkyunolide G	CDK5R1
5321250	Senkyunolide G	CHRM2
5321250	Senkyunolide G	CHRM4
5321250	Senkyunolide G	DCTPP1
5321250	Senkyunolide G	DYRK1A
5321250	Senkyunolide G	EPHX1
5321250	Senkyunolide G	FADS1
5321250	Senkyunolide G	FGFR1
5321250	Senkyunolide G	GRM4
5321250	Senkyunolide G	HCRTR1
5321250	Senkyunolide G	HCRTR2
5321250	Senkyunolide G	HMOX1
5321250	Senkyunolide G	HRH3
5321250	Senkyunolide G	HRH4
5321250	Senkyunolide G	IGF1R
5321250	Senkyunolide G	IMPDH2
5321250	Senkyunolide G	INSR
5321250	Senkyunolide G	JUN
5321250	Senkyunolide G	KCNA5
5321250	Senkyunolide G	KDR
5321250	Senkyunolide G	LRRK2
5321250	Senkyunolide G	MAPK1
5321250	Senkyunolide G	MAPK10
5321250	Senkyunolide G	MAPK14
5321250	Senkyunolide G	MAPK8
5321250	Senkyunolide G	MDM2
5321250	Senkyunolide G	MTNR1A
5321250	Senkyunolide G	MTNR1B
5321250	Senkyunolide G	NT5E
5321250	Senkyunolide G	NTRK1

5321250	Senkyunolide G	P2RX7
5321250	Senkyunolide G	PARP1
5321250	Senkyunolide G	PLA2G1B
5321250	Senkyunolide G	PLA2G2A
5321250	Senkyunolide G	PTK2B
5321250	Senkyunolide G	RIPK2
5321250	Senkyunolide G	SIGMAR1
5321250	Senkyunolide G	SLC6A4
5321250	Senkyunolide G	STK26
5321250	Senkyunolide G	STK3
5321250	Senkyunolide G	TNNT2
5321250	Senkyunolide G	TRPV3
5321250	Senkyunolide G	TTL
91726743	Senkyunone	ABCB1
91726743	Senkyunone	ABCC1
91726743	Senkyunone	ACACA
91726743	Senkyunone	ACACB
91726743	Senkyunone	ALOX5
91726743	Senkyunone	C3AR1
91726743	Senkyunone	CALCRL
91726743	Senkyunone	CALCRL
91726743	Senkyunone	CCKBR
91726743	Senkyunone	CFTR
91726743	Senkyunone	CRHR1
91726743	Senkyunone	CTSK
91726743	Senkyunone	CTSL
91726743	Senkyunone	CTSS
91726743	Senkyunone	CYP19A1
91726743	Senkyunone	CYP24A1
91726743	Senkyunone	CYP27A1
91726743	Senkyunone	DGAT2
91726743	Senkyunone	DKK1
91726743	Senkyunone	DRD2
91726743	Senkyunone	DRD3
91726743	Senkyunone	DRD4
91726743	Senkyunone	EPHX2
91726743	Senkyunone	F10
91726743	Senkyunone	F13A1
91726743	Senkyunone	F2R
91726743	Senkyunone	FASN
91726743	Senkyunone	GLI2
91726743	Senkyunone	GPR183
91726743	Senkyunone	GPR88
91726743	Senkyunone	GRM2

91726743	Senkyunone	GRM5
91726743	Senkyunone	HCRTR1
91726743	Senkyunone	HCRTR2
91726743	Senkyunone	HPGDS
91726743	Senkyunone	HRH1
91726743	Senkyunone	HSD17B2
91726743	Senkyunone	HTR1A
91726743	Senkyunone	HTR2A
91726743	Senkyunone	HTR2C
91726743	Senkyunone	HTR7
91726743	Senkyunone	ICAM1
91726743	Senkyunone	IMPDH2
91726743	Senkyunone	ITK
91726743	Senkyunone	KCNA5
91726743	Senkyunone	KCNH2
91726743	Senkyunone	KDR
91726743	Senkyunone	KIF11
91726743	Senkyunone	KNG1
91726743	Senkyunone	LSS
91726743	Senkyunone	LYPLA2
91726743	Senkyunone	MAOB
91726743	Senkyunone	MAPK10
91726743	Senkyunone	MAPK11
91726743	Senkyunone	MAPK14
91726743	Senkyunone	MDM2
91726743	Senkyunone	MGAT2
91726743	Senkyunone	MTNR1A
91726743	Senkyunone	MTNR1B
91726743	Senkyunone	NPY5R
91726743	Senkyunone	NR3C1
91726743	Senkyunone	OPRD1
91726743	Senkyunone	PARP1
91726743	Senkyunone	PDE10A
91726743	Senkyunone	PDE4B
91726743	Senkyunone	PDE7A
91726743	Senkyunone	PFKFB3
91726743	Senkyunone	PLK1
91726743	Senkyunone	POLH
91726743	Senkyunone	POLK
91726743	Senkyunone	POLL
91726743	Senkyunone	POLM
91726743	Senkyunone	PTGER4
91726743	Senkyunone	PTGS2
91726743	Senkyunone	RAMP1

91726743	Senkyunone	RORB
91726743	Senkyunone	SCARB1
91726743	Senkyunone	SCD
91726743	Senkyunone	SCN5A
91726743	Senkyunone	SCN9A
91726743	Senkyunone	SELE
91726743	Senkyunone	SLC5A1
91726743	Senkyunone	SMO
91726743	Senkyunone	SOAT1
91726743	Senkyunone	SRC
91726743	Senkyunone	SYK
91726743	Senkyunone	TEK
91726743	Senkyunone	TGM1
91726743	Senkyunone	TGM2
91726743	Senkyunone	THRA
91726743	Senkyunone	THRB
91726743	Senkyunone	TRPV1
91726743	Senkyunone	UTS2R
92231	Spathulenol	HSD11B1
92231	Spathulenol	ICMT
92231	Spathulenol	IDO1
92231	Spathulenol	NR1H3
92231	Spathulenol	PGR
92231	Spathulenol	PTGS1
92231	Spathulenol	UGT2B7
1174	Uracil	DPYD
1174	Uracil	PARP1
1174	Uracil	TYMS
1183	Vanillin	ALPG
1183	Vanillin	CA1
1183	Vanillin	CA12
1183	Vanillin	CA14
1183	Vanillin	CA2
1183	Vanillin	CA3
1183	Vanillin	CA4
1183	Vanillin	CA5A
1183	Vanillin	CA6
1183	Vanillin	CA7
1183	Vanillin	CA9
1183	Vanillin	CDC25B
1183	Vanillin	EP300
1183	Vanillin	ERN1
1183	Vanillin	NAT1
1183	Vanillin	PLAA

1183	Vanillin	PTPRC
1183	Vanillin	TTR
122736	Wallichilide	ATP12A
122736	Wallichilide	ATP1A1
122736	Wallichilide	BCL2L1
122736	Wallichilide	CCR5
122736	Wallichilide	F2RL1
122736	Wallichilide	GIPR
122736	Wallichilide	GLP1R
122736	Wallichilide	GLRA1
122736	Wallichilide	GLRA2
122736	Wallichilide	IARS
122736	Wallichilide	LTB4R
122736	Wallichilide	PDE5A
122736	Wallichilide	PRKCB
122736	Wallichilide	PRKCE
122736	Wallichilide	PRKCH
122736	Wallichilide	PRKCQ
122736	Wallichilide	PTGER1
122736	Wallichilide	PTGER3
122736	Wallichilide	PTGFR
122736	Wallichilide	PTGS2
122736	Wallichilide	PTPA
122736	Wallichilide	RORC
122736	Wallichilide	STAT3
10288	Chrysophanic Acid	ABL1
10288	Chrysophanic Acid	ACHE
10288	Chrysophanic Acid	CDC7
10288	Chrysophanic Acid	CDK1
10288	Chrysophanic Acid	CDK2
10288	Chrysophanic Acid	CHEK1
10288	Chrysophanic Acid	CXCR1
10288	Chrysophanic Acid	CXCR2
10288	Chrysophanic Acid	DAO
10288	Chrysophanic Acid	ESR1
10288	Chrysophanic Acid	ESR2
10288	Chrysophanic Acid	FADS1
10288	Chrysophanic Acid	GPR84
10288	Chrysophanic Acid	GSTA1
10288	Chrysophanic Acid	HDAC1
10288	Chrysophanic Acid	HSP90AA1
10288	Chrysophanic Acid	HSP90AB1
10288	Chrysophanic Acid	KCNMA1
10288	Chrysophanic Acid	MB

10288	Chrysophanic Acid	MCL1
10288	Chrysophanic Acid	NOX4
10288	Chrysophanic Acid	SRC
10288	Chrysophanic Acid	UPP1
10208	Chrysophanol	ABL1
10208	Chrysophanol	ACE
10208	Chrysophanol	ADAM17
10208	Chrysophanol	ADORA2B
10208	Chrysophanol	ADRA1A
10208	Chrysophanol	ADRA2A
10208	Chrysophanol	ADRA2B
10208	Chrysophanol	ALPG
10208	Chrysophanol	ALPL
10208	Chrysophanol	ASF1A
10208	Chrysophanol	BACE1
10208	Chrysophanol	BCL2
10208	Chrysophanol	CA14
10208	Chrysophanol	CA7
10208	Chrysophanol	CHEK1
10208	Chrysophanol	CISD1
10208	Chrysophanol	CSNK1A1
10208	Chrysophanol	CSNK1D
10208	Chrysophanol	CSNK2A1
10208	Chrysophanol	CTSV
10208	Chrysophanol	CYP19A1
10208	Chrysophanol	DAO
10208	Chrysophanol	DUSP3
10208	Chrysophanol	EGFR
10208	Chrysophanol	EIF2AK2
10208	Chrysophanol	ELANE
10208	Chrysophanol	ERN1
10208	Chrysophanol	ESR1
10208	Chrysophanol	ESR2
10208	Chrysophanol	FGFR1
10208	Chrysophanol	FLT1
10208	Chrysophanol	FNTA
10208	Chrysophanol	FNTB
10208	Chrysophanol	FTO
10208	Chrysophanol	GRM5
10208	Chrysophanol	GUSB
10208	Chrysophanol	HCK
10208	Chrysophanol	HDAC1
10208	Chrysophanol	HDAC4
10208	Chrysophanol	HDAC5

10208	Chrysophanol	HDAC6
10208	Chrysophanol	HDAC7
10208	Chrysophanol	HDAC8
10208	Chrysophanol	HMGCR
10208	Chrysophanol	HSD11B1
10208	Chrysophanol	HSP90AA1
10208	Chrysophanol	HSP90AB1
10208	Chrysophanol	HTR2B
10208	Chrysophanol	KDR
10208	Chrysophanol	LCK
10208	Chrysophanol	LDHA
10208	Chrysophanol	LDHB
10208	Chrysophanol	MAOB
10208	Chrysophanol	MAP2K1
10208	Chrysophanol	MCL1
10208	Chrysophanol	MMP1
10208	Chrysophanol	MMP12
10208	Chrysophanol	MMP13
10208	Chrysophanol	MMP14
10208	Chrysophanol	MMP16
10208	Chrysophanol	MMP2
10208	Chrysophanol	MMP3
10208	Chrysophanol	MMP7
10208	Chrysophanol	MMP8
10208	Chrysophanol	MMP9
10208	Chrysophanol	MTOR
10208	Chrysophanol	NAT1
10208	Chrysophanol	NISCH
10208	Chrysophanol	NOS2
10208	Chrysophanol	NOX4
10208	Chrysophanol	PI4KB
10208	Chrysophanol	PIK3CA
10208	Chrysophanol	PIK3CB
10208	Chrysophanol	PIK3CD
10208	Chrysophanol	PIK3CG
10208	Chrysophanol	PIM1
10208	Chrysophanol	PLA2G7
10208	Chrysophanol	PLAA
10208	Chrysophanol	PLEC
10208	Chrysophanol	PRKDC
10208	Chrysophanol	PTP4A3
10208	Chrysophanol	RET
10208	Chrysophanol	SRC

14900	Dilinoyl Palmitoyl Glyceride,Glycerol1-Monopalmitate	ABCB1
14900	Dilinoyl Palmitoyl Glyceride,Glycerol1-Monopalmitate	ACACB
14900	Dilinoyl Palmitoyl Glyceride,Glycerol1-Monopalmitate	ACP1
14900	Dilinoyl Palmitoyl Glyceride,Glycerol1-Monopalmitate	AKT1
14900	Dilinoyl Palmitoyl Glyceride,Glycerol1-Monopalmitate	APH1A
14900	Dilinoyl Palmitoyl Glyceride,Glycerol1-Monopalmitate	APH1B
14900	Dilinoyl Palmitoyl Glyceride,Glycerol1-Monopalmitate	FAAH
14900	Dilinoyl Palmitoyl Glyceride,Glycerol1-Monopalmitate	FASN
14900	Dilinoyl Palmitoyl Glyceride,Glycerol1-Monopalmitate	GPR119
14900	Dilinoyl Palmitoyl Glyceride,Glycerol1-Monopalmitate	HMGCR
14900	Dilinoyl Palmitoyl Glyceride,Glycerol1-Monopalmitate	HSD11B1
14900	Dilinoyl Palmitoyl Glyceride,Glycerol1-Monopalmitate	ICAM1
14900	Dilinoyl Palmitoyl Glyceride,Glycerol1-Monopalmitate	IKBKE
14900	Dilinoyl Palmitoyl Glyceride,Glycerol1-Monopalmitate	IL2

14900	Dilinoyl Palmitoyl Glyceride,Glycerol1-Monopalmitate	ITGAL
14900	Dilinoyl Palmitoyl Glyceride,Glycerol1-Monopalmitate	ITGB2
14900	Dilinoyl Palmitoyl Glyceride,Glycerol1-Monopalmitate	LPAR5
14900	Dilinoyl Palmitoyl Glyceride,Glycerol1-Monopalmitate	LPAR6
14900	Dilinoyl Palmitoyl Glyceride,Glycerol1-Monopalmitate	NCSTN
14900	Dilinoyl Palmitoyl Glyceride,Glycerol1-Monopalmitate	OPRK1
14900	Dilinoyl Palmitoyl Glyceride,Glycerol1-Monopalmitate	PAM
14900	Dilinoyl Palmitoyl Glyceride,Glycerol1-Monopalmitate	PPM1B
14900	Dilinoyl Palmitoyl Glyceride,Glycerol1-Monopalmitate	PPP1CA
14900	Dilinoyl Palmitoyl Glyceride,Glycerol1-Monopalmitate	PPP1CC
14900	Dilinoyl Palmitoyl Glyceride,Glycerol1-Monopalmitate	PPP5C
14900	Dilinoyl Palmitoyl Glyceride,Glycerol1-Monopalmitate	PRKCA
14900	Dilinoyl Palmitoyl Glyceride,Glycerol1-Monopalmitate	PRKCD
14900	Dilinoyl Palmitoyl Glyceride,Glycerol1-Monopalmitate	PRKCE

14900	Dilinoyl Palmitoyl Glyceride,Glycerol1-Monopalmitate	PRKCG
14900	Dilinoyl Palmitoyl Glyceride,Glycerol1-Monopalmitate	PRKCH
14900	Dilinoyl Palmitoyl Glyceride,Glycerol1-Monopalmitate	PRKCQ
14900	Dilinoyl Palmitoyl Glyceride,Glycerol1-Monopalmitate	PSEN1
14900	Dilinoyl Palmitoyl Glyceride,Glycerol1-Monopalmitate	PSEN2
14900	Dilinoyl Palmitoyl Glyceride,Glycerol1-Monopalmitate	PSENEN
14900	Dilinoyl Palmitoyl Glyceride,Glycerol1-Monopalmitate	PTPN1
14900	Dilinoyl Palmitoyl Glyceride,Glycerol1-Monopalmitate	S1PR1
14900	Dilinoyl Palmitoyl Glyceride,Glycerol1-Monopalmitate	S1PR3
14900	Dilinoyl Palmitoyl Glyceride,Glycerol1-Monopalmitate	S1PR4
14900	Dilinoyl Palmitoyl Glyceride,Glycerol1-Monopalmitate	S1PR5
14900	Dilinoyl Palmitoyl Glyceride,Glycerol1-Monopalmitate	SIRT2
14900	Dilinoyl Palmitoyl Glyceride,Glycerol1-Monopalmitate	TBK1
14900	Dilinoyl Palmitoyl Glyceride,Glycerol1-Monopalmitate	TRPV4

14900	Dilinoyl Palmitoyl Glyceride,Glycerol1-Monopalmitate	UGT2B7
14900	Dilinoyl Palmitoyl Glyceride,Glycerol1-Monopalmitate	VDR
38762	Ethylpentadecanoate	ACHE
38762	Ethylpentadecanoate	AKT1
38762	Ethylpentadecanoate	CA1
38762	Ethylpentadecanoate	CA12
38762	Ethylpentadecanoate	CA2
38762	Ethylpentadecanoate	CA9
38762	Ethylpentadecanoate	CDC25A
38762	Ethylpentadecanoate	CYP19A1
38762	Ethylpentadecanoate	CYP24A1
38762	Ethylpentadecanoate	CYP27B1
38762	Ethylpentadecanoate	F2R
38762	Ethylpentadecanoate	FAAH
38762	Ethylpentadecanoate	FABP2
38762	Ethylpentadecanoate	FABP3
38762	Ethylpentadecanoate	FABP4
38762	Ethylpentadecanoate	FABP5
38762	Ethylpentadecanoate	FFAR1
38762	Ethylpentadecanoate	FNTA
38762	Ethylpentadecanoate	GNRHR
38762	Ethylpentadecanoate	GRIN2B
38762	Ethylpentadecanoate	HMGCR
38762	Ethylpentadecanoate	HRH3
38762	Ethylpentadecanoate	HRH4
38762	Ethylpentadecanoate	HSD11B1
38762	Ethylpentadecanoate	HSD17B3
38762	Ethylpentadecanoate	KCNH2
38762	Ethylpentadecanoate	LIMK2
38762	Ethylpentadecanoate	MAPK14
38762	Ethylpentadecanoate	MCHR1
38762	Ethylpentadecanoate	PAM
38762	Ethylpentadecanoate	PGGT1B
38762	Ethylpentadecanoate	PPM1B
38762	Ethylpentadecanoate	PPP1CC
38762	Ethylpentadecanoate	PRKCQ
38762	Ethylpentadecanoate	PTPN1
38762	Ethylpentadecanoate	ROCK1
38762	Ethylpentadecanoate	SIRT2
38762	Ethylpentadecanoate	SSTR2

38762	Ethylpentadecanoate	SSTR4
38762	Ethylpentadecanoate	TRPV1
38762	Ethylpentadecanoate	UGT2B7
38762	Ethylpentadecanoate	VDR
8181	Methyl Hexadecanate,Methyl Palmitate	AKR1B10
8181	Methyl Hexadecanate,Methyl Palmitate	ALOX5
8181	Methyl Hexadecanate,Methyl Palmitate	CA1
8181	Methyl Hexadecanate,Methyl Palmitate	CA2
8181	Methyl Hexadecanate,Methyl Palmitate	CNR1
8181	Methyl Hexadecanate,Methyl Palmitate	CNR2
8181	Methyl Hexadecanate,Methyl Palmitate	CPT1A
8181	Methyl Hexadecanate,Methyl Palmitate	CYP19A1
8181	Methyl Hexadecanate,Methyl Palmitate	DCK
8181	Methyl Hexadecanate,Methyl Palmitate	F2R
8181	Methyl Hexadecanate,Methyl Palmitate	FABP2
8181	Methyl Hexadecanate,Methyl Palmitate	FABP3
8181	Methyl Hexadecanate,Methyl Palmitate	FABP4
8181	Methyl Hexadecanate,Methyl Palmitate	FABP5
8181	Methyl Hexadecanate,Methyl Palmitate	FFAR1
8181	Methyl Hexadecanate,Methyl Palmitate	G6PD
8181	Methyl Hexadecanate,Methyl Palmitate	GABBR1
8181	Methyl Hexadecanate,Methyl Palmitate	GPBAR1
8181	Methyl Hexadecanate,Methyl Palmitate	GRIN2B
8181	Methyl Hexadecanate,Methyl Palmitate	HMGCR

8181	Methyl Hexadecanate,Methyl Palmitate	HSD11B1
8181	Methyl Hexadecanate,Methyl Palmitate	HSD17B3
8181	Methyl Hexadecanate,Methyl Palmitate	KDM2A
8181	Methyl Hexadecanate,Methyl Palmitate	KDM5C
8181	Methyl Hexadecanate,Methyl Palmitate	MPEG1
8181	Methyl Hexadecanate,Methyl Palmitate	NR1H4
8181	Methyl Hexadecanate,Methyl Palmitate	PHF8
8181	Methyl Hexadecanate,Methyl Palmitate	PLA2G4A
8181	Methyl Hexadecanate,Methyl Palmitate	POLB
8181	Methyl Hexadecanate,Methyl Palmitate	PPARA
8181	Methyl Hexadecanate,Methyl Palmitate	PPARD
8181	Methyl Hexadecanate,Methyl Palmitate	PTGFR
8181	Methyl Hexadecanate,Methyl Palmitate	PTPN1
8181	Methyl Hexadecanate,Methyl Palmitate	SERPINA6
8181	Methyl Hexadecanate,Methyl Palmitate	SHBG
8181	Methyl Hexadecanate,Methyl Palmitate	SLC1A1
8181	Methyl Hexadecanate,Methyl Palmitate	SLC22A6
8181	Methyl Hexadecanate,Methyl Palmitate	TRPV1
8181	Methyl Hexadecanate,Methyl Palmitate	UGT2B7
8181	Methyl Hexadecanate,Methyl Palmitate	VDR
23518	Methyl Pentadecanoate	ABL1
23518	Methyl Pentadecanoate	ACHE
23518	Methyl Pentadecanoate	ADK
23518	Methyl Pentadecanoate	ALOX5AP

23518	Methyl Pentadecanoate	APH1B
23518	Methyl Pentadecanoate	APOB
23518	Methyl Pentadecanoate	AR
23518	Methyl Pentadecanoate	AVPR1A
23518	Methyl Pentadecanoate	AVPR2
23518	Methyl Pentadecanoate	BCL2
23518	Methyl Pentadecanoate	CA1
23518	Methyl Pentadecanoate	CA14
23518	Methyl Pentadecanoate	CA2
23518	Methyl Pentadecanoate	CCNE1
23518	Methyl Pentadecanoate	CDC25A
23518	Methyl Pentadecanoate	CDK2
23518	Methyl Pentadecanoate	CDK4
23518	Methyl Pentadecanoate	CLK1
23518	Methyl Pentadecanoate	CLK2
23518	Methyl Pentadecanoate	CLK4
23518	Methyl Pentadecanoate	CNR1
23518	Methyl Pentadecanoate	CNR2
23518	Methyl Pentadecanoate	CPT1A
23518	Methyl Pentadecanoate	CTSB
23518	Methyl Pentadecanoate	CTSK
23518	Methyl Pentadecanoate	CTSS
23518	Methyl Pentadecanoate	CYP19A1
23518	Methyl Pentadecanoate	DRD2
23518	Methyl Pentadecanoate	DYRK1A
23518	Methyl Pentadecanoate	DYRK1B
23518	Methyl Pentadecanoate	F2R
23518	Methyl Pentadecanoate	FABP2
23518	Methyl Pentadecanoate	FABP3
23518	Methyl Pentadecanoate	FABP4
23518	Methyl Pentadecanoate	FABP5
23518	Methyl Pentadecanoate	FFAR1
23518	Methyl Pentadecanoate	FNTA
23518	Methyl Pentadecanoate	FNTA
23518	Methyl Pentadecanoate	FNTB
23518	Methyl Pentadecanoate	GABBR1
23518	Methyl Pentadecanoate	GCGR
23518	Methyl Pentadecanoate	GRIN2B
23518	Methyl Pentadecanoate	GRK2
23518	Methyl Pentadecanoate	HMGCR
23518	Methyl Pentadecanoate	HRH3
23518	Methyl Pentadecanoate	HRH4
23518	Methyl Pentadecanoate	HSD11B1
23518	Methyl Pentadecanoate	HSD17B3

23518	Methyl Pentadecanoate	KDM2A
23518	Methyl Pentadecanoate	KDM5C
23518	Methyl Pentadecanoate	LIPE
23518	Methyl Pentadecanoate	LRRK2
23518	Methyl Pentadecanoate	MAPK14
23518	Methyl Pentadecanoate	MCL1
23518	Methyl Pentadecanoate	MDM2
23518	Methyl Pentadecanoate	MGLL
23518	Methyl Pentadecanoate	MTTP
23518	Methyl Pentadecanoate	NCSTN
23518	Methyl Pentadecanoate	NPY5R
23518	Methyl Pentadecanoate	NR1H4
23518	Methyl Pentadecanoate	PDE10A
23518	Methyl Pentadecanoate	PGGT1B
23518	Methyl Pentadecanoate	PHF8
23518	Methyl Pentadecanoate	POLB
23518	Methyl Pentadecanoate	PPARA
23518	Methyl Pentadecanoate	PPARD
23518	Methyl Pentadecanoate	PSEN1
23518	Methyl Pentadecanoate	PSEN2
23518	Methyl Pentadecanoate	PSENEN
23518	Methyl Pentadecanoate	PTPN1
23518	Methyl Pentadecanoate	RARA
23518	Methyl Pentadecanoate	RARB
23518	Methyl Pentadecanoate	RARG
23518	Methyl Pentadecanoate	ROCK1
23518	Methyl Pentadecanoate	SERPINA6
23518	Methyl Pentadecanoate	SLC1A1
23518	Methyl Pentadecanoate	SLC22A6
23518	Methyl Pentadecanoate	STS
23518	Methyl Pentadecanoate	TOP2A
23518	Methyl Pentadecanoate	TRPV1
23518	Methyl Pentadecanoate	TSPO
23518	Methyl Pentadecanoate	UGT2B7
23518	Methyl Pentadecanoate	VDR
161748	Myricanone	ABL1
161748	Myricanone	ADAM17
161748	Myricanone	ADAM9
161748	Myricanone	ADORA2A
161748	Myricanone	ADORA2B
161748	Myricanone	ADRA2A
161748	Myricanone	ADRA2B
161748	Myricanone	ADRA2C
161748	Myricanone	ALOX5

161748	Myricanone	ANPEP
161748	Myricanone	AURKA
161748	Myricanone	BLK
161748	Myricanone	BMX
161748	Myricanone	BRAF
161748	Myricanone	BTK
161748	Myricanone	CA9
161748	Myricanone	CCNA1
161748	Myricanone	CCNA2
161748	Myricanone	CDK1
161748	Myricanone	CDK2
161748	Myricanone	CDK5
161748	Myricanone	CDK5R1
161748	Myricanone	CMA1
161748	Myricanone	CNR1
161748	Myricanone	CNR2
161748	Myricanone	COQ8B
161748	Myricanone	CSK
161748	Myricanone	CTSV
161748	Myricanone	CXCR2
161748	Myricanone	CYP11B1
161748	Myricanone	CYP11B2
161748	Myricanone	DYRK1A
161748	Myricanone	EGFR
161748	Myricanone	EGLN1
161748	Myricanone	ELANE
161748	Myricanone	EPHA1
161748	Myricanone	EPHA2
161748	Myricanone	EPHA3
161748	Myricanone	EPHA4
161748	Myricanone	EPHA5
161748	Myricanone	EPHA6
161748	Myricanone	EPHB2
161748	Myricanone	EPHB3
161748	Myricanone	ERBB2
161748	Myricanone	FAAH
161748	Myricanone	FGR
161748	Myricanone	FLT1
161748	Myricanone	FLT3
161748	Myricanone	FLT4
161748	Myricanone	FYN
161748	Myricanone	GCGR
161748	Myricanone	GSK3B
161748	Myricanone	HDAC10

161748	Myricanone	HDAC11
161748	Myricanone	HDAC3
161748	Myricanone	HDAC3
161748	Myricanone	HDAC7
161748	Myricanone	HSP90AA1
161748	Myricanone	HSP90AB1
161748	Myricanone	JAK2
161748	Myricanone	LCK
161748	Myricanone	LYN
161748	Myricanone	MAP2K2
161748	Myricanone	MAPK14
161748	Myricanone	MAPKAPK2
161748	Myricanone	MMP1
161748	Myricanone	MMP13
161748	Myricanone	MMP2
161748	Myricanone	MMP3
161748	Myricanone	MMP7
161748	Myricanone	MMP9
161748	Myricanone	MYLK
161748	Myricanone	NCOR1
161748	Myricanone	NOX4
161748	Myricanone	NPY5R
161748	Myricanone	P2RX7
161748	Myricanone	PARP1
161748	Myricanone	PDE10A
161748	Myricanone	PDGFRA
161748	Myricanone	PIK3CD
161748	Myricanone	PIK3CG
161748	Myricanone	PIM1
161748	Myricanone	PIM2
161748	Myricanone	PIM3
161748	Myricanone	PLA2G7
161748	Myricanone	PPIA
161748	Myricanone	PRKCG
161748	Myricanone	PTK2B
161748	Myricanone	PTK6
161748	Myricanone	RAF1
161748	Myricanone	RET
161748	Myricanone	ROCK1
161748	Myricanone	ROCK2
161748	Myricanone	RPS6KB1
161748	Myricanone	STAT3
161748	Myricanone	STAT6
161748	Myricanone	SYK

161748	Myricanone	TAAR1
161748	Myricanone	TACR3
161748	Myricanone	TRPM8
161748	Myricanone	TXK
161748	Myricanone	TYMS
161748	Myricanone	TYRO3
161748	Myricanone	YES1
445354	Retinol	ADORA3
445354	Retinol	ADRA2B
445354	Retinol	CCNB2
445354	Retinol	CNR1
445354	Retinol	EPHX2
445354	Retinol	ESR1
445354	Retinol	ESR2
445354	Retinol	GRM2
445354	Retinol	HTR2B
445354	Retinol	MAPK1
445354	Retinol	MAPK14
445354	Retinol	RARA
445354	Retinol	RARB
445354	Retinol	RARG
445354	Retinol	RBP4
445354	Retinol	RORA
445354	Retinol	RORB
445354	Retinol	RORC
445354	Retinol	RXRA
445354	Retinol	RXRB
445354	Retinol	RXRG
445858	3-O-trans ferulylquinic acid	ABCB1
445858	3-O-trans ferulylquinic acid	ABCB1
445858	3-O-trans ferulylquinic acid	ADORA1
445858	3-O-trans ferulylquinic acid	ADORA2A
445858	3-O-trans ferulylquinic acid	ADORA2B
445858	3-O-trans ferulylquinic acid	AHR
445858	3-O-trans ferulylquinic acid	AKR1B1
445858	3-O-trans ferulylquinic acid	AKR1B10
445858	3-O-trans ferulylquinic acid	ALOX15
445858	3-O-trans ferulylquinic acid	ALOX5
445858	3-O-trans ferulylquinic acid	AMPD3
445858	3-O-trans ferulylquinic acid	APP
445858	3-O-trans ferulylquinic acid	BACE1
445858	3-O-trans ferulylquinic acid	CA1
445858	3-O-trans ferulylquinic acid	CA12
445858	3-O-trans ferulylquinic acid	CA13

445858	3-O-trans ferulylquinic acid	CA14
445858	3-O-trans ferulylquinic acid	CA2
445858	3-O-trans ferulylquinic acid	CA3
445858	3-O-trans ferulylquinic acid	CA4
445858	3-O-trans ferulylquinic acid	CA5A
445858	3-O-trans ferulylquinic acid	CA5B
445858	3-O-trans ferulylquinic acid	CA6
445858	3-O-trans ferulylquinic acid	CA7
445858	3-O-trans ferulylquinic acid	CA9
445858	3-O-trans ferulylquinic acid	CCND1
445858	3-O-trans ferulylquinic acid	CDK4
445858	3-O-trans ferulylquinic acid	CPA1
445858	3-O-trans ferulylquinic acid	CYP1A1
445858	3-O-trans ferulylquinic acid	CYP1A2
445858	3-O-trans ferulylquinic acid	CYP1B1
445858	3-O-trans ferulylquinic acid	EGFR
445858	3-O-trans ferulylquinic acid	ESR2
445858	3-O-trans ferulylquinic acid	F3
445858	3-O-trans ferulylquinic acid	FBP1
445858	3-O-trans ferulylquinic acid	FYN
445858	3-O-trans ferulylquinic acid	GLO1
445858	3-O-trans ferulylquinic acid	HSD11B1
445858	3-O-trans ferulylquinic acid	KDM4C
445858	3-O-trans ferulylquinic acid	LCK
445858	3-O-trans ferulylquinic acid	MAOB
445858	3-O-trans ferulylquinic acid	MET
445858	3-O-trans ferulylquinic acid	MMP1
445858	3-O-trans ferulylquinic acid	MMP2
445858	3-O-trans ferulylquinic acid	MMP9
445858	3-O-trans ferulylquinic acid	NFE2L2
445858	3-O-trans ferulylquinic acid	NOS2
445858	3-O-trans ferulylquinic acid	NQO2
445858	3-O-trans ferulylquinic acid	PARP1
445858	3-O-trans ferulylquinic acid	PRKCE
445858	3-O-trans ferulylquinic acid	PTGS1
445858	3-O-trans ferulylquinic acid	PTGS2
445858	3-O-trans ferulylquinic acid	PTPN1
445858	3-O-trans ferulylquinic acid	RELA
445858	3-O-trans ferulylquinic acid	SLC16A1
445858	3-O-trans ferulylquinic acid	STAT3
445858	3-O-trans ferulylquinic acid	TLR4
445858	3-O-trans ferulylquinic acid	TLR9
445858	3-O-trans ferulylquinic acid	TOP2A
445858	3-O-trans ferulylquinic acid	TTR

445858	3-O-trans ferulylquinic acid	TUBB1
445858	3-O-trans ferulylquinic acid	TUBB3
98455	stepharine	ACHE
98455	stepharine	AURKA
98455	stepharine	AURKB
98455	stepharine	BDKRB2
98455	stepharine	CDC25C
98455	stepharine	CDC42
98455	stepharine	CDC7
98455	stepharine	CDK1
98455	stepharine	CHEK1
98455	stepharine	CHRM1
98455	stepharine	CHRM3
98455	stepharine	CHRM5
98455	stepharine	CHRNA4
98455	stepharine	CSF1R
98455	stepharine	DPP4
98455	stepharine	DPP9
98455	stepharine	DRD1
98455	stepharine	DRD2
98455	stepharine	DRD3
98455	stepharine	FAAH
98455	stepharine	FAP
98455	stepharine	HDAC1
98455	stepharine	HDAC6
98455	stepharine	HPGD
98455	stepharine	HTR1A
98455	stepharine	HTR1F
98455	stepharine	HTR3A
98455	stepharine	JAK1
98455	stepharine	JAK2
98455	stepharine	JAK3
98455	stepharine	KCNH2
98455	stepharine	KCNJ1
98455	stepharine	MALT1
98455	stepharine	MAP2K1
98455	stepharine	MC4R
98455	stepharine	MPO
98455	stepharine	MTNR1A
98455	stepharine	MTNR1B
98455	stepharine	OPRD1
98455	stepharine	OPRK1
98455	stepharine	OPRM1
98455	stepharine	PAOX

98455	stepharine	PARP2
98455	stepharine	PARP3
98455	stepharine	PIM2
98455	stepharine	PIM3
98455	stepharine	PKN2
98455	stepharine	PRKCD
98455	stepharine	PRKCE
98455	stepharine	PRKD1
98455	stepharine	PRKX
98455	stepharine	ROCK1
98455	stepharine	ROCK2
98455	stepharine	ROCK2
98455	stepharine	RPS6KA5
98455	stepharine	RPS6KB1
98455	stepharine	SIGMAR1
98455	stepharine	SLC6A4
98455	stepharine	TTK
98455	stepharine	TYK2
98455	stepharine	WDR5
98455	stepharine	WEE1
5280450	Linoleic Acid	ACP1
5280450	Linoleic Acid	ADORA3
5280450	Linoleic Acid	ADRA2B
5280450	Linoleic Acid	AKR1B10
5280450	Linoleic Acid	ALOX12
5280450	Linoleic Acid	ALOX15
5280450	Linoleic Acid	ALOX5
5280450	Linoleic Acid	ALOX5AP
5280450	Linoleic Acid	AR
5280450	Linoleic Acid	BCHE
5280450	Linoleic Acid	CD81
5280450	Linoleic Acid	CDC25A
5280450	Linoleic Acid	CDC25B
5280450	Linoleic Acid	CES2
5280450	Linoleic Acid	CMA1
5280450	Linoleic Acid	CNR1
5280450	Linoleic Acid	CTSG
5280450	Linoleic Acid	CYP17A1
5280450	Linoleic Acid	CYP19A1
5280450	Linoleic Acid	CYP26A1
5280450	Linoleic Acid	CYP26B1
5280450	Linoleic Acid	CYP51A1
5280450	Linoleic Acid	DRD2
5280450	Linoleic Acid	ENPP2

5280450	Linoleic Acid	ESR1
5280450	Linoleic Acid	ESR2
5280450	Linoleic Acid	FAAH
5280450	Linoleic Acid	FABP1
5280450	Linoleic Acid	FABP2
5280450	Linoleic Acid	FABP3
5280450	Linoleic Acid	FABP4
5280450	Linoleic Acid	FABP5
5280450	Linoleic Acid	FFAR1
5280450	Linoleic Acid	FFAR4
5280450	Linoleic Acid	FNTA
5280450	Linoleic Acid	G6PD
5280450	Linoleic Acid	GLUL
5280450	Linoleic Acid	GPBAR1
5280450	Linoleic Acid	GRM2
5280450	Linoleic Acid	HMGCR
5280450	Linoleic Acid	HSD11B1
5280450	Linoleic Acid	HSD11B2
5280450	Linoleic Acid	HTR2B
5280450	Linoleic Acid	IL6
5280450	Linoleic Acid	LTB4R
5280450	Linoleic Acid	MAPK1
5280450	Linoleic Acid	MAPK14
5280450	Linoleic Acid	MAPK3
5280450	Linoleic Acid	NOS2
5280450	Linoleic Acid	NPC1L1
5280450	Linoleic Acid	NR1H3
5280450	Linoleic Acid	NR3C1
5280450	Linoleic Acid	NR3C2
5280450	Linoleic Acid	PDE4D
5280450	Linoleic Acid	PGR
5280450	Linoleic Acid	PLA2G1B
5280450	Linoleic Acid	PLA2G4A
5280450	Linoleic Acid	POLB
5280450	Linoleic Acid	PPARA
5280450	Linoleic Acid	PPARD
5280450	Linoleic Acid	PPARG
5280450	Linoleic Acid	PREP
5280450	Linoleic Acid	PRKCH
5280450	Linoleic Acid	PSEN2
5280450	Linoleic Acid	PTGDR2
5280450	Linoleic Acid	PTGER1
5280450	Linoleic Acid	PTGER2
5280450	Linoleic Acid	PTGER4

5280450	Linoleic Acid	PTGES
5280450	Linoleic Acid	PTGIR
5280450	Linoleic Acid	PTGS1
5280450	Linoleic Acid	PTGS2
5280450	Linoleic Acid	PTPN1
5280450	Linoleic Acid	PTPN11
5280450	Linoleic Acid	PTPN2
5280450	Linoleic Acid	PTPN6
5280450	Linoleic Acid	PTPRF
5280450	Linoleic Acid	RBP4
5280450	Linoleic Acid	RORB
5280450	Linoleic Acid	RORC
5280450	Linoleic Acid	RXRB
5280450	Linoleic Acid	SCD
5280450	Linoleic Acid	SERPINA6
5280450	Linoleic Acid	SHBG
5280450	Linoleic Acid	SIGMAR1
5280450	Linoleic Acid	SLC16A1
5280450	Linoleic Acid	SRD5A2
5280450	Linoleic Acid	TERT
5280450	Linoleic Acid	TOP1
5280450	Linoleic Acid	TOP2A
5280450	Linoleic Acid	TRPV1
11005	Myristic Acid	ADRA2B
11005	Myristic Acid	AKR1B10
11005	Myristic Acid	AKR1C3
11005	Myristic Acid	ALOX12
11005	Myristic Acid	ALOX15
11005	Myristic Acid	AR
11005	Myristic Acid	BCL2L1
11005	Myristic Acid	CA1
11005	Myristic Acid	CA2
11005	Myristic Acid	CACNA2D1
11005	Myristic Acid	CDC25A
11005	Myristic Acid	CHRNA7
11005	Myristic Acid	CMA1
11005	Myristic Acid	CTSG
11005	Myristic Acid	CYP19A1
11005	Myristic Acid	CYP26A1
11005	Myristic Acid	CYP26B1
11005	Myristic Acid	EDNRA
11005	Myristic Acid	EPHX2
11005	Myristic Acid	FABP2
11005	Myristic Acid	FABP3

11005	Myristic Acid	FABP4
11005	Myristic Acid	FABP5
11005	Myristic Acid	FDFT1
11005	Myristic Acid	FFAR1
11005	Myristic Acid	FFAR4
11005	Myristic Acid	FNTA
11005	Myristic Acid	G6PD
11005	Myristic Acid	GABBR1
11005	Myristic Acid	GABRA2
11005	Myristic Acid	GPBAR1
11005	Myristic Acid	GRM5
11005	Myristic Acid	GSTK1
11005	Myristic Acid	HAO1
11005	Myristic Acid	HSD11B1
11005	Myristic Acid	HSD11B2
11005	Myristic Acid	HSD17B3
11005	Myristic Acid	HTR2B
11005	Myristic Acid	IMPDH2
11005	Myristic Acid	KDM2A
11005	Myristic Acid	KDM5C
11005	Myristic Acid	KEAP1
11005	Myristic Acid	MAPK1
11005	Myristic Acid	MAPK14
11005	Myristic Acid	MDM2
11005	Myristic Acid	NPC1L1
11005	Myristic Acid	NR1H4
11005	Myristic Acid	NR3C2
11005	Myristic Acid	PGR
11005	Myristic Acid	PHF8
11005	Myristic Acid	PLA2G4A
11005	Myristic Acid	PLG
11005	Myristic Acid	POLB
11005	Myristic Acid	PPARA
11005	Myristic Acid	PPARD
11005	Myristic Acid	PPARG
11005	Myristic Acid	PTGDR
11005	Myristic Acid	PTGDR2
11005	Myristic Acid	PTGER2
11005	Myristic Acid	PTGER3
11005	Myristic Acid	PTGER4
11005	Myristic Acid	PTGES
11005	Myristic Acid	PTGES2
11005	Myristic Acid	PTGFR
11005	Myristic Acid	PTPN1

11005	Myristic Acid	RARA
11005	Myristic Acid	RARB
11005	Myristic Acid	RARG
11005	Myristic Acid	RBP4
11005	Myristic Acid	RORA
11005	Myristic Acid	RORB
11005	Myristic Acid	RXRA
11005	Myristic Acid	RXRB
11005	Myristic Acid	RXRG
11005	Myristic Acid	SERPINA6
11005	Myristic Acid	SHBG
11005	Myristic Acid	SLC16A1
11005	Myristic Acid	SLC22A12
11005	Myristic Acid	SLC22A6
11005	Myristic Acid	TBXA2R
11005	Myristic Acid	TRPA1
11005	Myristic Acid	UGT2B7
11005	Myristic Acid	VDR
5281	Stearic Acid	AKR1B10
5281	Stearic Acid	AR
5281	Stearic Acid	CA1
5281	Stearic Acid	CA2
5281	Stearic Acid	CACNA2D1
5281	Stearic Acid	CDC25A
5281	Stearic Acid	CDC45
5281	Stearic Acid	CHRNA7
5281	Stearic Acid	CYP19A1
5281	Stearic Acid	CYP26A1
5281	Stearic Acid	CYP26B1
5281	Stearic Acid	FAAH
5281	Stearic Acid	FABP1
5281	Stearic Acid	FABP2
5281	Stearic Acid	FABP3
5281	Stearic Acid	FABP4
5281	Stearic Acid	FABP5
5281	Stearic Acid	FFAR1
5281	Stearic Acid	FNTA
5281	Stearic Acid	G6PD
5281	Stearic Acid	GABBR1
5281	Stearic Acid	GABRA2
5281	Stearic Acid	GLRA1
5281	Stearic Acid	GPBAR1
5281	Stearic Acid	GRM5
5281	Stearic Acid	GSTK1

5281	Stearic Acid	HAO1
5281	Stearic Acid	HMGCR
5281	Stearic Acid	HSD11B1
5281	Stearic Acid	HSD11B2
5281	Stearic Acid	HSD17B3
5281	Stearic Acid	KDM2A
5281	Stearic Acid	KDM5C
5281	Stearic Acid	NPC1L1
5281	Stearic Acid	NR0B2
5281	Stearic Acid	NR1H4
5281	Stearic Acid	PDE4A
5281	Stearic Acid	PDE4B
5281	Stearic Acid	PHF8
5281	Stearic Acid	PLG
5281	Stearic Acid	POLB
5281	Stearic Acid	PPARA
5281	Stearic Acid	PPARD
5281	Stearic Acid	PPARG
5281	Stearic Acid	PTGER2
5281	Stearic Acid	PTGER4
5281	Stearic Acid	PTGFR
5281	Stearic Acid	PTPN1
5281	Stearic Acid	PTPRC
5281	Stearic Acid	RARA
5281	Stearic Acid	RARB
5281	Stearic Acid	RARG
5281	Stearic Acid	RXRA
5281	Stearic Acid	RXRB
5281	Stearic Acid	RXRG
5281	Stearic Acid	SAE1
5281	Stearic Acid	SCD
5281	Stearic Acid	SERPINA6
5281	Stearic Acid	SHBG
5281	Stearic Acid	SLC16A1
5281	Stearic Acid	SLC22A6
5281	Stearic Acid	TERT
5281	Stearic Acid	UGT2B7
5281	Stearic Acid	VDR
222656	Malic Acid	ACLY
222656	Malic Acid	CHRNA7
222656	Malic Acid	EGLN1
222656	Malic Acid	FDFT1
938	Nicotinic Acid	DDO
938	Nicotinic Acid	FYN

938	Nicotinic Acid	HCAR2
938	Nicotinic Acid	LCK
938	Nicotinic Acid	SIRT2
938	Nicotinic Acid	SIRT3
938	Nicotinic Acid	SLC22A6
470606	Alphitolic Acid	ACP1
470606	Alphitolic Acid	ADORA3
470606	Alphitolic Acid	AGTR1
470606	Alphitolic Acid	AKR1B10
470606	Alphitolic Acid	ALOX5
470606	Alphitolic Acid	ALOX5AP
470606	Alphitolic Acid	AR
470606	Alphitolic Acid	BACE1
470606	Alphitolic Acid	CD81
470606	Alphitolic Acid	CDC25A
470606	Alphitolic Acid	CDC25B
470606	Alphitolic Acid	CES2
470606	Alphitolic Acid	CYP17A1
470606	Alphitolic Acid	CYP19A1
470606	Alphitolic Acid	CYP2C19
470606	Alphitolic Acid	CYP51A1
470606	Alphitolic Acid	ESR1
470606	Alphitolic Acid	ESR2
470606	Alphitolic Acid	FAAH
470606	Alphitolic Acid	FABP1
470606	Alphitolic Acid	FABP3
470606	Alphitolic Acid	FABP4
470606	Alphitolic Acid	FABP5
470606	Alphitolic Acid	FNTA
470606	Alphitolic Acid	G6PD
470606	Alphitolic Acid	GPBAR1
470606	Alphitolic Acid	HMGCR
470606	Alphitolic Acid	HSD11B1
470606	Alphitolic Acid	HSD11B2
470606	Alphitolic Acid	LTB4R
470606	Alphitolic Acid	NOS2
470606	Alphitolic Acid	NPC1L1
470606	Alphitolic Acid	NR1H3
470606	Alphitolic Acid	NR1H4
470606	Alphitolic Acid	NR3C1
470606	Alphitolic Acid	NR3C2
470606	Alphitolic Acid	PDE4D
470606	Alphitolic Acid	PGR
470606	Alphitolic Acid	PLA2G1B

470606	Alphitolic Acid	PLA2G4A
470606	Alphitolic Acid	POLB
470606	Alphitolic Acid	PPARA
470606	Alphitolic Acid	PPARD
470606	Alphitolic Acid	PPARG
470606	Alphitolic Acid	PREP
470606	Alphitolic Acid	PRKCH
470606	Alphitolic Acid	PTGDR
470606	Alphitolic Acid	PTGER1
470606	Alphitolic Acid	PTGER2
470606	Alphitolic Acid	PTGER4
470606	Alphitolic Acid	PTGES
470606	Alphitolic Acid	PTGFR
470606	Alphitolic Acid	PTGIR
470606	Alphitolic Acid	PTGS1
470606	Alphitolic Acid	PTGS2
470606	Alphitolic Acid	PTPN1
470606	Alphitolic Acid	PTPN11
470606	Alphitolic Acid	PTPN2
470606	Alphitolic Acid	PTPN6
470606	Alphitolic Acid	PTPRF
470606	Alphitolic Acid	RORA
470606	Alphitolic Acid	RORC
470606	Alphitolic Acid	SAE1
470606	Alphitolic Acid	SCD
470606	Alphitolic Acid	SERPINA6
470606	Alphitolic Acid	SHBG
470606	Alphitolic Acid	SIGMAR1
470606	Alphitolic Acid	SLC22A6
470606	Alphitolic Acid	SLC6A3
470606	Alphitolic Acid	SRD5A2
470606	Alphitolic Acid	TLR9
470606	Alphitolic Acid	TOP1
470606	Alphitolic Acid	TOP2A
445638	Palmitoleic Acid	ACHE
445638	Palmitoleic Acid	ACP1
445638	Palmitoleic Acid	ADORA3
445638	Palmitoleic Acid	AKR1B10
445638	Palmitoleic Acid	ALOX12
445638	Palmitoleic Acid	ALOX5
445638	Palmitoleic Acid	AR
445638	Palmitoleic Acid	BACE1
445638	Palmitoleic Acid	BCHE
445638	Palmitoleic Acid	BCL2

445638	Palmitoleic Acid	CD81
445638	Palmitoleic Acid	CDC25A
445638	Palmitoleic Acid	CDC25B
445638	Palmitoleic Acid	CES2
445638	Palmitoleic Acid	CHRM2
445638	Palmitoleic Acid	CMA1
445638	Palmitoleic Acid	CNR1
445638	Palmitoleic Acid	CTSG
445638	Palmitoleic Acid	CYP17A1
445638	Palmitoleic Acid	CYP19A1
445638	Palmitoleic Acid	CYP26A1
445638	Palmitoleic Acid	CYP26B1
445638	Palmitoleic Acid	CYP2C19
445638	Palmitoleic Acid	CYP51A1
445638	Palmitoleic Acid	EDNRA
445638	Palmitoleic Acid	ENPP2
445638	Palmitoleic Acid	ESR1
445638	Palmitoleic Acid	ESR2
445638	Palmitoleic Acid	FAAH
445638	Palmitoleic Acid	FABP1
445638	Palmitoleic Acid	FABP2
445638	Palmitoleic Acid	FABP3
445638	Palmitoleic Acid	FABP4
445638	Palmitoleic Acid	FABP5
445638	Palmitoleic Acid	FDFT1
445638	Palmitoleic Acid	FFAR1
445638	Palmitoleic Acid	FFAR4
445638	Palmitoleic Acid	FNTA
445638	Palmitoleic Acid	G6PD
445638	Palmitoleic Acid	HMGCR
445638	Palmitoleic Acid	HNF4A
445638	Palmitoleic Acid	HSD11B1
445638	Palmitoleic Acid	HSD11B2
445638	Palmitoleic Acid	IMPDH2
445638	Palmitoleic Acid	LTB4R
445638	Palmitoleic Acid	MAPK3
445638	Palmitoleic Acid	MCL1
445638	Palmitoleic Acid	MDM2
445638	Palmitoleic Acid	NOS2
445638	Palmitoleic Acid	NPC1L1
445638	Palmitoleic Acid	NR1H3
445638	Palmitoleic Acid	NR1H4
445638	Palmitoleic Acid	NR1I3
445638	Palmitoleic Acid	NR3C1

445638	Palmitoleic Acid	NR3C2
445638	Palmitoleic Acid	OXER1
445638	Palmitoleic Acid	PDE4D
445638	Palmitoleic Acid	PGR
445638	Palmitoleic Acid	PIM2
445638	Palmitoleic Acid	PLA2G1B
445638	Palmitoleic Acid	POLB
445638	Palmitoleic Acid	PPARA
445638	Palmitoleic Acid	PPARD
445638	Palmitoleic Acid	PPARG
445638	Palmitoleic Acid	PREP
445638	Palmitoleic Acid	PRKCH
445638	Palmitoleic Acid	PTGDR
445638	Palmitoleic Acid	PTGDR2
445638	Palmitoleic Acid	PTGER1
445638	Palmitoleic Acid	PTGER2
445638	Palmitoleic Acid	PTGER3
445638	Palmitoleic Acid	PTGER4
445638	Palmitoleic Acid	PTGES
445638	Palmitoleic Acid	PTGIR
445638	Palmitoleic Acid	PTGS1
445638	Palmitoleic Acid	PTGS2
445638	Palmitoleic Acid	PTPN1
445638	Palmitoleic Acid	PTPN11
445638	Palmitoleic Acid	PTPN2
445638	Palmitoleic Acid	PTPN6
445638	Palmitoleic Acid	PTPRF
445638	Palmitoleic Acid	RORA
445638	Palmitoleic Acid	RORC
445638	Palmitoleic Acid	SCD
445638	Palmitoleic Acid	SERPINA6
445638	Palmitoleic Acid	SHBG
445638	Palmitoleic Acid	SIGMAR1
445638	Palmitoleic Acid	SLC16A1
445638	Palmitoleic Acid	SLC22A12
445638	Palmitoleic Acid	SLC6A2
445638	Palmitoleic Acid	SLC6A3
445638	Palmitoleic Acid	SLC6A4
445638	Palmitoleic Acid	SRD5A2
445638	Palmitoleic Acid	TERT
445638	Palmitoleic Acid	THRA
445638	Palmitoleic Acid	THRB
445638	Palmitoleic Acid	TOP1
445638	Palmitoleic Acid	TOP2A

445638	Palmitoleic Acid	TRPM8
445638	Palmitoleic Acid	TRPV1
160875	Asimilobine	ABAT
160875	Asimilobine	ABCB1
160875	Asimilobine	ADCY5
160875	Asimilobine	ADRA1A
160875	Asimilobine	ADRA1B
160875	Asimilobine	ADRA1D
160875	Asimilobine	ADRA2A
160875	Asimilobine	ADRA2B
160875	Asimilobine	ADRA2C
160875	Asimilobine	ADRB1
160875	Asimilobine	ADRB2
160875	Asimilobine	ADRB3
160875	Asimilobine	ALOX12
160875	Asimilobine	ALOX15
160875	Asimilobine	AOC3
160875	Asimilobine	CCL2
160875	Asimilobine	CDK9
160875	Asimilobine	CHRNA3
160875	Asimilobine	CHRNA4
160875	Asimilobine	CHRNA4
160875	Asimilobine	CHRNA7
160875	Asimilobine	CHRNB1
160875	Asimilobine	CHRNB3
160875	Asimilobine	CTSC
160875	Asimilobine	CYP1A2
160875	Asimilobine	DPP4
160875	Asimilobine	DPP9
160875	Asimilobine	DRD1
160875	Asimilobine	DRD2
160875	Asimilobine	DRD3
160875	Asimilobine	DRD4
160875	Asimilobine	DRD5
160875	Asimilobine	DYRK2
160875	Asimilobine	F3
160875	Asimilobine	FAP
160875	Asimilobine	GABBR2
160875	Asimilobine	GABRA1
160875	Asimilobine	GABRA2
160875	Asimilobine	GABRA3
160875	Asimilobine	GABRR1
160875	Asimilobine	HASPIN
160875	Asimilobine	HRH2

160875	Asimilobine	HTR1A
160875	Asimilobine	HTR1B
160875	Asimilobine	HTR1D
160875	Asimilobine	HTR1E
160875	Asimilobine	HTR1F
160875	Asimilobine	HTR2A
160875	Asimilobine	HTR2B
160875	Asimilobine	HTR2C
160875	Asimilobine	HTR3A
160875	Asimilobine	HTR4
160875	Asimilobine	HTR5A
160875	Asimilobine	HTR6
160875	Asimilobine	HTR7
160875	Asimilobine	IDO1
160875	Asimilobine	IKBKB
160875	Asimilobine	KAT2B
160875	Asimilobine	KCNH2
160875	Asimilobine	KISS1R
160875	Asimilobine	LTA4H
160875	Asimilobine	MAOA
160875	Asimilobine	MAPK8
160875	Asimilobine	MYLK
160875	Asimilobine	NOS2
160875	Asimilobine	NR4A1
160875	Asimilobine	OPRD1
160875	Asimilobine	OPRK1
160875	Asimilobine	OPRM1
160875	Asimilobine	PHLPP2
160875	Asimilobine	PKN1
160875	Asimilobine	PKN2
160875	Asimilobine	PLA2G2A
160875	Asimilobine	PRKCE
160875	Asimilobine	PRKX
160875	Asimilobine	PTPRCAP
160875	Asimilobine	QDPR
160875	Asimilobine	RCOR1
160875	Asimilobine	ROCK1
160875	Asimilobine	ROCK2
160875	Asimilobine	ROCK2
160875	Asimilobine	RPS6KA5
160875	Asimilobine	SCN2A
160875	Asimilobine	SCN4A
160875	Asimilobine	SIGMAR1
160875	Asimilobine	SLC6A1

160875	Asimilobine	SLC6A11
160875	Asimilobine	SLC6A13
160875	Asimilobine	SLC6A3
160875	Asimilobine	SLC6A4
160875	Asimilobine	TAAR1
160875	Asimilobine	TBXA2R
160875	Asimilobine	TH
160875	Asimilobine	TNKS2
160875	Asimilobine	TSPO
289	Catechol	CA1
289	Catechol	CA12
289	Catechol	CA2
289	Catechol	CA3
289	Catechol	CA4
289	Catechol	CA5A
289	Catechol	CA5B
289	Catechol	CA6
289	Catechol	CA9
289	Catechol	EGFR
289	Catechol	FYN
289	Catechol	IDO1
289	Catechol	PTPN22
5281707	coumestrol	ABCC1
5281707	coumestrol	ACHE
5281707	coumestrol	AHR
5281707	coumestrol	AKR1B1
5281707	coumestrol	AKT1
5281707	coumestrol	ALOX12
5281707	coumestrol	ALOX5
5281707	coumestrol	APP
5281707	coumestrol	ARG1
5281707	coumestrol	AURKA
5281707	coumestrol	AURKB
5281707	coumestrol	BACE1
5281707	coumestrol	CA1
5281707	coumestrol	CA12
5281707	coumestrol	CA13
5281707	coumestrol	CA14
5281707	coumestrol	CA2
5281707	coumestrol	CA4
5281707	coumestrol	CA5A
5281707	coumestrol	CA6
5281707	coumestrol	CA7
5281707	coumestrol	CA9

5281707	coumestrol	CBR1
5281707	coumestrol	CCNB3
5281707	coumestrol	CCND1
5281707	coumestrol	CD38
5281707	coumestrol	CDK2
5281707	coumestrol	CDK2
5281707	coumestrol	CDK5R1
5281707	coumestrol	CDK6
5281707	coumestrol	CFTR
5281707	coumestrol	CRHR1
5281707	coumestrol	CSNK2A1
5281707	coumestrol	DRD3
5281707	coumestrol	EGFR
5281707	coumestrol	EPHB4
5281707	coumestrol	ESR1
5281707	coumestrol	ESR2
5281707	coumestrol	FGR
5281707	coumestrol	FLT3
5281707	coumestrol	FLT4
5281707	coumestrol	GLO1
5281707	coumestrol	GPR35
5281707	coumestrol	GSK3B
5281707	coumestrol	GSR
5281707	coumestrol	HSD17B2
5281707	coumestrol	HSP90AA1
5281707	coumestrol	IGF1R
5281707	coumestrol	KDR
5281707	coumestrol	LYN
5281707	coumestrol	MAOA
5281707	coumestrol	MAP3K8
5281707	coumestrol	MET
5281707	coumestrol	MMP12
5281707	coumestrol	MMP2
5281707	coumestrol	MMP9
5281707	coumestrol	NFKB1
5281707	coumestrol	NOX4
5281707	coumestrol	NUAK1
5281707	coumestrol	PARP1
5281707	coumestrol	PDGFRB
5281707	coumestrol	PFKFB3
5281707	coumestrol	PLK1
5281707	coumestrol	PLK4
5281707	coumestrol	PTGS2
5281707	coumestrol	PTK2

5281707	coumestrol	PTP4A3
5281707	coumestrol	PTPRS
5281707	coumestrol	SQLE
5281707	coumestrol	SRC
5281707	coumestrol	SYK
5281707	coumestrol	TEK
5281707	coumestrol	TERT
5281707	coumestrol	TNKS
5281707	coumestrol	TNKS2
5281707	coumestrol	TOP1
5281707	coumestrol	TTR
5281707	coumestrol	TYR
5281707	coumestrol	XDH
21672700	Colubrinic Acid	ACE
21672700	Colubrinic Acid	ACP1
21672700	Colubrinic Acid	AGTR1
21672700	Colubrinic Acid	AKR1B10
21672700	Colubrinic Acid	ALOX5AP
21672700	Colubrinic Acid	AMPD2
21672700	Colubrinic Acid	AR
21672700	Colubrinic Acid	CD81
21672700	Colubrinic Acid	CDC25A
21672700	Colubrinic Acid	CDC25C
21672700	Colubrinic Acid	CES2
21672700	Colubrinic Acid	CYP17A1
21672700	Colubrinic Acid	CYP19A1
21672700	Colubrinic Acid	CYP51A1
21672700	Colubrinic Acid	FABP1
21672700	Colubrinic Acid	GABBR1
21672700	Colubrinic Acid	GPBAR1
21672700	Colubrinic Acid	HMGCR
21672700	Colubrinic Acid	HSD11B1
21672700	Colubrinic Acid	HSD17B3
21672700	Colubrinic Acid	MDM2
21672700	Colubrinic Acid	MMP1
21672700	Colubrinic Acid	MMP2
21672700	Colubrinic Acid	MMP3
21672700	Colubrinic Acid	NPC1L1
21672700	Colubrinic Acid	NR1H4
21672700	Colubrinic Acid	NR3C1
21672700	Colubrinic Acid	PGR
21672700	Colubrinic Acid	PLA2G4A
21672700	Colubrinic Acid	POLB
21672700	Colubrinic Acid	PPARG

21672700	Colubrinic Acid	PREP
21672700	Colubrinic Acid	PTGER1
21672700	Colubrinic Acid	PTGER2
21672700	Colubrinic Acid	PTGER4
21672700	Colubrinic Acid	PTGES
21672700	Colubrinic Acid	PTGS2
21672700	Colubrinic Acid	PTPN1
21672700	Colubrinic Acid	PTPN11
21672700	Colubrinic Acid	PTPN2
21672700	Colubrinic Acid	PTPRF
21672700	Colubrinic Acid	RARG
21672700	Colubrinic Acid	RORC
21672700	Colubrinic Acid	RXRG
21672700	Colubrinic Acid	SAE1
21672700	Colubrinic Acid	SCD
21672700	Colubrinic Acid	SIGMAR1
21672700	Colubrinic Acid	TERT
21672700	Colubrinic Acid	THRA
21672700	Colubrinic Acid	THRB
21672700	Colubrinic Acid	TOP1
21672700	Colubrinic Acid	TOP2A
21672700	Colubrinic Acid	UGT2B7
21672700	Colubrinic Acid	VDR
73659	2Î‘,3Î’-Dihydroxyolean-12-En-28-Oic Acid	ACP1
73659	2Î‘,3Î’-Dihydroxyolean-12-En-28-Oic Acid	AGTR1
73659	2Î‘,3Î’-Dihydroxyolean-12-En-28-Oic Acid	AKR1B10
73659	2Î‘,3Î’-Dihydroxyolean-12-En-28-Oic Acid	ALOX5
73659	2Î‘,3Î’-Dihydroxyolean-12-En-28-Oic Acid	ALOX5AP
73659	2Î‘,3Î’-Dihydroxyolean-12-En-28-Oic Acid	AR
73659	2Î‘,3Î’-Dihydroxyolean-12-En-28-Oic Acid	BACE1
73659	2Î‘,3Î’-Dihydroxyolean-12-En-28-Oic Acid	BCHE
73659	2Î‘,3Î’-Dihydroxyolean-12-En-28-Oic Acid	CD81
73659	2Î‘,3Î’-Dihydroxyolean-12-En-28-Oic Acid	CDC25A

73659	2Î‘,3Î’-Dihydroxyolean-12-En-28-Oic Acid	CDC25B
73659	2Î‘,3Î’-Dihydroxyolean-12-En-28-Oic Acid	CES2
73659	2Î‘,3Î’-Dihydroxyolean-12-En-28-Oic Acid	CHRM2
73659	2Î‘,3Î’-Dihydroxyolean-12-En-28-Oic Acid	CYP17A1
73659	2Î‘,3Î’-Dihydroxyolean-12-En-28-Oic Acid	CYP19A1
73659	2Î‘,3Î’-Dihydroxyolean-12-En-28-Oic Acid	CYP51A1
73659	2Î‘,3Î’-Dihydroxyolean-12-En-28-Oic Acid	ESR1
73659	2Î‘,3Î’-Dihydroxyolean-12-En-28-Oic Acid	ESR2
73659	2Î‘,3Î’-Dihydroxyolean-12-En-28-Oic Acid	FAAH
73659	2Î‘,3Î’-Dihydroxyolean-12-En-28-Oic Acid	FABP1
73659	2Î‘,3Î’-Dihydroxyolean-12-En-28-Oic Acid	FABP3
73659	2Î‘,3Î’-Dihydroxyolean-12-En-28-Oic Acid	FABP4
73659	2Î‘,3Î’-Dihydroxyolean-12-En-28-Oic Acid	FABP5
73659	2Î‘,3Î’-Dihydroxyolean-12-En-28-Oic Acid	FFAR1
73659	2Î‘,3Î’-Dihydroxyolean-12-En-28-Oic Acid	FNTA
73659	2Î‘,3Î’-Dihydroxyolean-12-En-28-Oic Acid	G6PD
73659	2Î‘,3Î’-Dihydroxyolean-12-En-28-Oic Acid	GPBAR1
73659	2Î‘,3Î’-Dihydroxyolean-12-En-28-Oic Acid	GRIK1
73659	2Î‘,3Î’-Dihydroxyolean-12-En-28-Oic Acid	GRIK2
73659	2Î‘,3Î’-Dihydroxyolean-12-En-28-Oic Acid	HMGCR
73659	2Î‘,3Î’-Dihydroxyolean-12-En-28-Oic Acid	HSD11B1
73659	2Î‘,3Î’-Dihydroxyolean-12-En-28-Oic Acid	HSD11B2

73659	2Î‘,3Î’-Dihydroxyolean-12-En-28-Oic Acid	LTB4R
73659	2Î‘,3Î’-Dihydroxyolean-12-En-28-Oic Acid	MDM2
73659	2Î‘,3Î’-Dihydroxyolean-12-En-28-Oic Acid	NOS2
73659	2Î‘,3Î’-Dihydroxyolean-12-En-28-Oic Acid	NR1H3
73659	2Î‘,3Î’-Dihydroxyolean-12-En-28-Oic Acid	NR1H4
73659	2Î‘,3Î’-Dihydroxyolean-12-En-28-Oic Acid	NR3C1
73659	2Î‘,3Î’-Dihydroxyolean-12-En-28-Oic Acid	NR3C2
73659	2Î‘,3Î’-Dihydroxyolean-12-En-28-Oic Acid	PDE4D
73659	2Î‘,3Î’-Dihydroxyolean-12-En-28-Oic Acid	PGR
73659	2Î‘,3Î’-Dihydroxyolean-12-En-28-Oic Acid	PLA2G1B
73659	2Î‘,3Î’-Dihydroxyolean-12-En-28-Oic Acid	POLB
73659	2Î‘,3Î’-Dihydroxyolean-12-En-28-Oic Acid	PPARA
73659	2Î‘,3Î’-Dihydroxyolean-12-En-28-Oic Acid	PPARD
73659	2Î‘,3Î’-Dihydroxyolean-12-En-28-Oic Acid	PPARG
73659	2Î‘,3Î’-Dihydroxyolean-12-En-28-Oic Acid	PREP
73659	2Î‘,3Î’-Dihydroxyolean-12-En-28-Oic Acid	PRKCH
73659	2Î‘,3Î’-Dihydroxyolean-12-En-28-Oic Acid	PTGDR2
73659	2Î‘,3Î’-Dihydroxyolean-12-En-28-Oic Acid	PTGER1
73659	2Î‘,3Î’-Dihydroxyolean-12-En-28-Oic Acid	PTGER2
73659	2Î‘,3Î’-Dihydroxyolean-12-En-28-Oic Acid	PTGER4
73659	2Î‘,3Î’-Dihydroxyolean-12-En-28-Oic Acid	PTGES
73659	2Î‘,3Î’-Dihydroxyolean-12-En-28-Oic Acid	PTGIR

73659	2Î‘,3Î’-Dihydroxyolean-12-En-28-Oic Acid	PTGS1
73659	2Î‘,3Î’-Dihydroxyolean-12-En-28-Oic Acid	PTGS2
73659	2Î‘,3Î’-Dihydroxyolean-12-En-28-Oic Acid	PTPN1
73659	2Î‘,3Î’-Dihydroxyolean-12-En-28-Oic Acid	PTPN11
73659	2Î‘,3Î’-Dihydroxyolean-12-En-28-Oic Acid	PTPN2
73659	2Î‘,3Î’-Dihydroxyolean-12-En-28-Oic Acid	PTPN6
73659	2Î‘,3Î’-Dihydroxyolean-12-En-28-Oic Acid	PTPRF
73659	2Î‘,3Î’-Dihydroxyolean-12-En-28-Oic Acid	RORA
73659	2Î‘,3Î’-Dihydroxyolean-12-En-28-Oic Acid	RORC
73659	2Î‘,3Î’-Dihydroxyolean-12-En-28-Oic Acid	SCD
73659	2Î‘,3Î’-Dihydroxyolean-12-En-28-Oic Acid	SERPINA6
73659	2Î‘,3Î’-Dihydroxyolean-12-En-28-Oic Acid	SHBG
73659	2Î‘,3Î’-Dihydroxyolean-12-En-28-Oic Acid	SLC10A1
73659	2Î‘,3Î’-Dihydroxyolean-12-En-28-Oic Acid	SLC10A2
73659	2Î‘,3Î’-Dihydroxyolean-12-En-28-Oic Acid	SLC6A3
73659	2Î‘,3Î’-Dihydroxyolean-12-En-28-Oic Acid	SLC6A4
73659	2Î‘,3Î’-Dihydroxyolean-12-En-28-Oic Acid	SRD5A2
73659	2Î‘,3Î’-Dihydroxyolean-12-En-28-Oic Acid	TERT
73659	2Î‘,3Î’-Dihydroxyolean-12-En-28-Oic Acid	TLR9
73659	2Î‘,3Î’-Dihydroxyolean-12-En-28-Oic Acid	TOP1
73659	2Î‘,3Î’-Dihydroxyolean-12-En-28-Oic Acid	TOP2A
1130	Vitamin B1	ABCC1
1130	Vitamin B1	ABCG2

1130	Vitamin B1	ADAMTS5
1130	Vitamin B1	AKR1C3
1130	Vitamin B1	BCL2
1130	Vitamin B1	CA1
1130	Vitamin B1	CA2
1130	Vitamin B1	CHRM1
1130	Vitamin B1	CHRM2
1130	Vitamin B1	CHRM3
1130	Vitamin B1	CSF1R
1130	Vitamin B1	CTSL
1130	Vitamin B1	CTSV
1130	Vitamin B1	CYP26A1
1130	Vitamin B1	CYP26B1
1130	Vitamin B1	FABP4
1130	Vitamin B1	GRIN1
1130	Vitamin B1	GRM2
1130	Vitamin B1	HCAR2
1130	Vitamin B1	HPGD
1130	Vitamin B1	MAPK8
1130	Vitamin B1	MCL1
1130	Vitamin B1	MDM2
1130	Vitamin B1	METAP2
1130	Vitamin B1	MMP1
1130	Vitamin B1	MMP14
1130	Vitamin B1	NR1H4
1130	Vitamin B1	PPARA
1130	Vitamin B1	PPARD
1130	Vitamin B1	PPARG
1130	Vitamin B1	PTGER1
1130	Vitamin B1	RBP4
1130	Vitamin B1	TKT
6443026	Mauritine D	ABL1
6443026	Mauritine D	ACACA
6443026	Mauritine D	ACACB
6443026	Mauritine D	ADRA2A
6443026	Mauritine D	ADRA2B
6443026	Mauritine D	AHCY
6443026	Mauritine D	ALK
6443026	Mauritine D	AURKB
6443026	Mauritine D	BAD
6443026	Mauritine D	BCL2
6443026	Mauritine D	BIRC8
6443026	Mauritine D	BLK
6443026	Mauritine D	BRAF

6443026	Mauritine D	BTK
6443026	Mauritine D	CCR3
6443026	Mauritine D	CCR4
6443026	Mauritine D	CDK1
6443026	Mauritine D	CDK2
6443026	Mauritine D	CNR2
6443026	Mauritine D	CSF1R
6443026	Mauritine D	CXCR4
6443026	Mauritine D	DRD1
6443026	Mauritine D	DRD3
6443026	Mauritine D	EGFR
6443026	Mauritine D	EPHA2
6443026	Mauritine D	ERBB2
6443026	Mauritine D	ERN1
6443026	Mauritine D	ESR2
6443026	Mauritine D	FGFR1
6443026	Mauritine D	FLT1
6443026	Mauritine D	FLT3
6443026	Mauritine D	FLT4
6443026	Mauritine D	GNRHR
6443026	Mauritine D	GRK7
6443026	Mauritine D	GSK3B
6443026	Mauritine D	HCN1
6443026	Mauritine D	HCN4
6443026	Mauritine D	HIPK4
6443026	Mauritine D	HTR1D
6443026	Mauritine D	HTR6
6443026	Mauritine D	ICK
6443026	Mauritine D	IDH1
6443026	Mauritine D	IGF1R
6443026	Mauritine D	IKBKB
6443026	Mauritine D	IKBKG
6443026	Mauritine D	INSR
6443026	Mauritine D	JAK2
6443026	Mauritine D	JAK3
6443026	Mauritine D	KCNA5
6443026	Mauritine D	KCNJ5
6443026	Mauritine D	KDR
6443026	Mauritine D	KIT
6443026	Mauritine D	LRRK2
6443026	Mauritine D	MAP2K3
6443026	Mauritine D	MAP3K12
6443026	Mauritine D	MAP3K13
6443026	Mauritine D	MAP3K15

6443026	Mauritine D	MAPK14
6443026	Mauritine D	MAPK8
6443026	Mauritine D	MAST1
6443026	Mauritine D	MC5R
6443026	Mauritine D	MCHR1
6443026	Mauritine D	MET
6443026	Mauritine D	NPY1R
6443026	Mauritine D	NPY2R
6443026	Mauritine D	NPY5R
6443026	Mauritine D	OPRL1
6443026	Mauritine D	OXSR1
6443026	Mauritine D	PARP1
6443026	Mauritine D	PDE11A
6443026	Mauritine D	PDE4B
6443026	Mauritine D	PDE4D
6443026	Mauritine D	PDE5A
6443026	Mauritine D	PDGFRA
6443026	Mauritine D	PDGFRB
6443026	Mauritine D	PHKG2
6443026	Mauritine D	PIK3CD
6443026	Mauritine D	PLK1
6443026	Mauritine D	PLK4
6443026	Mauritine D	PRCP
6443026	Mauritine D	PRKAA2
6443026	Mauritine D	PRKCA
6443026	Mauritine D	PRKCB
6443026	Mauritine D	PRKCD
6443026	Mauritine D	PRKCG
6443026	Mauritine D	PRKCZ
6443026	Mauritine D	PRPF4B
6443026	Mauritine D	PTPN22
6443026	Mauritine D	RET
6443026	Mauritine D	RPS6KA3
6443026	Mauritine D	SBK1
6443026	Mauritine D	SLC10A2
6443026	Mauritine D	SSTR3
6443026	Mauritine D	STK39
6443026	Mauritine D	TACR3
6443026	Mauritine D	TBK1
6443026	Mauritine D	TGFBR1
6443026	Mauritine D	TUBB1
6443026	Mauritine D	VDR
6443026	Mauritine D	YES1
2353	berberine	CYP2D6

2353	berberine	ABL1
2353	berberine	ACHE
2353	berberine	ADRA2B
2353	berberine	ADRA2C
2353	berberine	ALOX5AP
2353	berberine	AURKA
2353	berberine	AURKB
2353	berberine	BCAT2
2353	berberine	BCHE
2353	berberine	CCNA2
2353	berberine	CDC25B
2353	berberine	CDC42
2353	berberine	CDK2
2353	berberine	CDK4
2353	berberine	CHEK1
2353	berberine	CHEK2
2353	berberine	CHRM1
2353	berberine	CHRM4
2353	berberine	CSF1R
2353	berberine	CYP11B1
2353	berberine	CYP11B2
2353	berberine	DHFR
2353	berberine	EPHB4
2353	berberine	EPHX2
2353	berberine	F3
2353	berberine	FLT3
2353	berberine	GABRB3
2353	berberine	GABRB3
2353	berberine	GABRB3
2353	berberine	GRIA1
2353	berberine	HCRTR2
2353	berberine	HPGD
2353	berberine	HSD11B1
2353	berberine	HSD17B1
2353	berberine	HSD17B2
2353	berberine	HTR2B
2353	berberine	HTR3A
2353	berberine	ICAM1
2353	berberine	IKBKB
2353	berberine	JAK2
2353	berberine	KIT
2353	berberine	LCK
2353	berberine	LIMK1
2353	berberine	LYN

2353	berberine	MAOB
2353	berberine	MAP2K1
2353	berberine	MAPK14
2353	berberine	MAPK8
2353	berberine	MAPKAPK2
2353	berberine	MET
2353	berberine	MKNK1
2353	berberine	NTRK1
2353	berberine	PARP10
2353	berberine	PARP2
2353	berberine	PFKFB3
2353	berberine	PIK3CB
2353	berberine	PIK3CD
2353	berberine	PIK3CG
2353	berberine	PIM1
2353	berberine	PPIA
2353	berberine	PRF1
2353	berberine	PTGS2
2353	berberine	PTPN1
2353	berberine	RAC1
2353	berberine	ROCK2
2353	berberine	RPS27
2353	berberine	RPS6KB1
2353	berberine	SAE1
2353	berberine	SCN4A
2353	berberine	SELE
2353	berberine	SIGMAR1
2353	berberine	SLC1A3
2353	berberine	SRC
2353	berberine	TBXAS1
2353	berberine	TEK
2353	berberine	TRPC6
2353	berberine	TRPM8
2353	berberine	TYMS
2353	berberine	ZAP70
101650325	Ruvoside	ATP12A
101650325	Ruvoside	ATP1A1
101650325	Ruvoside	BACE1
101650325	Ruvoside	BCL2L1
101650325	Ruvoside	CDC25A
101650325	Ruvoside	CDC25B
101650325	Ruvoside	CTSD
101650325	Ruvoside	F2RL1
101650325	Ruvoside	GLRA1

101650325	Ruvoside	GLRA2
101650325	Ruvoside	IARS
101650325	Ruvoside	KMT5A
101650325	Ruvoside	PLA2G1B
101650325	Ruvoside	PPP2CA
101650325	Ruvoside	PRKCD
101650325	Ruvoside	PRKCE
101650325	Ruvoside	PRKCH
101650325	Ruvoside	PRKCQ
101650325	Ruvoside	PTPA
101650325	Ruvoside	PTPN1
101650325	Ruvoside	RORC
101650325	Ruvoside	RPS6KA5
101650325	Ruvoside	STAT3
101650325	Ruvoside	SYK
10146	Nuciferine	CYP2D6
10146	Nuciferine	ABCB1
10146	Nuciferine	ADORA3
10146	Nuciferine	ADRA1A
10146	Nuciferine	ADRA1B
10146	Nuciferine	ADRA1D
10146	Nuciferine	ADRA2A
10146	Nuciferine	ADRA2B
10146	Nuciferine	ADRA2C
10146	Nuciferine	ADRB1
10146	Nuciferine	ALOX12
10146	Nuciferine	ALOX15
10146	Nuciferine	BIRC2
10146	Nuciferine	CDK2
10146	Nuciferine	CDK9
10146	Nuciferine	CHRM1
10146	Nuciferine	CHRM2
10146	Nuciferine	CHRM3
10146	Nuciferine	CHRM4
10146	Nuciferine	CHRNA3
10146	Nuciferine	CHRNA4
10146	Nuciferine	CHRNA7
10146	Nuciferine	CHRNB1
10146	Nuciferine	CHRNB3
10146	Nuciferine	CYP1A2
10146	Nuciferine	DPP4
10146	Nuciferine	DRD1
10146	Nuciferine	DRD2
10146	Nuciferine	DRD3

10146	Nuciferine	DRD4
10146	Nuciferine	DRD5
10146	Nuciferine	EGFR
10146	Nuciferine	F3
10146	Nuciferine	FDFT1
10146	Nuciferine	FLT3
10146	Nuciferine	HASPIN
10146	Nuciferine	HPGDS
10146	Nuciferine	HRH2
10146	Nuciferine	HRH4
10146	Nuciferine	HTR1A
10146	Nuciferine	HTR1B
10146	Nuciferine	HTR1D
10146	Nuciferine	HTR1E
10146	Nuciferine	HTR1F
10146	Nuciferine	HTR2A
10146	Nuciferine	HTR2B
10146	Nuciferine	HTR2C
10146	Nuciferine	HTR3A
10146	Nuciferine	HTR5A
10146	Nuciferine	HTR6
10146	Nuciferine	HTR7
10146	Nuciferine	IRAK4
10146	Nuciferine	JAK2
10146	Nuciferine	KCNH2
10146	Nuciferine	LTA4H
10146	Nuciferine	MAOA
10146	Nuciferine	MET
10146	Nuciferine	NOS2
10146	Nuciferine	NR4A1
10146	Nuciferine	OPRD1
10146	Nuciferine	OPRK1
10146	Nuciferine	OPRM1
10146	Nuciferine	PHLPP2
10146	Nuciferine	PIM1
10146	Nuciferine	PIM2
10146	Nuciferine	PRKCQ
10146	Nuciferine	PTGS2
10146	Nuciferine	PTPRCAP
10146	Nuciferine	ROCK1
10146	Nuciferine	ROCK2
10146	Nuciferine	SCN4A
10146	Nuciferine	SIGMAR1
10146	Nuciferine	SLC6A3

10146	Nuciferine	SLC6A4
10146	Nuciferine	SSTR1
10146	Nuciferine	SSTR4
10146	Nuciferine	TBXA2R
10146	Nuciferine	TH
10146	Nuciferine	THRA
10146	Nuciferine	THRB
10146	Nuciferine	TSPO
10146	Nuciferine	XIAP
4970	Fumarine	ADRA1A
4970	Fumarine	ADRA1B
4970	Fumarine	ADRA1D
4970	Fumarine	APP
4970	Fumarine	CCNE1
4970	Fumarine	CDC7
4970	Fumarine	CDK2
4970	Fumarine	CHEK1
4970	Fumarine	CHRM4
4970	Fumarine	CYP19A1
4970	Fumarine	DRD1
4970	Fumarine	DRD2
4970	Fumarine	DRD3
4970	Fumarine	DRD4
4970	Fumarine	DRD5
4970	Fumarine	DUSP3
4970	Fumarine	ERN1
4970	Fumarine	F3
4970	Fumarine	FLT3
4970	Fumarine	HTR1A
4970	Fumarine	HTR7
4970	Fumarine	JAK1
4970	Fumarine	JAK2
4970	Fumarine	JAK3
4970	Fumarine	LRRK2
4970	Fumarine	MAPK8
4970	Fumarine	MKNK2
4970	Fumarine	NEK1
4970	Fumarine	PARP2
4970	Fumarine	PDPK1
4970	Fumarine	PIK3CA
4970	Fumarine	PIK3CB
4970	Fumarine	PIK3CD
4970	Fumarine	PIM1
4970	Fumarine	PIM3

4970	Fumarine	SIGMAR1
4970	Fumarine	SIRT1
4970	Fumarine	SIRT2
4970	Fumarine	SIRT3
4970	Fumarine	SLC6A3
4970	Fumarine	SYK
4970	Fumarine	TRHR
4970	Fumarine	TTK
4970	Fumarine	TYMS
5280537	Moupinamide	ABL1
5280537	Moupinamide	ACHE
5280537	Moupinamide	ADAM10
5280537	Moupinamide	ADAM17
5280537	Moupinamide	ADORA1
5280537	Moupinamide	ADORA2A
5280537	Moupinamide	ADORA3
5280537	Moupinamide	AGTR1
5280537	Moupinamide	AKT2
5280537	Moupinamide	ALDH2
5280537	Moupinamide	ALK
5280537	Moupinamide	ALOX5
5280537	Moupinamide	ANPEP
5280537	Moupinamide	AVPR1A
5280537	Moupinamide	BCHE
5280537	Moupinamide	BMP1
5280537	Moupinamide	BRAF
5280537	Moupinamide	CA12
5280537	Moupinamide	CA13
5280537	Moupinamide	CA14
5280537	Moupinamide	CA5A
5280537	Moupinamide	CA5B
5280537	Moupinamide	CA6
5280537	Moupinamide	CA7
5280537	Moupinamide	CA9
5280537	Moupinamide	CCNE1
5280537	Moupinamide	CCNE1
5280537	Moupinamide	CDK1
5280537	Moupinamide	CDK1
5280537	Moupinamide	CDK4
5280537	Moupinamide	CDK5R1
5280537	Moupinamide	CFD
5280537	Moupinamide	CHEK1
5280537	Moupinamide	CHEK2
5280537	Moupinamide	CNR2

5280537	Moupinamide	CTSL
5280537	Moupinamide	DNM1
5280537	Moupinamide	DRD2
5280537	Moupinamide	DRD3
5280537	Moupinamide	DUSP3
5280537	Moupinamide	EGFR
5280537	Moupinamide	EPHA2
5280537	Moupinamide	EPHA4
5280537	Moupinamide	EPHA5
5280537	Moupinamide	EPHA7
5280537	Moupinamide	EPHA8
5280537	Moupinamide	EPHB2
5280537	Moupinamide	EPHB3
5280537	Moupinamide	ESRRA
5280537	Moupinamide	ESRRB
5280537	Moupinamide	GLI2
5280537	Moupinamide	GRK2
5280537	Moupinamide	HDAC1
5280537	Moupinamide	HPGDS
5280537	Moupinamide	HSD17B1
5280537	Moupinamide	HSD17B2
5280537	Moupinamide	HSP90AA1
5280537	Moupinamide	HSP90AB1
5280537	Moupinamide	HSP90B1
5280537	Moupinamide	HTR3A
5280537	Moupinamide	INSR
5280537	Moupinamide	MAOB
5280537	Moupinamide	MAP2K1
5280537	Moupinamide	MIF
5280537	Moupinamide	MMP1
5280537	Moupinamide	MMP12
5280537	Moupinamide	MMP13
5280537	Moupinamide	MMP14
5280537	Moupinamide	MMP16
5280537	Moupinamide	MMP2
5280537	Moupinamide	MMP3
5280537	Moupinamide	MMP7
5280537	Moupinamide	MMP8
5280537	Moupinamide	MMP9
5280537	Moupinamide	MTNR1A
5280537	Moupinamide	MTOR
5280537	Moupinamide	NR1H4
5280537	Moupinamide	PDE4C
5280537	Moupinamide	PDE4D

5280537	Moupinamide	PDE7A
5280537	Moupinamide	PDK1
5280537	Moupinamide	PNMT
5280537	Moupinamide	PRKCZ
5280537	Moupinamide	PTGS2
5280537	Moupinamide	PTPN1
5280537	Moupinamide	ROCK2
5280537	Moupinamide	RPS6KB1
5280537	Moupinamide	SLC5A1
5280537	Moupinamide	SYK
5280537	Moupinamide	THRA
5280537	Moupinamide	THRB
5280537	Moupinamide	TNF
5280537	Moupinamide	TOP1
5280537	Moupinamide	TRAP1
5280537	Moupinamide	TRPM8
5280537	Moupinamide	TSPO
5280537	Moupinamide	TYMS
5280537	Moupinamide	TYR
5280537	Moupinamide	VCP
5280537	Moupinamide	WEE1
5351516	Peroxyergosterol	AKR1C3
5351516	Peroxyergosterol	AVPR1A
5351516	Peroxyergosterol	BACE1
5351516	Peroxyergosterol	CNR1
5351516	Peroxyergosterol	CNR2
5351516	Peroxyergosterol	CTSD
5351516	Peroxyergosterol	CXCR3
5351516	Peroxyergosterol	CYP24A1
5351516	Peroxyergosterol	DHCR7
5351516	Peroxyergosterol	FAAH
5351516	Peroxyergosterol	FASN
5351516	Peroxyergosterol	GLRA1
5351516	Peroxyergosterol	HPGDS
5351516	Peroxyergosterol	HSD11B2
5351516	Peroxyergosterol	ITK
5351516	Peroxyergosterol	MDM2
5351516	Peroxyergosterol	MDM4
5351516	Peroxyergosterol	METAP1
5351516	Peroxyergosterol	MTNR1A
5351516	Peroxyergosterol	MTNR1B
5351516	Peroxyergosterol	NOS2
5351516	Peroxyergosterol	NR1H2
5351516	Peroxyergosterol	PDE4A

5351516	Peroxyergosterol	PDE4B
5351516	Peroxyergosterol	PDE4C
5351516	Peroxyergosterol	PDE4D
5351516	Peroxyergosterol	PFKFB3
5351516	Peroxyergosterol	S1PR1
5351516	Peroxyergosterol	S1PR3
5351516	Peroxyergosterol	SHH
5351516	Peroxyergosterol	TRPV1
160487	(S)-Coclaurine	ABCB1
160487	(S)-Coclaurine	ACHE
160487	(S)-Coclaurine	ADRA1A
160487	(S)-Coclaurine	ADRA1B
160487	(S)-Coclaurine	ADRA1D
160487	(S)-Coclaurine	ADRA2A
160487	(S)-Coclaurine	ADRA2B
160487	(S)-Coclaurine	ADRA2C
160487	(S)-Coclaurine	ADRB1
160487	(S)-Coclaurine	ADRB2
160487	(S)-Coclaurine	ADRB3
160487	(S)-Coclaurine	AKT1
160487	(S)-Coclaurine	BCHE
160487	(S)-Coclaurine	CA1
160487	(S)-Coclaurine	CA2
160487	(S)-Coclaurine	CA7
160487	(S)-Coclaurine	CA9
160487	(S)-Coclaurine	CHRM4
160487	(S)-Coclaurine	CHRNA3
160487	(S)-Coclaurine	CHRNA4
160487	(S)-Coclaurine	CHRNB4
160487	(S)-Coclaurine	DHCR7
160487	(S)-Coclaurine	DPP4
160487	(S)-Coclaurine	DRD1
160487	(S)-Coclaurine	DRD2
160487	(S)-Coclaurine	DRD3
160487	(S)-Coclaurine	DRD4
160487	(S)-Coclaurine	DRD5
160487	(S)-Coclaurine	ESR1
160487	(S)-Coclaurine	ESR2
160487	(S)-Coclaurine	F3
160487	(S)-Coclaurine	GRIK2
160487	(S)-Coclaurine	HCRTR1
160487	(S)-Coclaurine	HCRTR2
160487	(S)-Coclaurine	HDAC1
160487	(S)-Coclaurine	HTR1A

160487	(S)-Coclaurine	HTR2A
160487	(S)-Coclaurine	HTR2C
160487	(S)-Coclaurine	HTR7
160487	(S)-Coclaurine	JAK1
160487	(S)-Coclaurine	JAK2
160487	(S)-Coclaurine	JAK3
160487	(S)-Coclaurine	JUN
160487	(S)-Coclaurine	KCNH2
160487	(S)-Coclaurine	KCNN1
160487	(S)-Coclaurine	KCNN2
160487	(S)-Coclaurine	KCNN3
160487	(S)-Coclaurine	KDM1A
160487	(S)-Coclaurine	MAOA
160487	(S)-Coclaurine	MTNR1B
160487	(S)-Coclaurine	OPRD1
160487	(S)-Coclaurine	OPRK1
160487	(S)-Coclaurine	OPRM1
160487	(S)-Coclaurine	PARP1
160487	(S)-Coclaurine	PTPRCAP
160487	(S)-Coclaurine	RBBP9
160487	(S)-Coclaurine	ROCK2
160487	(S)-Coclaurine	SIGMAR1
160487	(S)-Coclaurine	SLC18A2
160487	(S)-Coclaurine	SLC47A1
160487	(S)-Coclaurine	SLC6A2
160487	(S)-Coclaurine	SLC6A3
160487	(S)-Coclaurine	SLC6A4
160487	(S)-Coclaurine	TBXA2R
160487	(S)-Coclaurine	TYK2
122691	Lysicamine	ABCG2
122691	Lysicamine	ACE
122691	Lysicamine	ACVR1B
122691	Lysicamine	ADORA2A
122691	Lysicamine	ADORA2B
122691	Lysicamine	AKR1C3
122691	Lysicamine	AKT1
122691	Lysicamine	ALDH1A1
122691	Lysicamine	ALPL
122691	Lysicamine	BDKRB2
122691	Lysicamine	BMP4
122691	Lysicamine	BTK
122691	Lysicamine	CA12
122691	Lysicamine	CA7
122691	Lysicamine	CA9

122691	Lysicamine	CACNA1B
122691	Lysicamine	CAPN1
122691	Lysicamine	CCNB3
122691	Lysicamine	CCNC
122691	Lysicamine	CCND3
122691	Lysicamine	CCR5
122691	Lysicamine	CCR8
122691	Lysicamine	CDC7
122691	Lysicamine	CDK1
122691	Lysicamine	CDK2
122691	Lysicamine	CDK7
122691	Lysicamine	CES1
122691	Lysicamine	CES2
122691	Lysicamine	CHRM1
122691	Lysicamine	CHRM2
122691	Lysicamine	CSNK1D
122691	Lysicamine	CTSK
122691	Lysicamine	CTSS
122691	Lysicamine	CYP17A1
122691	Lysicamine	CYP19A1
122691	Lysicamine	DUSP3
122691	Lysicamine	EGFR
122691	Lysicamine	ELANE
122691	Lysicamine	EPHX2
122691	Lysicamine	ERBB2
122691	Lysicamine	FAP
122691	Lysicamine	GABRA5
122691	Lysicamine	GABRB3
122691	Lysicamine	GAPDH
122691	Lysicamine	GPR139
122691	Lysicamine	GRM4
122691	Lysicamine	GSK3A
122691	Lysicamine	GSK3B
122691	Lysicamine	GSR
122691	Lysicamine	GUSB
122691	Lysicamine	HCRTR1
122691	Lysicamine	HCRTR2
122691	Lysicamine	HDAC3
122691	Lysicamine	HDAC6
122691	Lysicamine	HRH1
122691	Lysicamine	HRH4
122691	Lysicamine	HSD11B1
122691	Lysicamine	HTR4
122691	Lysicamine	ICAM1

122691	Lysicamine	LCK
122691	Lysicamine	MAP2K1
122691	Lysicamine	MAPK10
122691	Lysicamine	MAPK8
122691	Lysicamine	MAPK9
122691	Lysicamine	MET
122691	Lysicamine	MMP1
122691	Lysicamine	MMP13
122691	Lysicamine	MMP8
122691	Lysicamine	NAMPT
122691	Lysicamine	NOTUM
122691	Lysicamine	NQO2
122691	Lysicamine	P2RX7
122691	Lysicamine	PABPC1
122691	Lysicamine	PARP2
122691	Lysicamine	PDE7A
122691	Lysicamine	PGGT1B
122691	Lysicamine	PIK3CA
122691	Lysicamine	PREP
122691	Lysicamine	PRKDC
122691	Lysicamine	PTGER2
122691	Lysicamine	PTGER4
122691	Lysicamine	PTP4A3
122691	Lysicamine	RPS6KA5
122691	Lysicamine	SELE
122691	Lysicamine	SHH
122691	Lysicamine	SIGMAR1
122691	Lysicamine	SIRT2
122691	Lysicamine	SLC22A12
122691	Lysicamine	SRC
122691	Lysicamine	STAT3
122691	Lysicamine	TAAR1
122691	Lysicamine	TGFBR1
122691	Lysicamine	TGM2
122691	Lysicamine	TLR9
122691	Lysicamine	TNFRSF1A
122691	Lysicamine	TNKS
122691	Lysicamine	TNKS2
122691	Lysicamine	TRPA1
122691	Lysicamine	TRPV1
122691	Lysicamine	UTS2R
73299	hederagenin	ACP1
73299	hederagenin	AKR1B10
73299	hederagenin	ALOX5

73299	hederagenin	ALOX5AP
73299	hederagenin	AR
73299	hederagenin	BACE1
73299	hederagenin	BCHE
73299	hederagenin	CD81
73299	hederagenin	CDC25A
73299	hederagenin	CDC25B
73299	hederagenin	CES2
73299	hederagenin	CHRM2
73299	hederagenin	CYP17A1
73299	hederagenin	CYP19A1
73299	hederagenin	CYP51A1
73299	hederagenin	ESR1
73299	hederagenin	ESR2
73299	hederagenin	FAAH
73299	hederagenin	FABP1
73299	hederagenin	FABP3
73299	hederagenin	FABP4
73299	hederagenin	FABP5
73299	hederagenin	FFAR1
73299	hederagenin	FNTA
73299	hederagenin	G6PD
73299	hederagenin	GLUL
73299	hederagenin	GPBAR1
73299	hederagenin	GRIK1
73299	hederagenin	GRIK2
73299	hederagenin	HMGCR
73299	hederagenin	HSD11B1
73299	hederagenin	HSD11B2
73299	hederagenin	IL6
73299	hederagenin	LTB4R
73299	hederagenin	NOS2
73299	hederagenin	NR1H3
73299	hederagenin	NR1H4
73299	hederagenin	NR3C1
73299	hederagenin	NR3C2
73299	hederagenin	PDE4D
73299	hederagenin	PGR
73299	hederagenin	PLA2G1B
73299	hederagenin	POLB
73299	hederagenin	PPARA
73299	hederagenin	PPARD
73299	hederagenin	PPARG
73299	hederagenin	PREP

73299	hederagenin	PRKCH
73299	hederagenin	PTGDR
73299	hederagenin	PTGDR2
73299	hederagenin	PTGER1
73299	hederagenin	PTGER2
73299	hederagenin	PTGER4
73299	hederagenin	PTGES
73299	hederagenin	PTGIR
73299	hederagenin	PTGS1
73299	hederagenin	PTGS2
73299	hederagenin	PTPN1
73299	hederagenin	PTPN11
73299	hederagenin	PTPN2
73299	hederagenin	PTPN6
73299	hederagenin	PTPRF
73299	hederagenin	RORA
73299	hederagenin	RORC
73299	hederagenin	SCD
73299	hederagenin	SERPINA6
73299	hederagenin	SHBG
73299	hederagenin	SIGMAR1
73299	hederagenin	SLC10A1
73299	hederagenin	SLC10A2
73299	hederagenin	SRD5A2
73299	hederagenin	TERT
73299	hederagenin	TLR9
73299	hederagenin	TOP1
73299	hederagenin	TOP2A
10181133	Cerevisterol	CYP2D6
10181133	Cerevisterol	ACHE
10181133	Cerevisterol	ADORA1
10181133	Cerevisterol	ADORA2A
10181133	Cerevisterol	AKR1C3
10181133	Cerevisterol	ALK
10181133	Cerevisterol	AR
10181133	Cerevisterol	AVPR1A
10181133	Cerevisterol	BCHE
10181133	Cerevisterol	CA4
10181133	Cerevisterol	CA7
10181133	Cerevisterol	CALCRL
10181133	Cerevisterol	CCND1
10181133	Cerevisterol	CCNE2
10181133	Cerevisterol	CCNT1
10181133	Cerevisterol	CCR1

10181133	Cerevisterol	CDK2
10181133	Cerevisterol	CDK4
10181133	Cerevisterol	CDK6
10181133	Cerevisterol	CDK9
10181133	Cerevisterol	CHRM2
10181133	Cerevisterol	CHUK
10181133	Cerevisterol	CNR2
10181133	Cerevisterol	CSF1R
10181133	Cerevisterol	CTSL
10181133	Cerevisterol	CYP17A1
10181133	Cerevisterol	CYP19A1
10181133	Cerevisterol	CYP2C19
10181133	Cerevisterol	CYP2C9
10181133	Cerevisterol	CYP3A4
10181133	Cerevisterol	CYP51A1
10181133	Cerevisterol	DGAT1
10181133	Cerevisterol	EGFR
10181133	Cerevisterol	ESR1
10181133	Cerevisterol	ESR2
10181133	Cerevisterol	FGFR2
10181133	Cerevisterol	GPR18
10181133	Cerevisterol	GPR55
10181133	Cerevisterol	GRM2
10181133	Cerevisterol	GSK3B
10181133	Cerevisterol	HCRTR1
10181133	Cerevisterol	HCRTR2
10181133	Cerevisterol	HMGCR
10181133	Cerevisterol	HSD11B1
10181133	Cerevisterol	IKBKB
10181133	Cerevisterol	IL6ST
10181133	Cerevisterol	KDR
10181133	Cerevisterol	KIT
10181133	Cerevisterol	MAPK14
10181133	Cerevisterol	MAPK8
10181133	Cerevisterol	MAST3
10181133	Cerevisterol	MDM2
10181133	Cerevisterol	MDM4
10181133	Cerevisterol	MET
10181133	Cerevisterol	METAP1
10181133	Cerevisterol	MGLL
10181133	Cerevisterol	MTNR1A
10181133	Cerevisterol	MTNR1B
10181133	Cerevisterol	MTOR
10181133	Cerevisterol	NPC1L1

10181133	Cerevisterol	NR1H2
10181133	Cerevisterol	NR1H3
10181133	Cerevisterol	NR1I2
10181133	Cerevisterol	NTRK1
10181133	Cerevisterol	OPRD1
10181133	Cerevisterol	OPRK1
10181133	Cerevisterol	OPRL1
10181133	Cerevisterol	OPRM1
10181133	Cerevisterol	PDE2A
10181133	Cerevisterol	PDE3A
10181133	Cerevisterol	PDE3B
10181133	Cerevisterol	PDE4B
10181133	Cerevisterol	PDGFRB
10181133	Cerevisterol	PFKFB3
10181133	Cerevisterol	PIM1
10181133	Cerevisterol	PIM3
10181133	Cerevisterol	PRKCA
10181133	Cerevisterol	PRKCB
10181133	Cerevisterol	PRKCE
10181133	Cerevisterol	PSEN2
10181133	Cerevisterol	PTGDR
10181133	Cerevisterol	PTGER1
10181133	Cerevisterol	PTGER3
10181133	Cerevisterol	PTGFR
10181133	Cerevisterol	PTPN1
10181133	Cerevisterol	PYGL
10181133	Cerevisterol	RORA
10181133	Cerevisterol	RORC
10181133	Cerevisterol	S1PR1
10181133	Cerevisterol	S1PR3
10181133	Cerevisterol	SERPINA6
10181133	Cerevisterol	SHBG
10181133	Cerevisterol	SLC6A2
10181133	Cerevisterol	SLC6A4
10181133	Cerevisterol	SMO
10181133	Cerevisterol	SRC
10181133	Cerevisterol	SREBF2
10181133	Cerevisterol	TNF
10181133	Cerevisterol	TRPV1
10181133	Cerevisterol	VDR
10743008	(2R)-2-[(3S,5R,10S,13R,14R,16R,17R)-3,16-dihydroxy-4,4,10,13,14-pentamethyl-	ACP1

	2,3,5,6,12,15,16,17-octahydro-1H-cyclopenta[a]phenanthren-17-yl]-6-methylhept-5-enoic acid	
10743008	(2R)-2-[(3S,5R,10S,13R,14R,16R,17R)-3,16-dihydroxy-4,4,10,13,14-pentamethyl-2,3,5,6,12,15,16,17-octahydro-1H-cyclopenta[a]phenanthren-17-yl]-6-methylhept-5-enoic acid	AGTR1
10743008	(2R)-2-[(3S,5R,10S,13R,14R,16R,17R)-3,16-dihydroxy-4,4,10,13,14-pentamethyl-2,3,5,6,12,15,16,17-octahydro-1H-cyclopenta[a]phenanthren-17-yl]-6-methylhept-5-enoic acid	AKR1B10
10743008	(2R)-2-[(3S,5R,10S,13R,14R,16R,17R)-3,16-dihydroxy-4,4,10,13,14-pentamethyl-2,3,5,6,12,15,16,17-octahydro-1H-cyclopenta[a]phenanthren-17-yl]-6-methylhept-5-enoic acid	ALOX12
10743008	(2R)-2-[(3S,5R,10S,13R,14R,16R,17R)-3,16-dihydroxy-4,4,10,13,14-pentamethyl-2,3,5,6,12,15,16,17-octahydro-1H-cyclopenta[a]phenanthren-17-yl]-6-methylhept-5-enoic acid	ALOX5AP
10743008	(2R)-2-[(3S,5R,10S,13R,14R,16R,17R)-3,16-dihydroxy-4,4,10,13,14-pentamethyl-2,3,5,6,12,15,16,17-octahydro-1H-cyclopenta[a]phenanthren-17-yl]-6-methylhept-5-enoic acid	AR

10743008	(2R)-2-[(3S,5R,10S,13R,14R,16R,17R)-3,16-dihydroxy-4,4,10,13,14-pentamethyl-2,3,5,6,12,15,16,17-octahydro-1H-cyclopenta[a]phenanthren-17-yl]-6-methylhept-5-enoic acid	BACE1
10743008	(2R)-2-[(3S,5R,10S,13R,14R,16R,17R)-3,16-dihydroxy-4,4,10,13,14-pentamethyl-2,3,5,6,12,15,16,17-octahydro-1H-cyclopenta[a]phenanthren-17-yl]-6-methylhept-5-enoic acid	BCHE
10743008	(2R)-2-[(3S,5R,10S,13R,14R,16R,17R)-3,16-dihydroxy-4,4,10,13,14-pentamethyl-2,3,5,6,12,15,16,17-octahydro-1H-cyclopenta[a]phenanthren-17-yl]-6-methylhept-5-enoic acid	CD81
10743008	(2R)-2-[(3S,5R,10S,13R,14R,16R,17R)-3,16-dihydroxy-4,4,10,13,14-pentamethyl-2,3,5,6,12,15,16,17-octahydro-1H-cyclopenta[a]phenanthren-17-yl]-6-methylhept-5-enoic acid	CDC25B
10743008	(2R)-2-[(3S,5R,10S,13R,14R,16R,17R)-3,16-dihydroxy-4,4,10,13,14-pentamethyl-2,3,5,6,12,15,16,17-octahydro-1H-cyclopenta[a]phenanthren-17-yl]-6-methylhept-5-enoic acid	CES2
10743008	(2R)-2-[(3S,5R,10S,13R,14R,16R,17R)-3,16-dihydroxy-4,4,10,13,14-pentamethyl-	CTSA

	2,3,5,6,12,15,16,17-octahydro-1H-cyclopenta[a]phenanthren-17-yl]-6-methylhept-5-enoic acid	
10743008	(2R)-2-[(3S,5R,10S,13R,14R,16R,17R)-3,16-dihydroxy-4,4,10,13,14-pentamethyl-2,3,5,6,12,15,16,17-octahydro-1H-cyclopenta[a]phenanthren-17-yl]-6-methylhept-5-enoic acid	CYP17A1
10743008	(2R)-2-[(3S,5R,10S,13R,14R,16R,17R)-3,16-dihydroxy-4,4,10,13,14-pentamethyl-2,3,5,6,12,15,16,17-octahydro-1H-cyclopenta[a]phenanthren-17-yl]-6-methylhept-5-enoic acid	CYP19A1
10743008	(2R)-2-[(3S,5R,10S,13R,14R,16R,17R)-3,16-dihydroxy-4,4,10,13,14-pentamethyl-2,3,5,6,12,15,16,17-octahydro-1H-cyclopenta[a]phenanthren-17-yl]-6-methylhept-5-enoic acid	CYP51A1
10743008	(2R)-2-[(3S,5R,10S,13R,14R,16R,17R)-3,16-dihydroxy-4,4,10,13,14-pentamethyl-2,3,5,6,12,15,16,17-octahydro-1H-cyclopenta[a]phenanthren-17-yl]-6-methylhept-5-enoic acid	CYSLTR1
10743008	(2R)-2-[(3S,5R,10S,13R,14R,16R,17R)-3,16-dihydroxy-4,4,10,13,14-pentamethyl-2,3,5,6,12,15,16,17-octahydro-1H-cyclopenta[a]phenanthren-17-yl]-6-methylhept-5-enoic acid	EDNRA

10743008	(2R)-2-[(3S,5R,10S,13R,14R,16R,17R)-3,16-dihydroxy-4,4,10,13,14-pentamethyl-2,3,5,6,12,15,16,17-octahydro-1H-cyclopenta[a]phenanthren-17-yl]-6-methylhept-5-enoic acid	ESR1
10743008	(2R)-2-[(3S,5R,10S,13R,14R,16R,17R)-3,16-dihydroxy-4,4,10,13,14-pentamethyl-2,3,5,6,12,15,16,17-octahydro-1H-cyclopenta[a]phenanthren-17-yl]-6-methylhept-5-enoic acid	ESR2
10743008	(2R)-2-[(3S,5R,10S,13R,14R,16R,17R)-3,16-dihydroxy-4,4,10,13,14-pentamethyl-2,3,5,6,12,15,16,17-octahydro-1H-cyclopenta[a]phenanthren-17-yl]-6-methylhept-5-enoic acid	FABP1
10743008	(2R)-2-[(3S,5R,10S,13R,14R,16R,17R)-3,16-dihydroxy-4,4,10,13,14-pentamethyl-2,3,5,6,12,15,16,17-octahydro-1H-cyclopenta[a]phenanthren-17-yl]-6-methylhept-5-enoic acid	FNTA
10743008	(2R)-2-[(3S,5R,10S,13R,14R,16R,17R)-3,16-dihydroxy-4,4,10,13,14-pentamethyl-2,3,5,6,12,15,16,17-octahydro-1H-cyclopenta[a]phenanthren-17-yl]-6-methylhept-5-enoic acid	G6PD
10743008	(2R)-2-[(3S,5R,10S,13R,14R,16R,17R)-3,16-dihydroxy-4,4,10,13,14-pentamethyl-	HAO1

	2,3,5,6,12,15,16,17-octahydro-1H-cyclopenta[a]phenanthren-17-yl]-6-methylhept-5-enoic acid	
10743008	(2R)-2-[(3S,5R,10S,13R,14R,16R,17R)-3,16-dihydroxy-4,4,10,13,14-pentamethyl-2,3,5,6,12,15,16,17-octahydro-1H-cyclopenta[a]phenanthren-17-yl]-6-methylhept-5-enoic acid	HMGCR
10743008	(2R)-2-[(3S,5R,10S,13R,14R,16R,17R)-3,16-dihydroxy-4,4,10,13,14-pentamethyl-2,3,5,6,12,15,16,17-octahydro-1H-cyclopenta[a]phenanthren-17-yl]-6-methylhept-5-enoic acid	HSD11B1
10743008	(2R)-2-[(3S,5R,10S,13R,14R,16R,17R)-3,16-dihydroxy-4,4,10,13,14-pentamethyl-2,3,5,6,12,15,16,17-octahydro-1H-cyclopenta[a]phenanthren-17-yl]-6-methylhept-5-enoic acid	HSD11B2
10743008	(2R)-2-[(3S,5R,10S,13R,14R,16R,17R)-3,16-dihydroxy-4,4,10,13,14-pentamethyl-2,3,5,6,12,15,16,17-octahydro-1H-cyclopenta[a]phenanthren-17-yl]-6-methylhept-5-enoic acid	ITGAV
10743008	(2R)-2-[(3S,5R,10S,13R,14R,16R,17R)-3,16-dihydroxy-4,4,10,13,14-pentamethyl-2,3,5,6,12,15,16,17-octahydro-1H-cyclopenta[a]phenanthren-17-yl]-6-methylhept-5-enoic acid	MDM2

10743008	(2R)-2-[(3S,5R,10S,13R,14R,16R,17R)-3,16-dihydroxy-4,4,10,13,14-pentamethyl-2,3,5,6,12,15,16,17-octahydro-1H-cyclopenta[a]phenanthren-17-yl]-6-methylhept-5-enoic acid	MMP1
10743008	(2R)-2-[(3S,5R,10S,13R,14R,16R,17R)-3,16-dihydroxy-4,4,10,13,14-pentamethyl-2,3,5,6,12,15,16,17-octahydro-1H-cyclopenta[a]phenanthren-17-yl]-6-methylhept-5-enoic acid	MMP10
10743008	(2R)-2-[(3S,5R,10S,13R,14R,16R,17R)-3,16-dihydroxy-4,4,10,13,14-pentamethyl-2,3,5,6,12,15,16,17-octahydro-1H-cyclopenta[a]phenanthren-17-yl]-6-methylhept-5-enoic acid	MMP12
10743008	(2R)-2-[(3S,5R,10S,13R,14R,16R,17R)-3,16-dihydroxy-4,4,10,13,14-pentamethyl-2,3,5,6,12,15,16,17-octahydro-1H-cyclopenta[a]phenanthren-17-yl]-6-methylhept-5-enoic acid	MMP13
10743008	(2R)-2-[(3S,5R,10S,13R,14R,16R,17R)-3,16-dihydroxy-4,4,10,13,14-pentamethyl-2,3,5,6,12,15,16,17-octahydro-1H-cyclopenta[a]phenanthren-17-yl]-6-methylhept-5-enoic acid	MMP2
10743008	(2R)-2-[(3S,5R,10S,13R,14R,16R,17R)-3,16-dihydroxy-4,4,10,13,14-pentamethyl-	MMP3

	2,3,5,6,12,15,16,17-octahydro-1H-cyclopenta[a]phenanthren-17-yl]-6-methylhept-5-enoic acid	
10743008	(2R)-2-[(3S,5R,10S,13R,14R,16R,17R)-3,16-dihydroxy-4,4,10,13,14-pentamethyl-2,3,5,6,12,15,16,17-octahydro-1H-cyclopenta[a]phenanthren-17-yl]-6-methylhept-5-enoic acid	MMP8
10743008	(2R)-2-[(3S,5R,10S,13R,14R,16R,17R)-3,16-dihydroxy-4,4,10,13,14-pentamethyl-2,3,5,6,12,15,16,17-octahydro-1H-cyclopenta[a]phenanthren-17-yl]-6-methylhept-5-enoic acid	MMP9
10743008	(2R)-2-[(3S,5R,10S,13R,14R,16R,17R)-3,16-dihydroxy-4,4,10,13,14-pentamethyl-2,3,5,6,12,15,16,17-octahydro-1H-cyclopenta[a]phenanthren-17-yl]-6-methylhept-5-enoic acid	NOS2
10743008	(2R)-2-[(3S,5R,10S,13R,14R,16R,17R)-3,16-dihydroxy-4,4,10,13,14-pentamethyl-2,3,5,6,12,15,16,17-octahydro-1H-cyclopenta[a]phenanthren-17-yl]-6-methylhept-5-enoic acid	NPC1L1
10743008	(2R)-2-[(3S,5R,10S,13R,14R,16R,17R)-3,16-dihydroxy-4,4,10,13,14-pentamethyl-2,3,5,6,12,15,16,17-octahydro-1H-cyclopenta[a]phenanthren-17-yl]-6-methylhept-5-enoic acid	NR1H3

10743008	(2R)-2-[(3S,5R,10S,13R,14R,16R,17R)-3,16-dihydroxy-4,4,10,13,14-pentamethyl-2,3,5,6,12,15,16,17-octahydro-1H-cyclopenta[a]phenanthren-17-yl]-6-methylhept-5-enoic acid	NR3C1
10743008	(2R)-2-[(3S,5R,10S,13R,14R,16R,17R)-3,16-dihydroxy-4,4,10,13,14-pentamethyl-2,3,5,6,12,15,16,17-octahydro-1H-cyclopenta[a]phenanthren-17-yl]-6-methylhept-5-enoic acid	OPRD1
10743008	(2R)-2-[(3S,5R,10S,13R,14R,16R,17R)-3,16-dihydroxy-4,4,10,13,14-pentamethyl-2,3,5,6,12,15,16,17-octahydro-1H-cyclopenta[a]phenanthren-17-yl]-6-methylhept-5-enoic acid	OPRK1
10743008	(2R)-2-[(3S,5R,10S,13R,14R,16R,17R)-3,16-dihydroxy-4,4,10,13,14-pentamethyl-2,3,5,6,12,15,16,17-octahydro-1H-cyclopenta[a]phenanthren-17-yl]-6-methylhept-5-enoic acid	PDE4D
10743008	(2R)-2-[(3S,5R,10S,13R,14R,16R,17R)-3,16-dihydroxy-4,4,10,13,14-pentamethyl-2,3,5,6,12,15,16,17-octahydro-1H-cyclopenta[a]phenanthren-17-yl]-6-methylhept-5-enoic acid	PGR
10743008	(2R)-2-[(3S,5R,10S,13R,14R,16R,17R)-3,16-dihydroxy-4,4,10,13,14-pentamethyl-	PLA2G1B

	2,3,5,6,12,15,16,17-octahydro-1H-cyclopenta[a]phenanthren-17-yl]-6-methylhept-5-enoic acid	
10743008	(2R)-2-[(3S,5R,10S,13R,14R,16R,17R)-3,16-dihydroxy-4,4,10,13,14-pentamethyl-2,3,5,6,12,15,16,17-octahydro-1H-cyclopenta[a]phenanthren-17-yl]-6-methylhept-5-enoic acid	POLB
10743008	(2R)-2-[(3S,5R,10S,13R,14R,16R,17R)-3,16-dihydroxy-4,4,10,13,14-pentamethyl-2,3,5,6,12,15,16,17-octahydro-1H-cyclopenta[a]phenanthren-17-yl]-6-methylhept-5-enoic acid	PPARA
10743008	(2R)-2-[(3S,5R,10S,13R,14R,16R,17R)-3,16-dihydroxy-4,4,10,13,14-pentamethyl-2,3,5,6,12,15,16,17-octahydro-1H-cyclopenta[a]phenanthren-17-yl]-6-methylhept-5-enoic acid	PPARD
10743008	(2R)-2-[(3S,5R,10S,13R,14R,16R,17R)-3,16-dihydroxy-4,4,10,13,14-pentamethyl-2,3,5,6,12,15,16,17-octahydro-1H-cyclopenta[a]phenanthren-17-yl]-6-methylhept-5-enoic acid	PPARG
10743008	(2R)-2-[(3S,5R,10S,13R,14R,16R,17R)-3,16-dihydroxy-4,4,10,13,14-pentamethyl-2,3,5,6,12,15,16,17-octahydro-1H-cyclopenta[a]phenanthren-17-yl]-6-methylhept-5-enoic acid	PREP

10743008	(2R)-2-[(3S,5R,10S,13R,14R,16R,17R)-3,16-dihydroxy-4,4,10,13,14-pentamethyl-2,3,5,6,12,15,16,17-octahydro-1H-cyclopenta[a]phenanthren-17-yl]-6-methylhept-5-enoic acid	PRKCH
10743008	(2R)-2-[(3S,5R,10S,13R,14R,16R,17R)-3,16-dihydroxy-4,4,10,13,14-pentamethyl-2,3,5,6,12,15,16,17-octahydro-1H-cyclopenta[a]phenanthren-17-yl]-6-methylhept-5-enoic acid	PTGDR
10743008	(2R)-2-[(3S,5R,10S,13R,14R,16R,17R)-3,16-dihydroxy-4,4,10,13,14-pentamethyl-2,3,5,6,12,15,16,17-octahydro-1H-cyclopenta[a]phenanthren-17-yl]-6-methylhept-5-enoic acid	PTGDR2
10743008	(2R)-2-[(3S,5R,10S,13R,14R,16R,17R)-3,16-dihydroxy-4,4,10,13,14-pentamethyl-2,3,5,6,12,15,16,17-octahydro-1H-cyclopenta[a]phenanthren-17-yl]-6-methylhept-5-enoic acid	PTGER1
10743008	(2R)-2-[(3S,5R,10S,13R,14R,16R,17R)-3,16-dihydroxy-4,4,10,13,14-pentamethyl-2,3,5,6,12,15,16,17-octahydro-1H-cyclopenta[a]phenanthren-17-yl]-6-methylhept-5-enoic acid	PTGER2
10743008	(2R)-2-[(3S,5R,10S,13R,14R,16R,17R)-3,16-dihydroxy-4,4,10,13,14-pentamethyl-	PTGER3

	2,3,5,6,12,15,16,17-octahydro-1H-cyclopenta[a]phenanthren-17-yl]-6-methylhept-5-enoic acid	
10743008	(2R)-2-[(3S,5R,10S,13R,14R,16R,17R)-3,16-dihydroxy-4,4,10,13,14-pentamethyl-2,3,5,6,12,15,16,17-octahydro-1H-cyclopenta[a]phenanthren-17-yl]-6-methylhept-5-enoic acid	PTGER4
10743008	(2R)-2-[(3S,5R,10S,13R,14R,16R,17R)-3,16-dihydroxy-4,4,10,13,14-pentamethyl-2,3,5,6,12,15,16,17-octahydro-1H-cyclopenta[a]phenanthren-17-yl]-6-methylhept-5-enoic acid	PTGES
10743008	(2R)-2-[(3S,5R,10S,13R,14R,16R,17R)-3,16-dihydroxy-4,4,10,13,14-pentamethyl-2,3,5,6,12,15,16,17-octahydro-1H-cyclopenta[a]phenanthren-17-yl]-6-methylhept-5-enoic acid	PTGFR
10743008	(2R)-2-[(3S,5R,10S,13R,14R,16R,17R)-3,16-dihydroxy-4,4,10,13,14-pentamethyl-2,3,5,6,12,15,16,17-octahydro-1H-cyclopenta[a]phenanthren-17-yl]-6-methylhept-5-enoic acid	PTPN1
10743008	(2R)-2-[(3S,5R,10S,13R,14R,16R,17R)-3,16-dihydroxy-4,4,10,13,14-pentamethyl-2,3,5,6,12,15,16,17-octahydro-1H-cyclopenta[a]phenanthren-17-yl]-6-methylhept-5-enoic acid	PTPN11

10743008	(2R)-2-[(3S,5R,10S,13R,14R,16R,17R)-3,16-dihydroxy-4,4,10,13,14-pentamethyl-2,3,5,6,12,15,16,17-octahydro-1H-cyclopenta[a]phenanthren-17-yl]-6-methylhept-5-enoic acid	PTPN2
10743008	(2R)-2-[(3S,5R,10S,13R,14R,16R,17R)-3,16-dihydroxy-4,4,10,13,14-pentamethyl-2,3,5,6,12,15,16,17-octahydro-1H-cyclopenta[a]phenanthren-17-yl]-6-methylhept-5-enoic acid	PTPRF
10743008	(2R)-2-[(3S,5R,10S,13R,14R,16R,17R)-3,16-dihydroxy-4,4,10,13,14-pentamethyl-2,3,5,6,12,15,16,17-octahydro-1H-cyclopenta[a]phenanthren-17-yl]-6-methylhept-5-enoic acid	RASGRP3
10743008	(2R)-2-[(3S,5R,10S,13R,14R,16R,17R)-3,16-dihydroxy-4,4,10,13,14-pentamethyl-2,3,5,6,12,15,16,17-octahydro-1H-cyclopenta[a]phenanthren-17-yl]-6-methylhept-5-enoic acid	RORC
10743008	(2R)-2-[(3S,5R,10S,13R,14R,16R,17R)-3,16-dihydroxy-4,4,10,13,14-pentamethyl-2,3,5,6,12,15,16,17-octahydro-1H-cyclopenta[a]phenanthren-17-yl]-6-methylhept-5-enoic acid	SERPINA6
10743008	(2R)-2-[(3S,5R,10S,13R,14R,16R,17R)-3,16-dihydroxy-4,4,10,13,14-pentamethyl-	SHBG

	2,3,5,6,12,15,16,17-octahydro-1H-cyclopenta[a]phenanthren-17-yl]-6-methylhept-5-enoic acid	
10743008	(2R)-2-[(3S,5R,10S,13R,14R,16R,17R)-3,16-dihydroxy-4,4,10,13,14-pentamethyl-2,3,5,6,12,15,16,17-octahydro-1H-cyclopenta[a]phenanthren-17-yl]-6-methylhept-5-enoic acid	SIGMAR1
10743008	(2R)-2-[(3S,5R,10S,13R,14R,16R,17R)-3,16-dihydroxy-4,4,10,13,14-pentamethyl-2,3,5,6,12,15,16,17-octahydro-1H-cyclopenta[a]phenanthren-17-yl]-6-methylhept-5-enoic acid	SLC10A1
10743008	(2R)-2-[(3S,5R,10S,13R,14R,16R,17R)-3,16-dihydroxy-4,4,10,13,14-pentamethyl-2,3,5,6,12,15,16,17-octahydro-1H-cyclopenta[a]phenanthren-17-yl]-6-methylhept-5-enoic acid	SLC10A2
10743008	(2R)-2-[(3S,5R,10S,13R,14R,16R,17R)-3,16-dihydroxy-4,4,10,13,14-pentamethyl-2,3,5,6,12,15,16,17-octahydro-1H-cyclopenta[a]phenanthren-17-yl]-6-methylhept-5-enoic acid	THRA
10743008	(2R)-2-[(3S,5R,10S,13R,14R,16R,17R)-3,16-dihydroxy-4,4,10,13,14-pentamethyl-2,3,5,6,12,15,16,17-octahydro-1H-cyclopenta[a]phenanthren-17-yl]-6-methylhept-5-enoic acid	THRB

10743008	(2R)-2-[(3S,5R,10S,13R,14R,16R,17R)-3,16-dihydroxy-4,4,10,13,14-pentamethyl-2,3,5,6,12,15,16,17-octahydro-1H-cyclopenta[a]phenanthren-17-yl]-6-methylhept-5-enoic acid	TNF
10743008	(2R)-2-[(3S,5R,10S,13R,14R,16R,17R)-3,16-dihydroxy-4,4,10,13,14-pentamethyl-2,3,5,6,12,15,16,17-octahydro-1H-cyclopenta[a]phenanthren-17-yl]-6-methylhept-5-enoic acid	TOP2A
10743008	(2R)-2-[(3S,5R,10S,13R,14R,16R,17R)-3,16-dihydroxy-4,4,10,13,14-pentamethyl-2,3,5,6,12,15,16,17-octahydro-1H-cyclopenta[a]phenanthren-17-yl]-6-methylhept-5-enoic acid	TRPM8
10743008	(2R)-2-[(3S,5R,10S,13R,14R,16R,17R)-3,16-dihydroxy-4,4,10,13,14-pentamethyl-2,3,5,6,12,15,16,17-octahydro-1H-cyclopenta[a]phenanthren-17-yl]-6-methylhept-5-enoic acid	VDR
3893	Lauric Acid	AKR1A1
3893	Lauric Acid	AKR1B1
3893	Lauric Acid	AKR1B10
3893	Lauric Acid	AKR1C3
3893	Lauric Acid	AR
3893	Lauric Acid	BCL2
3893	Lauric Acid	BMP1
3893	Lauric Acid	CA1
3893	Lauric Acid	CA12
3893	Lauric Acid	CA2
3893	Lauric Acid	CACNA2D1
3893	Lauric Acid	CDC25A

3893	Lauric Acid	CDC45
3893	Lauric Acid	CHRNA7
3893	Lauric Acid	CMA1
3893	Lauric Acid	CTSG
3893	Lauric Acid	CXCL8
3893	Lauric Acid	CYP19A1
3893	Lauric Acid	CYP1A2
3893	Lauric Acid	EDNRA
3893	Lauric Acid	FABP1
3893	Lauric Acid	FABP2
3893	Lauric Acid	FABP3
3893	Lauric Acid	FABP4
3893	Lauric Acid	FABP5
3893	Lauric Acid	FFAR1
3893	Lauric Acid	FFAR4
3893	Lauric Acid	FNTA
3893	Lauric Acid	G6PD
3893	Lauric Acid	GABBR1
3893	Lauric Acid	GABRA2
3893	Lauric Acid	GPBAR1
3893	Lauric Acid	GRM5
3893	Lauric Acid	GSR
3893	Lauric Acid	GSTK1
3893	Lauric Acid	HAO1
3893	Lauric Acid	HMGCR
3893	Lauric Acid	HSD11B1
3893	Lauric Acid	HSD11B2
3893	Lauric Acid	HSD17B3
3893	Lauric Acid	IMPDH2
3893	Lauric Acid	KDM2A
3893	Lauric Acid	KDM5C
3893	Lauric Acid	LTA4H
3893	Lauric Acid	MAPK1
3893	Lauric Acid	MCL1
3893	Lauric Acid	MDM2
3893	Lauric Acid	MMP12
3893	Lauric Acid	MMP13
3893	Lauric Acid	MMP14
3893	Lauric Acid	MMP2
3893	Lauric Acid	MMP9
3893	Lauric Acid	NPC1L1
3893	Lauric Acid	NR1H4
3893	Lauric Acid	PHF8
3893	Lauric Acid	PLG

3893	Lauric Acid	POLB
3893	Lauric Acid	PPARA
3893	Lauric Acid	PPARD
3893	Lauric Acid	PTGDR
3893	Lauric Acid	PTGDR2
3893	Lauric Acid	PTGER2
3893	Lauric Acid	PTGER3
3893	Lauric Acid	PTGER4
3893	Lauric Acid	PTGES
3893	Lauric Acid	PTGFR
3893	Lauric Acid	PTPN1
3893	Lauric Acid	PTPRC
3893	Lauric Acid	RARA
3893	Lauric Acid	RARB
3893	Lauric Acid	RARG
3893	Lauric Acid	RBP4
3893	Lauric Acid	RXRA
3893	Lauric Acid	SAE1
3893	Lauric Acid	SERPINA6
3893	Lauric Acid	SHBG
3893	Lauric Acid	SLC16A1
3893	Lauric Acid	SLC22A12
3893	Lauric Acid	SLC22A6
3893	Lauric Acid	SLC6A4
3893	Lauric Acid	STS
3893	Lauric Acid	TBXA2R
3893	Lauric Acid	THRA
3893	Lauric Acid	THRB
3893	Lauric Acid	TRPA1
3893	Lauric Acid	TTR
3893	Lauric Acid	UGT2B7
3893	Lauric Acid	VDR
379	Caprylic Acid	AKR1B1
379	Caprylic Acid	AKR1B10
379	Caprylic Acid	AR
379	Caprylic Acid	CA1
379	Caprylic Acid	CA2
379	Caprylic Acid	CACNA2D1
379	Caprylic Acid	CDC25A
379	Caprylic Acid	CDC45
379	Caprylic Acid	CYP19A1
379	Caprylic Acid	FABP2
379	Caprylic Acid	FABP3
379	Caprylic Acid	FABP4

379	Caprylic Acid	FABP5
379	Caprylic Acid	FDFT1
379	Caprylic Acid	FFAR1
379	Caprylic Acid	FNTA
379	Caprylic Acid	G6PD
379	Caprylic Acid	GABBR1
379	Caprylic Acid	GABRA2
379	Caprylic Acid	GPBAR1
379	Caprylic Acid	GRM5
379	Caprylic Acid	GSTK1
379	Caprylic Acid	HAO1
379	Caprylic Acid	HSD11B1
379	Caprylic Acid	HSD11B2
379	Caprylic Acid	HSD17B3
379	Caprylic Acid	KDM2A
379	Caprylic Acid	KDM5C
379	Caprylic Acid	NPC1L1
379	Caprylic Acid	NR1H4
379	Caprylic Acid	PHF8
379	Caprylic Acid	PLG
379	Caprylic Acid	POLB
379	Caprylic Acid	PPARA
379	Caprylic Acid	PPARD
379	Caprylic Acid	PTGDR2
379	Caprylic Acid	PTGER2
379	Caprylic Acid	PTGES
379	Caprylic Acid	PTGFR
379	Caprylic Acid	PTGS2
379	Caprylic Acid	PTPN1
379	Caprylic Acid	SAE1
379	Caprylic Acid	SERPINA6
379	Caprylic Acid	SHBG
379	Caprylic Acid	SLC22A6
379	Caprylic Acid	UGT2B7
379	Caprylic Acid	VDR
125207	Dodecenoic Acid	ACP1
125207	Dodecenoic Acid	AKR1B1
125207	Dodecenoic Acid	AKR1B10
125207	Dodecenoic Acid	AR
125207	Dodecenoic Acid	CDC25A
125207	Dodecenoic Acid	CDC25C
125207	Dodecenoic Acid	CES2
125207	Dodecenoic Acid	CMA1
125207	Dodecenoic Acid	CNR1

125207	Dodecenoic Acid	CSNK2A1
125207	Dodecenoic Acid	CTSG
125207	Dodecenoic Acid	CYP19A1
125207	Dodecenoic Acid	EDNRA
125207	Dodecenoic Acid	FAAH
125207	Dodecenoic Acid	FABP1
125207	Dodecenoic Acid	FABP2
125207	Dodecenoic Acid	FABP3
125207	Dodecenoic Acid	FABP4
125207	Dodecenoic Acid	FABP5
125207	Dodecenoic Acid	FFAR1
125207	Dodecenoic Acid	FFAR4
125207	Dodecenoic Acid	G6PD
125207	Dodecenoic Acid	GABBR1
125207	Dodecenoic Acid	GSR
125207	Dodecenoic Acid	HMGCR
125207	Dodecenoic Acid	HSD11B1
125207	Dodecenoic Acid	HSD17B3
125207	Dodecenoic Acid	IMPDH2
125207	Dodecenoic Acid	KDM2A
125207	Dodecenoic Acid	KDM5C
125207	Dodecenoic Acid	LTB4R
125207	Dodecenoic Acid	MAPK1
125207	Dodecenoic Acid	MAPK14
125207	Dodecenoic Acid	MMP12
125207	Dodecenoic Acid	MMP2
125207	Dodecenoic Acid	NR1H3
125207	Dodecenoic Acid	NR1H4
125207	Dodecenoic Acid	PDE4A
125207	Dodecenoic Acid	PDE4B
125207	Dodecenoic Acid	PDE4D
125207	Dodecenoic Acid	PHF8
125207	Dodecenoic Acid	PIN1
125207	Dodecenoic Acid	POLB
125207	Dodecenoic Acid	PPARA
125207	Dodecenoic Acid	PPARD
125207	Dodecenoic Acid	PPARG
125207	Dodecenoic Acid	PREP
125207	Dodecenoic Acid	PTGES
125207	Dodecenoic Acid	PTGFR
125207	Dodecenoic Acid	PTGS1
125207	Dodecenoic Acid	PTGS2
125207	Dodecenoic Acid	PTPN1
125207	Dodecenoic Acid	PTPN2

125207	Dodecenoic Acid	PTPN6
125207	Dodecenoic Acid	PTPRF
125207	Dodecenoic Acid	RARA
125207	Dodecenoic Acid	RARB
125207	Dodecenoic Acid	RARG
125207	Dodecenoic Acid	RORC
125207	Dodecenoic Acid	SAE1
125207	Dodecenoic Acid	SCD
125207	Dodecenoic Acid	SERPINA6
125207	Dodecenoic Acid	SHBG
125207	Dodecenoic Acid	SLC16A1
125207	Dodecenoic Acid	SLC22A12
125207	Dodecenoic Acid	SLC22A6
125207	Dodecenoic Acid	TERT
125207	Dodecenoic Acid	THRA
125207	Dodecenoic Acid	THRB
125207	Dodecenoic Acid	TOP1
125207	Dodecenoic Acid	TOP2A
125207	Dodecenoic Acid	UGT2B7
125207	Dodecenoic Acid	VDR
190	Adenine	ADORA1
190	Adenine	ADORA2A
190	Adenine	ADORA2B
190	Adenine	CCNE1
190	Adenine	GDA
10368709	25-Hydroxy-3-Epidehydrotumulosic Acid	ACE
10368709	25-Hydroxy-3-Epidehydrotumulosic Acid	ACP1
10368709	25-Hydroxy-3-Epidehydrotumulosic Acid	AGTR1
10368709	25-Hydroxy-3-Epidehydrotumulosic Acid	AGTR2
10368709	25-Hydroxy-3-Epidehydrotumulosic Acid	AKR1B10
10368709	25-Hydroxy-3-Epidehydrotumulosic Acid	ALOX12
10368709	25-Hydroxy-3-Epidehydrotumulosic Acid	ALOX5AP
10368709	25-Hydroxy-3-Epidehydrotumulosic Acid	AR
10368709	25-Hydroxy-3-Epidehydrotumulosic Acid	BACE1

10368709	25-Hydroxy-3-Epidehydrotumulosic Acid	CASP1
10368709	25-Hydroxy-3-Epidehydrotumulosic Acid	CASP3
10368709	25-Hydroxy-3-Epidehydrotumulosic Acid	CASP8
10368709	25-Hydroxy-3-Epidehydrotumulosic Acid	CCR1
10368709	25-Hydroxy-3-Epidehydrotumulosic Acid	CD81
10368709	25-Hydroxy-3-Epidehydrotumulosic Acid	CDC25B
10368709	25-Hydroxy-3-Epidehydrotumulosic Acid	CES2
10368709	25-Hydroxy-3-Epidehydrotumulosic Acid	CTSA
10368709	25-Hydroxy-3-Epidehydrotumulosic Acid	CYP17A1
10368709	25-Hydroxy-3-Epidehydrotumulosic Acid	CYP19A1
10368709	25-Hydroxy-3-Epidehydrotumulosic Acid	CYP51A1
10368709	25-Hydroxy-3-Epidehydrotumulosic Acid	CYSLTR1
10368709	25-Hydroxy-3-Epidehydrotumulosic Acid	EDNRA
10368709	25-Hydroxy-3-Epidehydrotumulosic Acid	EDNRB
10368709	25-Hydroxy-3-Epidehydrotumulosic Acid	ESR1
10368709	25-Hydroxy-3-Epidehydrotumulosic Acid	ESR2
10368709	25-Hydroxy-3-Epidehydrotumulosic Acid	F10
10368709	25-Hydroxy-3-Epidehydrotumulosic Acid	F11
10368709	25-Hydroxy-3-Epidehydrotumulosic Acid	FDFT1
10368709	25-Hydroxy-3-Epidehydrotumulosic Acid	FNTA
10368709	25-Hydroxy-3-Epidehydrotumulosic Acid	G6PD
10368709	25-Hydroxy-3-Epidehydrotumulosic Acid	GPBAR1

10368709	25-Hydroxy-3-Epidehydrotumulosic Acid	HMGCR
10368709	25-Hydroxy-3-Epidehydrotumulosic Acid	HSD11B1
10368709	25-Hydroxy-3-Epidehydrotumulosic Acid	HSD11B2
10368709	25-Hydroxy-3-Epidehydrotumulosic Acid	HSP90AB1
10368709	25-Hydroxy-3-Epidehydrotumulosic Acid	IMPDH1
10368709	25-Hydroxy-3-Epidehydrotumulosic Acid	IMPDH2
10368709	25-Hydroxy-3-Epidehydrotumulosic Acid	ITGA2B
10368709	25-Hydroxy-3-Epidehydrotumulosic Acid	ITGA4
10368709	25-Hydroxy-3-Epidehydrotumulosic Acid	ITGAV
10368709	25-Hydroxy-3-Epidehydrotumulosic Acid	ITGAV
10368709	25-Hydroxy-3-Epidehydrotumulosic Acid	ITGAV
10368709	25-Hydroxy-3-Epidehydrotumulosic Acid	ITGB1
10368709	25-Hydroxy-3-Epidehydrotumulosic Acid	ITGB5
10368709	25-Hydroxy-3-Epidehydrotumulosic Acid	KCNH2
10368709	25-Hydroxy-3-Epidehydrotumulosic Acid	LNPEP
10368709	25-Hydroxy-3-Epidehydrotumulosic Acid	LTB4R
10368709	25-Hydroxy-3-Epidehydrotumulosic Acid	MME
10368709	25-Hydroxy-3-Epidehydrotumulosic Acid	MMP1
10368709	25-Hydroxy-3-Epidehydrotumulosic Acid	MMP10
10368709	25-Hydroxy-3-Epidehydrotumulosic Acid	MMP12
10368709	25-Hydroxy-3-Epidehydrotumulosic Acid	MMP13
10368709	25-Hydroxy-3-Epidehydrotumulosic Acid	MMP2

10368709	25-Hydroxy-3-Epidehydrotumulosic Acid	MMP3
10368709	25-Hydroxy-3-Epidehydrotumulosic Acid	MMP8
10368709	25-Hydroxy-3-Epidehydrotumulosic Acid	MMP9
10368709	25-Hydroxy-3-Epidehydrotumulosic Acid	NOS2
10368709	25-Hydroxy-3-Epidehydrotumulosic Acid	NR3C1
10368709	25-Hydroxy-3-Epidehydrotumulosic Acid	OPRD1
10368709	25-Hydroxy-3-Epidehydrotumulosic Acid	OPRK1
10368709	25-Hydroxy-3-Epidehydrotumulosic Acid	PDE4D
10368709	25-Hydroxy-3-Epidehydrotumulosic Acid	PDE5A
10368709	25-Hydroxy-3-Epidehydrotumulosic Acid	PGR
10368709	25-Hydroxy-3-Epidehydrotumulosic Acid	PIK3CA
10368709	25-Hydroxy-3-Epidehydrotumulosic Acid	PLA2G1B
10368709	25-Hydroxy-3-Epidehydrotumulosic Acid	POLB
10368709	25-Hydroxy-3-Epidehydrotumulosic Acid	PPARA
10368709	25-Hydroxy-3-Epidehydrotumulosic Acid	PPARD
10368709	25-Hydroxy-3-Epidehydrotumulosic Acid	PPARG
10368709	25-Hydroxy-3-Epidehydrotumulosic Acid	PRKCH
10368709	25-Hydroxy-3-Epidehydrotumulosic Acid	PTGDR
10368709	25-Hydroxy-3-Epidehydrotumulosic Acid	PTGDR2
10368709	25-Hydroxy-3-Epidehydrotumulosic Acid	PTGER1
10368709	25-Hydroxy-3-Epidehydrotumulosic Acid	PTGER2
10368709	25-Hydroxy-3-Epidehydrotumulosic Acid	PTGER3

10368709	25-Hydroxy-3-Epidehydrotumulosic Acid	PTGER4
10368709	25-Hydroxy-3-Epidehydrotumulosic Acid	PTGES
10368709	25-Hydroxy-3-Epidehydrotumulosic Acid	PTGFR
10368709	25-Hydroxy-3-Epidehydrotumulosic Acid	PTPN1
10368709	25-Hydroxy-3-Epidehydrotumulosic Acid	PTPN11
10368709	25-Hydroxy-3-Epidehydrotumulosic Acid	PTPN2
10368709	25-Hydroxy-3-Epidehydrotumulosic Acid	PTPRF
10368709	25-Hydroxy-3-Epidehydrotumulosic Acid	RASGRP3
10368709	25-Hydroxy-3-Epidehydrotumulosic Acid	REN
10368709	25-Hydroxy-3-Epidehydrotumulosic Acid	RORC
10368709	25-Hydroxy-3-Epidehydrotumulosic Acid	S1PR2
10368709	25-Hydroxy-3-Epidehydrotumulosic Acid	SERPINA6
10368709	25-Hydroxy-3-Epidehydrotumulosic Acid	SHBG
10368709	25-Hydroxy-3-Epidehydrotumulosic Acid	TBXAS1
10368709	25-Hydroxy-3-Epidehydrotumulosic Acid	THRA
10368709	25-Hydroxy-3-Epidehydrotumulosic Acid	THRB
10368709	25-Hydroxy-3-Epidehydrotumulosic Acid	TNF
10368709	25-Hydroxy-3-Epidehydrotumulosic Acid	TOP2A
10368709	25-Hydroxy-3-Epidehydrotumulosic Acid	TRPM8
10368709	25-Hydroxy-3-Epidehydrotumulosic Acid	TYMS
10368709	25-Hydroxy-3-Epidehydrotumulosic Acid	VDR
10368709	25-Hydroxy-3-Epidehydrotumulosic Acid	XIAP

8180	Undecanoic Acid	AKR1B1
8180	Undecanoic Acid	AKR1B10
8180	Undecanoic Acid	AKR1C3
8180	Undecanoic Acid	AR
8180	Undecanoic Acid	BCAT2
8180	Undecanoic Acid	CA1
8180	Undecanoic Acid	CA12
8180	Undecanoic Acid	CA2
8180	Undecanoic Acid	CACNA2D1
8180	Undecanoic Acid	CDC25A
8180	Undecanoic Acid	CDC45
8180	Undecanoic Acid	CHRNA7
8180	Undecanoic Acid	CYP19A1
8180	Undecanoic Acid	DUSP3
8180	Undecanoic Acid	FABP2
8180	Undecanoic Acid	FABP3
8180	Undecanoic Acid	FABP4
8180	Undecanoic Acid	FABP5
8180	Undecanoic Acid	FFAR1
8180	Undecanoic Acid	FFAR4
8180	Undecanoic Acid	FNTA
8180	Undecanoic Acid	G6PD
8180	Undecanoic Acid	GABBR1
8180	Undecanoic Acid	GABRA2
8180	Undecanoic Acid	GPBAR1
8180	Undecanoic Acid	GRM5
8180	Undecanoic Acid	GSTK1
8180	Undecanoic Acid	HAO1
8180	Undecanoic Acid	HMGCR
8180	Undecanoic Acid	HSD11B1
8180	Undecanoic Acid	HSD11B2
8180	Undecanoic Acid	HSD17B3
8180	Undecanoic Acid	IMPDH2
8180	Undecanoic Acid	KDM2A
8180	Undecanoic Acid	KDM5C
8180	Undecanoic Acid	LTA4H
8180	Undecanoic Acid	NPC1L1
8180	Undecanoic Acid	NR1H4
8180	Undecanoic Acid	PHF8
8180	Undecanoic Acid	POLB
8180	Undecanoic Acid	PPARA
8180	Undecanoic Acid	PPARD
8180	Undecanoic Acid	PTGDR2
8180	Undecanoic Acid	PTGER2

8180	Undecanoic Acid	PTGFR
8180	Undecanoic Acid	PTPN1
8180	Undecanoic Acid	PTPRC
8180	Undecanoic Acid	RARA
8180	Undecanoic Acid	RARB
8180	Undecanoic Acid	RARG
8180	Undecanoic Acid	RBP4
8180	Undecanoic Acid	RXRA
8180	Undecanoic Acid	SAE1
8180	Undecanoic Acid	SERPINA6
8180	Undecanoic Acid	SHBG
8180	Undecanoic Acid	SLC16A1
8180	Undecanoic Acid	SLC22A12
8180	Undecanoic Acid	SLC22A6
8180	Undecanoic Acid	TBXA2R
8180	Undecanoic Acid	THRA
8180	Undecanoic Acid	THRB
8180	Undecanoic Acid	UGT2B7
8180	Undecanoic Acid	VDR
91510	Inermine	ADAM17
91510	Inermine	ADCY5
91510	Inermine	ALOX12
91510	Inermine	ALOX15
91510	Inermine	AR
91510	Inermine	BRAF
91510	Inermine	CA14
91510	Inermine	CCND1
91510	Inermine	CCND2
91510	Inermine	CCND3
91510	Inermine	CDK4
91510	Inermine	CHEK1
91510	Inermine	CHEK2
91510	Inermine	CLK1
91510	Inermine	DGAT1
91510	Inermine	DYRK1B
91510	Inermine	ERN1
91510	Inermine	ESR1
91510	Inermine	ESRRA
91510	Inermine	ESRRB
91510	Inermine	EZR
91510	Inermine	F3
91510	Inermine	GRK2
91510	Inermine	GSK3B
91510	Inermine	GSTM2

91510	Inermine	GSTP1
91510	Inermine	HDAC2
91510	Inermine	HDAC2
91510	Inermine	HDAC4
91510	Inermine	HSD17B2
91510	Inermine	HSD17B3
91510	Inermine	IKBKB
91510	Inermine	IMPDH1
91510	Inermine	IMPDH2
91510	Inermine	JAK3
91510	Inermine	KDR
91510	Inermine	LCK
91510	Inermine	LNPEP
91510	Inermine	MAP3K8
91510	Inermine	MAPKAPK2
91510	Inermine	MIF
91510	Inermine	MMP8
91510	Inermine	MTOR
91510	Inermine	NEK1
91510	Inermine	PARP1
91510	Inermine	PGF
91510	Inermine	PIK3CA
91510	Inermine	PIK3CB
91510	Inermine	PIK3CD
91510	Inermine	PIK3CG
91510	Inermine	PIM2
91510	Inermine	PTPN1
91510	Inermine	RAF1
91510	Inermine	RET
91510	Inermine	RPS6KA1
91510	Inermine	RPS6KA3
91510	Inermine	SRC
91510	Inermine	SYK
91510	Inermine	TBK1
91510	Inermine	TNKS
91510	Inermine	TNKS2
91510	Inermine	TRPM8
91510	Inermine	TTR
91510	Inermine	TUBB1
91510	Inermine	VEGFA
91510	Inermine	WEE1
5280448	Calycosin	ABCB1
5280448	Calycosin	ABCC1
5280448	Calycosin	ABCG2

5280448	Calycosin	ACHE
5280448	Calycosin	ADORA1
5280448	Calycosin	ADORA2A
5280448	Calycosin	ALDH2
5280448	Calycosin	ALOX12
5280448	Calycosin	ALOX15
5280448	Calycosin	CA1
5280448	Calycosin	CA12
5280448	Calycosin	CA2
5280448	Calycosin	CA4
5280448	Calycosin	CA5B
5280448	Calycosin	CA7
5280448	Calycosin	CBR1
5280448	Calycosin	CDC25B
5280448	Calycosin	CDC7
5280448	Calycosin	CYP19A1
5280448	Calycosin	CYP1B1
5280448	Calycosin	DUSP3
5280448	Calycosin	EGFR
5280448	Calycosin	ESR1
5280448	Calycosin	ESR2
5280448	Calycosin	ESRRA
5280448	Calycosin	ESRRB
5280448	Calycosin	F10
5280448	Calycosin	HSD17B1
5280448	Calycosin	HSD17B2
5280448	Calycosin	HSP90AB1
5280448	Calycosin	HTR2A
5280448	Calycosin	HTR2C
5280448	Calycosin	IL2
5280448	Calycosin	MAOA
5280448	Calycosin	MAOB
5280448	Calycosin	MGAM
5280448	Calycosin	MIF
5280448	Calycosin	NOX4
5280448	Calycosin	PFKFB3
5280448	Calycosin	PLAT
5280448	Calycosin	PLAU
5280448	Calycosin	PON1
5280448	Calycosin	PPARA
5280448	Calycosin	PTGS1
5280448	Calycosin	PTPN1
5280448	Calycosin	PTPRS
5280448	Calycosin	SLC6A2

5280448	Calycosin	STS
5280448	Calycosin	TBXAS1
5280448	Calycosin	TLR9
5280448	Calycosin	TYR
5280448	Calycosin	XDH
5280863	kaempferol	ABCB1
5280863	kaempferol	ABCC1
5280863	kaempferol	ABCG2
5280863	kaempferol	ACHE
5280863	kaempferol	ADORA1
5280863	kaempferol	ADORA2A
5280863	kaempferol	AHR
5280863	kaempferol	AKR1A1
5280863	kaempferol	AKR1B1
5280863	kaempferol	AKR1B10
5280863	kaempferol	AKR1C1
5280863	kaempferol	AKR1C2
5280863	kaempferol	AKR1C3
5280863	kaempferol	AKR1C4
5280863	kaempferol	AKT1
5280863	kaempferol	ALK
5280863	kaempferol	ALOX12
5280863	kaempferol	ALOX15
5280863	kaempferol	ALOX5
5280863	kaempferol	AMY1A
5280863	kaempferol	APP
5280863	kaempferol	ARG1
5280863	kaempferol	AURKB
5280863	kaempferol	AVPR2
5280863	kaempferol	AXL
5280863	kaempferol	BACE1
5280863	kaempferol	BCHE
5280863	kaempferol	CA1
5280863	kaempferol	CA12
5280863	kaempferol	CA13
5280863	kaempferol	CA14
5280863	kaempferol	CA2
5280863	kaempferol	CA3
5280863	kaempferol	CA4
5280863	kaempferol	CA5A
5280863	kaempferol	CA6
5280863	kaempferol	CA7
5280863	kaempferol	CA9
5280863	kaempferol	CAMK2B

5280863	kaempferol	CCNB1
5280863	kaempferol	CCNB2
5280863	kaempferol	CCNB3
5280863	kaempferol	CD38
5280863	kaempferol	CDK1
5280863	kaempferol	CDK1
5280863	kaempferol	CDK2
5280863	kaempferol	CDK5
5280863	kaempferol	CDK5R1
5280863	kaempferol	CDK6
5280863	kaempferol	CFTR
5280863	kaempferol	CSNK2A1
5280863	kaempferol	CXCR1
5280863	kaempferol	CYP19A1
5280863	kaempferol	CYP1B1
5280863	kaempferol	DAPK1
5280863	kaempferol	DRD4
5280863	kaempferol	EGFR
5280863	kaempferol	ESR1
5280863	kaempferol	ESR2
5280863	kaempferol	ESRRA
5280863	kaempferol	F2
5280863	kaempferol	FLT3
5280863	kaempferol	GLO1
5280863	kaempferol	GPR35
5280863	kaempferol	GRK6
5280863	kaempferol	GSK3B
5280863	kaempferol	HSD17B1
5280863	kaempferol	HSD17B2
5280863	kaempferol	IGF1R
5280863	kaempferol	KDR
5280863	kaempferol	MAOA
5280863	kaempferol	MET
5280863	kaempferol	MMP12
5280863	kaempferol	MMP13
5280863	kaempferol	MMP2
5280863	kaempferol	MMP3
5280863	kaempferol	MMP9
5280863	kaempferol	MPG
5280863	kaempferol	MPO
5280863	kaempferol	NEK2
5280863	kaempferol	NEK6
5280863	kaempferol	NOX4
5280863	kaempferol	NUAK1

5280863	kaempferol	PARP1
5280863	kaempferol	PFKFB3
5280863	kaempferol	PIK3R1
5280863	kaempferol	PIM1
5280863	kaempferol	PKN1
5280863	kaempferol	PLA2G1B
5280863	kaempferol	PLK1
5280863	kaempferol	PTGS2
5280863	kaempferol	PTK2
5280863	kaempferol	PTPRS
5280863	kaempferol	PYGL
5280863	kaempferol	SLC22A12
5280863	kaempferol	SRC
5280863	kaempferol	SYK
5280863	kaempferol	TERT
5280863	kaempferol	TNKS
5280863	kaempferol	TNKS2
5280863	kaempferol	TOP1
5280863	kaempferol	TTR
5280863	kaempferol	TYR
5280863	kaempferol	XDH
439246	naringenin	ABCC1
439246	naringenin	ABCG2
439246	naringenin	ADCY5
439246	naringenin	ADORA1
439246	naringenin	ADORA3
439246	naringenin	AKR1B1
439246	naringenin	AKR1C3
439246	naringenin	ALOX12
439246	naringenin	APP
439246	naringenin	BACE1
439246	naringenin	BCHE
439246	naringenin	BCL2
439246	naringenin	BCL2L1
439246	naringenin	CA1
439246	naringenin	CA12
439246	naringenin	CA13
439246	naringenin	CA2
439246	naringenin	CA3
439246	naringenin	CA4
439246	naringenin	CA5A
439246	naringenin	CA5B
439246	naringenin	CA6
439246	naringenin	CA7

439246	naringenin	CA9
439246	naringenin	CBR1
439246	naringenin	CDK5
439246	naringenin	CDK5R1
439246	naringenin	CES1
439246	naringenin	CES2
439246	naringenin	CHRNA7
439246	naringenin	CLK1
439246	naringenin	CTSL
439246	naringenin	CYP19A1
439246	naringenin	CYP1B1
439246	naringenin	CYP2C9
439246	naringenin	CYP3A4
439246	naringenin	DYRK1A
439246	naringenin	DYRK1B
439246	naringenin	EDNRA
439246	naringenin	ESR1
439246	naringenin	ESR2
439246	naringenin	ESRRA
439246	naringenin	ESRRB
439246	naringenin	F3
439246	naringenin	FGFR1
439246	naringenin	GRM5
439246	naringenin	HNF4A
439246	naringenin	HSD17B1
439246	naringenin	HSD17B14
439246	naringenin	HSD17B2
439246	naringenin	IGF1R
439246	naringenin	IGFBP3
439246	naringenin	INSR
439246	naringenin	KDR
439246	naringenin	KIT
439246	naringenin	KLK1
439246	naringenin	KLK2
439246	naringenin	LCK
439246	naringenin	MAOB
439246	naringenin	MET
439246	naringenin	MMP12
439246	naringenin	MMP13
439246	naringenin	MMP2
439246	naringenin	MMP3
439246	naringenin	NOX4
439246	naringenin	NQO2
439246	naringenin	PGF

439246	naringenin	PIK3CA
439246	naringenin	PIK3CB
439246	naringenin	PLA2G10
439246	naringenin	PLA2G1B
439246	naringenin	PLA2G2A
439246	naringenin	PLA2G5
439246	naringenin	POLB
439246	naringenin	PPARG
439246	naringenin	PTGER1
439246	naringenin	PTGER2
439246	naringenin	PTGER3
439246	naringenin	PTGER4
439246	naringenin	PTGS1
439246	naringenin	RXRA
439246	naringenin	SERPINE1
439246	naringenin	SHBG
439246	naringenin	SIRT2
439246	naringenin	SLC5A2
439246	naringenin	SRC
439246	naringenin	SYK
439246	naringenin	TAS2R31
439246	naringenin	VCP
439246	naringenin	VEGFA
439246	naringenin	WEE1
439246	naringenin	YWHAG
197678	(2S)-2-[4-hydroxy-3-(3-methylbut-2-enyl)phenyl]-8,8-dimethyl-2,3-dihydropyrano[2,3-f]chromen-4-one	CTSK
197678	(2S)-2-[4-hydroxy-3-(3-methylbut-2-enyl)phenyl]-8,8-dimethyl-2,3-dihydropyrano[2,3-f]chromen-4-one	ODC1
10291003	euchrenone	ABCC1
10291003	euchrenone	ABCG2
10291003	euchrenone	ABL1
10291003	euchrenone	ACHE
10291003	euchrenone	ADAM17
10291003	euchrenone	ADORA1
10291003	euchrenone	ADORA2A
10291003	euchrenone	ADORA2B
10291003	euchrenone	ADORA3
10291003	euchrenone	ADRA2A
10291003	euchrenone	ADRA2B

10291003	euchrenone	ADRA2C
10291003	euchrenone	AKR1B1
10291003	euchrenone	AKR1B10
10291003	euchrenone	AKR1C3
10291003	euchrenone	ALOX5
10291003	euchrenone	AURKA
10291003	euchrenone	BACE1
10291003	euchrenone	BCHE
10291003	euchrenone	BMP1
10291003	euchrenone	BRAF
10291003	euchrenone	CA12
10291003	euchrenone	CA4
10291003	euchrenone	CA7
10291003	euchrenone	CAMK2D
10291003	euchrenone	CBR1
10291003	euchrenone	CDK4
10291003	euchrenone	CES1
10291003	euchrenone	CES2
10291003	euchrenone	CHEK1
10291003	euchrenone	CHRNA7
10291003	euchrenone	CLK1
10291003	euchrenone	CTSK
10291003	euchrenone	CTSL
10291003	euchrenone	CYP19A1
10291003	euchrenone	CYP1B1
10291003	euchrenone	DRD1
10291003	euchrenone	DRD3
10291003	euchrenone	DYRK1B
10291003	euchrenone	EPHA1
10291003	euchrenone	EPHA2
10291003	euchrenone	EPHA3
10291003	euchrenone	EPHA4
10291003	euchrenone	EPHA5
10291003	euchrenone	EPHA7
10291003	euchrenone	EPHA8
10291003	euchrenone	EPHB1
10291003	euchrenone	EPHB2
10291003	euchrenone	EPHB3
10291003	euchrenone	EPHB4
10291003	euchrenone	ESR1
10291003	euchrenone	ESR2
10291003	euchrenone	ESRRA
10291003	euchrenone	ESRRB
10291003	euchrenone	GCGR

10291003	euchrenone	GSK3B
10291003	euchrenone	HDAC2
10291003	euchrenone	HSD17B1
10291003	euchrenone	HSD17B2
10291003	euchrenone	HSP90AA1
10291003	euchrenone	HSP90AB1
10291003	euchrenone	KDM1A
10291003	euchrenone	KLK1
10291003	euchrenone	KLK2
10291003	euchrenone	LCK
10291003	euchrenone	MAOB
10291003	euchrenone	MAPK8
10291003	euchrenone	MCL1
10291003	euchrenone	MMP1
10291003	euchrenone	MMP12
10291003	euchrenone	MMP13
10291003	euchrenone	MMP3
10291003	euchrenone	MMP7
10291003	euchrenone	MMP8
10291003	euchrenone	NAAA
10291003	euchrenone	NOX4
10291003	euchrenone	ODC1
10291003	euchrenone	PDE7A
10291003	euchrenone	PIM1
10291003	euchrenone	PIM2
10291003	euchrenone	PIM3
10291003	euchrenone	PITRM1
10291003	euchrenone	PLA2G10
10291003	euchrenone	PLA2G1B
10291003	euchrenone	PLA2G2A
10291003	euchrenone	PLA2G5
10291003	euchrenone	POLB
10291003	euchrenone	PPARG
10291003	euchrenone	PTGS1
10291003	euchrenone	PTPN1
10291003	euchrenone	RPS6KB1
10291003	euchrenone	RXRA
10291003	euchrenone	SERPINE1
10291003	euchrenone	SGK1
10291003	euchrenone	SHBG
10291003	euchrenone	SLC5A2
10291003	euchrenone	SYK
10291003	euchrenone	TAS2R31
10291003	euchrenone	TNF

10291003	euchrenone	WEE1
480784	glyasperin B	ABCG2
480784	glyasperin B	ABL1
480784	glyasperin B	ACACB
480784	glyasperin B	ACVR1
480784	glyasperin B	ADAM17
480784	glyasperin B	ADAMTS4
480784	glyasperin B	ADAMTS5
480784	glyasperin B	ADORA2A
480784	glyasperin B	ADORA2B
480784	glyasperin B	AKR1B10
480784	glyasperin B	ALDH2
480784	glyasperin B	ALK
480784	glyasperin B	ALOX15
480784	glyasperin B	ALPL
480784	glyasperin B	AR
480784	glyasperin B	AURKA
480784	glyasperin B	BLK
480784	glyasperin B	BMP1
480784	glyasperin B	BMX
480784	glyasperin B	BRAF
480784	glyasperin B	CASP3
480784	glyasperin B	CASP6
480784	glyasperin B	CASP7
480784	glyasperin B	CCNA1
480784	glyasperin B	CCNA2
480784	glyasperin B	CCNB1
480784	glyasperin B	CCNB1
480784	glyasperin B	CCNB2
480784	glyasperin B	CCNB3
480784	glyasperin B	CCNE1
480784	glyasperin B	CCNE1
480784	glyasperin B	CDC25A
480784	glyasperin B	CDK1
480784	glyasperin B	CDK1
480784	glyasperin B	CDK2
480784	glyasperin B	CDK2
480784	glyasperin B	CDK3
480784	glyasperin B	CHEK1
480784	glyasperin B	CHEK2
480784	glyasperin B	CHRNA7
480784	glyasperin B	COMT
480784	glyasperin B	CSK
480784	glyasperin B	CTSL

480784	glyasperin B	CYP19A1
480784	glyasperin B	DRD1
480784	glyasperin B	DRD3
480784	glyasperin B	ENPP1
480784	glyasperin B	EPHA2
480784	glyasperin B	EPHB2
480784	glyasperin B	ESR1
480784	glyasperin B	ESR2
480784	glyasperin B	FNTA
480784	glyasperin B	FNTB
480784	glyasperin B	FYN
480784	glyasperin B	GSK3A
480784	glyasperin B	GSK3B
480784	glyasperin B	GSTP1
480784	glyasperin B	HDAC1
480784	glyasperin B	HDAC2
480784	glyasperin B	HDAC3
480784	glyasperin B	HDAC6
480784	glyasperin B	HDAC8
480784	glyasperin B	HDAC9
480784	glyasperin B	HSD17B3
480784	glyasperin B	HSP90AA1
480784	glyasperin B	IMPDH1
480784	glyasperin B	JAK3
480784	glyasperin B	KDM1A
480784	glyasperin B	KDR
480784	glyasperin B	KIT
480784	glyasperin B	KLK1
480784	glyasperin B	KLK2
480784	glyasperin B	LCK
480784	glyasperin B	MAOB
480784	glyasperin B	MARK1
480784	glyasperin B	MMP1
480784	glyasperin B	MMP3
480784	glyasperin B	MMP7
480784	glyasperin B	MMP8
480784	glyasperin B	MTOR
480784	glyasperin B	MYLK
480784	glyasperin B	NPY5R
480784	glyasperin B	PDE10A
480784	glyasperin B	PDE4A
480784	glyasperin B	PDE4B
480784	glyasperin B	PDE4C
480784	glyasperin B	PDGFRA

480784	glyasperin B	PDGFRB
480784	glyasperin B	PDGFRB
480784	glyasperin B	PDK1
480784	glyasperin B	PIK3CA
480784	glyasperin B	PIM1
480784	glyasperin B	PIM2
480784	glyasperin B	PIM3
480784	glyasperin B	PLAA
480784	glyasperin B	PLK1
480784	glyasperin B	PPARG
480784	glyasperin B	PTPN1
480784	glyasperin B	RAF1
480784	glyasperin B	RET
480784	glyasperin B	ROCK1
480784	glyasperin B	ROCK2
480784	glyasperin B	RXRA
480784	glyasperin B	SCD
480784	glyasperin B	STAT6
480784	glyasperin B	SYK
480784	glyasperin B	TAS2R31
480784	glyasperin B	TGFBR1
480784	glyasperin B	TYR
480784	glyasperin B	YES1
392442	glyasperin F	ADAM17
392442	glyasperin F	ADORA2A
392442	glyasperin F	AGTR1
392442	glyasperin F	AR
392442	glyasperin F	AURKA
392442	glyasperin F	AURKB
392442	glyasperin F	BCL2
392442	glyasperin F	BCL2L1
392442	glyasperin F	BMP1
392442	glyasperin F	BRAF
392442	glyasperin F	CCNB1
392442	glyasperin F	CCNE1
392442	glyasperin F	CCNE1
392442	glyasperin F	CCNE1
392442	glyasperin F	CCNE2
392442	glyasperin F	CDK1
392442	glyasperin F	CDK2
392442	glyasperin F	CDK2
392442	glyasperin F	CDK3
392442	glyasperin F	CHEK1
392442	glyasperin F	CHRNA7

392442	glyasperin F	CSNK1G1
392442	glyasperin F	CTSK
392442	glyasperin F	DNM1
392442	glyasperin F	DPP4
392442	glyasperin F	DPP7
392442	glyasperin F	DPP8
392442	glyasperin F	DRD1
392442	glyasperin F	DUT
392442	glyasperin F	ECE1
392442	glyasperin F	EIF2AK2
392442	glyasperin F	EPHB4
392442	glyasperin F	ESR1
392442	glyasperin F	ESR2
392442	glyasperin F	FLT3
392442	glyasperin F	GRK2
392442	glyasperin F	GUSB
392442	glyasperin F	HDAC1
392442	glyasperin F	HDAC4
392442	glyasperin F	HDAC5
392442	glyasperin F	HDAC7
392442	glyasperin F	HDAC8
392442	glyasperin F	HSD17B2
392442	glyasperin F	HTR7
392442	glyasperin F	KDM1A
392442	glyasperin F	LCK
392442	glyasperin F	MAOB
392442	glyasperin F	MAPK1
392442	glyasperin F	MAPK14
392442	glyasperin F	MAPK8
392442	glyasperin F	MMP14
392442	glyasperin F	MMP15
392442	glyasperin F	MMP16
392442	glyasperin F	MMP2
392442	glyasperin F	MMP26
392442	glyasperin F	MMP7
392442	glyasperin F	MMP8
392442	glyasperin F	NFKB1
392442	glyasperin F	NOX4
392442	glyasperin F	NR1H4
392442	glyasperin F	NTRK1
392442	glyasperin F	ODC1
392442	glyasperin F	PARP1
392442	glyasperin F	PARP2
392442	glyasperin F	PDE10A

392442	glyasperin F	PDE4A
392442	glyasperin F	PDE4B
392442	glyasperin F	PDE4C
392442	glyasperin F	PDE4D
392442	glyasperin F	PDK1
392442	glyasperin F	PDPK1
392442	glyasperin F	PLG
392442	glyasperin F	PLK1
392442	glyasperin F	PTPN1
392442	glyasperin F	RAF1
392442	glyasperin F	RELA
392442	glyasperin F	RET
392442	glyasperin F	ROCK1
392442	glyasperin F	RPS6KA1
392442	glyasperin F	RPS6KB1
392442	glyasperin F	SGK1
392442	glyasperin F	SLC29A1
392442	glyasperin F	SLC5A1
392442	glyasperin F	SPHK1
392442	glyasperin F	SPHK2
392442	glyasperin F	SYK
392442	glyasperin F	TGM2
392442	glyasperin F	TNF
392442	glyasperin F	TNKS
392442	glyasperin F	TNKS2
392442	glyasperin F	TRPM8
392442	glyasperin F	WEE1
480859	Glyasperin C	ACVR1
480859	Glyasperin C	ADAM17
480859	Glyasperin C	ADAMTS4
480859	Glyasperin C	ADAMTS5
480859	Glyasperin C	ADORA2A
480859	Glyasperin C	ADORA2B
480859	Glyasperin C	ADRA1A
480859	Glyasperin C	ADRA1B
480859	Glyasperin C	AKR1B10
480859	Glyasperin C	ALDH2
480859	Glyasperin C	ALOX12
480859	Glyasperin C	ALOX15
480859	Glyasperin C	ALOX15B
480859	Glyasperin C	AURKA
480859	Glyasperin C	AURKB
480859	Glyasperin C	BRAF
480859	Glyasperin C	CCKBR

480859	Glyasperin C	CDC25A
480859	Glyasperin C	CDC25B
480859	Glyasperin C	CHEK1
480859	Glyasperin C	CHEK2
480859	Glyasperin C	CHRM2
480859	Glyasperin C	CHRM3
480859	Glyasperin C	CMA1
480859	Glyasperin C	CTSD
480859	Glyasperin C	CTSL
480859	Glyasperin C	CYP11B1
480859	Glyasperin C	CYP11B2
480859	Glyasperin C	CYP2C9
480859	Glyasperin C	CYP3A4
480859	Glyasperin C	DPP4
480859	Glyasperin C	EP300
480859	Glyasperin C	EPHX2
480859	Glyasperin C	ERN1
480859	Glyasperin C	ESRRA
480859	Glyasperin C	ESRRB
480859	Glyasperin C	FAAH
480859	Glyasperin C	GNRHR
480859	Glyasperin C	GSK3B
480859	Glyasperin C	HDAC2
480859	Glyasperin C	HDAC3
480859	Glyasperin C	HDAC5
480859	Glyasperin C	HSD11B1
480859	Glyasperin C	HSD17B2
480859	Glyasperin C	ICAM1
480859	Glyasperin C	ITGAL
480859	Glyasperin C	ITGB2
480859	Glyasperin C	ITK
480859	Glyasperin C	KDM1A
480859	Glyasperin C	KIF11
480859	Glyasperin C	LCK
480859	Glyasperin C	MAOB
480859	Glyasperin C	MCL1
480859	Glyasperin C	MDM2
480859	Glyasperin C	MDM4
480859	Glyasperin C	ME1
480859	Glyasperin C	MELK
480859	Glyasperin C	MIF
480859	Glyasperin C	MMP1
480859	Glyasperin C	MMP3
480859	Glyasperin C	MMP8

480859	Glyasperin C	MPEG1
480859	Glyasperin C	MTOR
480859	Glyasperin C	NAAA
480859	Glyasperin C	NOX1
480859	Glyasperin C	NOX4
480859	Glyasperin C	P2RX3
480859	Glyasperin C	PDGFRA
480859	Glyasperin C	PDGFRB
480859	Glyasperin C	PDK1
480859	Glyasperin C	PIK3CA
480859	Glyasperin C	PTGIR
480859	Glyasperin C	PTPN1
480859	Glyasperin C	PTPN11
480859	Glyasperin C	PTPN6
480859	Glyasperin C	RAF1
480859	Glyasperin C	RET
480859	Glyasperin C	ROCK1
480859	Glyasperin C	ROCK2
480859	Glyasperin C	RPS6KB1
480859	Glyasperin C	S1PR1
480859	Glyasperin C	S1PR3
480859	Glyasperin C	SAE1
480859	Glyasperin C	SCARB1
480859	Glyasperin C	SCD
480859	Glyasperin C	SLC29A1
480859	Glyasperin C	STAT6
480859	Glyasperin C	TACR3
480859	Glyasperin C	TBXA2R
480859	Glyasperin C	TGFBR1
480859	Glyasperin C	TRPV1
480859	Glyasperin C	TYR
480859	Glyasperin C	UBA2
480859	Glyasperin C	WEE1
5318679	Isotrifoliol	ABCC1
5318679	Isotrifoliol	ACHE
5318679	Isotrifoliol	AHR
5318679	Isotrifoliol	AKR1A1
5318679	Isotrifoliol	AKR1B1
5318679	Isotrifoliol	AKR1B10
5318679	Isotrifoliol	AKR1C2
5318679	Isotrifoliol	AKR1C4
5318679	Isotrifoliol	ALOX12
5318679	Isotrifoliol	ALOX5
5318679	Isotrifoliol	APP

5318679	Isotrifoliol	ARG1
5318679	Isotrifoliol	AURKB
5318679	Isotrifoliol	AVPR2
5318679	Isotrifoliol	CA1
5318679	Isotrifoliol	CA12
5318679	Isotrifoliol	CA13
5318679	Isotrifoliol	CA14
5318679	Isotrifoliol	CA2
5318679	Isotrifoliol	CA4
5318679	Isotrifoliol	CA5A
5318679	Isotrifoliol	CA5B
5318679	Isotrifoliol	CA6
5318679	Isotrifoliol	CA7
5318679	Isotrifoliol	CA9
5318679	Isotrifoliol	CAMK2B
5318679	Isotrifoliol	CBR1
5318679	Isotrifoliol	CCNB1
5318679	Isotrifoliol	CCNB2
5318679	Isotrifoliol	CCNB3
5318679	Isotrifoliol	CD38
5318679	Isotrifoliol	CDC7
5318679	Isotrifoliol	CDK1
5318679	Isotrifoliol	CDK1
5318679	Isotrifoliol	CDK2
5318679	Isotrifoliol	CDK5
5318679	Isotrifoliol	CDK5R1
5318679	Isotrifoliol	CDK6
5318679	Isotrifoliol	CFTR
5318679	Isotrifoliol	CRHR1
5318679	Isotrifoliol	CSNK2A1
5318679	Isotrifoliol	CXCR1
5318679	Isotrifoliol	CYP1A2
5318679	Isotrifoliol	DAPK1
5318679	Isotrifoliol	DHODH
5318679	Isotrifoliol	DRD4
5318679	Isotrifoliol	EGFR
5318679	Isotrifoliol	ESR1
5318679	Isotrifoliol	ESR2
5318679	Isotrifoliol	ESRRA
5318679	Isotrifoliol	ESRRB
5318679	Isotrifoliol	F10
5318679	Isotrifoliol	F2
5318679	Isotrifoliol	FNTA
5318679	Isotrifoliol	FNTB

5318679	Isotrifoliol	GLO1
5318679	Isotrifoliol	GPR35
5318679	Isotrifoliol	GSK3B
5318679	Isotrifoliol	GSR
5318679	Isotrifoliol	HSD17B1
5318679	Isotrifoliol	HTR2C
5318679	Isotrifoliol	IGF1R
5318679	Isotrifoliol	IGFBP1
5318679	Isotrifoliol	IGFBP2
5318679	Isotrifoliol	IGFBP3
5318679	Isotrifoliol	IGFBP4
5318679	Isotrifoliol	IGFBP5
5318679	Isotrifoliol	IGFBP6
5318679	Isotrifoliol	KCNA3
5318679	Isotrifoliol	KCNA5
5318679	Isotrifoliol	KDR
5318679	Isotrifoliol	MAOA
5318679	Isotrifoliol	MCL1
5318679	Isotrifoliol	MET
5318679	Isotrifoliol	MGAM
5318679	Isotrifoliol	MMP12
5318679	Isotrifoliol	MMP13
5318679	Isotrifoliol	MMP2
5318679	Isotrifoliol	MMP3
5318679	Isotrifoliol	MMP9
5318679	Isotrifoliol	MPG
5318679	Isotrifoliol	MPO
5318679	Isotrifoliol	NEK2
5318679	Isotrifoliol	NEK6
5318679	Isotrifoliol	NOX4
5318679	Isotrifoliol	PARP1
5318679	Isotrifoliol	PFKFB3
5318679	Isotrifoliol	PIK3R1
5318679	Isotrifoliol	PKN1
5318679	Isotrifoliol	PLA2G1B
5318679	Isotrifoliol	PLAT
5318679	Isotrifoliol	PLAU
5318679	Isotrifoliol	PLK1
5318679	Isotrifoliol	PTGS2
5318679	Isotrifoliol	PTP4A3
5318679	Isotrifoliol	PYGL
5318679	Isotrifoliol	SLC22A12
5318679	Isotrifoliol	SNCA
5318679	Isotrifoliol	SYK

5318679	Isotrifoliol	TBXAS1
5318679	Isotrifoliol	TERT
5318679	Isotrifoliol	TNKS2
5318679	Isotrifoliol	TOP1
5318679	Isotrifoliol	TYR
5318679	Isotrifoliol	XDH
10881804	(E)-1-(2,4-dihydroxyphenyl)-3-(2,2-dimethylchromen-6-yl)prop-2-en-1-one	ABCG2
10881804	(E)-1-(2,4-dihydroxyphenyl)-3-(2,2-dimethylchromen-6-yl)prop-2-en-1-one	ADAM17
10881804	(E)-1-(2,4-dihydroxyphenyl)-3-(2,2-dimethylchromen-6-yl)prop-2-en-1-one	ADAMTS4
10881804	(E)-1-(2,4-dihydroxyphenyl)-3-(2,2-dimethylchromen-6-yl)prop-2-en-1-one	ADAMTS5
10881804	(E)-1-(2,4-dihydroxyphenyl)-3-(2,2-dimethylchromen-6-yl)prop-2-en-1-one	AKR1B1
10881804	(E)-1-(2,4-dihydroxyphenyl)-3-(2,2-dimethylchromen-6-yl)prop-2-en-1-one	ALDH2
10881804	(E)-1-(2,4-dihydroxyphenyl)-3-(2,2-dimethylchromen-6-yl)prop-2-en-1-one	ALOX5
10881804	(E)-1-(2,4-dihydroxyphenyl)-3-(2,2-dimethylchromen-6-yl)prop-2-en-1-one	APP
10881804	(E)-1-(2,4-dihydroxyphenyl)-3-(2,2-dimethylchromen-6-yl)prop-2-en-1-one	AR
10881804	(E)-1-(2,4-dihydroxyphenyl)-3-(2,2-dimethylchromen-6-yl)prop-2-en-1-one	BACE1
10881804	(E)-1-(2,4-dihydroxyphenyl)-3-(2,2-dimethylchromen-6-yl)prop-2-en-1-one	BACE2
10881804	(E)-1-(2,4-dihydroxyphenyl)-3-(2,2-dimethylchromen-6-yl)prop-2-en-1-one	BMP1

10881804	(E)-1-(2,4-dihydroxyphenyl)-3-(2,2-dimethylchromen-6-yl)prop-2-en-1-one	BRAF
10881804	(E)-1-(2,4-dihydroxyphenyl)-3-(2,2-dimethylchromen-6-yl)prop-2-en-1-one	CDC25A
10881804	(E)-1-(2,4-dihydroxyphenyl)-3-(2,2-dimethylchromen-6-yl)prop-2-en-1-one	CDC25B
10881804	(E)-1-(2,4-dihydroxyphenyl)-3-(2,2-dimethylchromen-6-yl)prop-2-en-1-one	CES1
10881804	(E)-1-(2,4-dihydroxyphenyl)-3-(2,2-dimethylchromen-6-yl)prop-2-en-1-one	CES2
10881804	(E)-1-(2,4-dihydroxyphenyl)-3-(2,2-dimethylchromen-6-yl)prop-2-en-1-one	CHEK1
10881804	(E)-1-(2,4-dihydroxyphenyl)-3-(2,2-dimethylchromen-6-yl)prop-2-en-1-one	CHRNA7
10881804	(E)-1-(2,4-dihydroxyphenyl)-3-(2,2-dimethylchromen-6-yl)prop-2-en-1-one	CTSL
10881804	(E)-1-(2,4-dihydroxyphenyl)-3-(2,2-dimethylchromen-6-yl)prop-2-en-1-one	CYP11B1
10881804	(E)-1-(2,4-dihydroxyphenyl)-3-(2,2-dimethylchromen-6-yl)prop-2-en-1-one	CYP11B2
10881804	(E)-1-(2,4-dihydroxyphenyl)-3-(2,2-dimethylchromen-6-yl)prop-2-en-1-one	CYP19A1
10881804	(E)-1-(2,4-dihydroxyphenyl)-3-(2,2-dimethylchromen-6-yl)prop-2-en-1-one	DPP4
10881804	(E)-1-(2,4-dihydroxyphenyl)-3-(2,2-dimethylchromen-6-yl)prop-2-en-1-one	DRD1
10881804	(E)-1-(2,4-dihydroxyphenyl)-3-(2,2-dimethylchromen-6-yl)prop-2-en-1-one	DRD3

10881804	(E)-1-(2,4-dihydroxyphenyl)-3-(2,2-dimethylchromen-6-yl)prop-2-en-1-one	DUSP3
10881804	(E)-1-(2,4-dihydroxyphenyl)-3-(2,2-dimethylchromen-6-yl)prop-2-en-1-one	EP300
10881804	(E)-1-(2,4-dihydroxyphenyl)-3-(2,2-dimethylchromen-6-yl)prop-2-en-1-one	EPHX2
10881804	(E)-1-(2,4-dihydroxyphenyl)-3-(2,2-dimethylchromen-6-yl)prop-2-en-1-one	ERN1
10881804	(E)-1-(2,4-dihydroxyphenyl)-3-(2,2-dimethylchromen-6-yl)prop-2-en-1-one	ESRRA
10881804	(E)-1-(2,4-dihydroxyphenyl)-3-(2,2-dimethylchromen-6-yl)prop-2-en-1-one	F3
10881804	(E)-1-(2,4-dihydroxyphenyl)-3-(2,2-dimethylchromen-6-yl)prop-2-en-1-one	FAAH
10881804	(E)-1-(2,4-dihydroxyphenyl)-3-(2,2-dimethylchromen-6-yl)prop-2-en-1-one	FASN
10881804	(E)-1-(2,4-dihydroxyphenyl)-3-(2,2-dimethylchromen-6-yl)prop-2-en-1-one	FLT3
10881804	(E)-1-(2,4-dihydroxyphenyl)-3-(2,2-dimethylchromen-6-yl)prop-2-en-1-one	FNTA
10881804	(E)-1-(2,4-dihydroxyphenyl)-3-(2,2-dimethylchromen-6-yl)prop-2-en-1-one	FNTB
10881804	(E)-1-(2,4-dihydroxyphenyl)-3-(2,2-dimethylchromen-6-yl)prop-2-en-1-one	GCGR
10881804	(E)-1-(2,4-dihydroxyphenyl)-3-(2,2-dimethylchromen-6-yl)prop-2-en-1-one	HDAC1
10881804	(E)-1-(2,4-dihydroxyphenyl)-3-(2,2-dimethylchromen-6-yl)prop-2-en-1-one	HDAC10

10881804	(E)-1-(2,4-dihydroxyphenyl)-3-(2,2-dimethylchromen-6-yl)prop-2-en-1-one	HDAC11
10881804	(E)-1-(2,4-dihydroxyphenyl)-3-(2,2-dimethylchromen-6-yl)prop-2-en-1-one	HDAC3
10881804	(E)-1-(2,4-dihydroxyphenyl)-3-(2,2-dimethylchromen-6-yl)prop-2-en-1-one	HDAC3
10881804	(E)-1-(2,4-dihydroxyphenyl)-3-(2,2-dimethylchromen-6-yl)prop-2-en-1-one	HDAC3
10881804	(E)-1-(2,4-dihydroxyphenyl)-3-(2,2-dimethylchromen-6-yl)prop-2-en-1-one	HDAC4
10881804	(E)-1-(2,4-dihydroxyphenyl)-3-(2,2-dimethylchromen-6-yl)prop-2-en-1-one	HDAC5
10881804	(E)-1-(2,4-dihydroxyphenyl)-3-(2,2-dimethylchromen-6-yl)prop-2-en-1-one	HDAC6
10881804	(E)-1-(2,4-dihydroxyphenyl)-3-(2,2-dimethylchromen-6-yl)prop-2-en-1-one	HDAC7
10881804	(E)-1-(2,4-dihydroxyphenyl)-3-(2,2-dimethylchromen-6-yl)prop-2-en-1-one	HDAC8
10881804	(E)-1-(2,4-dihydroxyphenyl)-3-(2,2-dimethylchromen-6-yl)prop-2-en-1-one	HDAC9
10881804	(E)-1-(2,4-dihydroxyphenyl)-3-(2,2-dimethylchromen-6-yl)prop-2-en-1-one	HIF1A
10881804	(E)-1-(2,4-dihydroxyphenyl)-3-(2,2-dimethylchromen-6-yl)prop-2-en-1-one	HPGD
10881804	(E)-1-(2,4-dihydroxyphenyl)-3-(2,2-dimethylchromen-6-yl)prop-2-en-1-one	HPGDS
10881804	(E)-1-(2,4-dihydroxyphenyl)-3-(2,2-dimethylchromen-6-yl)prop-2-en-1-one	HSD11B1

10881804	(E)-1-(2,4-dihydroxyphenyl)-3-(2,2-dimethylchromen-6-yl)prop-2-en-1-one	HSD17B3
10881804	(E)-1-(2,4-dihydroxyphenyl)-3-(2,2-dimethylchromen-6-yl)prop-2-en-1-one	IKBKE
10881804	(E)-1-(2,4-dihydroxyphenyl)-3-(2,2-dimethylchromen-6-yl)prop-2-en-1-one	KIF11
10881804	(E)-1-(2,4-dihydroxyphenyl)-3-(2,2-dimethylchromen-6-yl)prop-2-en-1-one	MAOA
10881804	(E)-1-(2,4-dihydroxyphenyl)-3-(2,2-dimethylchromen-6-yl)prop-2-en-1-one	MAOB
10881804	(E)-1-(2,4-dihydroxyphenyl)-3-(2,2-dimethylchromen-6-yl)prop-2-en-1-one	MAPK14
10881804	(E)-1-(2,4-dihydroxyphenyl)-3-(2,2-dimethylchromen-6-yl)prop-2-en-1-one	MAPKAPK2
10881804	(E)-1-(2,4-dihydroxyphenyl)-3-(2,2-dimethylchromen-6-yl)prop-2-en-1-one	MAPKAPK5
10881804	(E)-1-(2,4-dihydroxyphenyl)-3-(2,2-dimethylchromen-6-yl)prop-2-en-1-one	MELK
10881804	(E)-1-(2,4-dihydroxyphenyl)-3-(2,2-dimethylchromen-6-yl)prop-2-en-1-one	MMP1
10881804	(E)-1-(2,4-dihydroxyphenyl)-3-(2,2-dimethylchromen-6-yl)prop-2-en-1-one	MMP12
10881804	(E)-1-(2,4-dihydroxyphenyl)-3-(2,2-dimethylchromen-6-yl)prop-2-en-1-one	MMP13
10881804	(E)-1-(2,4-dihydroxyphenyl)-3-(2,2-dimethylchromen-6-yl)prop-2-en-1-one	MMP14
10881804	(E)-1-(2,4-dihydroxyphenyl)-3-(2,2-dimethylchromen-6-yl)prop-2-en-1-one	MMP3

10881804	(E)-1-(2,4-dihydroxyphenyl)-3-(2,2-dimethylchromen-6-yl)prop-2-en-1-one	MMP8
10881804	(E)-1-(2,4-dihydroxyphenyl)-3-(2,2-dimethylchromen-6-yl)prop-2-en-1-one	MMP9
10881804	(E)-1-(2,4-dihydroxyphenyl)-3-(2,2-dimethylchromen-6-yl)prop-2-en-1-one	MPEG1
10881804	(E)-1-(2,4-dihydroxyphenyl)-3-(2,2-dimethylchromen-6-yl)prop-2-en-1-one	NCOR1
10881804	(E)-1-(2,4-dihydroxyphenyl)-3-(2,2-dimethylchromen-6-yl)prop-2-en-1-one	NCOR2
10881804	(E)-1-(2,4-dihydroxyphenyl)-3-(2,2-dimethylchromen-6-yl)prop-2-en-1-one	NOS2
10881804	(E)-1-(2,4-dihydroxyphenyl)-3-(2,2-dimethylchromen-6-yl)prop-2-en-1-one	ODC1
10881804	(E)-1-(2,4-dihydroxyphenyl)-3-(2,2-dimethylchromen-6-yl)prop-2-en-1-one	PDE4A
10881804	(E)-1-(2,4-dihydroxyphenyl)-3-(2,2-dimethylchromen-6-yl)prop-2-en-1-one	PDE4B
10881804	(E)-1-(2,4-dihydroxyphenyl)-3-(2,2-dimethylchromen-6-yl)prop-2-en-1-one	PDE4C
10881804	(E)-1-(2,4-dihydroxyphenyl)-3-(2,2-dimethylchromen-6-yl)prop-2-en-1-one	PDE7A
10881804	(E)-1-(2,4-dihydroxyphenyl)-3-(2,2-dimethylchromen-6-yl)prop-2-en-1-one	PLA2G1B
10881804	(E)-1-(2,4-dihydroxyphenyl)-3-(2,2-dimethylchromen-6-yl)prop-2-en-1-one	PLEC
10881804	(E)-1-(2,4-dihydroxyphenyl)-3-(2,2-dimethylchromen-6-yl)prop-2-en-1-one	PLK1

10881804	(E)-1-(2,4-dihydroxyphenyl)-3-(2,2-dimethylchromen-6-yl)prop-2-en-1-one	PRKCD
10881804	(E)-1-(2,4-dihydroxyphenyl)-3-(2,2-dimethylchromen-6-yl)prop-2-en-1-one	PTAFR
10881804	(E)-1-(2,4-dihydroxyphenyl)-3-(2,2-dimethylchromen-6-yl)prop-2-en-1-one	PTGS2
10881804	(E)-1-(2,4-dihydroxyphenyl)-3-(2,2-dimethylchromen-6-yl)prop-2-en-1-one	PTPN1
10881804	(E)-1-(2,4-dihydroxyphenyl)-3-(2,2-dimethylchromen-6-yl)prop-2-en-1-one	ROCK1
10881804	(E)-1-(2,4-dihydroxyphenyl)-3-(2,2-dimethylchromen-6-yl)prop-2-en-1-one	ROCK2
10881804	(E)-1-(2,4-dihydroxyphenyl)-3-(2,2-dimethylchromen-6-yl)prop-2-en-1-one	S1PR1
10881804	(E)-1-(2,4-dihydroxyphenyl)-3-(2,2-dimethylchromen-6-yl)prop-2-en-1-one	SCD
10881804	(E)-1-(2,4-dihydroxyphenyl)-3-(2,2-dimethylchromen-6-yl)prop-2-en-1-one	SF3B3
10881804	(E)-1-(2,4-dihydroxyphenyl)-3-(2,2-dimethylchromen-6-yl)prop-2-en-1-one	SIGMAR1
10881804	(E)-1-(2,4-dihydroxyphenyl)-3-(2,2-dimethylchromen-6-yl)prop-2-en-1-one	SLC5A1
10881804	(E)-1-(2,4-dihydroxyphenyl)-3-(2,2-dimethylchromen-6-yl)prop-2-en-1-one	SLC9A1
10881804	(E)-1-(2,4-dihydroxyphenyl)-3-(2,2-dimethylchromen-6-yl)prop-2-en-1-one	SREBF2
10881804	(E)-1-(2,4-dihydroxyphenyl)-3-(2,2-dimethylchromen-6-yl)prop-2-en-1-one	SYK

10881804	(E)-1-(2,4-dihydroxyphenyl)-3-(2,2-dimethylchromen-6-yl)prop-2-en-1-one	TAOK1
10881804	(E)-1-(2,4-dihydroxyphenyl)-3-(2,2-dimethylchromen-6-yl)prop-2-en-1-one	TAOK3
10881804	(E)-1-(2,4-dihydroxyphenyl)-3-(2,2-dimethylchromen-6-yl)prop-2-en-1-one	TBK1
10881804	(E)-1-(2,4-dihydroxyphenyl)-3-(2,2-dimethylchromen-6-yl)prop-2-en-1-one	TLR9
10881804	(E)-1-(2,4-dihydroxyphenyl)-3-(2,2-dimethylchromen-6-yl)prop-2-en-1-one	TRPV1
10881804	(E)-1-(2,4-dihydroxyphenyl)-3-(2,2-dimethylchromen-6-yl)prop-2-en-1-one	TYR
10881804	(E)-1-(2,4-dihydroxyphenyl)-3-(2,2-dimethylchromen-6-yl)prop-2-en-1-one	VCP
114829	DFV	ABCC1
114829	DFV	ABCG2
114829	DFV	ACHE
114829	DFV	ADORA1
114829	DFV	ADORA3
114829	DFV	AKR1B1
114829	DFV	AKR1C3
114829	DFV	ALOX12
114829	DFV	AURKA
114829	DFV	BACE1
114829	DFV	BCHE
114829	DFV	BCL2L1
114829	DFV	CA1
114829	DFV	CA12
114829	DFV	CA13
114829	DFV	CA2
114829	DFV	CA3
114829	DFV	CA4
114829	DFV	CA5A
114829	DFV	CA5B
114829	DFV	CA6
114829	DFV	CA7
114829	DFV	CA9

114829	DFV	CALM1
114829	DFV	CBR1
114829	DFV	CCNE1
114829	DFV	CCNE1
114829	DFV	CDK2
114829	DFV	CDK3
114829	DFV	CES1
114829	DFV	CES2
114829	DFV	CHRNA7
114829	DFV	CLK1
114829	DFV	CYP19A1
114829	DFV	CYP1B1
114829	DFV	CYP2C9
114829	DFV	CYP3A4
114829	DFV	DNM1
114829	DFV	DUSP3
114829	DFV	DYRK1A
114829	DFV	DYRK1B
114829	DFV	EDNRA
114829	DFV	ERN1
114829	DFV	ESR1
114829	DFV	ESR2
114829	DFV	ESRRA
114829	DFV	ESRRB
114829	DFV	F3
114829	DFV	FGFR1
114829	DFV	GCGR
114829	DFV	GRM2
114829	DFV	GRM5
114829	DFV	GSK3B
114829	DFV	HDAC5
114829	DFV	HDAC7
114829	DFV	HDAC9
114829	DFV	HNF4A
114829	DFV	HSD17B1
114829	DFV	HSD17B14
114829	DFV	HSD17B2
114829	DFV	IGF1R
114829	DFV	INSR
114829	DFV	KDR
114829	DFV	KIT
114829	DFV	KLK1
114829	DFV	KLK2
114829	DFV	MAOB

114829	DFV	MET
114829	DFV	MMP12
114829	DFV	MMP13
114829	DFV	MMP2
114829	DFV	MMP3
114829	DFV	MMP9
114829	DFV	NOX4
114829	DFV	NQO2
114829	DFV	PDPK1
114829	DFV	PGF
114829	DFV	PIK3CA
114829	DFV	PIK3CB
114829	DFV	PLA2G10
114829	DFV	PLA2G1B
114829	DFV	PLA2G2A
114829	DFV	PLA2G5
114829	DFV	POLB
114829	DFV	PPARG
114829	DFV	PTGER1
114829	DFV	PTGER2
114829	DFV	PTGER3
114829	DFV	PTGER4
114829	DFV	PTGS1
114829	DFV	PTPN1
114829	DFV	RPS6KA5
114829	DFV	RXRA
114829	DFV	SERPINE1
114829	DFV	SHBG
114829	DFV	SLC5A2
114829	DFV	SRC
114829	DFV	TAS2R31
114829	DFV	VCP
114829	DFV	VEGFA
114829	DFV	WEE1
114829	DFV	YWHAG
15380912	kanzonols W	ACVR1
15380912	kanzonols W	ADAM17
15380912	kanzonols W	ADAMTS4
15380912	kanzonols W	ADAMTS5
15380912	kanzonols W	ADORA2B
15380912	kanzonols W	AGPAT2
15380912	kanzonols W	AKR1B10
15380912	kanzonols W	ALDH2
15380912	kanzonols W	ALOX5

15380912	kanzonols W	APP
15380912	kanzonols W	AR
15380912	kanzonols W	AURKA
15380912	kanzonols W	AURKB
15380912	kanzonols W	BMP1
15380912	kanzonols W	BMP4
15380912	kanzonols W	BRAF
15380912	kanzonols W	CA1
15380912	kanzonols W	CA12
15380912	kanzonols W	CA13
15380912	kanzonols W	CA7
15380912	kanzonols W	CA9
15380912	kanzonols W	CCNB1
15380912	kanzonols W	CCNB2
15380912	kanzonols W	CCNB3
15380912	kanzonols W	CDC25A
15380912	kanzonols W	CDC25B
15380912	kanzonols W	CDK1
15380912	kanzonols W	CDK2
15380912	kanzonols W	CDK5
15380912	kanzonols W	CDK5R1
15380912	kanzonols W	CES1
15380912	kanzonols W	CES2
15380912	kanzonols W	CHEK1
15380912	kanzonols W	CHRNA7
15380912	kanzonols W	COMT
15380912	kanzonols W	CTSL
15380912	kanzonols W	CYP11B1
15380912	kanzonols W	CYP11B2
15380912	kanzonols W	CYP1A2
15380912	kanzonols W	CYP1B1
15380912	kanzonols W	DPP4
15380912	kanzonols W	ELANE
15380912	kanzonols W	EP300
15380912	kanzonols W	EPHX2
15380912	kanzonols W	ERN1
15380912	kanzonols W	FLT3
15380912	kanzonols W	GCGR
15380912	kanzonols W	GRIN1
15380912	kanzonols W	GRIN2B
15380912	kanzonols W	GRK6
15380912	kanzonols W	GSK3A
15380912	kanzonols W	GSK3B
15380912	kanzonols W	HDAC1

15380912	kanzonols W	HDAC2
15380912	kanzonols W	HDAC3
15380912	kanzonols W	HDAC6
15380912	kanzonols W	HDAC8
15380912	kanzonols W	HIF1A
15380912	kanzonols W	HPGD
15380912	kanzonols W	HPGDS
15380912	kanzonols W	HSD11B1
15380912	kanzonols W	HSP90AA1
15380912	kanzonols W	HTR3A
15380912	kanzonols W	IKBKE
15380912	kanzonols W	KAT2A
15380912	kanzonols W	KAT2B
15380912	kanzonols W	KDR
15380912	kanzonols W	MAOB
15380912	kanzonols W	MAPK1
15380912	kanzonols W	MAPT
15380912	kanzonols W	MCHR1
15380912	kanzonols W	MCL1
15380912	kanzonols W	MET
15380912	kanzonols W	METAP1
15380912	kanzonols W	MMP1
15380912	kanzonols W	MMP12
15380912	kanzonols W	MMP13
15380912	kanzonols W	MMP14
15380912	kanzonols W	MMP2
15380912	kanzonols W	MMP3
15380912	kanzonols W	MMP7
15380912	kanzonols W	MMP8
15380912	kanzonols W	MMP9
15380912	kanzonols W	MPEG1
15380912	kanzonols W	ODC1
15380912	kanzonols W	PARP1
15380912	kanzonols W	PDE4D
15380912	kanzonols W	PDK1
15380912	kanzonols W	PPARG
15380912	kanzonols W	PTK6
15380912	kanzonols W	PTPN1
15380912	kanzonols W	RAF1
15380912	kanzonols W	ROCK2
15380912	kanzonols W	S1PR1
15380912	kanzonols W	S1PR3
15380912	kanzonols W	SCD
15380912	kanzonols W	SNCA

15380912	kanzonols W	SRC
15380912	kanzonols W	SREBF2
15380912	kanzonols W	TAS2R31
15380912	kanzonols W	TBK1
15380912	kanzonols W	TBXA2R
15380912	kanzonols W	TGFBR1
15380912	kanzonols W	TNNT2
15380912	kanzonols W	TRPV1
637112	(2S)-6-(2,4-dihydroxyphenyl)-2-(2-hydroxypropan-2-yl)-4-methoxy-2,3-dihydrofuro[3,2-g]chromen-7-one	ABHD6
637112	(2S)-6-(2,4-dihydroxyphenyl)-2-(2-hydroxypropan-2-yl)-4-methoxy-2,3-dihydrofuro[3,2-g]chromen-7-one	AURKA
637112	(2S)-6-(2,4-dihydroxyphenyl)-2-(2-hydroxypropan-2-yl)-4-methoxy-2,3-dihydrofuro[3,2-g]chromen-7-one	BACE2
637112	(2S)-6-(2,4-dihydroxyphenyl)-2-(2-hydroxypropan-2-yl)-4-methoxy-2,3-dihydrofuro[3,2-g]chromen-7-one	CCNB1
637112	(2S)-6-(2,4-dihydroxyphenyl)-2-(2-hydroxypropan-2-yl)-4-methoxy-2,3-dihydrofuro[3,2-g]chromen-7-one	CCNE1
637112	(2S)-6-(2,4-dihydroxyphenyl)-2-(2-hydroxypropan-2-yl)-4-methoxy-2,3-dihydrofuro[3,2-g]chromen-7-one	CDK1
637112	(2S)-6-(2,4-dihydroxyphenyl)-2-(2-hydroxypropan-2-yl)-4-methoxy-2,3-dihydrofuro[3,2-g]chromen-7-one	CDK2
637112	(2S)-6-(2,4-dihydroxyphenyl)-2-(2-hydroxypropan-2-yl)-4-methoxy-2,3-dihydrofuro[3,2-g]chromen-7-one	CHEK1
637112	(2S)-6-(2,4-dihydroxyphenyl)-2-(2-hydroxypropan-2-yl)-4-methoxy-2,3-dihydrofuro[3,2-g]chromen-7-one	CSF1R

637112	(2S)-6-(2,4-dihydroxyphenyl)-2-(2-hydroxypropan-2-yl)-4-methoxy-2,3-dihydrofuro[3,2-g]chromen-7-one	F2
637112	(2S)-6-(2,4-dihydroxyphenyl)-2-(2-hydroxypropan-2-yl)-4-methoxy-2,3-dihydrofuro[3,2-g]chromen-7-one	FAAH
637112	(2S)-6-(2,4-dihydroxyphenyl)-2-(2-hydroxypropan-2-yl)-4-methoxy-2,3-dihydrofuro[3,2-g]chromen-7-one	GRM5
637112	(2S)-6-(2,4-dihydroxyphenyl)-2-(2-hydroxypropan-2-yl)-4-methoxy-2,3-dihydrofuro[3,2-g]chromen-7-one	HIF1A
637112	(2S)-6-(2,4-dihydroxyphenyl)-2-(2-hydroxypropan-2-yl)-4-methoxy-2,3-dihydrofuro[3,2-g]chromen-7-one	KCNH2
637112	(2S)-6-(2,4-dihydroxyphenyl)-2-(2-hydroxypropan-2-yl)-4-methoxy-2,3-dihydrofuro[3,2-g]chromen-7-one	KDR
637112	(2S)-6-(2,4-dihydroxyphenyl)-2-(2-hydroxypropan-2-yl)-4-methoxy-2,3-dihydrofuro[3,2-g]chromen-7-one	MET
637112	(2S)-6-(2,4-dihydroxyphenyl)-2-(2-hydroxypropan-2-yl)-4-methoxy-2,3-dihydrofuro[3,2-g]chromen-7-one	MGLL
637112	(2S)-6-(2,4-dihydroxyphenyl)-2-(2-hydroxypropan-2-yl)-4-methoxy-2,3-dihydrofuro[3,2-g]chromen-7-one	MMP1
637112	(2S)-6-(2,4-dihydroxyphenyl)-2-(2-hydroxypropan-2-yl)-4-methoxy-2,3-dihydrofuro[3,2-g]chromen-7-one	MMP13
637112	(2S)-6-(2,4-dihydroxyphenyl)-2-(2-hydroxypropan-2-yl)-4-methoxy-2,3-dihydrofuro[3,2-g]chromen-7-one	MMP2

637112	(2S)-6-(2,4-dihydroxyphenyl)-2-(2-hydroxypropan-2-yl)-4-methoxy-2,3-dihydrofuro[3,2-g]chromen-7-one	MMP9
637112	(2S)-6-(2,4-dihydroxyphenyl)-2-(2-hydroxypropan-2-yl)-4-methoxy-2,3-dihydrofuro[3,2-g]chromen-7-one	NR3C1
637112	(2S)-6-(2,4-dihydroxyphenyl)-2-(2-hydroxypropan-2-yl)-4-methoxy-2,3-dihydrofuro[3,2-g]chromen-7-one	RPS6KB1
637112	(2S)-6-(2,4-dihydroxyphenyl)-2-(2-hydroxypropan-2-yl)-4-methoxy-2,3-dihydrofuro[3,2-g]chromen-7-one	SCN9A
637112	(2S)-6-(2,4-dihydroxyphenyl)-2-(2-hydroxypropan-2-yl)-4-methoxy-2,3-dihydrofuro[3,2-g]chromen-7-one	SLC5A1
637112	(2S)-6-(2,4-dihydroxyphenyl)-2-(2-hydroxypropan-2-yl)-4-methoxy-2,3-dihydrofuro[3,2-g]chromen-7-one	SOAT1
637112	(2S)-6-(2,4-dihydroxyphenyl)-2-(2-hydroxypropan-2-yl)-4-methoxy-2,3-dihydrofuro[3,2-g]chromen-7-one	SOAT2
637112	(2S)-6-(2,4-dihydroxyphenyl)-2-(2-hydroxypropan-2-yl)-4-methoxy-2,3-dihydrofuro[3,2-g]chromen-7-one	TYMS
5481948	Semilicoisoflavone B	ADRB1
5481948	Semilicoisoflavone B	ADRB1
5481948	Semilicoisoflavone B	ADRB2
5481948	Semilicoisoflavone B	ADRB2
5481948	Semilicoisoflavone B	CA7
5481948	Semilicoisoflavone B	CYP19A1
5481948	Semilicoisoflavone B	CYP19A1
5481948	Semilicoisoflavone B	GCGR
5481948	Semilicoisoflavone B	HSP90AA1
5481948	Semilicoisoflavone B	HSP90AB1
5481948	Semilicoisoflavone B	PTPN1
5481948	Semilicoisoflavone B	PTPN1

5481948	Semilicoisoflavone B	RARA
5281619	Glepidotin A	ABCB1
5281619	Glepidotin A	ABCC1
5281619	Glepidotin A	ABCG2
5281619	Glepidotin A	ACHE
5281619	Glepidotin A	ADORA1
5281619	Glepidotin A	ADORA2A
5281619	Glepidotin A	AHR
5281619	Glepidotin A	AKR1B1
5281619	Glepidotin A	AKR1C1
5281619	Glepidotin A	AKR1C2
5281619	Glepidotin A	AKR1C3
5281619	Glepidotin A	AKR1C4
5281619	Glepidotin A	AKT1
5281619	Glepidotin A	ALK
5281619	Glepidotin A	ALOX12
5281619	Glepidotin A	ALOX15
5281619	Glepidotin A	ALOX5
5281619	Glepidotin A	ALPL
5281619	Glepidotin A	APEX1
5281619	Glepidotin A	ARG1
5281619	Glepidotin A	AVPR2
5281619	Glepidotin A	BACE1
5281619	Glepidotin A	BCHE
5281619	Glepidotin A	BCL2
5281619	Glepidotin A	CA1
5281619	Glepidotin A	CA12
5281619	Glepidotin A	CA13
5281619	Glepidotin A	CA2
5281619	Glepidotin A	CA3
5281619	Glepidotin A	CA4
5281619	Glepidotin A	CA5A
5281619	Glepidotin A	CA7
5281619	Glepidotin A	CA9
5281619	Glepidotin A	CBR1
5281619	Glepidotin A	CCND1
5281619	Glepidotin A	CDK1
5281619	Glepidotin A	CDK2
5281619	Glepidotin A	CDK4
5281619	Glepidotin A	CFTR
5281619	Glepidotin A	CNR2
5281619	Glepidotin A	CSNK2A1
5281619	Glepidotin A	CXCR1
5281619	Glepidotin A	CYP19A1

5281619	Glepidotin A	CYP1B1
5281619	Glepidotin A	DAPK1
5281619	Glepidotin A	DHFR
5281619	Glepidotin A	DRD4
5281619	Glepidotin A	EGFR
5281619	Glepidotin A	EGLN1
5281619	Glepidotin A	EP300
5281619	Glepidotin A	ERBB2
5281619	Glepidotin A	ESR1
5281619	Glepidotin A	ESR2
5281619	Glepidotin A	ESRRA
5281619	Glepidotin A	F2
5281619	Glepidotin A	GABRA1
5281619	Glepidotin A	GABRA3
5281619	Glepidotin A	GABRB3
5281619	Glepidotin A	GABRB3
5281619	Glepidotin A	GABRG2
5281619	Glepidotin A	GABRG2
5281619	Glepidotin A	GCGR
5281619	Glepidotin A	GPR35
5281619	Glepidotin A	GPR84
5281619	Glepidotin A	GSK3B
5281619	Glepidotin A	HSD17B1
5281619	Glepidotin A	HSD17B2
5281619	Glepidotin A	HSP90AA1
5281619	Glepidotin A	HSP90AB1
5281619	Glepidotin A	HSP90B1
5281619	Glepidotin A	KDM4E
5281619	Glepidotin A	MAOA
5281619	Glepidotin A	MAPT
5281619	Glepidotin A	MCL1
5281619	Glepidotin A	MMP13
5281619	Glepidotin A	MMP2
5281619	Glepidotin A	MMP3
5281619	Glepidotin A	MMP9
5281619	Glepidotin A	MPG
5281619	Glepidotin A	MPO
5281619	Glepidotin A	MYLK
5281619	Glepidotin A	NAE1
5281619	Glepidotin A	NEK2
5281619	Glepidotin A	NEK6
5281619	Glepidotin A	NOX4
5281619	Glepidotin A	PDE10A
5281619	Glepidotin A	PDE5A

5281619	Glepidotin A	PDK1
5281619	Glepidotin A	PFKFB3
5281619	Glepidotin A	PIK3R1
5281619	Glepidotin A	PLA2G1B
5281619	Glepidotin A	PLAU
5281619	Glepidotin A	PLK1
5281619	Glepidotin A	PTAFR
5281619	Glepidotin A	PTGS2
5281619	Glepidotin A	PTPN1
5281619	Glepidotin A	PTPRS
5281619	Glepidotin A	PYGL
5281619	Glepidotin A	SYK
5281619	Glepidotin A	TOP2A
5281619	Glepidotin A	TYR
5281619	Glepidotin A	XDH
162412	Phaseolinisoflavan	ABL1
162412	Phaseolinisoflavan	ADORA1
162412	Phaseolinisoflavan	ADORA2A
162412	Phaseolinisoflavan	ADORA2B
162412	Phaseolinisoflavan	ALDH2
162412	Phaseolinisoflavan	ALK
162412	Phaseolinisoflavan	ALPL
162412	Phaseolinisoflavan	AR
162412	Phaseolinisoflavan	AURKA
162412	Phaseolinisoflavan	BLK
162412	Phaseolinisoflavan	CA13
162412	Phaseolinisoflavan	CA7
162412	Phaseolinisoflavan	CASK
162412	Phaseolinisoflavan	CCNB1
162412	Phaseolinisoflavan	CCNB2
162412	Phaseolinisoflavan	CCNB3
162412	Phaseolinisoflavan	CCND1
162412	Phaseolinisoflavan	CCND2
162412	Phaseolinisoflavan	CCND3
162412	Phaseolinisoflavan	CCR4
162412	Phaseolinisoflavan	CDK1
162412	Phaseolinisoflavan	CDK4
162412	Phaseolinisoflavan	CDK5
162412	Phaseolinisoflavan	CDK5R1
162412	Phaseolinisoflavan	CDK7
162412	Phaseolinisoflavan	CHEK1
162412	Phaseolinisoflavan	CHEK2
162412	Phaseolinisoflavan	CSF1R
162412	Phaseolinisoflavan	CSK

162412	Phaseolinisoflavan	CSNK1A1
162412	Phaseolinisoflavan	CSNK1D
162412	Phaseolinisoflavan	CSNK1G1
162412	Phaseolinisoflavan	CSNK1G2
162412	Phaseolinisoflavan	DAPK1
162412	Phaseolinisoflavan	DAPK2
162412	Phaseolinisoflavan	DAPK3
162412	Phaseolinisoflavan	DRD4
162412	Phaseolinisoflavan	DSTYK
162412	Phaseolinisoflavan	EGFR
162412	Phaseolinisoflavan	ELANE
162412	Phaseolinisoflavan	EP300
162412	Phaseolinisoflavan	EPHA2
162412	Phaseolinisoflavan	ERBB2
162412	Phaseolinisoflavan	ERN1
162412	Phaseolinisoflavan	ESR1
162412	Phaseolinisoflavan	ESR1
162412	Phaseolinisoflavan	ESR2
162412	Phaseolinisoflavan	ESR2
162412	Phaseolinisoflavan	FGFR1
162412	Phaseolinisoflavan	FGFR3
162412	Phaseolinisoflavan	FLT1
162412	Phaseolinisoflavan	FLT3
162412	Phaseolinisoflavan	FLT4
162412	Phaseolinisoflavan	FNTA
162412	Phaseolinisoflavan	FNTB
162412	Phaseolinisoflavan	FYN
162412	Phaseolinisoflavan	GABRA1
162412	Phaseolinisoflavan	GABRA3
162412	Phaseolinisoflavan	GABRA5
162412	Phaseolinisoflavan	GABRA6
162412	Phaseolinisoflavan	GABRB3
162412	Phaseolinisoflavan	GABRB3
162412	Phaseolinisoflavan	GABRB3
162412	Phaseolinisoflavan	GABRB3
162412	Phaseolinisoflavan	GABRG2
162412	Phaseolinisoflavan	GABRG2
162412	Phaseolinisoflavan	GABRG2
162412	Phaseolinisoflavan	GABRG2
162412	Phaseolinisoflavan	GCGR
162412	Phaseolinisoflavan	GPR84
162412	Phaseolinisoflavan	GRK7
162412	Phaseolinisoflavan	GRM4
162412	Phaseolinisoflavan	GSK3A

162412	Phaseolinisoflavan	HIPK4
162412	Phaseolinisoflavan	HSD17B1
162412	Phaseolinisoflavan	HSD17B2
162412	Phaseolinisoflavan	HSD17B3
162412	Phaseolinisoflavan	HSP90AA1
162412	Phaseolinisoflavan	HSP90AB1
162412	Phaseolinisoflavan	HSP90B1
162412	Phaseolinisoflavan	JAK1
162412	Phaseolinisoflavan	JAK2
162412	Phaseolinisoflavan	JAK3
162412	Phaseolinisoflavan	KDM1A
162412	Phaseolinisoflavan	KDR
162412	Phaseolinisoflavan	KIT
162412	Phaseolinisoflavan	LCK
162412	Phaseolinisoflavan	LNPEP
162412	Phaseolinisoflavan	LRRK2
162412	Phaseolinisoflavan	LTA4H
162412	Phaseolinisoflavan	MAP2K2
162412	Phaseolinisoflavan	MAP2K3
162412	Phaseolinisoflavan	MAP2K4
162412	Phaseolinisoflavan	MAP3K9
162412	Phaseolinisoflavan	MAPK14
162412	Phaseolinisoflavan	MET
162412	Phaseolinisoflavan	MMP1
162412	Phaseolinisoflavan	MTOR
162412	Phaseolinisoflavan	MYLK
162412	Phaseolinisoflavan	NTRK1
162412	Phaseolinisoflavan	PDE10A
162412	Phaseolinisoflavan	PDE5A
162412	Phaseolinisoflavan	PDE5A
162412	Phaseolinisoflavan	PDGFRA
162412	Phaseolinisoflavan	PDGFRB
162412	Phaseolinisoflavan	PHKG2
162412	Phaseolinisoflavan	PIK3C2G
162412	Phaseolinisoflavan	PIK3CB
162412	Phaseolinisoflavan	PIK3CD
162412	Phaseolinisoflavan	PIM1
162412	Phaseolinisoflavan	PIP4K2C
162412	Phaseolinisoflavan	PIP5K1C
162412	Phaseolinisoflavan	PLK1
162412	Phaseolinisoflavan	PRKDC
162412	Phaseolinisoflavan	PRKG2
162412	Phaseolinisoflavan	PTPN1
162412	Phaseolinisoflavan	PTPN1

162412	Phaseolinisoflavan	RELA
162412	Phaseolinisoflavan	RET
162412	Phaseolinisoflavan	RORC
162412	Phaseolinisoflavan	RPS6KA1
162412	Phaseolinisoflavan	RPS6KA2
162412	Phaseolinisoflavan	RPS6KA3
162412	Phaseolinisoflavan	RPS6KA4
162412	Phaseolinisoflavan	SRC
162412	Phaseolinisoflavan	SRD5A1
162412	Phaseolinisoflavan	SYK
162412	Phaseolinisoflavan	TAOK2
162412	Phaseolinisoflavan	TLR9
162412	Phaseolinisoflavan	TRPM8
162412	Phaseolinisoflavan	VCP
162412	Phaseolinisoflavan	WEE1
162412	Phaseolinisoflavan	XPO1
162412	Phaseolinisoflavan	YES1
5317768	Glypallichalcone	ABCB1
5317768	Glypallichalcone	ABCG2
5317768	Glypallichalcone	ACHE
5317768	Glypallichalcone	ADAM10
5317768	Glypallichalcone	ADAM17
5317768	Glypallichalcone	ADAM9
5317768	Glypallichalcone	AKR1B1
5317768	Glypallichalcone	ALDH2
5317768	Glypallichalcone	ALOX15
5317768	Glypallichalcone	ALOX5
5317768	Glypallichalcone	APP
5317768	Glypallichalcone	BACE1
5317768	Glypallichalcone	BCHE
5317768	Glypallichalcone	BMP1
5317768	Glypallichalcone	BRAF
5317768	Glypallichalcone	CCNA1
5317768	Glypallichalcone	CCNA2
5317768	Glypallichalcone	CCNC
5317768	Glypallichalcone	CCND1
5317768	Glypallichalcone	CCNE1
5317768	Glypallichalcone	CCNE2
5317768	Glypallichalcone	CDC25A
5317768	Glypallichalcone	CDC25B
5317768	Glypallichalcone	CDK2
5317768	Glypallichalcone	CDK2
5317768	Glypallichalcone	CDK2
5317768	Glypallichalcone	CDK4

5317768	Glypallichalcone	CDK8
5317768	Glypallichalcone	CDK8
5317768	Glypallichalcone	CHEK1
5317768	Glypallichalcone	CHRNA7
5317768	Glypallichalcone	CTSS
5317768	Glypallichalcone	CXCR4
5317768	Glypallichalcone	CYP19A1
5317768	Glypallichalcone	DUSP3
5317768	Glypallichalcone	EDNRA
5317768	Glypallichalcone	EGFR
5317768	Glypallichalcone	EZR
5317768	Glypallichalcone	F3
5317768	Glypallichalcone	FCER2
5317768	Glypallichalcone	GRIA1
5317768	Glypallichalcone	GSK3A
5317768	Glypallichalcone	GSTM2
5317768	Glypallichalcone	GSTP1
5317768	Glypallichalcone	HDAC1
5317768	Glypallichalcone	HDAC3
5317768	Glypallichalcone	HDAC5
5317768	Glypallichalcone	HDAC6
5317768	Glypallichalcone	HDAC7
5317768	Glypallichalcone	HDAC8
5317768	Glypallichalcone	HDAC9
5317768	Glypallichalcone	HPGDS
5317768	Glypallichalcone	HTR1A
5317768	Glypallichalcone	KCNA3
5317768	Glypallichalcone	KIT
5317768	Glypallichalcone	LNPEP
5317768	Glypallichalcone	MAOA
5317768	Glypallichalcone	MAOB
5317768	Glypallichalcone	MAP2K1
5317768	Glypallichalcone	MAPK14
5317768	Glypallichalcone	MELK
5317768	Glypallichalcone	MIF
5317768	Glypallichalcone	MMP1
5317768	Glypallichalcone	MMP12
5317768	Glypallichalcone	MMP13
5317768	Glypallichalcone	MMP14
5317768	Glypallichalcone	MMP16
5317768	Glypallichalcone	MMP2
5317768	Glypallichalcone	MMP25
5317768	Glypallichalcone	MMP3
5317768	Glypallichalcone	MMP7

5317768	Glypallichalcone	MMP8
5317768	Glypallichalcone	MMP9
5317768	Glypallichalcone	MPEG1
5317768	Glypallichalcone	NOS2
5317768	Glypallichalcone	NOX4
5317768	Glypallichalcone	NR1D1
5317768	Glypallichalcone	PDE4A
5317768	Glypallichalcone	PDE4B
5317768	Glypallichalcone	PDE4C
5317768	Glypallichalcone	PDE4D
5317768	Glypallichalcone	PDK1
5317768	Glypallichalcone	PDPK1
5317768	Glypallichalcone	PFKFB3
5317768	Glypallichalcone	PIK3CA
5317768	Glypallichalcone	PIK3CB
5317768	Glypallichalcone	PIK3CD
5317768	Glypallichalcone	PIK3R1
5317768	Glypallichalcone	PIM1
5317768	Glypallichalcone	PIM2
5317768	Glypallichalcone	PLK1
5317768	Glypallichalcone	PNMT
5317768	Glypallichalcone	PPARG
5317768	Glypallichalcone	PTGS1
5317768	Glypallichalcone	PTGS2
5317768	Glypallichalcone	PTPN1
5317768	Glypallichalcone	RAF1
5317768	Glypallichalcone	RET
5317768	Glypallichalcone	ROCK1
5317768	Glypallichalcone	ROCK2
5317768	Glypallichalcone	SLC1A3
5317768	Glypallichalcone	SNCA
5317768	Glypallichalcone	TERT
5317768	Glypallichalcone	TLR9
5317768	Glypallichalcone	TUBB1
5317768	Glypallichalcone	VCP
5317768	Glypallichalcone	WEE1
10542808	8-(6-hydroxy-2-benzofuranyl)-2,2-dimethyl-5-chromenol	ADRA2A
10542808	8-(6-hydroxy-2-benzofuranyl)-2,2-dimethyl-5-chromenol	BACE1
10542808	8-(6-hydroxy-2-benzofuranyl)-2,2-dimethyl-5-chromenol	BMP1
10542808	8-(6-hydroxy-2-benzofuranyl)-2,2-dimethyl-5-chromenol	BRAF

10542808	8-(6-hydroxy-2-benzofuranyl)-2,2-dimethyl-5-chromenol	EDNRA
10542808	8-(6-hydroxy-2-benzofuranyl)-2,2-dimethyl-5-chromenol	EIF2AK3
10542808	8-(6-hydroxy-2-benzofuranyl)-2,2-dimethyl-5-chromenol	ESR1
10542808	8-(6-hydroxy-2-benzofuranyl)-2,2-dimethyl-5-chromenol	GSK3B
10542808	8-(6-hydroxy-2-benzofuranyl)-2,2-dimethyl-5-chromenol	HSD17B1
10542808	8-(6-hydroxy-2-benzofuranyl)-2,2-dimethyl-5-chromenol	HSD17B2
10542808	8-(6-hydroxy-2-benzofuranyl)-2,2-dimethyl-5-chromenol	HSP90AA1
10542808	8-(6-hydroxy-2-benzofuranyl)-2,2-dimethyl-5-chromenol	HSP90AB1
10542808	8-(6-hydroxy-2-benzofuranyl)-2,2-dimethyl-5-chromenol	LRRK2
10542808	8-(6-hydroxy-2-benzofuranyl)-2,2-dimethyl-5-chromenol	MAP3K12
10542808	8-(6-hydroxy-2-benzofuranyl)-2,2-dimethyl-5-chromenol	MAPK1
10542808	8-(6-hydroxy-2-benzofuranyl)-2,2-dimethyl-5-chromenol	MTOR
10542808	8-(6-hydroxy-2-benzofuranyl)-2,2-dimethyl-5-chromenol	OPRD1
10542808	8-(6-hydroxy-2-benzofuranyl)-2,2-dimethyl-5-chromenol	PIK3CA
5318999	Licochalcone B	ABCB1
5318999	Licochalcone B	ABCC1
5318999	Licochalcone B	ABCG2
5318999	Licochalcone B	ABL1
5318999	Licochalcone B	ADAM17
5318999	Licochalcone B	ADCY5
5318999	Licochalcone B	AKR1B1
5318999	Licochalcone B	ALOX5
5318999	Licochalcone B	APP
5318999	Licochalcone B	BACE1
5318999	Licochalcone B	BCL2
5318999	Licochalcone B	BCL2L1
5318999	Licochalcone B	BRAF
5318999	Licochalcone B	CALM1
5318999	Licochalcone B	CAPN1
5318999	Licochalcone B	CBR1

5318999	Licochalcone B	CCNB1
5318999	Licochalcone B	CCNE1
5318999	Licochalcone B	CCNE1
5318999	Licochalcone B	CDK1
5318999	Licochalcone B	CDK2
5318999	Licochalcone B	CDK3
5318999	Licochalcone B	CDK4
5318999	Licochalcone B	CHEK1
5318999	Licochalcone B	CHRNA7
5318999	Licochalcone B	CLK1
5318999	Licochalcone B	CXCR4
5318999	Licochalcone B	CYP19A1
5318999	Licochalcone B	CYP1B1
5318999	Licochalcone B	DUSP3
5318999	Licochalcone B	EGFR
5318999	Licochalcone B	ERN1
5318999	Licochalcone B	ESR1
5318999	Licochalcone B	ESR2
5318999	Licochalcone B	ESRRA
5318999	Licochalcone B	ESRRB
5318999	Licochalcone B	F3
5318999	Licochalcone B	FLT3
5318999	Licochalcone B	GLI1
5318999	Licochalcone B	GLI2
5318999	Licochalcone B	GRK2
5318999	Licochalcone B	HDAC4
5318999	Licochalcone B	HDAC8
5318999	Licochalcone B	HPGDS
5318999	Licochalcone B	HSD17B14
5318999	Licochalcone B	IGF1R
5318999	Licochalcone B	INSR
5318999	Licochalcone B	KCNA3
5318999	Licochalcone B	MAOA
5318999	Licochalcone B	MAOB
5318999	Licochalcone B	MAP4K4
5318999	Licochalcone B	MAPT
5318999	Licochalcone B	MMP2
5318999	Licochalcone B	MMP3
5318999	Licochalcone B	MMP8
5318999	Licochalcone B	MTOR
5318999	Licochalcone B	NOS2
5318999	Licochalcone B	NOX4
5318999	Licochalcone B	NQO2
5318999	Licochalcone B	ODC1

5318999	Licochalcone B	PDGFRB
5318999	Licochalcone B	PDK1
5318999	Licochalcone B	PDPK1
5318999	Licochalcone B	PLAU
5318999	Licochalcone B	PLK1
5318999	Licochalcone B	PTGS1
5318999	Licochalcone B	PTGS2
5318999	Licochalcone B	PTPN1
5318999	Licochalcone B	RAF1
5318999	Licochalcone B	RPS6KB1
5318999	Licochalcone B	SHBG
5318999	Licochalcone B	SIGMAR1
5318999	Licochalcone B	SLC9A1
5318999	Licochalcone B	SNCA
5318999	Licochalcone B	TERT
5318999	Licochalcone B	THRA
5318999	Licochalcone B	THRB
5318999	Licochalcone B	TUBB1
5318999	Licochalcone B	TYR
5318999	Licochalcone B	VCP
5318999	Licochalcone B	WEE1
5318999	Licochalcone B	YWHAG
49856081	licochalcone G	ABCB1
49856081	licochalcone G	ABCG2
49856081	licochalcone G	ABL1
49856081	licochalcone G	ACHE
49856081	licochalcone G	ADAM17
49856081	licochalcone G	ADORA1
49856081	licochalcone G	AKR1B1
49856081	licochalcone G	ALDH2
49856081	licochalcone G	ALOX5
49856081	licochalcone G	APP
49856081	licochalcone G	BACE1
49856081	licochalcone G	BCHE
49856081	licochalcone G	BMP1
49856081	licochalcone G	BRAF
49856081	licochalcone G	CA6
49856081	licochalcone G	CCND1
49856081	licochalcone G	CCNE1
49856081	licochalcone G	CCNE2
49856081	licochalcone G	CDC25A
49856081	licochalcone G	CDK2
49856081	licochalcone G	CDK4
49856081	licochalcone G	CHRNA7

49856081	licochalcone G	CSF1R
49856081	licochalcone G	CTSD
49856081	licochalcone G	CYP19A1
49856081	licochalcone G	ECE1
49856081	licochalcone G	EGFR
49856081	licochalcone G	ESRRB
49856081	licochalcone G	F10
49856081	licochalcone G	F3
49856081	licochalcone G	FLT3
49856081	licochalcone G	GNRHR
49856081	licochalcone G	HDAC1
49856081	licochalcone G	HDAC10
49856081	licochalcone G	HDAC3
49856081	licochalcone G	HDAC4
49856081	licochalcone G	HDAC5
49856081	licochalcone G	HDAC6
49856081	licochalcone G	HDAC7
49856081	licochalcone G	HDAC8
49856081	licochalcone G	HDAC9
49856081	licochalcone G	HPGDS
49856081	licochalcone G	HSP90AB1
49856081	licochalcone G	ITK
49856081	licochalcone G	JAK2
49856081	licochalcone G	KCNA3
49856081	licochalcone G	KDM1A
49856081	licochalcone G	MAOA
49856081	licochalcone G	MAOB
49856081	licochalcone G	MAPK14
49856081	licochalcone G	MAPKAPK2
49856081	licochalcone G	MAPKAPK5
49856081	licochalcone G	MMP1
49856081	licochalcone G	MMP13
49856081	licochalcone G	MMP14
49856081	licochalcone G	MMP16
49856081	licochalcone G	MMP8
49856081	licochalcone G	MMP9
49856081	licochalcone G	NAAA
49856081	licochalcone G	NOS2
49856081	licochalcone G	NR1H4
49856081	licochalcone G	ODC1
49856081	licochalcone G	PDE4B
49856081	licochalcone G	PDGFRB
49856081	licochalcone G	PDK1
49856081	licochalcone G	PDPK1

49856081	licochalcone G	PIM1
49856081	licochalcone G	PIM2
49856081	licochalcone G	PIM3
49856081	licochalcone G	PLA2G1B
49856081	licochalcone G	PLA2G2A
49856081	licochalcone G	PRKCB
49856081	licochalcone G	PTGS1
49856081	licochalcone G	PTGS2
49856081	licochalcone G	PTPN1
49856081	licochalcone G	PTPN11
49856081	licochalcone G	PTPN6
49856081	licochalcone G	RAF1
49856081	licochalcone G	RET
49856081	licochalcone G	RPS6KB1
49856081	licochalcone G	SLC29A1
49856081	licochalcone G	SLC5A1
49856081	licochalcone G	SLC5A2
49856081	licochalcone G	SSTR2
49856081	licochalcone G	TERT
49856081	licochalcone G	TGFBR1
49856081	licochalcone G	THRA
49856081	licochalcone G	THRB
49856081	licochalcone G	TKT
49856081	licochalcone G	TNF
49856081	licochalcone G	TRPM8
49856081	licochalcone G	TYR
5320083	Glycyrol	ALOX12
5320083	Glycyrol	ALOX15
5320083	Glycyrol	CA13
5320083	Glycyrol	CCR4
5320083	Glycyrol	CFTR
5320083	Glycyrol	CXCR2
5320083	Glycyrol	EGLN1
5320083	Glycyrol	EP300
5320083	Glycyrol	ESR1
5320083	Glycyrol	ESR2
5320083	Glycyrol	FNTA
5320083	Glycyrol	FNTB
5320083	Glycyrol	GCGR
5320083	Glycyrol	GPR84
5320083	Glycyrol	HSD17B1
5320083	Glycyrol	HSP90AA1
5320083	Glycyrol	HSP90AB1
5320083	Glycyrol	HSP90B1

5320083	Glycyrol	IDH1
5320083	Glycyrol	MPI
5320083	Glycyrol	NFKB1
5320083	Glycyrol	NOS2
5320083	Glycyrol	NOX4
5320083	Glycyrol	PDE10A
5320083	Glycyrol	PDE4D
5320083	Glycyrol	PLAU
5320083	Glycyrol	ROCK2
5320083	Glycyrol	SRD5A1
5320083	Glycyrol	XPO1
10090416	3-(2,4-dihydroxyphenyl)-8-(1,1-dimethylprop-2-enyl)-7-hydroxy-5-methoxy-coumarin	ABL1
10090416	3-(2,4-dihydroxyphenyl)-8-(1,1-dimethylprop-2-enyl)-7-hydroxy-5-methoxy-coumarin	ACHE
10090416	3-(2,4-dihydroxyphenyl)-8-(1,1-dimethylprop-2-enyl)-7-hydroxy-5-methoxy-coumarin	ADORA2B
10090416	3-(2,4-dihydroxyphenyl)-8-(1,1-dimethylprop-2-enyl)-7-hydroxy-5-methoxy-coumarin	AKR1B10
10090416	3-(2,4-dihydroxyphenyl)-8-(1,1-dimethylprop-2-enyl)-7-hydroxy-5-methoxy-coumarin	AURKA
10090416	3-(2,4-dihydroxyphenyl)-8-(1,1-dimethylprop-2-enyl)-7-hydroxy-5-methoxy-coumarin	AURKB
10090416	3-(2,4-dihydroxyphenyl)-8-(1,1-dimethylprop-2-enyl)-7-hydroxy-5-methoxy-coumarin	BMP1
10090416	3-(2,4-dihydroxyphenyl)-8-(1,1-dimethylprop-2-enyl)-7-hydroxy-5-methoxy-coumarin	CA1
10090416	3-(2,4-dihydroxyphenyl)-8-(1,1-dimethylprop-2-enyl)-7-hydroxy-5-methoxy-coumarin	CA2
10090416	3-(2,4-dihydroxyphenyl)-8-(1,1-dimethylprop-2-enyl)-7-hydroxy-5-methoxy-coumarin	CDC25A
10090416	3-(2,4-dihydroxyphenyl)-8-(1,1-dimethylprop-2-enyl)-7-hydroxy-5-methoxy-coumarin	CDC25B

10090416	3-(2,4-dihydroxyphenyl)-8-(1,1-dimethylprop-2-enyl)-7-hydroxy-5-methoxy-coumarin	CTSD
10090416	3-(2,4-dihydroxyphenyl)-8-(1,1-dimethylprop-2-enyl)-7-hydroxy-5-methoxy-coumarin	CYP19A1
10090416	3-(2,4-dihydroxyphenyl)-8-(1,1-dimethylprop-2-enyl)-7-hydroxy-5-methoxy-coumarin	CYP2C19
10090416	3-(2,4-dihydroxyphenyl)-8-(1,1-dimethylprop-2-enyl)-7-hydroxy-5-methoxy-coumarin	CYP3A4
10090416	3-(2,4-dihydroxyphenyl)-8-(1,1-dimethylprop-2-enyl)-7-hydroxy-5-methoxy-coumarin	DNM1
10090416	3-(2,4-dihydroxyphenyl)-8-(1,1-dimethylprop-2-enyl)-7-hydroxy-5-methoxy-coumarin	EP300
10090416	3-(2,4-dihydroxyphenyl)-8-(1,1-dimethylprop-2-enyl)-7-hydroxy-5-methoxy-coumarin	ESR1
10090416	3-(2,4-dihydroxyphenyl)-8-(1,1-dimethylprop-2-enyl)-7-hydroxy-5-methoxy-coumarin	ESR2
10090416	3-(2,4-dihydroxyphenyl)-8-(1,1-dimethylprop-2-enyl)-7-hydroxy-5-methoxy-coumarin	F10
10090416	3-(2,4-dihydroxyphenyl)-8-(1,1-dimethylprop-2-enyl)-7-hydroxy-5-methoxy-coumarin	FLT1
10090416	3-(2,4-dihydroxyphenyl)-8-(1,1-dimethylprop-2-enyl)-7-hydroxy-5-methoxy-coumarin	FLT3
10090416	3-(2,4-dihydroxyphenyl)-8-(1,1-dimethylprop-2-enyl)-7-hydroxy-5-methoxy-coumarin	GNRHR
10090416	3-(2,4-dihydroxyphenyl)-8-(1,1-dimethylprop-2-enyl)-7-hydroxy-5-methoxy-coumarin	HDAC2
10090416	3-(2,4-dihydroxyphenyl)-8-(1,1-dimethylprop-2-enyl)-7-hydroxy-5-methoxy-coumarin	HDAC3

10090416	3-(2,4-dihydroxyphenyl)-8-(1,1-dimethylprop-2-enyl)-7-hydroxy-5-methoxy-coumarin	HSD17B1
10090416	3-(2,4-dihydroxyphenyl)-8-(1,1-dimethylprop-2-enyl)-7-hydroxy-5-methoxy-coumarin	HSD17B2
10090416	3-(2,4-dihydroxyphenyl)-8-(1,1-dimethylprop-2-enyl)-7-hydroxy-5-methoxy-coumarin	HSP90AA1
10090416	3-(2,4-dihydroxyphenyl)-8-(1,1-dimethylprop-2-enyl)-7-hydroxy-5-methoxy-coumarin	KDR
10090416	3-(2,4-dihydroxyphenyl)-8-(1,1-dimethylprop-2-enyl)-7-hydroxy-5-methoxy-coumarin	MAOB
10090416	3-(2,4-dihydroxyphenyl)-8-(1,1-dimethylprop-2-enyl)-7-hydroxy-5-methoxy-coumarin	MAPK14
10090416	3-(2,4-dihydroxyphenyl)-8-(1,1-dimethylprop-2-enyl)-7-hydroxy-5-methoxy-coumarin	MDM2
10090416	3-(2,4-dihydroxyphenyl)-8-(1,1-dimethylprop-2-enyl)-7-hydroxy-5-methoxy-coumarin	MDM4
10090416	3-(2,4-dihydroxyphenyl)-8-(1,1-dimethylprop-2-enyl)-7-hydroxy-5-methoxy-coumarin	MET
10090416	3-(2,4-dihydroxyphenyl)-8-(1,1-dimethylprop-2-enyl)-7-hydroxy-5-methoxy-coumarin	MMP1
10090416	3-(2,4-dihydroxyphenyl)-8-(1,1-dimethylprop-2-enyl)-7-hydroxy-5-methoxy-coumarin	MMP13
10090416	3-(2,4-dihydroxyphenyl)-8-(1,1-dimethylprop-2-enyl)-7-hydroxy-5-methoxy-coumarin	NPY5R
10090416	3-(2,4-dihydroxyphenyl)-8-(1,1-dimethylprop-2-enyl)-7-hydroxy-5-methoxy-coumarin	NR1H4
10090416	3-(2,4-dihydroxyphenyl)-8-(1,1-dimethylprop-2-enyl)-7-hydroxy-5-methoxy-coumarin	OPRM1

10090416	3-(2,4-dihydroxyphenyl)-8-(1,1-dimethylprop-2-enyl)-7-hydroxy-5-methoxy-coumarin	P2RX3
10090416	3-(2,4-dihydroxyphenyl)-8-(1,1-dimethylprop-2-enyl)-7-hydroxy-5-methoxy-coumarin	PDE10A
10090416	3-(2,4-dihydroxyphenyl)-8-(1,1-dimethylprop-2-enyl)-7-hydroxy-5-methoxy-coumarin	PDE4D
10090416	3-(2,4-dihydroxyphenyl)-8-(1,1-dimethylprop-2-enyl)-7-hydroxy-5-methoxy-coumarin	PDGFRA
10090416	3-(2,4-dihydroxyphenyl)-8-(1,1-dimethylprop-2-enyl)-7-hydroxy-5-methoxy-coumarin	PDGFRB
10090416	3-(2,4-dihydroxyphenyl)-8-(1,1-dimethylprop-2-enyl)-7-hydroxy-5-methoxy-coumarin	PDGFRB
10090416	3-(2,4-dihydroxyphenyl)-8-(1,1-dimethylprop-2-enyl)-7-hydroxy-5-methoxy-coumarin	PDK1
10090416	3-(2,4-dihydroxyphenyl)-8-(1,1-dimethylprop-2-enyl)-7-hydroxy-5-methoxy-coumarin	PDPK1
10090416	3-(2,4-dihydroxyphenyl)-8-(1,1-dimethylprop-2-enyl)-7-hydroxy-5-methoxy-coumarin	PIM1
10090416	3-(2,4-dihydroxyphenyl)-8-(1,1-dimethylprop-2-enyl)-7-hydroxy-5-methoxy-coumarin	PIM2
10090416	3-(2,4-dihydroxyphenyl)-8-(1,1-dimethylprop-2-enyl)-7-hydroxy-5-methoxy-coumarin	PIM3
10090416	3-(2,4-dihydroxyphenyl)-8-(1,1-dimethylprop-2-enyl)-7-hydroxy-5-methoxy-coumarin	PLK1
10090416	3-(2,4-dihydroxyphenyl)-8-(1,1-dimethylprop-2-enyl)-7-hydroxy-5-methoxy-coumarin	PTGIR
10090416	3-(2,4-dihydroxyphenyl)-8-(1,1-dimethylprop-2-enyl)-7-hydroxy-5-methoxy-coumarin	PTGS1

10090416	3-(2,4-dihydroxyphenyl)-8-(1,1-dimethylprop-2-enyl)-7-hydroxy-5-methoxy-coumarin	PTPN1
10090416	3-(2,4-dihydroxyphenyl)-8-(1,1-dimethylprop-2-enyl)-7-hydroxy-5-methoxy-coumarin	PTPN11
10090416	3-(2,4-dihydroxyphenyl)-8-(1,1-dimethylprop-2-enyl)-7-hydroxy-5-methoxy-coumarin	PTPN6
10090416	3-(2,4-dihydroxyphenyl)-8-(1,1-dimethylprop-2-enyl)-7-hydroxy-5-methoxy-coumarin	RET
10090416	3-(2,4-dihydroxyphenyl)-8-(1,1-dimethylprop-2-enyl)-7-hydroxy-5-methoxy-coumarin	RPS6KB1
10090416	3-(2,4-dihydroxyphenyl)-8-(1,1-dimethylprop-2-enyl)-7-hydroxy-5-methoxy-coumarin	SLC29A1
10090416	3-(2,4-dihydroxyphenyl)-8-(1,1-dimethylprop-2-enyl)-7-hydroxy-5-methoxy-coumarin	SLC5A1
10090416	3-(2,4-dihydroxyphenyl)-8-(1,1-dimethylprop-2-enyl)-7-hydroxy-5-methoxy-coumarin	SLC5A2
10090416	3-(2,4-dihydroxyphenyl)-8-(1,1-dimethylprop-2-enyl)-7-hydroxy-5-methoxy-coumarin	SRC
10090416	3-(2,4-dihydroxyphenyl)-8-(1,1-dimethylprop-2-enyl)-7-hydroxy-5-methoxy-coumarin	TAS2R31
10090416	3-(2,4-dihydroxyphenyl)-8-(1,1-dimethylprop-2-enyl)-7-hydroxy-5-methoxy-coumarin	TGFBR1
10090416	3-(2,4-dihydroxyphenyl)-8-(1,1-dimethylprop-2-enyl)-7-hydroxy-5-methoxy-coumarin	TGM2
10090416	3-(2,4-dihydroxyphenyl)-8-(1,1-dimethylprop-2-enyl)-7-hydroxy-5-methoxy-coumarin	THRA
10090416	3-(2,4-dihydroxyphenyl)-8-(1,1-dimethylprop-2-enyl)-7-hydroxy-5-methoxy-coumarin	THRB

10090416	3-(2,4-dihydroxyphenyl)-8-(1,1-dimethylprop-2-enyl)-7-hydroxy-5-methoxy-coumarin	TKT
10090416	3-(2,4-dihydroxyphenyl)-8-(1,1-dimethylprop-2-enyl)-7-hydroxy-5-methoxy-coumarin	TRPM8
10090416	3-(2,4-dihydroxyphenyl)-8-(1,1-dimethylprop-2-enyl)-7-hydroxy-5-methoxy-coumarin	TRPV1
10090416	3-(2,4-dihydroxyphenyl)-8-(1,1-dimethylprop-2-enyl)-7-hydroxy-5-methoxy-coumarin	XPO1
5319013	Licoricone	ABCB1
5319013	Licoricone	ACHE
5319013	Licoricone	ACP1
5319013	Licoricone	ADRB1
5319013	Licoricone	ADRB2
5319013	Licoricone	AGTR1
5319013	Licoricone	ALDH2
5319013	Licoricone	CA4
5319013	Licoricone	CBR1
5319013	Licoricone	CCND1
5319013	Licoricone	CDK1
5319013	Licoricone	CDK2
5319013	Licoricone	CDK4
5319013	Licoricone	CHEK2
5319013	Licoricone	CNOT7
5319013	Licoricone	CYP19A1
5319013	Licoricone	F10
5319013	Licoricone	GCGR
5319013	Licoricone	HSP90AA1
5319013	Licoricone	HSP90AB1
5319013	Licoricone	HSP90B1
5319013	Licoricone	IL2
5319013	Licoricone	MELK
5319013	Licoricone	MTOR
5319013	Licoricone	OPRK1
5319013	Licoricone	PDK1
5319013	Licoricone	PIM1
5319013	Licoricone	PIM2
5319013	Licoricone	PIM3
5319013	Licoricone	PTPN1
5319013	Licoricone	SIRT1
5319013	Licoricone	SRC

5317478	Gancaonin A	ABCB1
5317478	Gancaonin A	ABCG2
5317478	Gancaonin A	ACHE
5317478	Gancaonin A	ADORA1
5317478	Gancaonin A	ADORA2A
5317478	Gancaonin A	AGTR1
5317478	Gancaonin A	ALDH2
5317478	Gancaonin A	ALOX12
5317478	Gancaonin A	ALOX15
5317478	Gancaonin A	CA12
5317478	Gancaonin A	CA4
5317478	Gancaonin A	CA7
5317478	Gancaonin A	CALCA
5317478	Gancaonin A	CBR1
5317478	Gancaonin A	CDK2
5317478	Gancaonin A	CDK4
5317478	Gancaonin A	CNOT7
5317478	Gancaonin A	CXCR2
5317478	Gancaonin A	CYP19A1
5317478	Gancaonin A	DUSP3
5317478	Gancaonin A	EGFR
5317478	Gancaonin A	EGLN1
5317478	Gancaonin A	ESR1
5317478	Gancaonin A	ESR2
5317478	Gancaonin A	ESRRA
5317478	Gancaonin A	ESRRB
5317478	Gancaonin A	FBP1
5317478	Gancaonin A	GABRA1
5317478	Gancaonin A	GABRA3
5317478	Gancaonin A	GABRB3
5317478	Gancaonin A	GABRB3
5317478	Gancaonin A	GABRG2
5317478	Gancaonin A	GABRG2
5317478	Gancaonin A	HSD17B1
5317478	Gancaonin A	HSD17B2
5317478	Gancaonin A	HSP90AA1
5317478	Gancaonin A	HSP90AB1
5317478	Gancaonin A	HSP90B1
5317478	Gancaonin A	HTR2A
5317478	Gancaonin A	HTR2C
5317478	Gancaonin A	IL2
5317478	Gancaonin A	KISS1R
5317478	Gancaonin A	MAOA
5317478	Gancaonin A	MGAM

5317478	Gancaonin A	MIF
5317478	Gancaonin A	NOS2
5317478	Gancaonin A	OPRK1
5317478	Gancaonin A	PDE5A
5317478	Gancaonin A	PDK1
5317478	Gancaonin A	PFKFB3
5317478	Gancaonin A	PPARA
5317478	Gancaonin A	PTGS1
5317478	Gancaonin A	PTPN1
5317478	Gancaonin A	RELA
5317478	Gancaonin A	SLC6A2
5317478	Gancaonin A	TBXAS1
5317478	Gancaonin A	TYR
5317479	Gancaonin B	ABCB1
5317479	Gancaonin B	ABCG2
5317479	Gancaonin B	ACHE
5317479	Gancaonin B	ADORA1
5317479	Gancaonin B	ALDH2
5317479	Gancaonin B	ALOX12
5317479	Gancaonin B	ALOX15
5317479	Gancaonin B	CA1
5317479	Gancaonin B	CA12
5317479	Gancaonin B	CA2
5317479	Gancaonin B	CA4
5317479	Gancaonin B	CA7
5317479	Gancaonin B	CBR1
5317479	Gancaonin B	CHEK1
5317479	Gancaonin B	CHEK2
5317479	Gancaonin B	CYP19A1
5317479	Gancaonin B	DUSP3
5317479	Gancaonin B	EGFR
5317479	Gancaonin B	ESR1
5317479	Gancaonin B	ESR2
5317479	Gancaonin B	ESRRA
5317479	Gancaonin B	ESRRB
5317479	Gancaonin B	F10
5317479	Gancaonin B	FASN
5317479	Gancaonin B	FBP1
5317479	Gancaonin B	HSD17B1
5317479	Gancaonin B	HSD17B2
5317479	Gancaonin B	HSP90AA1
5317479	Gancaonin B	HSP90AB1
5317479	Gancaonin B	HSP90B1
5317479	Gancaonin B	HTR2A

5317479	Gancaonin B	HTR2C
5317479	Gancaonin B	IDH1
5317479	Gancaonin B	IKBKB
5317479	Gancaonin B	IL2
5317479	Gancaonin B	KISS1R
5317479	Gancaonin B	MGAM
5317479	Gancaonin B	MIF
5317479	Gancaonin B	NOX4
5317479	Gancaonin B	PDK1
5317479	Gancaonin B	PFKFB3
5317479	Gancaonin B	PLAU
5317479	Gancaonin B	PPARA
5317479	Gancaonin B	PRKCA
5317479	Gancaonin B	PRKCB
5317479	Gancaonin B	PRKCD
5317479	Gancaonin B	PRKCE
5317479	Gancaonin B	PRKCG
5317479	Gancaonin B	PRKCH
5317479	Gancaonin B	PTGES
5317479	Gancaonin B	PTGS1
5317479	Gancaonin B	PTGS2
5317479	Gancaonin B	PTPN1
5317479	Gancaonin B	PTPRS
5317479	Gancaonin B	RELA
5317479	Gancaonin B	SIRT1
5317479	Gancaonin B	SLC6A2
5317479	Gancaonin B	TBXAS1
5317479	Gancaonin B	TYR
14604077	3-(3,4-dihydroxyphenyl)-5,7-dihydroxy-8-(3-methylbut-2-enyl)chromone	ABCB1
14604077	3-(3,4-dihydroxyphenyl)-5,7-dihydroxy-8-(3-methylbut-2-enyl)chromone	ACHE
14604077	3-(3,4-dihydroxyphenyl)-5,7-dihydroxy-8-(3-methylbut-2-enyl)chromone	AKT1
14604077	3-(3,4-dihydroxyphenyl)-5,7-dihydroxy-8-(3-methylbut-2-enyl)chromone	ALDH2
14604077	3-(3,4-dihydroxyphenyl)-5,7-dihydroxy-8-(3-methylbut-2-enyl)chromone	ALOX12

14604077	3-(3,4-dihydroxyphenyl)-5,7-dihydroxy-8-(3-methylbut-2-enyl)chromone	ALOX15
14604077	3-(3,4-dihydroxyphenyl)-5,7-dihydroxy-8-(3-methylbut-2-enyl)chromone	BACE1
14604077	3-(3,4-dihydroxyphenyl)-5,7-dihydroxy-8-(3-methylbut-2-enyl)chromone	BCL2
14604077	3-(3,4-dihydroxyphenyl)-5,7-dihydroxy-8-(3-methylbut-2-enyl)chromone	CA4
14604077	3-(3,4-dihydroxyphenyl)-5,7-dihydroxy-8-(3-methylbut-2-enyl)chromone	CCR4
14604077	3-(3,4-dihydroxyphenyl)-5,7-dihydroxy-8-(3-methylbut-2-enyl)chromone	EGFR
14604077	3-(3,4-dihydroxyphenyl)-5,7-dihydroxy-8-(3-methylbut-2-enyl)chromone	ESR1
14604077	3-(3,4-dihydroxyphenyl)-5,7-dihydroxy-8-(3-methylbut-2-enyl)chromone	ESR2
14604077	3-(3,4-dihydroxyphenyl)-5,7-dihydroxy-8-(3-methylbut-2-enyl)chromone	ESRRA
14604077	3-(3,4-dihydroxyphenyl)-5,7-dihydroxy-8-(3-methylbut-2-enyl)chromone	ESRRB
14604077	3-(3,4-dihydroxyphenyl)-5,7-dihydroxy-8-(3-methylbut-2-enyl)chromone	F10
14604077	3-(3,4-dihydroxyphenyl)-5,7-dihydroxy-8-(3-methylbut-2-enyl)chromone	GCGR
14604077	3-(3,4-dihydroxyphenyl)-5,7-dihydroxy-8-(3-methylbut-2-enyl)chromone	HSD17B1
14604077	3-(3,4-dihydroxyphenyl)-5,7-dihydroxy-8-(3-methylbut-2-enyl)chromone	HSP90AA1

14604077	3-(3,4-dihydroxyphenyl)-5,7-dihydroxy-8-(3-methylbut-2-enyl)chromone	HSP90AB1
14604077	3-(3,4-dihydroxyphenyl)-5,7-dihydroxy-8-(3-methylbut-2-enyl)chromone	HSP90B1
14604077	3-(3,4-dihydroxyphenyl)-5,7-dihydroxy-8-(3-methylbut-2-enyl)chromone	HTR2A
14604077	3-(3,4-dihydroxyphenyl)-5,7-dihydroxy-8-(3-methylbut-2-enyl)chromone	HTR2C
14604077	3-(3,4-dihydroxyphenyl)-5,7-dihydroxy-8-(3-methylbut-2-enyl)chromone	MAOA
14604077	3-(3,4-dihydroxyphenyl)-5,7-dihydroxy-8-(3-methylbut-2-enyl)chromone	MCL1
14604077	3-(3,4-dihydroxyphenyl)-5,7-dihydroxy-8-(3-methylbut-2-enyl)chromone	MGAM
14604077	3-(3,4-dihydroxyphenyl)-5,7-dihydroxy-8-(3-methylbut-2-enyl)chromone	MIF
14604077	3-(3,4-dihydroxyphenyl)-5,7-dihydroxy-8-(3-methylbut-2-enyl)chromone	NOS2
14604077	3-(3,4-dihydroxyphenyl)-5,7-dihydroxy-8-(3-methylbut-2-enyl)chromone	NOX4
14604077	3-(3,4-dihydroxyphenyl)-5,7-dihydroxy-8-(3-methylbut-2-enyl)chromone	PDE10A
14604077	3-(3,4-dihydroxyphenyl)-5,7-dihydroxy-8-(3-methylbut-2-enyl)chromone	PDE5A
14604077	3-(3,4-dihydroxyphenyl)-5,7-dihydroxy-8-(3-methylbut-2-enyl)chromone	PFKFB3
14604077	3-(3,4-dihydroxyphenyl)-5,7-dihydroxy-8-(3-methylbut-2-enyl)chromone	PLAU

14604077	3-(3,4-dihydroxyphenyl)-5,7-dihydroxy-8-(3-methylbut-2-enyl)chromone	PTPN1
14604077	3-(3,4-dihydroxyphenyl)-5,7-dihydroxy-8-(3-methylbut-2-enyl)chromone	PTPRS
14604077	3-(3,4-dihydroxyphenyl)-5,7-dihydroxy-8-(3-methylbut-2-enyl)chromone	RELA
14604077	3-(3,4-dihydroxyphenyl)-5,7-dihydroxy-8-(3-methylbut-2-enyl)chromone	SIRT1
14604077	3-(3,4-dihydroxyphenyl)-5,7-dihydroxy-8-(3-methylbut-2-enyl)chromone	TBXAS1
14604077	3-(3,4-dihydroxyphenyl)-5,7-dihydroxy-8-(3-methylbut-2-enyl)chromone	TYR
14604077	3-(3,4-dihydroxyphenyl)-5,7-dihydroxy-8-(3-methylbut-2-enyl)chromone	XDH
14604078	5,7-dihydroxy-3-(4-methoxyphenyl)-8-(3-methylbut-2-enyl)chromone	ABCB1
14604078	5,7-dihydroxy-3-(4-methoxyphenyl)-8-(3-methylbut-2-enyl)chromone	ABCG2
14604078	5,7-dihydroxy-3-(4-methoxyphenyl)-8-(3-methylbut-2-enyl)chromone	ACHE
14604078	5,7-dihydroxy-3-(4-methoxyphenyl)-8-(3-methylbut-2-enyl)chromone	ADORA1
14604078	5,7-dihydroxy-3-(4-methoxyphenyl)-8-(3-methylbut-2-enyl)chromone	ALDH2
14604078	5,7-dihydroxy-3-(4-methoxyphenyl)-8-(3-methylbut-2-enyl)chromone	ALOX15
14604078	5,7-dihydroxy-3-(4-methoxyphenyl)-8-(3-methylbut-2-enyl)chromone	BAD

14604078	5,7-dihydroxy-3-(4-methoxyphenyl)-8-(3-methylbut-2-enyl)chromone	BCL2
14604078	5,7-dihydroxy-3-(4-methoxyphenyl)-8-(3-methylbut-2-enyl)chromone	CA12
14604078	5,7-dihydroxy-3-(4-methoxyphenyl)-8-(3-methylbut-2-enyl)chromone	CA4
14604078	5,7-dihydroxy-3-(4-methoxyphenyl)-8-(3-methylbut-2-enyl)chromone	CA7
14604078	5,7-dihydroxy-3-(4-methoxyphenyl)-8-(3-methylbut-2-enyl)chromone	CBR1
14604078	5,7-dihydroxy-3-(4-methoxyphenyl)-8-(3-methylbut-2-enyl)chromone	CDK1
14604078	5,7-dihydroxy-3-(4-methoxyphenyl)-8-(3-methylbut-2-enyl)chromone	CDK2
14604078	5,7-dihydroxy-3-(4-methoxyphenyl)-8-(3-methylbut-2-enyl)chromone	CDK4
14604078	5,7-dihydroxy-3-(4-methoxyphenyl)-8-(3-methylbut-2-enyl)chromone	CNOT7
14604078	5,7-dihydroxy-3-(4-methoxyphenyl)-8-(3-methylbut-2-enyl)chromone	CYP19A1
14604078	5,7-dihydroxy-3-(4-methoxyphenyl)-8-(3-methylbut-2-enyl)chromone	DUSP3
14604078	5,7-dihydroxy-3-(4-methoxyphenyl)-8-(3-methylbut-2-enyl)chromone	EGFR
14604078	5,7-dihydroxy-3-(4-methoxyphenyl)-8-(3-methylbut-2-enyl)chromone	EP300
14604078	5,7-dihydroxy-3-(4-methoxyphenyl)-8-(3-methylbut-2-enyl)chromone	ESR2

14604078	5,7-dihydroxy-3-(4-methoxyphenyl)-8-(3-methylbut-2-enyl)chromone	ESRRA
14604078	5,7-dihydroxy-3-(4-methoxyphenyl)-8-(3-methylbut-2-enyl)chromone	ESRRB
14604078	5,7-dihydroxy-3-(4-methoxyphenyl)-8-(3-methylbut-2-enyl)chromone	FASN
14604078	5,7-dihydroxy-3-(4-methoxyphenyl)-8-(3-methylbut-2-enyl)chromone	FBP1
14604078	5,7-dihydroxy-3-(4-methoxyphenyl)-8-(3-methylbut-2-enyl)chromone	HSD17B1
14604078	5,7-dihydroxy-3-(4-methoxyphenyl)-8-(3-methylbut-2-enyl)chromone	HSD17B2
14604078	5,7-dihydroxy-3-(4-methoxyphenyl)-8-(3-methylbut-2-enyl)chromone	HSP90AA1
14604078	5,7-dihydroxy-3-(4-methoxyphenyl)-8-(3-methylbut-2-enyl)chromone	HSP90AB1
14604078	5,7-dihydroxy-3-(4-methoxyphenyl)-8-(3-methylbut-2-enyl)chromone	HSP90B1
14604078	5,7-dihydroxy-3-(4-methoxyphenyl)-8-(3-methylbut-2-enyl)chromone	HTR2A
14604078	5,7-dihydroxy-3-(4-methoxyphenyl)-8-(3-methylbut-2-enyl)chromone	HTR2C
14604078	5,7-dihydroxy-3-(4-methoxyphenyl)-8-(3-methylbut-2-enyl)chromone	IL2
14604078	5,7-dihydroxy-3-(4-methoxyphenyl)-8-(3-methylbut-2-enyl)chromone	MCL1
14604078	5,7-dihydroxy-3-(4-methoxyphenyl)-8-(3-methylbut-2-enyl)chromone	MELK

14604078	5,7-dihydroxy-3-(4-methoxyphenyl)-8-(3-methylbut-2-enyl)chromone	MGAM
14604078	5,7-dihydroxy-3-(4-methoxyphenyl)-8-(3-methylbut-2-enyl)chromone	MPI
14604078	5,7-dihydroxy-3-(4-methoxyphenyl)-8-(3-methylbut-2-enyl)chromone	NOS2
14604078	5,7-dihydroxy-3-(4-methoxyphenyl)-8-(3-methylbut-2-enyl)chromone	PDE10A
14604078	5,7-dihydroxy-3-(4-methoxyphenyl)-8-(3-methylbut-2-enyl)chromone	PDE4D
14604078	5,7-dihydroxy-3-(4-methoxyphenyl)-8-(3-methylbut-2-enyl)chromone	PDE5A
14604078	5,7-dihydroxy-3-(4-methoxyphenyl)-8-(3-methylbut-2-enyl)chromone	PDK1
14604078	5,7-dihydroxy-3-(4-methoxyphenyl)-8-(3-methylbut-2-enyl)chromone	PTGS1
14604078	5,7-dihydroxy-3-(4-methoxyphenyl)-8-(3-methylbut-2-enyl)chromone	PTPN1
14604078	5,7-dihydroxy-3-(4-methoxyphenyl)-8-(3-methylbut-2-enyl)chromone	RELA
14604078	5,7-dihydroxy-3-(4-methoxyphenyl)-8-(3-methylbut-2-enyl)chromone	SLC6A2
14604081	2-(3,4-dihydroxyphenyl)-5,7-dihydroxy-6-(3-methylbut-2-enyl)chromone	ABCB1
14604081	2-(3,4-dihydroxyphenyl)-5,7-dihydroxy-6-(3-methylbut-2-enyl)chromone	ABCC1
14604081	2-(3,4-dihydroxyphenyl)-5,7-dihydroxy-6-(3-methylbut-2-enyl)chromone	ABCG2

14604081	2-(3,4-dihydroxyphenyl)-5,7-dihydroxy-6-(3-methylbut-2-enyl)chromone	ACHE
14604081	2-(3,4-dihydroxyphenyl)-5,7-dihydroxy-6-(3-methylbut-2-enyl)chromone	ADORA1
14604081	2-(3,4-dihydroxyphenyl)-5,7-dihydroxy-6-(3-methylbut-2-enyl)chromone	ADORA2A
14604081	2-(3,4-dihydroxyphenyl)-5,7-dihydroxy-6-(3-methylbut-2-enyl)chromone	AHR
14604081	2-(3,4-dihydroxyphenyl)-5,7-dihydroxy-6-(3-methylbut-2-enyl)chromone	AKR1A1
14604081	2-(3,4-dihydroxyphenyl)-5,7-dihydroxy-6-(3-methylbut-2-enyl)chromone	AKR1B1
14604081	2-(3,4-dihydroxyphenyl)-5,7-dihydroxy-6-(3-methylbut-2-enyl)chromone	AKR1B10
14604081	2-(3,4-dihydroxyphenyl)-5,7-dihydroxy-6-(3-methylbut-2-enyl)chromone	AKR1C1
14604081	2-(3,4-dihydroxyphenyl)-5,7-dihydroxy-6-(3-methylbut-2-enyl)chromone	AKR1C2
14604081	2-(3,4-dihydroxyphenyl)-5,7-dihydroxy-6-(3-methylbut-2-enyl)chromone	AKR1C3
14604081	2-(3,4-dihydroxyphenyl)-5,7-dihydroxy-6-(3-methylbut-2-enyl)chromone	AKR1C4
14604081	2-(3,4-dihydroxyphenyl)-5,7-dihydroxy-6-(3-methylbut-2-enyl)chromone	AKT1
14604081	2-(3,4-dihydroxyphenyl)-5,7-dihydroxy-6-(3-methylbut-2-enyl)chromone	ALK
14604081	2-(3,4-dihydroxyphenyl)-5,7-dihydroxy-6-(3-methylbut-2-enyl)chromone	ALOX12

14604081	2-(3,4-dihydroxyphenyl)-5,7-dihydroxy-6-(3-methylbut-2-enyl)chromone	ALOX15
14604081	2-(3,4-dihydroxyphenyl)-5,7-dihydroxy-6-(3-methylbut-2-enyl)chromone	ALOX5
14604081	2-(3,4-dihydroxyphenyl)-5,7-dihydroxy-6-(3-methylbut-2-enyl)chromone	AMY1A
14604081	2-(3,4-dihydroxyphenyl)-5,7-dihydroxy-6-(3-methylbut-2-enyl)chromone	APEX1
14604081	2-(3,4-dihydroxyphenyl)-5,7-dihydroxy-6-(3-methylbut-2-enyl)chromone	APP
14604081	2-(3,4-dihydroxyphenyl)-5,7-dihydroxy-6-(3-methylbut-2-enyl)chromone	AR
14604081	2-(3,4-dihydroxyphenyl)-5,7-dihydroxy-6-(3-methylbut-2-enyl)chromone	ARG1
14604081	2-(3,4-dihydroxyphenyl)-5,7-dihydroxy-6-(3-methylbut-2-enyl)chromone	AURKB
14604081	2-(3,4-dihydroxyphenyl)-5,7-dihydroxy-6-(3-methylbut-2-enyl)chromone	AVPR2
14604081	2-(3,4-dihydroxyphenyl)-5,7-dihydroxy-6-(3-methylbut-2-enyl)chromone	BACE1
14604081	2-(3,4-dihydroxyphenyl)-5,7-dihydroxy-6-(3-methylbut-2-enyl)chromone	BCHE
14604081	2-(3,4-dihydroxyphenyl)-5,7-dihydroxy-6-(3-methylbut-2-enyl)chromone	CA1
14604081	2-(3,4-dihydroxyphenyl)-5,7-dihydroxy-6-(3-methylbut-2-enyl)chromone	CA12
14604081	2-(3,4-dihydroxyphenyl)-5,7-dihydroxy-6-(3-methylbut-2-enyl)chromone	CA13

14604081	2-(3,4-dihydroxyphenyl)-5,7-dihydroxy-6-(3-methylbut-2-enyl)chromone	CA2
14604081	2-(3,4-dihydroxyphenyl)-5,7-dihydroxy-6-(3-methylbut-2-enyl)chromone	CA3
14604081	2-(3,4-dihydroxyphenyl)-5,7-dihydroxy-6-(3-methylbut-2-enyl)chromone	CA4
14604081	2-(3,4-dihydroxyphenyl)-5,7-dihydroxy-6-(3-methylbut-2-enyl)chromone	CA5A
14604081	2-(3,4-dihydroxyphenyl)-5,7-dihydroxy-6-(3-methylbut-2-enyl)chromone	CA6
14604081	2-(3,4-dihydroxyphenyl)-5,7-dihydroxy-6-(3-methylbut-2-enyl)chromone	CA7
14604081	2-(3,4-dihydroxyphenyl)-5,7-dihydroxy-6-(3-methylbut-2-enyl)chromone	CA9
14604081	2-(3,4-dihydroxyphenyl)-5,7-dihydroxy-6-(3-methylbut-2-enyl)chromone	CAMK2B
14604081	2-(3,4-dihydroxyphenyl)-5,7-dihydroxy-6-(3-methylbut-2-enyl)chromone	CBR1
14604081	2-(3,4-dihydroxyphenyl)-5,7-dihydroxy-6-(3-methylbut-2-enyl)chromone	CCNB1
14604081	2-(3,4-dihydroxyphenyl)-5,7-dihydroxy-6-(3-methylbut-2-enyl)chromone	CCNB2
14604081	2-(3,4-dihydroxyphenyl)-5,7-dihydroxy-6-(3-methylbut-2-enyl)chromone	CCNB3
14604081	2-(3,4-dihydroxyphenyl)-5,7-dihydroxy-6-(3-methylbut-2-enyl)chromone	CD38
14604081	2-(3,4-dihydroxyphenyl)-5,7-dihydroxy-6-(3-methylbut-2-enyl)chromone	CDK1

14604081	2-(3,4-dihydroxyphenyl)-5,7-dihydroxy-6-(3-methylbut-2-enyl)chromone	CDK5
14604081	2-(3,4-dihydroxyphenyl)-5,7-dihydroxy-6-(3-methylbut-2-enyl)chromone	CDK5R1
14604081	2-(3,4-dihydroxyphenyl)-5,7-dihydroxy-6-(3-methylbut-2-enyl)chromone	CDK6
14604081	2-(3,4-dihydroxyphenyl)-5,7-dihydroxy-6-(3-methylbut-2-enyl)chromone	CFTR
14604081	2-(3,4-dihydroxyphenyl)-5,7-dihydroxy-6-(3-methylbut-2-enyl)chromone	CSNK2A1
14604081	2-(3,4-dihydroxyphenyl)-5,7-dihydroxy-6-(3-methylbut-2-enyl)chromone	CYP19A1
14604081	2-(3,4-dihydroxyphenyl)-5,7-dihydroxy-6-(3-methylbut-2-enyl)chromone	CYP1B1
14604081	2-(3,4-dihydroxyphenyl)-5,7-dihydroxy-6-(3-methylbut-2-enyl)chromone	DAPK1
14604081	2-(3,4-dihydroxyphenyl)-5,7-dihydroxy-6-(3-methylbut-2-enyl)chromone	DRD4
14604081	2-(3,4-dihydroxyphenyl)-5,7-dihydroxy-6-(3-methylbut-2-enyl)chromone	ESRRA
14604081	2-(3,4-dihydroxyphenyl)-5,7-dihydroxy-6-(3-methylbut-2-enyl)chromone	F2
14604081	2-(3,4-dihydroxyphenyl)-5,7-dihydroxy-6-(3-methylbut-2-enyl)chromone	FLT3
14604081	2-(3,4-dihydroxyphenyl)-5,7-dihydroxy-6-(3-methylbut-2-enyl)chromone	GLO1
14604081	2-(3,4-dihydroxyphenyl)-5,7-dihydroxy-6-(3-methylbut-2-enyl)chromone	GPR35

14604081	2-(3,4-dihydroxyphenyl)-5,7-dihydroxy-6-(3-methylbut-2-enyl)chromone	GSK3B
14604081	2-(3,4-dihydroxyphenyl)-5,7-dihydroxy-6-(3-methylbut-2-enyl)chromone	IGF1R
14604081	2-(3,4-dihydroxyphenyl)-5,7-dihydroxy-6-(3-methylbut-2-enyl)chromone	INSR
14604081	2-(3,4-dihydroxyphenyl)-5,7-dihydroxy-6-(3-methylbut-2-enyl)chromone	KDM4E
14604081	2-(3,4-dihydroxyphenyl)-5,7-dihydroxy-6-(3-methylbut-2-enyl)chromone	KDR
14604081	2-(3,4-dihydroxyphenyl)-5,7-dihydroxy-6-(3-methylbut-2-enyl)chromone	MAOA
14604081	2-(3,4-dihydroxyphenyl)-5,7-dihydroxy-6-(3-methylbut-2-enyl)chromone	MAPT
14604081	2-(3,4-dihydroxyphenyl)-5,7-dihydroxy-6-(3-methylbut-2-enyl)chromone	MET
14604081	2-(3,4-dihydroxyphenyl)-5,7-dihydroxy-6-(3-methylbut-2-enyl)chromone	MMP12
14604081	2-(3,4-dihydroxyphenyl)-5,7-dihydroxy-6-(3-methylbut-2-enyl)chromone	MMP13
14604081	2-(3,4-dihydroxyphenyl)-5,7-dihydroxy-6-(3-methylbut-2-enyl)chromone	MMP2
14604081	2-(3,4-dihydroxyphenyl)-5,7-dihydroxy-6-(3-methylbut-2-enyl)chromone	MMP3
14604081	2-(3,4-dihydroxyphenyl)-5,7-dihydroxy-6-(3-methylbut-2-enyl)chromone	MMP9
14604081	2-(3,4-dihydroxyphenyl)-5,7-dihydroxy-6-(3-methylbut-2-enyl)chromone	MPO

14604081	2-(3,4-dihydroxyphenyl)-5,7-dihydroxy-6-(3-methylbut-2-enyl)chromone	MYLK
14604081	2-(3,4-dihydroxyphenyl)-5,7-dihydroxy-6-(3-methylbut-2-enyl)chromone	NAE1
14604081	2-(3,4-dihydroxyphenyl)-5,7-dihydroxy-6-(3-methylbut-2-enyl)chromone	NEK2
14604081	2-(3,4-dihydroxyphenyl)-5,7-dihydroxy-6-(3-methylbut-2-enyl)chromone	NEK6
14604081	2-(3,4-dihydroxyphenyl)-5,7-dihydroxy-6-(3-methylbut-2-enyl)chromone	NOX4
14604081	2-(3,4-dihydroxyphenyl)-5,7-dihydroxy-6-(3-methylbut-2-enyl)chromone	NUAK1
14604081	2-(3,4-dihydroxyphenyl)-5,7-dihydroxy-6-(3-methylbut-2-enyl)chromone	PARP1
14604081	2-(3,4-dihydroxyphenyl)-5,7-dihydroxy-6-(3-methylbut-2-enyl)chromone	PDE5A
14604081	2-(3,4-dihydroxyphenyl)-5,7-dihydroxy-6-(3-methylbut-2-enyl)chromone	PFKFB3
14604081	2-(3,4-dihydroxyphenyl)-5,7-dihydroxy-6-(3-methylbut-2-enyl)chromone	PIK3CG
14604081	2-(3,4-dihydroxyphenyl)-5,7-dihydroxy-6-(3-methylbut-2-enyl)chromone	PIK3R1
14604081	2-(3,4-dihydroxyphenyl)-5,7-dihydroxy-6-(3-methylbut-2-enyl)chromone	PIM1
14604081	2-(3,4-dihydroxyphenyl)-5,7-dihydroxy-6-(3-methylbut-2-enyl)chromone	PKN1
14604081	2-(3,4-dihydroxyphenyl)-5,7-dihydroxy-6-(3-methylbut-2-enyl)chromone	PLA2G1B

14604081	2-(3,4-dihydroxyphenyl)-5,7-dihydroxy-6-(3-methylbut-2-enyl)chromone	PLA2G2A
14604081	2-(3,4-dihydroxyphenyl)-5,7-dihydroxy-6-(3-methylbut-2-enyl)chromone	PLG
14604081	2-(3,4-dihydroxyphenyl)-5,7-dihydroxy-6-(3-methylbut-2-enyl)chromone	PLK1
14604081	2-(3,4-dihydroxyphenyl)-5,7-dihydroxy-6-(3-methylbut-2-enyl)chromone	PPARG
14604081	2-(3,4-dihydroxyphenyl)-5,7-dihydroxy-6-(3-methylbut-2-enyl)chromone	PTK2
14604081	2-(3,4-dihydroxyphenyl)-5,7-dihydroxy-6-(3-methylbut-2-enyl)chromone	PTPN1
14604081	2-(3,4-dihydroxyphenyl)-5,7-dihydroxy-6-(3-methylbut-2-enyl)chromone	PTPRS
14604081	2-(3,4-dihydroxyphenyl)-5,7-dihydroxy-6-(3-methylbut-2-enyl)chromone	PYGL
14604081	2-(3,4-dihydroxyphenyl)-5,7-dihydroxy-6-(3-methylbut-2-enyl)chromone	ST6GAL1
14604081	2-(3,4-dihydroxyphenyl)-5,7-dihydroxy-6-(3-methylbut-2-enyl)chromone	SYK
14604081	2-(3,4-dihydroxyphenyl)-5,7-dihydroxy-6-(3-methylbut-2-enyl)chromone	TNKS
14604081	2-(3,4-dihydroxyphenyl)-5,7-dihydroxy-6-(3-methylbut-2-enyl)chromone	TNKS2
14604081	2-(3,4-dihydroxyphenyl)-5,7-dihydroxy-6-(3-methylbut-2-enyl)chromone	TOP1
14604081	2-(3,4-dihydroxyphenyl)-5,7-dihydroxy-6-(3-methylbut-2-enyl)chromone	TOP2A

14604081	2-(3,4-dihydroxyphenyl)-5,7-dihydroxy-6-(3-methylbut-2-enyl)chromone	TTR
14604081	2-(3,4-dihydroxyphenyl)-5,7-dihydroxy-6-(3-methylbut-2-enyl)chromone	TYR
14604081	2-(3,4-dihydroxyphenyl)-5,7-dihydroxy-6-(3-methylbut-2-enyl)chromone	XDH
480787	Glycyrin	ACHE
480787	Glycyrin	ACVR1
480787	Glycyrin	ADAM17
480787	Glycyrin	ADORA2B
480787	Glycyrin	AKR1B10
480787	Glycyrin	ALDH2
480787	Glycyrin	AURKA
480787	Glycyrin	AVPR2
480787	Glycyrin	BACE2
480787	Glycyrin	BMP1
480787	Glycyrin	CA12
480787	Glycyrin	CES1
480787	Glycyrin	CES2
480787	Glycyrin	CHRM3
480787	Glycyrin	CHRNA7
480787	Glycyrin	COMT
480787	Glycyrin	CSNK1A1
480787	Glycyrin	CTSB
480787	Glycyrin	CTSL
480787	Glycyrin	CYP19A1
480787	Glycyrin	DPP4
480787	Glycyrin	DUSP3
480787	Glycyrin	EP300
480787	Glycyrin	EPHX2
480787	Glycyrin	ERN1
480787	Glycyrin	F3
480787	Glycyrin	FKBP1A
480787	Glycyrin	FLT1
480787	Glycyrin	FLT3
480787	Glycyrin	FNTA
480787	Glycyrin	FNTB
480787	Glycyrin	GCGR
480787	Glycyrin	HDAC1
480787	Glycyrin	HDAC10
480787	Glycyrin	HDAC11

480787	Glycyrin	HDAC2
480787	Glycyrin	HDAC3
480787	Glycyrin	HDAC3
480787	Glycyrin	HDAC4
480787	Glycyrin	HDAC5
480787	Glycyrin	HDAC6
480787	Glycyrin	HDAC7
480787	Glycyrin	HDAC8
480787	Glycyrin	HDAC9
480787	Glycyrin	HPGD
480787	Glycyrin	HPGDS
480787	Glycyrin	HSD17B2
480787	Glycyrin	HTR3A
480787	Glycyrin	IKBKE
480787	Glycyrin	KDR
480787	Glycyrin	LIMK1
480787	Glycyrin	LIMK2
480787	Glycyrin	MAOB
480787	Glycyrin	MAPK14
480787	Glycyrin	MELK
480787	Glycyrin	MERTK
480787	Glycyrin	MET
480787	Glycyrin	MGLL
480787	Glycyrin	MIF
480787	Glycyrin	MMP1
480787	Glycyrin	MMP13
480787	Glycyrin	MMP2
480787	Glycyrin	MMP3
480787	Glycyrin	MMP9
480787	Glycyrin	MPI
480787	Glycyrin	NCOR1
480787	Glycyrin	NCOR2
480787	Glycyrin	NOS2
480787	Glycyrin	ODC1
480787	Glycyrin	PDE4A
480787	Glycyrin	PDE4B
480787	Glycyrin	PDE4C
480787	Glycyrin	PDE4D
480787	Glycyrin	PDE7A
480787	Glycyrin	PDK1
480787	Glycyrin	PIK3CA
480787	Glycyrin	PLEC
480787	Glycyrin	PLK1
480787	Glycyrin	PPARG

480787	Glycyrin	PRF1
480787	Glycyrin	PTAFR
480787	Glycyrin	PTPN1
480787	Glycyrin	ROCK1
480787	Glycyrin	ROCK2
480787	Glycyrin	RPS6KA3
480787	Glycyrin	S1PR1
480787	Glycyrin	S1PR3
480787	Glycyrin	SCD
480787	Glycyrin	SF3B3
480787	Glycyrin	SLC29A1
480787	Glycyrin	SLC5A1
480787	Glycyrin	SREBF2
480787	Glycyrin	STK17B
480787	Glycyrin	SYK
480787	Glycyrin	TAOK1
480787	Glycyrin	TAOK3
480787	Glycyrin	TBK1
480787	Glycyrin	TBXA2R
480787	Glycyrin	TGFBR1
480787	Glycyrin	TLR9
480787	Glycyrin	TRPV1
480787	Glycyrin	XPO1
503731	Licocoumarone	ABHD6
503731	Licocoumarone	ACHE
503731	Licocoumarone	ADRA2A
503731	Licocoumarone	AGTR1
503731	Licocoumarone	ALOX5AP
503731	Licocoumarone	APH1A
503731	Licocoumarone	APH1B
503731	Licocoumarone	BACE1
503731	Licocoumarone	BACE2
503731	Licocoumarone	BCHE
503731	Licocoumarone	CASP3
503731	Licocoumarone	CASP7
503731	Licocoumarone	CCNB1
503731	Licocoumarone	CCNE1
503731	Licocoumarone	CCNE1
503731	Licocoumarone	CCNE2
503731	Licocoumarone	CDC25A
503731	Licocoumarone	CDC25B
503731	Licocoumarone	CDK1
503731	Licocoumarone	CDK2
503731	Licocoumarone	CDK2

503731	Licocoumarone	CHEK1
503731	Licocoumarone	CXCR2
503731	Licocoumarone	CYP17A1
503731	Licocoumarone	DUT
503731	Licocoumarone	EDNRA
503731	Licocoumarone	EDNRB
503731	Licocoumarone	EPHA2
503731	Licocoumarone	ESR1
503731	Licocoumarone	FAAH
503731	Licocoumarone	FGFR1
503731	Licocoumarone	FLT1
503731	Licocoumarone	FNTA
503731	Licocoumarone	FNTB
503731	Licocoumarone	GCGR
503731	Licocoumarone	GRM5
503731	Licocoumarone	HIF1A
503731	Licocoumarone	HPGD
503731	Licocoumarone	HPSE
503731	Licocoumarone	HSP90AB1
503731	Licocoumarone	HTR6
503731	Licocoumarone	HTR7
503731	Licocoumarone	IMPDH2
503731	Licocoumarone	JAK1
503731	Licocoumarone	JAK3
503731	Licocoumarone	LRRK2
503731	Licocoumarone	MGLL
503731	Licocoumarone	MKNK1
503731	Licocoumarone	MMP1
503731	Licocoumarone	MMP7
503731	Licocoumarone	MMP8
503731	Licocoumarone	NCSTN
503731	Licocoumarone	NR3C2
503731	Licocoumarone	P2RX3
503731	Licocoumarone	PGR
503731	Licocoumarone	PIK3CA
503731	Licocoumarone	PPARG
503731	Licocoumarone	PRKCA
503731	Licocoumarone	PSEN1
503731	Licocoumarone	PSEN2
503731	Licocoumarone	PSENEN
503731	Licocoumarone	RASGRP3
503731	Licocoumarone	ROCK2
503731	Licocoumarone	RPS6KA1
503731	Licocoumarone	SCN9A

503731	Licocoumarone	SLC5A1
503731	Licocoumarone	SLC6A2
503731	Licocoumarone	WEE1
5281789	Licoisoflavone	ABCB1
5281789	Licoisoflavone	ABCG2
5281789	Licoisoflavone	ACHE
5281789	Licoisoflavone	ADORA2A
5281789	Licoisoflavone	ADRB1
5281789	Licoisoflavone	ADRB2
5281789	Licoisoflavone	ALDH2
5281789	Licoisoflavone	AR
5281789	Licoisoflavone	BCL2L1
5281789	Licoisoflavone	CA12
5281789	Licoisoflavone	CHEK2
5281789	Licoisoflavone	CYP19A1
5281789	Licoisoflavone	EGFR
5281789	Licoisoflavone	ESR1
5281789	Licoisoflavone	ESRRA
5281789	Licoisoflavone	ESRRB
5281789	Licoisoflavone	GCGR
5281789	Licoisoflavone	HSD17B1
5281789	Licoisoflavone	HSD17B2
5281789	Licoisoflavone	HSP90AA1
5281789	Licoisoflavone	HSP90AB1
5281789	Licoisoflavone	HTR2A
5281789	Licoisoflavone	IL2
5281789	Licoisoflavone	MAOA
5281789	Licoisoflavone	MGAM
5281789	Licoisoflavone	MIF
5281789	Licoisoflavone	NOX4
5281789	Licoisoflavone	PTPN1
5281789	Licoisoflavone	PTPRS
5281789	Licoisoflavone	RELA
5281789	Licoisoflavone	TBXAS1
5281789	Licoisoflavone	TYR
5281789	Licoisoflavone	XDH
5318869	Jaranol	ABCB1
5318869	Jaranol	ABCC1
5318869	Jaranol	ABCG2
5318869	Jaranol	ACHE
5318869	Jaranol	ADORA1
5318869	Jaranol	ADORA2A
5318869	Jaranol	ADORA3
5318869	Jaranol	AHR

5318869	Jaranol	AKR1B1
5318869	Jaranol	AKR1B10
5318869	Jaranol	AKR1C1
5318869	Jaranol	AKT1
5318869	Jaranol	ALK
5318869	Jaranol	ALOX15
5318869	Jaranol	ALOX5
5318869	Jaranol	AMY1A
5318869	Jaranol	APEX1
5318869	Jaranol	APP
5318869	Jaranol	AR
5318869	Jaranol	ARG1
5318869	Jaranol	AURKB
5318869	Jaranol	AVPR2
5318869	Jaranol	AXL
5318869	Jaranol	BACE1
5318869	Jaranol	BCHE
5318869	Jaranol	CA1
5318869	Jaranol	CA12
5318869	Jaranol	CA13
5318869	Jaranol	CA14
5318869	Jaranol	CA2
5318869	Jaranol	CA3
5318869	Jaranol	CA4
5318869	Jaranol	CA5A
5318869	Jaranol	CA6
5318869	Jaranol	CA7
5318869	Jaranol	CA9
5318869	Jaranol	CAMK2B
5318869	Jaranol	CCNB1
5318869	Jaranol	CCNB2
5318869	Jaranol	CCNB3
5318869	Jaranol	CDK1
5318869	Jaranol	CDK1
5318869	Jaranol	CDK2
5318869	Jaranol	CDK5
5318869	Jaranol	CDK5R1
5318869	Jaranol	CDK6
5318869	Jaranol	CSNK2A1
5318869	Jaranol	CYP19A1
5318869	Jaranol	CYP1B1
5318869	Jaranol	DAPK1
5318869	Jaranol	DRD4
5318869	Jaranol	EGFR

5318869	Jaranol	ESR1
5318869	Jaranol	ESR2
5318869	Jaranol	ESRRA
5318869	Jaranol	F2
5318869	Jaranol	FLT3
5318869	Jaranol	GLO1
5318869	Jaranol	GPR35
5318869	Jaranol	GSK3B
5318869	Jaranol	HSD17B1
5318869	Jaranol	HSD17B2
5318869	Jaranol	IGF1R
5318869	Jaranol	KDM4E
5318869	Jaranol	KDR
5318869	Jaranol	KIT
5318869	Jaranol	MAOA
5318869	Jaranol	MCL1
5318869	Jaranol	MET
5318869	Jaranol	MMP12
5318869	Jaranol	MMP13
5318869	Jaranol	MMP2
5318869	Jaranol	MMP3
5318869	Jaranol	MMP9
5318869	Jaranol	MPG
5318869	Jaranol	MYLK
5318869	Jaranol	NAE1
5318869	Jaranol	NEK6
5318869	Jaranol	NOS2
5318869	Jaranol	NOX4
5318869	Jaranol	NUAK1
5318869	Jaranol	ODC1
5318869	Jaranol	OPRD1
5318869	Jaranol	OPRM1
5318869	Jaranol	PARP1
5318869	Jaranol	PIK3CG
5318869	Jaranol	PIK3R1
5318869	Jaranol	PIM1
5318869	Jaranol	PLG
5318869	Jaranol	PLK1
5318869	Jaranol	PTGS2
5318869	Jaranol	PTK2
5318869	Jaranol	PTPN1
5318869	Jaranol	PTPRS
5318869	Jaranol	SLC22A12
5318869	Jaranol	SRC

5318869	Jaranol	SYK
5318869	Jaranol	TAS2R31
5318869	Jaranol	TERT
5318869	Jaranol	TNKS
5318869	Jaranol	TNKS2
5318869	Jaranol	TOP2A
5318869	Jaranol	TYR
5318869	Jaranol	XDH
392443	licoisoflavanone	ABAT
392443	licoisoflavanone	ABL1
392443	licoisoflavanone	ADAM17
392443	licoisoflavanone	ADAMTS4
392443	licoisoflavanone	ADAMTS5
392443	licoisoflavanone	ADORA2A
392443	licoisoflavanone	ADORA2B
392443	licoisoflavanone	ALK
392443	licoisoflavanone	ALPG
392443	licoisoflavanone	ALPL
392443	licoisoflavanone	AURKA
392443	licoisoflavanone	AURKB
392443	licoisoflavanone	BMP1
392443	licoisoflavanone	BRAF
392443	licoisoflavanone	CCNB1
392443	licoisoflavanone	CCNB1
392443	licoisoflavanone	CCNB2
392443	licoisoflavanone	CCNB3
392443	licoisoflavanone	CCND1
392443	licoisoflavanone	CCND1
392443	licoisoflavanone	CCND2
392443	licoisoflavanone	CCND3
392443	licoisoflavanone	CCNE1
392443	licoisoflavanone	CCNE2
392443	licoisoflavanone	CDC7
392443	licoisoflavanone	CDK1
392443	licoisoflavanone	CDK1
392443	licoisoflavanone	CDK2
392443	licoisoflavanone	CDK4
392443	licoisoflavanone	CDK4
392443	licoisoflavanone	CHEK1
392443	licoisoflavanone	CHRNA7
392443	licoisoflavanone	CSNK1G1
392443	licoisoflavanone	CTSD
392443	licoisoflavanone	CXCR1
392443	licoisoflavanone	CXCR2

392443	licoisoflavanone	DBF4
392443	licoisoflavanone	DNM1
392443	licoisoflavanone	EP300
392443	licoisoflavanone	EPHB2
392443	licoisoflavanone	EPHB4
392443	licoisoflavanone	ERN1
392443	licoisoflavanone	ESR1
392443	licoisoflavanone	ESR2
392443	licoisoflavanone	EZR
392443	licoisoflavanone	F10
392443	licoisoflavanone	FGFR2
392443	licoisoflavanone	FLT1
392443	licoisoflavanone	FLT3
392443	licoisoflavanone	GRM4
392443	licoisoflavanone	GSK3A
392443	licoisoflavanone	HCK
392443	licoisoflavanone	HDAC10
392443	licoisoflavanone	HDAC11
392443	licoisoflavanone	HDAC3
392443	licoisoflavanone	HDAC3
392443	licoisoflavanone	HDAC5
392443	licoisoflavanone	HDAC7
392443	licoisoflavanone	HDAC9
392443	licoisoflavanone	HSD11B1
392443	licoisoflavanone	HSD17B2
392443	licoisoflavanone	HSD17B3
392443	licoisoflavanone	IMPDH1
392443	licoisoflavanone	IMPDH2
392443	licoisoflavanone	JAK3
392443	licoisoflavanone	KDM1A
392443	licoisoflavanone	LCK
392443	licoisoflavanone	MAOB
392443	licoisoflavanone	MAP2K1
392443	licoisoflavanone	MAPK1
392443	licoisoflavanone	MAPK14
392443	licoisoflavanone	MARK1
392443	licoisoflavanone	MME
392443	licoisoflavanone	MMP14
392443	licoisoflavanone	MMP2
392443	licoisoflavanone	MMP7
392443	licoisoflavanone	MMP8
392443	licoisoflavanone	MTOR
392443	licoisoflavanone	MYLK
392443	licoisoflavanone	NCOR1

392443	licoisoflavanone	NCOR2
392443	licoisoflavanone	ODC1
392443	licoisoflavanone	PARP1
392443	licoisoflavanone	PDE4A
392443	licoisoflavanone	PDE4B
392443	licoisoflavanone	PDE4C
392443	licoisoflavanone	PDE7A
392443	licoisoflavanone	PDGFRB
392443	licoisoflavanone	PDPK1
392443	licoisoflavanone	PI4KB
392443	licoisoflavanone	PIK3CB
392443	licoisoflavanone	PIK3CD
392443	licoisoflavanone	PIK3CG
392443	licoisoflavanone	PIK3R1
392443	licoisoflavanone	PIM1
392443	licoisoflavanone	PIM2
392443	licoisoflavanone	PLAA
392443	licoisoflavanone	PLK1
392443	licoisoflavanone	PRKDC
392443	licoisoflavanone	PTK6
392443	licoisoflavanone	PTPN1
392443	licoisoflavanone	RAF1
392443	licoisoflavanone	RET
392443	licoisoflavanone	ROCK1
392443	licoisoflavanone	RPS6KA1
392443	licoisoflavanone	RPS6KA2
392443	licoisoflavanone	SPHK1
392443	licoisoflavanone	SPHK2
392443	licoisoflavanone	SYK
392443	licoisoflavanone	TRPM8
392443	licoisoflavanone	TUBB1
392443	licoisoflavanone	VCP
392443	licoisoflavanone	WEE1
10336244	shinpterocarpin	ABL1
10336244	shinpterocarpin	ADORA2B
10336244	shinpterocarpin	AKT1
10336244	shinpterocarpin	ALDH2
10336244	shinpterocarpin	AR
10336244	shinpterocarpin	AURKA
10336244	shinpterocarpin	BAD
10336244	shinpterocarpin	BLK
10336244	shinpterocarpin	CASK
10336244	shinpterocarpin	CCNB1
10336244	shinpterocarpin	CCNB2

10336244	shinpterocarpin	CCNB3
10336244	shinpterocarpin	CDK1
10336244	shinpterocarpin	CDK1
10336244	shinpterocarpin	CDK2
10336244	shinpterocarpin	CDK4
10336244	shinpterocarpin	CDK5
10336244	shinpterocarpin	CDK5R1
10336244	shinpterocarpin	CHEK1
10336244	shinpterocarpin	CLK3
10336244	shinpterocarpin	COMT
10336244	shinpterocarpin	CSF1R
10336244	shinpterocarpin	CSNK1D
10336244	shinpterocarpin	CSNK1G1
10336244	shinpterocarpin	CTSS
10336244	shinpterocarpin	DAPK3
10336244	shinpterocarpin	DPP4
10336244	shinpterocarpin	DSTYK
10336244	shinpterocarpin	EGFR
10336244	shinpterocarpin	ELANE
10336244	shinpterocarpin	EP300
10336244	shinpterocarpin	EPHA2
10336244	shinpterocarpin	EPHB4
10336244	shinpterocarpin	ERBB2
10336244	shinpterocarpin	ERN1
10336244	shinpterocarpin	ESR1
10336244	shinpterocarpin	ESR2
10336244	shinpterocarpin	FLT1
10336244	shinpterocarpin	FLT3
10336244	shinpterocarpin	FLT4
10336244	shinpterocarpin	FNTA
10336244	shinpterocarpin	FNTB
10336244	shinpterocarpin	FYN
10336244	shinpterocarpin	GABRA5
10336244	shinpterocarpin	GABRB3
10336244	shinpterocarpin	GABRG2
10336244	shinpterocarpin	GCGR
10336244	shinpterocarpin	GRK7
10336244	shinpterocarpin	GRM4
10336244	shinpterocarpin	GSK3B
10336244	shinpterocarpin	HCK
10336244	shinpterocarpin	HDAC1
10336244	shinpterocarpin	HIPK4
10336244	shinpterocarpin	HPGDS
10336244	shinpterocarpin	HSD17B2

10336244	shinpterocarpin	HSD17B3
10336244	shinpterocarpin	JAK3
10336244	shinpterocarpin	LCK
10336244	shinpterocarpin	LNPEP
10336244	shinpterocarpin	LRRK2
10336244	shinpterocarpin	LTA4H
10336244	shinpterocarpin	MAP2K3
10336244	shinpterocarpin	MAPK1
10336244	shinpterocarpin	MAPK14
10336244	shinpterocarpin	MBD2
10336244	shinpterocarpin	MERTK
10336244	shinpterocarpin	MIF
10336244	shinpterocarpin	MTOR
10336244	shinpterocarpin	MYLK
10336244	shinpterocarpin	NR1D1
10336244	shinpterocarpin	NR4A1
10336244	shinpterocarpin	PARP1
10336244	shinpterocarpin	PDE3A
10336244	shinpterocarpin	PDE4B
10336244	shinpterocarpin	PDE5A
10336244	shinpterocarpin	PDE7A
10336244	shinpterocarpin	PDGFRA
10336244	shinpterocarpin	PDGFRA
10336244	shinpterocarpin	PDGFRB
10336244	shinpterocarpin	PDGFRB
10336244	shinpterocarpin	PFKFB3
10336244	shinpterocarpin	PHKG2
10336244	shinpterocarpin	PI4KB
10336244	shinpterocarpin	PIK3C2G
10336244	shinpterocarpin	PIK3CB
10336244	shinpterocarpin	PIK3CD
10336244	shinpterocarpin	PIK3CG
10336244	shinpterocarpin	PIM1
10336244	shinpterocarpin	PIM2
10336244	shinpterocarpin	PIM3
10336244	shinpterocarpin	PIP4K2C
10336244	shinpterocarpin	PIP5K1C
10336244	shinpterocarpin	PLAA
10336244	shinpterocarpin	PLK1
10336244	shinpterocarpin	PRKDC
10336244	shinpterocarpin	PTAFR
10336244	shinpterocarpin	PTPN1
10336244	shinpterocarpin	RET
10336244	shinpterocarpin	RPS6KA3

10336244	shinpterocarpin	SPHK1
10336244	shinpterocarpin	SPHK2
10336244	shinpterocarpin	SREBF2
10336244	shinpterocarpin	SYK
10336244	shinpterocarpin	TAOK2
10336244	shinpterocarpin	TLR9
10336244	shinpterocarpin	VCP
10336244	shinpterocarpin	WEE1
10336244	shinpterocarpin	YES1
11267805	(E)-3-[3,4-dihydroxy-5-(3-methylbut-2-enyl)phenyl]-1-(2,4-dihydroxyphenyl)prop-2-en-1-one	ABCB1
11267805	(E)-3-[3,4-dihydroxy-5-(3-methylbut-2-enyl)phenyl]-1-(2,4-dihydroxyphenyl)prop-2-en-1-one	ABCG2
11267805	(E)-3-[3,4-dihydroxy-5-(3-methylbut-2-enyl)phenyl]-1-(2,4-dihydroxyphenyl)prop-2-en-1-one	ADAM17
11267805	(E)-3-[3,4-dihydroxy-5-(3-methylbut-2-enyl)phenyl]-1-(2,4-dihydroxyphenyl)prop-2-en-1-one	ADAMTS4
11267805	(E)-3-[3,4-dihydroxy-5-(3-methylbut-2-enyl)phenyl]-1-(2,4-dihydroxyphenyl)prop-2-en-1-one	ADAMTS5
11267805	(E)-3-[3,4-dihydroxy-5-(3-methylbut-2-enyl)phenyl]-1-(2,4-dihydroxyphenyl)prop-2-en-1-one	AKR1B1
11267805	(E)-3-[3,4-dihydroxy-5-(3-methylbut-2-enyl)phenyl]-1-(2,4-dihydroxyphenyl)prop-2-en-1-one	AKT2
11267805	(E)-3-[3,4-dihydroxy-5-(3-methylbut-2-enyl)phenyl]-1-(2,4-dihydroxyphenyl)prop-2-en-1-one	ALDH2
11267805	(E)-3-[3,4-dihydroxy-5-(3-methylbut-2-enyl)phenyl]-1-	ALOX5

	(2,4-dihydroxyphenyl)prop-2-en-1-one	
11267805	(E)-3-[3,4-dihydroxy-5-(3-methylbut-2-enyl)phenyl]-1-(2,4-dihydroxyphenyl)prop-2-en-1-one	APP
11267805	(E)-3-[3,4-dihydroxy-5-(3-methylbut-2-enyl)phenyl]-1-(2,4-dihydroxyphenyl)prop-2-en-1-one	AR
11267805	(E)-3-[3,4-dihydroxy-5-(3-methylbut-2-enyl)phenyl]-1-(2,4-dihydroxyphenyl)prop-2-en-1-one	AURKA
11267805	(E)-3-[3,4-dihydroxy-5-(3-methylbut-2-enyl)phenyl]-1-(2,4-dihydroxyphenyl)prop-2-en-1-one	BACE1
11267805	(E)-3-[3,4-dihydroxy-5-(3-methylbut-2-enyl)phenyl]-1-(2,4-dihydroxyphenyl)prop-2-en-1-one	BRAF
11267805	(E)-3-[3,4-dihydroxy-5-(3-methylbut-2-enyl)phenyl]-1-(2,4-dihydroxyphenyl)prop-2-en-1-one	CCNE1
11267805	(E)-3-[3,4-dihydroxy-5-(3-methylbut-2-enyl)phenyl]-1-(2,4-dihydroxyphenyl)prop-2-en-1-one	CCNE1
11267805	(E)-3-[3,4-dihydroxy-5-(3-methylbut-2-enyl)phenyl]-1-(2,4-dihydroxyphenyl)prop-2-en-1-one	CDK2
11267805	(E)-3-[3,4-dihydroxy-5-(3-methylbut-2-enyl)phenyl]-1-(2,4-dihydroxyphenyl)prop-2-en-1-one	CDK3
11267805	(E)-3-[3,4-dihydroxy-5-(3-methylbut-2-enyl)phenyl]-1-(2,4-dihydroxyphenyl)prop-2-en-1-one	CHEK1
11267805	(E)-3-[3,4-dihydroxy-5-(3-methylbut-2-enyl)phenyl]-1-	CHRNA7

	(2,4-dihydroxyphenyl)prop-2-en-1-one	
11267805	(E)-3-[3,4-dihydroxy-5-(3-methylbut-2-enyl)phenyl]-1-(2,4-dihydroxyphenyl)prop-2-en-1-one	CYP19A1
11267805	(E)-3-[3,4-dihydroxy-5-(3-methylbut-2-enyl)phenyl]-1-(2,4-dihydroxyphenyl)prop-2-en-1-one	DHODH
11267805	(E)-3-[3,4-dihydroxy-5-(3-methylbut-2-enyl)phenyl]-1-(2,4-dihydroxyphenyl)prop-2-en-1-one	DNM1
11267805	(E)-3-[3,4-dihydroxy-5-(3-methylbut-2-enyl)phenyl]-1-(2,4-dihydroxyphenyl)prop-2-en-1-one	DYRK1B
11267805	(E)-3-[3,4-dihydroxy-5-(3-methylbut-2-enyl)phenyl]-1-(2,4-dihydroxyphenyl)prop-2-en-1-one	EGFR
11267805	(E)-3-[3,4-dihydroxy-5-(3-methylbut-2-enyl)phenyl]-1-(2,4-dihydroxyphenyl)prop-2-en-1-one	ERN1
11267805	(E)-3-[3,4-dihydroxy-5-(3-methylbut-2-enyl)phenyl]-1-(2,4-dihydroxyphenyl)prop-2-en-1-one	ESR1
11267805	(E)-3-[3,4-dihydroxy-5-(3-methylbut-2-enyl)phenyl]-1-(2,4-dihydroxyphenyl)prop-2-en-1-one	ESR2
11267805	(E)-3-[3,4-dihydroxy-5-(3-methylbut-2-enyl)phenyl]-1-(2,4-dihydroxyphenyl)prop-2-en-1-one	ESRRA
11267805	(E)-3-[3,4-dihydroxy-5-(3-methylbut-2-enyl)phenyl]-1-(2,4-dihydroxyphenyl)prop-2-en-1-one	ESRRB
11267805	(E)-3-[3,4-dihydroxy-5-(3-methylbut-2-enyl)phenyl]-1-	F3

	(2,4-dihydroxyphenyl)prop-2-en-1-one	
11267805	(E)-3-[3,4-dihydroxy-5-(3-methylbut-2-enyl)phenyl]-1-(2,4-dihydroxyphenyl)prop-2-en-1-one	FLT3
11267805	(E)-3-[3,4-dihydroxy-5-(3-methylbut-2-enyl)phenyl]-1-(2,4-dihydroxyphenyl)prop-2-en-1-one	GRM2
11267805	(E)-3-[3,4-dihydroxy-5-(3-methylbut-2-enyl)phenyl]-1-(2,4-dihydroxyphenyl)prop-2-en-1-one	GSK3A
11267805	(E)-3-[3,4-dihydroxy-5-(3-methylbut-2-enyl)phenyl]-1-(2,4-dihydroxyphenyl)prop-2-en-1-one	HDAC1
11267805	(E)-3-[3,4-dihydroxy-5-(3-methylbut-2-enyl)phenyl]-1-(2,4-dihydroxyphenyl)prop-2-en-1-one	HDAC2
11267805	(E)-3-[3,4-dihydroxy-5-(3-methylbut-2-enyl)phenyl]-1-(2,4-dihydroxyphenyl)prop-2-en-1-one	HDAC3
11267805	(E)-3-[3,4-dihydroxy-5-(3-methylbut-2-enyl)phenyl]-1-(2,4-dihydroxyphenyl)prop-2-en-1-one	HDAC4
11267805	(E)-3-[3,4-dihydroxy-5-(3-methylbut-2-enyl)phenyl]-1-(2,4-dihydroxyphenyl)prop-2-en-1-one	HDAC5
11267805	(E)-3-[3,4-dihydroxy-5-(3-methylbut-2-enyl)phenyl]-1-(2,4-dihydroxyphenyl)prop-2-en-1-one	HDAC6
11267805	(E)-3-[3,4-dihydroxy-5-(3-methylbut-2-enyl)phenyl]-1-(2,4-dihydroxyphenyl)prop-2-en-1-one	HDAC7
11267805	(E)-3-[3,4-dihydroxy-5-(3-methylbut-2-enyl)phenyl]-1-	HDAC8

	(2,4-dihydroxyphenyl)prop-2-en-1-one	
11267805	(E)-3-[3,4-dihydroxy-5-(3-methylbut-2-enyl)phenyl]-1-(2,4-dihydroxyphenyl)prop-2-en-1-one	HDAC9
11267805	(E)-3-[3,4-dihydroxy-5-(3-methylbut-2-enyl)phenyl]-1-(2,4-dihydroxyphenyl)prop-2-en-1-one	HPGDS
11267805	(E)-3-[3,4-dihydroxy-5-(3-methylbut-2-enyl)phenyl]-1-(2,4-dihydroxyphenyl)prop-2-en-1-one	HSD17B1
11267805	(E)-3-[3,4-dihydroxy-5-(3-methylbut-2-enyl)phenyl]-1-(2,4-dihydroxyphenyl)prop-2-en-1-one	HSD17B2
11267805	(E)-3-[3,4-dihydroxy-5-(3-methylbut-2-enyl)phenyl]-1-(2,4-dihydroxyphenyl)prop-2-en-1-one	HSP90AA1
11267805	(E)-3-[3,4-dihydroxy-5-(3-methylbut-2-enyl)phenyl]-1-(2,4-dihydroxyphenyl)prop-2-en-1-one	ILK
11267805	(E)-3-[3,4-dihydroxy-5-(3-methylbut-2-enyl)phenyl]-1-(2,4-dihydroxyphenyl)prop-2-en-1-one	MAOA
11267805	(E)-3-[3,4-dihydroxy-5-(3-methylbut-2-enyl)phenyl]-1-(2,4-dihydroxyphenyl)prop-2-en-1-one	MAOB
11267805	(E)-3-[3,4-dihydroxy-5-(3-methylbut-2-enyl)phenyl]-1-(2,4-dihydroxyphenyl)prop-2-en-1-one	MMP13
11267805	(E)-3-[3,4-dihydroxy-5-(3-methylbut-2-enyl)phenyl]-1-(2,4-dihydroxyphenyl)prop-2-en-1-one	MMP14
11267805	(E)-3-[3,4-dihydroxy-5-(3-methylbut-2-enyl)phenyl]-1-	MMP3

	(2,4-dihydroxyphenyl)prop-2-en-1-one	
11267805	(E)-3-[3,4-dihydroxy-5-(3-methylbut-2-enyl)phenyl]-1-(2,4-dihydroxyphenyl)prop-2-en-1-one	MMP8
11267805	(E)-3-[3,4-dihydroxy-5-(3-methylbut-2-enyl)phenyl]-1-(2,4-dihydroxyphenyl)prop-2-en-1-one	NOS2
11267805	(E)-3-[3,4-dihydroxy-5-(3-methylbut-2-enyl)phenyl]-1-(2,4-dihydroxyphenyl)prop-2-en-1-one	NPY5R
11267805	(E)-3-[3,4-dihydroxy-5-(3-methylbut-2-enyl)phenyl]-1-(2,4-dihydroxyphenyl)prop-2-en-1-one	ODC1
11267805	(E)-3-[3,4-dihydroxy-5-(3-methylbut-2-enyl)phenyl]-1-(2,4-dihydroxyphenyl)prop-2-en-1-one	PDK1
11267805	(E)-3-[3,4-dihydroxy-5-(3-methylbut-2-enyl)phenyl]-1-(2,4-dihydroxyphenyl)prop-2-en-1-one	PDPK1
11267805	(E)-3-[3,4-dihydroxy-5-(3-methylbut-2-enyl)phenyl]-1-(2,4-dihydroxyphenyl)prop-2-en-1-one	PLK1
11267805	(E)-3-[3,4-dihydroxy-5-(3-methylbut-2-enyl)phenyl]-1-(2,4-dihydroxyphenyl)prop-2-en-1-one	PRKCZ
11267805	(E)-3-[3,4-dihydroxy-5-(3-methylbut-2-enyl)phenyl]-1-(2,4-dihydroxyphenyl)prop-2-en-1-one	PTGES
11267805	(E)-3-[3,4-dihydroxy-5-(3-methylbut-2-enyl)phenyl]-1-(2,4-dihydroxyphenyl)prop-2-en-1-one	PTGS1
11267805	(E)-3-[3,4-dihydroxy-5-(3-methylbut-2-enyl)phenyl]-1-	PTGS2

	(2,4-dihydroxyphenyl)prop-2-en-1-one	
11267805	(E)-3-[3,4-dihydroxy-5-(3-methylbut-2-enyl)phenyl]-1-(2,4-dihydroxyphenyl)prop-2-en-1-one	PTPN1
11267805	(E)-3-[3,4-dihydroxy-5-(3-methylbut-2-enyl)phenyl]-1-(2,4-dihydroxyphenyl)prop-2-en-1-one	QPCT
11267805	(E)-3-[3,4-dihydroxy-5-(3-methylbut-2-enyl)phenyl]-1-(2,4-dihydroxyphenyl)prop-2-en-1-one	RAF1
11267805	(E)-3-[3,4-dihydroxy-5-(3-methylbut-2-enyl)phenyl]-1-(2,4-dihydroxyphenyl)prop-2-en-1-one	ROCK2
11267805	(E)-3-[3,4-dihydroxy-5-(3-methylbut-2-enyl)phenyl]-1-(2,4-dihydroxyphenyl)prop-2-en-1-one	RPS6KB1
11267805	(E)-3-[3,4-dihydroxy-5-(3-methylbut-2-enyl)phenyl]-1-(2,4-dihydroxyphenyl)prop-2-en-1-one	SERPINE1
11267805	(E)-3-[3,4-dihydroxy-5-(3-methylbut-2-enyl)phenyl]-1-(2,4-dihydroxyphenyl)prop-2-en-1-one	SNCA
11267805	(E)-3-[3,4-dihydroxy-5-(3-methylbut-2-enyl)phenyl]-1-(2,4-dihydroxyphenyl)prop-2-en-1-one	TAS2R31
11267805	(E)-3-[3,4-dihydroxy-5-(3-methylbut-2-enyl)phenyl]-1-(2,4-dihydroxyphenyl)prop-2-en-1-one	TERT
11267805	(E)-3-[3,4-dihydroxy-5-(3-methylbut-2-enyl)phenyl]-1-(2,4-dihydroxyphenyl)prop-2-en-1-one	TNF
11267805	(E)-3-[3,4-dihydroxy-5-(3-methylbut-2-enyl)phenyl]-1-	TRPV1

	(2,4-dihydroxyphenyl)prop-2-en-1-one	
11267805	(E)-3-[3,4-dihydroxy-5-(3-methylbut-2-enyl)phenyl]-1-(2,4-dihydroxyphenyl)prop-2-en-1-one	TUBB1
11267805	(E)-3-[3,4-dihydroxy-5-(3-methylbut-2-enyl)phenyl]-1-(2,4-dihydroxyphenyl)prop-2-en-1-one	TYR
11267805	(E)-3-[3,4-dihydroxy-5-(3-methylbut-2-enyl)phenyl]-1-(2,4-dihydroxyphenyl)prop-2-en-1-one	WEE1
122851	licopyranocoumarin	ACVRL1
122851	licopyranocoumarin	ADORA3
122851	licopyranocoumarin	AKT1
122851	licopyranocoumarin	ALOX5AP
122851	licopyranocoumarin	AURKA
122851	licopyranocoumarin	AURKB
122851	licopyranocoumarin	CDC42BPA
122851	licopyranocoumarin	CHRM1
122851	licopyranocoumarin	CSF1R
122851	licopyranocoumarin	DHFR
122851	licopyranocoumarin	DNM1
122851	licopyranocoumarin	ERN1
122851	licopyranocoumarin	FKBP1A
122851	licopyranocoumarin	HDAC10
122851	licopyranocoumarin	HDAC11
122851	licopyranocoumarin	HDAC3
122851	licopyranocoumarin	HSP90AA1
122851	licopyranocoumarin	MAOB
122851	licopyranocoumarin	MAP2K1
122851	licopyranocoumarin	MTOR
122851	licopyranocoumarin	MTOR
122851	licopyranocoumarin	PDE10A
122851	licopyranocoumarin	PIK3CA
122851	licopyranocoumarin	PIK3CA
122851	licopyranocoumarin	PIK3CG
122851	licopyranocoumarin	PIK3R1
122851	licopyranocoumarin	ROCK1
122851	licopyranocoumarin	ROCK2
122851	licopyranocoumarin	SLC5A2
122851	licopyranocoumarin	TNKS2

122851	licopyranocoumarin	WDR5
122851	licopyranocoumarin	WNT3A
195396	3,22-Dihydroxy-11-oxo-delta(12)-oleanene-27-alpha-methoxycarbonyl-29-oic acid	ACACB
195396	3,22-Dihydroxy-11-oxo-delta(12)-oleanene-27-alpha-methoxycarbonyl-29-oic acid	ACHE
195396	3,22-Dihydroxy-11-oxo-delta(12)-oleanene-27-alpha-methoxycarbonyl-29-oic acid	ACKR3
195396	3,22-Dihydroxy-11-oxo-delta(12)-oleanene-27-alpha-methoxycarbonyl-29-oic acid	AKR1C1
195396	3,22-Dihydroxy-11-oxo-delta(12)-oleanene-27-alpha-methoxycarbonyl-29-oic acid	AKR1C2
195396	3,22-Dihydroxy-11-oxo-delta(12)-oleanene-27-alpha-methoxycarbonyl-29-oic acid	APP
195396	3,22-Dihydroxy-11-oxo-delta(12)-oleanene-27-alpha-methoxycarbonyl-29-oic acid	AR
195396	3,22-Dihydroxy-11-oxo-delta(12)-oleanene-27-alpha-methoxycarbonyl-29-oic acid	AURKA
195396	3,22-Dihydroxy-11-oxo-delta(12)-oleanene-27-alpha-methoxycarbonyl-29-oic acid	AVPR1A
195396	3,22-Dihydroxy-11-oxo-delta(12)-oleanene-27-alpha-methoxycarbonyl-29-oic acid	AVPR2
195396	3,22-Dihydroxy-11-oxo-delta(12)-oleanene-27-alpha-methoxycarbonyl-29-oic acid	C5AR1
195396	3,22-Dihydroxy-11-oxo-delta(12)-oleanene-27-alpha-methoxycarbonyl-29-oic acid	CAPN1
195396	3,22-Dihydroxy-11-oxo-delta(12)-oleanene-27-alpha-methoxycarbonyl-29-oic acid	CAPN2
195396	3,22-Dihydroxy-11-oxo-delta(12)-oleanene-27-alpha-methoxycarbonyl-29-oic acid	CASP3

195396	3,22-Dihydroxy-11-oxo-delta(12)-oleanene-27-alpha-methoxycarbonyl-29-oic acid	CASP7
195396	3,22-Dihydroxy-11-oxo-delta(12)-oleanene-27-alpha-methoxycarbonyl-29-oic acid	CASR
195396	3,22-Dihydroxy-11-oxo-delta(12)-oleanene-27-alpha-methoxycarbonyl-29-oic acid	CCKBR
195396	3,22-Dihydroxy-11-oxo-delta(12)-oleanene-27-alpha-methoxycarbonyl-29-oic acid	CCNA1
195396	3,22-Dihydroxy-11-oxo-delta(12)-oleanene-27-alpha-methoxycarbonyl-29-oic acid	CCNA2
195396	3,22-Dihydroxy-11-oxo-delta(12)-oleanene-27-alpha-methoxycarbonyl-29-oic acid	CCNT1
195396	3,22-Dihydroxy-11-oxo-delta(12)-oleanene-27-alpha-methoxycarbonyl-29-oic acid	CCR1
195396	3,22-Dihydroxy-11-oxo-delta(12)-oleanene-27-alpha-methoxycarbonyl-29-oic acid	CDK2
195396	3,22-Dihydroxy-11-oxo-delta(12)-oleanene-27-alpha-methoxycarbonyl-29-oic acid	CDK8
195396	3,22-Dihydroxy-11-oxo-delta(12)-oleanene-27-alpha-methoxycarbonyl-29-oic acid	CES2
195396	3,22-Dihydroxy-11-oxo-delta(12)-oleanene-27-alpha-methoxycarbonyl-29-oic acid	CTRC
195396	3,22-Dihydroxy-11-oxo-delta(12)-oleanene-27-alpha-methoxycarbonyl-29-oic acid	CTSB
195396	3,22-Dihydroxy-11-oxo-delta(12)-oleanene-27-alpha-methoxycarbonyl-29-oic acid	CTSK
195396	3,22-Dihydroxy-11-oxo-delta(12)-oleanene-27-alpha-methoxycarbonyl-29-oic acid	CTSL

195396	3,22-Dihydroxy-11-oxo-delta(12)-oleanene-27-alpha-methoxycarbonyl-29-oic acid	CTSS
195396	3,22-Dihydroxy-11-oxo-delta(12)-oleanene-27-alpha-methoxycarbonyl-29-oic acid	CXCR3
195396	3,22-Dihydroxy-11-oxo-delta(12)-oleanene-27-alpha-methoxycarbonyl-29-oic acid	CYP17A1
195396	3,22-Dihydroxy-11-oxo-delta(12)-oleanene-27-alpha-methoxycarbonyl-29-oic acid	CYP19A1
195396	3,22-Dihydroxy-11-oxo-delta(12)-oleanene-27-alpha-methoxycarbonyl-29-oic acid	DNTT
195396	3,22-Dihydroxy-11-oxo-delta(12)-oleanene-27-alpha-methoxycarbonyl-29-oic acid	EPAS1
195396	3,22-Dihydroxy-11-oxo-delta(12)-oleanene-27-alpha-methoxycarbonyl-29-oic acid	EPHB4
195396	3,22-Dihydroxy-11-oxo-delta(12)-oleanene-27-alpha-methoxycarbonyl-29-oic acid	EPHX2
195396	3,22-Dihydroxy-11-oxo-delta(12)-oleanene-27-alpha-methoxycarbonyl-29-oic acid	EZH2
195396	3,22-Dihydroxy-11-oxo-delta(12)-oleanene-27-alpha-methoxycarbonyl-29-oic acid	F2
195396	3,22-Dihydroxy-11-oxo-delta(12)-oleanene-27-alpha-methoxycarbonyl-29-oic acid	FLT3
195396	3,22-Dihydroxy-11-oxo-delta(12)-oleanene-27-alpha-methoxycarbonyl-29-oic acid	FNTA
195396	3,22-Dihydroxy-11-oxo-delta(12)-oleanene-27-alpha-methoxycarbonyl-29-oic acid	FNTB
195396	3,22-Dihydroxy-11-oxo-delta(12)-oleanene-27-alpha-methoxycarbonyl-29-oic acid	GSK3B

195396	3,22-Dihydroxy-11-oxo-delta(12)-oleanene-27-alpha-methoxycarbonyl-29-oic acid	HCRTR1
195396	3,22-Dihydroxy-11-oxo-delta(12)-oleanene-27-alpha-methoxycarbonyl-29-oic acid	HCRTR2
195396	3,22-Dihydroxy-11-oxo-delta(12)-oleanene-27-alpha-methoxycarbonyl-29-oic acid	HMGCR
195396	3,22-Dihydroxy-11-oxo-delta(12)-oleanene-27-alpha-methoxycarbonyl-29-oic acid	HPGDS
195396	3,22-Dihydroxy-11-oxo-delta(12)-oleanene-27-alpha-methoxycarbonyl-29-oic acid	HSD11B1
195396	3,22-Dihydroxy-11-oxo-delta(12)-oleanene-27-alpha-methoxycarbonyl-29-oic acid	HSD11B2
195396	3,22-Dihydroxy-11-oxo-delta(12)-oleanene-27-alpha-methoxycarbonyl-29-oic acid	HSD17B2
195396	3,22-Dihydroxy-11-oxo-delta(12)-oleanene-27-alpha-methoxycarbonyl-29-oic acid	HSP90AA1
195396	3,22-Dihydroxy-11-oxo-delta(12)-oleanene-27-alpha-methoxycarbonyl-29-oic acid	ICAM1
195396	3,22-Dihydroxy-11-oxo-delta(12)-oleanene-27-alpha-methoxycarbonyl-29-oic acid	IDH1
195396	3,22-Dihydroxy-11-oxo-delta(12)-oleanene-27-alpha-methoxycarbonyl-29-oic acid	IGF1R
195396	3,22-Dihydroxy-11-oxo-delta(12)-oleanene-27-alpha-methoxycarbonyl-29-oic acid	ITGAL
195396	3,22-Dihydroxy-11-oxo-delta(12)-oleanene-27-alpha-methoxycarbonyl-29-oic acid	ITGB2
195396	3,22-Dihydroxy-11-oxo-delta(12)-oleanene-27-alpha-methoxycarbonyl-29-oic acid	KDR

195396	3,22-Dihydroxy-11-oxo-delta(12)-oleanene-27-alpha-methoxycarbonyl-29-oic acid	KIF11
195396	3,22-Dihydroxy-11-oxo-delta(12)-oleanene-27-alpha-methoxycarbonyl-29-oic acid	MAPK1
195396	3,22-Dihydroxy-11-oxo-delta(12)-oleanene-27-alpha-methoxycarbonyl-29-oic acid	MAPK14
195396	3,22-Dihydroxy-11-oxo-delta(12)-oleanene-27-alpha-methoxycarbonyl-29-oic acid	MAPK8
195396	3,22-Dihydroxy-11-oxo-delta(12)-oleanene-27-alpha-methoxycarbonyl-29-oic acid	MAST3
195396	3,22-Dihydroxy-11-oxo-delta(12)-oleanene-27-alpha-methoxycarbonyl-29-oic acid	MDM2
195396	3,22-Dihydroxy-11-oxo-delta(12)-oleanene-27-alpha-methoxycarbonyl-29-oic acid	MDM4
195396	3,22-Dihydroxy-11-oxo-delta(12)-oleanene-27-alpha-methoxycarbonyl-29-oic acid	MTNR1A
195396	3,22-Dihydroxy-11-oxo-delta(12)-oleanene-27-alpha-methoxycarbonyl-29-oic acid	MTNR1B
195396	3,22-Dihydroxy-11-oxo-delta(12)-oleanene-27-alpha-methoxycarbonyl-29-oic acid	MTOR
195396	3,22-Dihydroxy-11-oxo-delta(12)-oleanene-27-alpha-methoxycarbonyl-29-oic acid	NOS2
195396	3,22-Dihydroxy-11-oxo-delta(12)-oleanene-27-alpha-methoxycarbonyl-29-oic acid	NPY5R
195396	3,22-Dihydroxy-11-oxo-delta(12)-oleanene-27-alpha-methoxycarbonyl-29-oic acid	NR1H4
195396	3,22-Dihydroxy-11-oxo-delta(12)-oleanene-27-alpha-methoxycarbonyl-29-oic acid	NR3C1

195396	3,22-Dihydroxy-11-oxo-delta(12)-oleanene-27-alpha-methoxycarbonyl-29-oic acid	NR3C2
195396	3,22-Dihydroxy-11-oxo-delta(12)-oleanene-27-alpha-methoxycarbonyl-29-oic acid	P2RX3
195396	3,22-Dihydroxy-11-oxo-delta(12)-oleanene-27-alpha-methoxycarbonyl-29-oic acid	P2RY1
195396	3,22-Dihydroxy-11-oxo-delta(12)-oleanene-27-alpha-methoxycarbonyl-29-oic acid	PDE10A
195396	3,22-Dihydroxy-11-oxo-delta(12)-oleanene-27-alpha-methoxycarbonyl-29-oic acid	PDGFRB
195396	3,22-Dihydroxy-11-oxo-delta(12)-oleanene-27-alpha-methoxycarbonyl-29-oic acid	PDPK1
195396	3,22-Dihydroxy-11-oxo-delta(12)-oleanene-27-alpha-methoxycarbonyl-29-oic acid	PFKFB3
195396	3,22-Dihydroxy-11-oxo-delta(12)-oleanene-27-alpha-methoxycarbonyl-29-oic acid	PGGT1B
195396	3,22-Dihydroxy-11-oxo-delta(12)-oleanene-27-alpha-methoxycarbonyl-29-oic acid	PGR
195396	3,22-Dihydroxy-11-oxo-delta(12)-oleanene-27-alpha-methoxycarbonyl-29-oic acid	PI4KB
195396	3,22-Dihydroxy-11-oxo-delta(12)-oleanene-27-alpha-methoxycarbonyl-29-oic acid	PIK3C3
195396	3,22-Dihydroxy-11-oxo-delta(12)-oleanene-27-alpha-methoxycarbonyl-29-oic acid	PIK3CA
195396	3,22-Dihydroxy-11-oxo-delta(12)-oleanene-27-alpha-methoxycarbonyl-29-oic acid	PIK3CB
195396	3,22-Dihydroxy-11-oxo-delta(12)-oleanene-27-alpha-methoxycarbonyl-29-oic acid	PIK3CD

195396	3,22-Dihydroxy-11-oxo-delta(12)-oleanene-27-alpha-methoxycarbonyl-29-oic acid	PIK3CG
195396	3,22-Dihydroxy-11-oxo-delta(12)-oleanene-27-alpha-methoxycarbonyl-29-oic acid	PLA2G2A
195396	3,22-Dihydroxy-11-oxo-delta(12)-oleanene-27-alpha-methoxycarbonyl-29-oic acid	PLA2G7
195396	3,22-Dihydroxy-11-oxo-delta(12)-oleanene-27-alpha-methoxycarbonyl-29-oic acid	POLB
195396	3,22-Dihydroxy-11-oxo-delta(12)-oleanene-27-alpha-methoxycarbonyl-29-oic acid	PRKCA
195396	3,22-Dihydroxy-11-oxo-delta(12)-oleanene-27-alpha-methoxycarbonyl-29-oic acid	PRKD1
195396	3,22-Dihydroxy-11-oxo-delta(12)-oleanene-27-alpha-methoxycarbonyl-29-oic acid	PRSS1
195396	3,22-Dihydroxy-11-oxo-delta(12)-oleanene-27-alpha-methoxycarbonyl-29-oic acid	PTGS2
195396	3,22-Dihydroxy-11-oxo-delta(12)-oleanene-27-alpha-methoxycarbonyl-29-oic acid	PTPN1
195396	3,22-Dihydroxy-11-oxo-delta(12)-oleanene-27-alpha-methoxycarbonyl-29-oic acid	PYGL
195396	3,22-Dihydroxy-11-oxo-delta(12)-oleanene-27-alpha-methoxycarbonyl-29-oic acid	REN
195396	3,22-Dihydroxy-11-oxo-delta(12)-oleanene-27-alpha-methoxycarbonyl-29-oic acid	S1PR3
195396	3,22-Dihydroxy-11-oxo-delta(12)-oleanene-27-alpha-methoxycarbonyl-29-oic acid	SERPINA6
195396	3,22-Dihydroxy-11-oxo-delta(12)-oleanene-27-alpha-methoxycarbonyl-29-oic acid	SHBG

195396	3,22-Dihydroxy-11-oxo-delta(12)-oleanene-27-alpha-methoxycarbonyl-29-oic acid	SLC16A1
195396	3,22-Dihydroxy-11-oxo-delta(12)-oleanene-27-alpha-methoxycarbonyl-29-oic acid	SLC6A2
195396	3,22-Dihydroxy-11-oxo-delta(12)-oleanene-27-alpha-methoxycarbonyl-29-oic acid	SLC6A3
195396	3,22-Dihydroxy-11-oxo-delta(12)-oleanene-27-alpha-methoxycarbonyl-29-oic acid	TACR2
195396	3,22-Dihydroxy-11-oxo-delta(12)-oleanene-27-alpha-methoxycarbonyl-29-oic acid	TERT
195396	3,22-Dihydroxy-11-oxo-delta(12)-oleanene-27-alpha-methoxycarbonyl-29-oic acid	TNF
195396	3,22-Dihydroxy-11-oxo-delta(12)-oleanene-27-alpha-methoxycarbonyl-29-oic acid	VHL
5317777	Glyzaglabrin	ABCB1
5317777	Glyzaglabrin	ABCG2
5317777	Glyzaglabrin	ACHE
5317777	Glyzaglabrin	ADORA1
5317777	Glyzaglabrin	ADORA2A
5317777	Glyzaglabrin	ALOX12
5317777	Glyzaglabrin	BCHE
5317777	Glyzaglabrin	CA12
5317777	Glyzaglabrin	CA2
5317777	Glyzaglabrin	CA4
5317777	Glyzaglabrin	CA7
5317777	Glyzaglabrin	CBR1
5317777	Glyzaglabrin	CDC25B
5317777	Glyzaglabrin	CDC7
5317777	Glyzaglabrin	CYP19A1
5317777	Glyzaglabrin	DUSP3
5317777	Glyzaglabrin	EGFR
5317777	Glyzaglabrin	ESR1
5317777	Glyzaglabrin	ESR2
5317777	Glyzaglabrin	ESRRA
5317777	Glyzaglabrin	ESRRB
5317777	Glyzaglabrin	HSD17B1
5317777	Glyzaglabrin	HSD17B2

5317777	Glyzaglabrin	HTR2A
5317777	Glyzaglabrin	HTR2C
5317777	Glyzaglabrin	LCK
5317777	Glyzaglabrin	MAOA
5317777	Glyzaglabrin	MAOB
5317777	Glyzaglabrin	MGAM
5317777	Glyzaglabrin	MIF
5317777	Glyzaglabrin	NOX4
5317777	Glyzaglabrin	PPARA
5317777	Glyzaglabrin	PTK6
5317777	Glyzaglabrin	PTPRS
5317777	Glyzaglabrin	SIRT1
5317777	Glyzaglabrin	TBXAS1
5317777	Glyzaglabrin	TLR9
5317777	Glyzaglabrin	TYR
5317777	Glyzaglabrin	XDH
124052	Glabridin	ADAM17
124052	Glabridin	ADAMTS4
124052	Glabridin	ADAMTS5
124052	Glabridin	ADORA1
124052	Glabridin	ADORA2A
124052	Glabridin	ADORA2B
124052	Glabridin	ALDH2
124052	Glabridin	AURKA
124052	Glabridin	AURKB
124052	Glabridin	AVPR1A
124052	Glabridin	BMP4
124052	Glabridin	BRAF
124052	Glabridin	CCNB1
124052	Glabridin	CCNB2
124052	Glabridin	CCNB3
124052	Glabridin	CDC25A
124052	Glabridin	CDC25B
124052	Glabridin	CDK1
124052	Glabridin	CDK5
124052	Glabridin	CDK5R1
124052	Glabridin	CES1
124052	Glabridin	CES2
124052	Glabridin	CHEK1
124052	Glabridin	CHRNA7
124052	Glabridin	CMA1
124052	Glabridin	COMT
124052	Glabridin	CSNK1D
124052	Glabridin	CYP11B1

124052	Glabridin	CYP11B2
124052	Glabridin	CYP17A1
124052	Glabridin	DPP4
124052	Glabridin	DRD1
124052	Glabridin	DRD2
124052	Glabridin	DRD3
124052	Glabridin	EGFR
124052	Glabridin	ELANE
124052	Glabridin	EPHX2
124052	Glabridin	ERBB2
124052	Glabridin	ERN1
124052	Glabridin	ESR1
124052	Glabridin	ESR2
124052	Glabridin	FLT3
124052	Glabridin	FNTA
124052	Glabridin	FNTB
124052	Glabridin	GCGR
124052	Glabridin	GRIN1
124052	Glabridin	GRIN2B
124052	Glabridin	GSK3A
124052	Glabridin	HDAC1
124052	Glabridin	HDAC10
124052	Glabridin	HDAC2
124052	Glabridin	HDAC3
124052	Glabridin	HDAC5
124052	Glabridin	HDAC6
124052	Glabridin	HDAC7
124052	Glabridin	HDAC8
124052	Glabridin	HSD11B1
124052	Glabridin	HSD17B1
124052	Glabridin	HSD17B2
124052	Glabridin	HSD17B3
124052	Glabridin	HSP90AA1
124052	Glabridin	HTR3A
124052	Glabridin	IDH1
124052	Glabridin	IKBKE
124052	Glabridin	KDR
124052	Glabridin	KIF11
124052	Glabridin	MAOB
124052	Glabridin	MAPK1
124052	Glabridin	MAPK14
124052	Glabridin	MERTK
124052	Glabridin	MET
124052	Glabridin	MMP1

124052	Glabridin	MMP12
124052	Glabridin	MMP13
124052	Glabridin	MMP14
124052	Glabridin	MMP3
124052	Glabridin	MMP7
124052	Glabridin	MMP8
124052	Glabridin	MPEG1
124052	Glabridin	NPY5R
124052	Glabridin	ODC1
124052	Glabridin	PARP1
124052	Glabridin	PDK1
124052	Glabridin	PFKFB3
124052	Glabridin	PLA2G1B
124052	Glabridin	PLEC
124052	Glabridin	PLK1
124052	Glabridin	PRF1
124052	Glabridin	PTPN1
124052	Glabridin	RAF1
124052	Glabridin	ROCK2
124052	Glabridin	S1PR1
124052	Glabridin	S1PR3
124052	Glabridin	SCD
124052	Glabridin	SIGMAR1
124052	Glabridin	SRC
124052	Glabridin	SREBF2
124052	Glabridin	STAT6
124052	Glabridin	STK17B
124052	Glabridin	TBK1
124052	Glabridin	TBXA2R
124052	Glabridin	TLR9
124052	Glabridin	TNNT2
124052	Glabridin	TRPV1
124052	Glabridin	TYR
124052	Glabridin	VCP
124049	Glabranin	ABCC1
124049	Glabranin	ABCG2
124049	Glabranin	ACHE
124049	Glabranin	ADORA1
124049	Glabranin	ADORA2A
124049	Glabranin	ADORA3
124049	Glabranin	AKR1C3
124049	Glabranin	ANPEP
124049	Glabranin	BACE1
124049	Glabranin	BCHE

124049	Glabranin	BCL2A1
124049	Glabranin	BLK
124049	Glabranin	BMX
124049	Glabranin	BTK
124049	Glabranin	CA1
124049	Glabranin	CA12
124049	Glabranin	CA2
124049	Glabranin	CA4
124049	Glabranin	CA7
124049	Glabranin	CASR
124049	Glabranin	CBR1
124049	Glabranin	CES1
124049	Glabranin	CES2
124049	Glabranin	COQ8B
124049	Glabranin	CSF1R
124049	Glabranin	CSK
124049	Glabranin	CTSL
124049	Glabranin	CXCR2
124049	Glabranin	CYP11B1
124049	Glabranin	CYP11B2
124049	Glabranin	CYP17A1
124049	Glabranin	CYP19A1
124049	Glabranin	CYP1B1
124049	Glabranin	EPHA1
124049	Glabranin	EPHA2
124049	Glabranin	EPHA3
124049	Glabranin	EPHA4
124049	Glabranin	EPHA5
124049	Glabranin	EPHA6
124049	Glabranin	EPHB2
124049	Glabranin	EPHB3
124049	Glabranin	ERBB2
124049	Glabranin	ERN1
124049	Glabranin	ESR1
124049	Glabranin	ESR2
124049	Glabranin	FADS1
124049	Glabranin	FAP
124049	Glabranin	FGR
124049	Glabranin	FPR1
124049	Glabranin	FYN
124049	Glabranin	GSK3B
124049	Glabranin	GSTA1
124049	Glabranin	HSD17B1
124049	Glabranin	HSD17B2

124049	Glabranin	HSD17B3
124049	Glabranin	HSP90AA1
124049	Glabranin	JAK2
124049	Glabranin	KDM4E
124049	Glabranin	KLK2
124049	Glabranin	LCK
124049	Glabranin	LYN
124049	Glabranin	MAOB
124049	Glabranin	MAP3K5
124049	Glabranin	MET
124049	Glabranin	MMP1
124049	Glabranin	MMP12
124049	Glabranin	MMP13
124049	Glabranin	MTNR1A
124049	Glabranin	MTNR1B
124049	Glabranin	ODC1
124049	Glabranin	PARP1
124049	Glabranin	PDE10A
124049	Glabranin	PDE4B
124049	Glabranin	PIM1
124049	Glabranin	PIM2
124049	Glabranin	PIM3
124049	Glabranin	PLA2G1B
124049	Glabranin	PLA2G2A
124049	Glabranin	PLA2G5
124049	Glabranin	POLB
124049	Glabranin	PPARG
124049	Glabranin	PTGS1
124049	Glabranin	PTK2B
124049	Glabranin	PTK6
124049	Glabranin	PTPN1
124049	Glabranin	QPCT
124049	Glabranin	RAF1
124049	Glabranin	ROCK1
124049	Glabranin	ROCK2
124049	Glabranin	RXRA
124049	Glabranin	SERPINE1
124049	Glabranin	SHBG
124049	Glabranin	SLC5A2
124049	Glabranin	STAT6
124049	Glabranin	SYK
124049	Glabranin	TACR3
124049	Glabranin	TAS2R31
124049	Glabranin	TXK

124049	Glabranin	TYRO3
124049	Glabranin	YES1
480774	Glabrene	ABL1
480774	Glabrene	ACHE
480774	Glabrene	ADAM17
480774	Glabrene	ADORA1
480774	Glabrene	ADORA3
480774	Glabrene	AKR1B1
480774	Glabrene	AKR1B10
480774	Glabrene	ALOX15
480774	Glabrene	ASAH1
480774	Glabrene	AVPR1A
480774	Glabrene	BACE1
480774	Glabrene	CAPN1
480774	Glabrene	CCND1
480774	Glabrene	CCNE1
480774	Glabrene	CCNE1
480774	Glabrene	CCNE1
480774	Glabrene	CCNE2
480774	Glabrene	CDK2
480774	Glabrene	CDK2
480774	Glabrene	CDK3
480774	Glabrene	CDK4
480774	Glabrene	CHEK1
480774	Glabrene	CTSD
480774	Glabrene	CYP2C19
480774	Glabrene	CYP2C9
480774	Glabrene	CYP3A4
480774	Glabrene	DRD1
480774	Glabrene	DRD3
480774	Glabrene	DRD4
480774	Glabrene	DRD5
480774	Glabrene	EGFR
480774	Glabrene	ELANE
480774	Glabrene	EPHA1
480774	Glabrene	EPHA2
480774	Glabrene	EPHA3
480774	Glabrene	EPHA4
480774	Glabrene	EPHA5
480774	Glabrene	EPHA7
480774	Glabrene	EPHA8
480774	Glabrene	EPHB1
480774	Glabrene	EPHB2
480774	Glabrene	EPHB3

480774	Glabrene	EPHB4
480774	Glabrene	ESR1
480774	Glabrene	ESR2
480774	Glabrene	ESRRA
480774	Glabrene	ESRRB
480774	Glabrene	F10
480774	Glabrene	F3
480774	Glabrene	FASN
480774	Glabrene	FLT3
480774	Glabrene	GCGR
480774	Glabrene	HDAC1
480774	Glabrene	HSD17B1
480774	Glabrene	HSD17B2
480774	Glabrene	HSP90AA1
480774	Glabrene	HTR1A
480774	Glabrene	HTR1B
480774	Glabrene	JAK2
480774	Glabrene	JAK3
480774	Glabrene	KIF11
480774	Glabrene	LCK
480774	Glabrene	MAPK14
480774	Glabrene	MMP1
480774	Glabrene	MMP2
480774	Glabrene	MMP9
480774	Glabrene	MTOR
480774	Glabrene	NAAA
480774	Glabrene	NR1H4
480774	Glabrene	PDE7A
480774	Glabrene	PDK1
480774	Glabrene	PHLPP2
480774	Glabrene	PLK1
480774	Glabrene	PRKCZ
480774	Glabrene	PTGS1
480774	Glabrene	PTPN1
480774	Glabrene	RAF1
480774	Glabrene	RARA
480774	Glabrene	RARG
480774	Glabrene	SLC6A2
480774	Glabrene	TACR2
480774	Glabrene	THRA
480774	Glabrene	THRB
480774	Glabrene	TNKS
480774	Glabrene	TNKS2
480774	Glabrene	TRPM8

480774	Glabrene	TSPO
480774	Glabrene	WEE1
336327	Medicarpin	ABCB1
336327	Medicarpin	ABL1
336327	Medicarpin	ADAM17
336327	Medicarpin	ADORA2B
336327	Medicarpin	ALK
336327	Medicarpin	ALOX12
336327	Medicarpin	ALOX15
336327	Medicarpin	ALPL
336327	Medicarpin	CA1
336327	Medicarpin	CA12
336327	Medicarpin	CA14
336327	Medicarpin	CA2
336327	Medicarpin	CA4
336327	Medicarpin	CA5A
336327	Medicarpin	CA5B
336327	Medicarpin	CA9
336327	Medicarpin	CASP3
336327	Medicarpin	CASP7
336327	Medicarpin	CCND1
336327	Medicarpin	CCND2
336327	Medicarpin	CCND3
336327	Medicarpin	CDK2
336327	Medicarpin	CDK4
336327	Medicarpin	CDK4
336327	Medicarpin	CHEK1
336327	Medicarpin	COMT
336327	Medicarpin	CTSS
336327	Medicarpin	CYP11B1
336327	Medicarpin	CYP11B2
336327	Medicarpin	CYP17A1
336327	Medicarpin	CYP19A1
336327	Medicarpin	DAO
336327	Medicarpin	DGAT1
336327	Medicarpin	EIF4A1
336327	Medicarpin	EPHA3
336327	Medicarpin	EPHB2
336327	Medicarpin	EPHB4
336327	Medicarpin	ERN1
336327	Medicarpin	ESR1
336327	Medicarpin	ESR2
336327	Medicarpin	FGFR1
336327	Medicarpin	GABRA5

336327	Medicarpin	GABRB3
336327	Medicarpin	GABRG2
336327	Medicarpin	GCK
336327	Medicarpin	GRIA1
336327	Medicarpin	GSK3B
336327	Medicarpin	GSTM2
336327	Medicarpin	GSTP1
336327	Medicarpin	HCK
336327	Medicarpin	HDAC2
336327	Medicarpin	HDAC4
336327	Medicarpin	HSD17B14
336327	Medicarpin	HSD17B2
336327	Medicarpin	HSD17B3
336327	Medicarpin	HTT
336327	Medicarpin	IRAK4
336327	Medicarpin	JAK3
336327	Medicarpin	KDM4C
336327	Medicarpin	KDR
336327	Medicarpin	KIT
336327	Medicarpin	LCK
336327	Medicarpin	LNPEP
336327	Medicarpin	MAP2K1
336327	Medicarpin	MAPKAPK2
336327	Medicarpin	MBD2
336327	Medicarpin	MET
336327	Medicarpin	MIF
336327	Medicarpin	MKNK1
336327	Medicarpin	MMP1
336327	Medicarpin	MMP3
336327	Medicarpin	MMP7
336327	Medicarpin	MMP8
336327	Medicarpin	MPEG1
336327	Medicarpin	MTOR
336327	Medicarpin	PARP1
336327	Medicarpin	PDE4A
336327	Medicarpin	PDE4B
336327	Medicarpin	PDE4C
336327	Medicarpin	PDE4D
336327	Medicarpin	PGK1
336327	Medicarpin	PIK3CA
336327	Medicarpin	PIK3CA
336327	Medicarpin	PIK3CB
336327	Medicarpin	PIK3CD
336327	Medicarpin	PIK3CG

336327	Medicarpin	PIK3R1
336327	Medicarpin	PIK3R1
336327	Medicarpin	PIM1
336327	Medicarpin	PIM2
336327	Medicarpin	PLK1
336327	Medicarpin	PRKDC
336327	Medicarpin	PTGS1
336327	Medicarpin	PTPN1
336327	Medicarpin	RAF1
336327	Medicarpin	RET
336327	Medicarpin	RPS6KA2
336327	Medicarpin	RPS6KA5
336327	Medicarpin	SRC
336327	Medicarpin	SYK
336327	Medicarpin	TUBB1
336327	Medicarpin	TUBB3
336327	Medicarpin	TYMS
336327	Medicarpin	VCP
336327	Medicarpin	WEE1
336327	Medicarpin	XIAP
5317652	Glabrone	ADRB1
5317652	Glabrone	ADRB2
5317652	Glabrone	ALDH2
5317652	Glabrone	CA7
5317652	Glabrone	GCGR
5317652	Glabrone	HSP90AA1
5317652	Glabrone	HSP90AB1
5317652	Glabrone	PDE10A
5317652	Glabrone	PDE4D
5317652	Glabrone	PTPN1
11558452	1,3-dihydroxy-9-methoxy-6-benzofurano[3,2-c]chromenone	AKR1B1
11558452	1,3-dihydroxy-9-methoxy-6-benzofurano[3,2-c]chromenone	ALOX5
11558452	1,3-dihydroxy-9-methoxy-6-benzofurano[3,2-c]chromenone	AURKA
11558452	1,3-dihydroxy-9-methoxy-6-benzofurano[3,2-c]chromenone	BRAF
11558452	1,3-dihydroxy-9-methoxy-6-benzofurano[3,2-c]chromenone	CA1
11558452	1,3-dihydroxy-9-methoxy-6-benzofurano[3,2-c]chromenone	CA12
11558452	1,3-dihydroxy-9-methoxy-6-benzofurano[3,2-c]chromenone	CA13

11558452	1,3-dihydroxy-9-methoxy-6-benzofurano[3,2-c]chromenone	CA14
11558452	1,3-dihydroxy-9-methoxy-6-benzofurano[3,2-c]chromenone	CA4
11558452	1,3-dihydroxy-9-methoxy-6-benzofurano[3,2-c]chromenone	CA5A
11558452	1,3-dihydroxy-9-methoxy-6-benzofurano[3,2-c]chromenone	CA5B
11558452	1,3-dihydroxy-9-methoxy-6-benzofurano[3,2-c]chromenone	CA6
11558452	1,3-dihydroxy-9-methoxy-6-benzofurano[3,2-c]chromenone	CA7
11558452	1,3-dihydroxy-9-methoxy-6-benzofurano[3,2-c]chromenone	CA9
11558452	1,3-dihydroxy-9-methoxy-6-benzofurano[3,2-c]chromenone	CBR1
11558452	1,3-dihydroxy-9-methoxy-6-benzofurano[3,2-c]chromenone	CCNA1
11558452	1,3-dihydroxy-9-methoxy-6-benzofurano[3,2-c]chromenone	CCNA2
11558452	1,3-dihydroxy-9-methoxy-6-benzofurano[3,2-c]chromenone	CCND1
11558452	1,3-dihydroxy-9-methoxy-6-benzofurano[3,2-c]chromenone	CDK2
11558452	1,3-dihydroxy-9-methoxy-6-benzofurano[3,2-c]chromenone	CDK4
11558452	1,3-dihydroxy-9-methoxy-6-benzofurano[3,2-c]chromenone	CYP1A2
11558452	1,3-dihydroxy-9-methoxy-6-benzofurano[3,2-c]chromenone	EGFR
11558452	1,3-dihydroxy-9-methoxy-6-benzofurano[3,2-c]chromenone	EPHB4
11558452	1,3-dihydroxy-9-methoxy-6-benzofurano[3,2-c]chromenone	ERBB2
11558452	1,3-dihydroxy-9-methoxy-6-benzofurano[3,2-c]chromenone	ESR1
11558452	1,3-dihydroxy-9-methoxy-6-benzofurano[3,2-c]chromenone	ESR2
11558452	1,3-dihydroxy-9-methoxy-6-benzofurano[3,2-c]chromenone	FEN1
11558452	1,3-dihydroxy-9-methoxy-6-benzofurano[3,2-c]chromenone	FGR
11558452	1,3-dihydroxy-9-methoxy-6-benzofurano[3,2-c]chromenone	FLT4

11558452	1,3-dihydroxy-9-methoxy-6-benzofurano[3,2-c]chromenone	GSR
11558452	1,3-dihydroxy-9-methoxy-6-benzofurano[3,2-c]chromenone	HSD17B1
11558452	1,3-dihydroxy-9-methoxy-6-benzofurano[3,2-c]chromenone	INSR
11558452	1,3-dihydroxy-9-methoxy-6-benzofurano[3,2-c]chromenone	KCNA3
11558452	1,3-dihydroxy-9-methoxy-6-benzofurano[3,2-c]chromenone	KCNA5
11558452	1,3-dihydroxy-9-methoxy-6-benzofurano[3,2-c]chromenone	KCNMA1
11558452	1,3-dihydroxy-9-methoxy-6-benzofurano[3,2-c]chromenone	LYN
11558452	1,3-dihydroxy-9-methoxy-6-benzofurano[3,2-c]chromenone	MAP3K8
11558452	1,3-dihydroxy-9-methoxy-6-benzofurano[3,2-c]chromenone	PDGFRB
11558452	1,3-dihydroxy-9-methoxy-6-benzofurano[3,2-c]chromenone	PLK4
11558452	1,3-dihydroxy-9-methoxy-6-benzofurano[3,2-c]chromenone	SQLE
11558452	1,3-dihydroxy-9-methoxy-6-benzofurano[3,2-c]chromenone	SRC
11558452	1,3-dihydroxy-9-methoxy-6-benzofurano[3,2-c]chromenone	TEK
11602329	1,3-dihydroxy-8,9-dimethoxy-6-benzofurano[3,2-c]chromenone	AKR1B1
11602329	1,3-dihydroxy-8,9-dimethoxy-6-benzofurano[3,2-c]chromenone	ALOX5
11602329	1,3-dihydroxy-8,9-dimethoxy-6-benzofurano[3,2-c]chromenone	AURKA
11602329	1,3-dihydroxy-8,9-dimethoxy-6-benzofurano[3,2-c]chromenone	BRAF
11602329	1,3-dihydroxy-8,9-dimethoxy-6-benzofurano[3,2-c]chromenone	CA12
11602329	1,3-dihydroxy-8,9-dimethoxy-6-benzofurano[3,2-c]chromenone	CA13
11602329	1,3-dihydroxy-8,9-dimethoxy-6-benzofurano[3,2-c]chromenone	CA14
11602329	1,3-dihydroxy-8,9-dimethoxy-6-benzofurano[3,2-c]chromenone	CA5A
11602329	1,3-dihydroxy-8,9-dimethoxy-6-benzofurano[3,2-c]chromenone	CA5B

11602329	1,3-dihydroxy-8,9-dimethoxy-6-benzofurano[3,2-c]chromenone	CA9
11602329	1,3-dihydroxy-8,9-dimethoxy-6-benzofurano[3,2-c]chromenone	CBR1
11602329	1,3-dihydroxy-8,9-dimethoxy-6-benzofurano[3,2-c]chromenone	CCNA1
11602329	1,3-dihydroxy-8,9-dimethoxy-6-benzofurano[3,2-c]chromenone	CCNA2
11602329	1,3-dihydroxy-8,9-dimethoxy-6-benzofurano[3,2-c]chromenone	CCND1
11602329	1,3-dihydroxy-8,9-dimethoxy-6-benzofurano[3,2-c]chromenone	CDK2
11602329	1,3-dihydroxy-8,9-dimethoxy-6-benzofurano[3,2-c]chromenone	CDK4
11602329	1,3-dihydroxy-8,9-dimethoxy-6-benzofurano[3,2-c]chromenone	CSNK2A1
11602329	1,3-dihydroxy-8,9-dimethoxy-6-benzofurano[3,2-c]chromenone	EGFR
11602329	1,3-dihydroxy-8,9-dimethoxy-6-benzofurano[3,2-c]chromenone	EPHB4
11602329	1,3-dihydroxy-8,9-dimethoxy-6-benzofurano[3,2-c]chromenone	ERBB2
11602329	1,3-dihydroxy-8,9-dimethoxy-6-benzofurano[3,2-c]chromenone	ESR1
11602329	1,3-dihydroxy-8,9-dimethoxy-6-benzofurano[3,2-c]chromenone	ESR2
11602329	1,3-dihydroxy-8,9-dimethoxy-6-benzofurano[3,2-c]chromenone	FGR
11602329	1,3-dihydroxy-8,9-dimethoxy-6-benzofurano[3,2-c]chromenone	FLT4
11602329	1,3-dihydroxy-8,9-dimethoxy-6-benzofurano[3,2-c]chromenone	GSK3B
11602329	1,3-dihydroxy-8,9-dimethoxy-6-benzofurano[3,2-c]chromenone	GSR
11602329	1,3-dihydroxy-8,9-dimethoxy-6-benzofurano[3,2-c]chromenone	INSR
11602329	1,3-dihydroxy-8,9-dimethoxy-6-benzofurano[3,2-c]chromenone	KCNA3
11602329	1,3-dihydroxy-8,9-dimethoxy-6-benzofurano[3,2-c]chromenone	KCNA5
11602329	1,3-dihydroxy-8,9-dimethoxy-6-benzofurano[3,2-c]chromenone	LYN
11602329	1,3-dihydroxy-8,9-dimethoxy-6-benzofurano[3,2-c]chromenone	MAOA

11602329	1,3-dihydroxy-8,9-dimethoxy-6-benzofurano[3,2-c]chromenone	MAP3K8
11602329	1,3-dihydroxy-8,9-dimethoxy-6-benzofurano[3,2-c]chromenone	PDGFRB
11602329	1,3-dihydroxy-8,9-dimethoxy-6-benzofurano[3,2-c]chromenone	PLK4
11602329	1,3-dihydroxy-8,9-dimethoxy-6-benzofurano[3,2-c]chromenone	SQLE
11602329	1,3-dihydroxy-8,9-dimethoxy-6-benzofurano[3,2-c]chromenone	TEK
5317300	Eurycarpin A	ABCB1
5317300	Eurycarpin A	ABCG2
5317300	Eurycarpin A	ACHE
5317300	Eurycarpin A	ADRB1
5317300	Eurycarpin A	ADRB2
5317300	Eurycarpin A	ALDH2
5317300	Eurycarpin A	AR
5317300	Eurycarpin A	BCL2L1
5317300	Eurycarpin A	CA12
5317300	Eurycarpin A	CHEK2
5317300	Eurycarpin A	CYP19A1
5317300	Eurycarpin A	EGFR
5317300	Eurycarpin A	ESR1
5317300	Eurycarpin A	ESRRA
5317300	Eurycarpin A	ESRRB
5317300	Eurycarpin A	GCGR
5317300	Eurycarpin A	HSD17B1
5317300	Eurycarpin A	HSD17B2
5317300	Eurycarpin A	HSP90AA1
5317300	Eurycarpin A	HSP90AB1
5317300	Eurycarpin A	HTR2A
5317300	Eurycarpin A	IL2
5317300	Eurycarpin A	MAOA
5317300	Eurycarpin A	MGAM
5317300	Eurycarpin A	MIF
5317300	Eurycarpin A	NOX4
5317300	Eurycarpin A	PDE10A
5317300	Eurycarpin A	PFKFB3
5317300	Eurycarpin A	PTPN1
5317300	Eurycarpin A	PTPRS
5317300	Eurycarpin A	RELA
5317300	Eurycarpin A	TBXAS1
5317300	Eurycarpin A	TYR
5317300	Eurycarpin A	XDH

23724664	(-)-Medicocarpin	ABL1
23724664	(-)-Medicocarpin	ADK
23724664	(-)-Medicocarpin	ADORA1
23724664	(-)-Medicocarpin	ADORA2A
23724664	(-)-Medicocarpin	ADORA2B
23724664	(-)-Medicocarpin	CA14
23724664	(-)-Medicocarpin	CASP3
23724664	(-)-Medicocarpin	CTSK
23724664	(-)-Medicocarpin	CTSL
23724664	(-)-Medicocarpin	CTSS
23724664	(-)-Medicocarpin	CYP19A1
23724664	(-)-Medicocarpin	EGFR
23724664	(-)-Medicocarpin	EIF4A1
23724664	(-)-Medicocarpin	GBA
23724664	(-)-Medicocarpin	HSPA5
23724664	(-)-Medicocarpin	MAP2K1
23724664	(-)-Medicocarpin	MGMT
23724664	(-)-Medicocarpin	PDGFRA
23724664	(-)-Medicocarpin	PDGFRB
23724664	(-)-Medicocarpin	PYGL
23724664	(-)-Medicocarpin	SLC28A2
23724664	(-)-Medicocarpin	SLC29A1
23724664	(-)-Medicocarpin	SLC2A1
23724664	(-)-Medicocarpin	SLC5A1
23724664	(-)-Medicocarpin	SLC5A2
23724664	(-)-Medicocarpin	SLC5A4
23724664	(-)-Medicocarpin	TYR
73205	Sigmoidin-B	ABCC1
73205	Sigmoidin-B	ABCG2
73205	Sigmoidin-B	ABL1
73205	Sigmoidin-B	ACHE
73205	Sigmoidin-B	ADAM17
73205	Sigmoidin-B	ADORA1
73205	Sigmoidin-B	ADORA2B
73205	Sigmoidin-B	ADORA3
73205	Sigmoidin-B	AKR1B10
73205	Sigmoidin-B	BACE1
73205	Sigmoidin-B	BCHE
73205	Sigmoidin-B	BCL2
73205	Sigmoidin-B	BCL2L1
73205	Sigmoidin-B	BRAF
73205	Sigmoidin-B	CA12
73205	Sigmoidin-B	CA4
73205	Sigmoidin-B	CA7

73205	Sigmoidin-B	CBR1
73205	Sigmoidin-B	CES1
73205	Sigmoidin-B	CES2
73205	Sigmoidin-B	CHEK1
73205	Sigmoidin-B	CHRNA7
73205	Sigmoidin-B	CTSL
73205	Sigmoidin-B	CYP19A1
73205	Sigmoidin-B	CYP1B1
73205	Sigmoidin-B	DNM1
73205	Sigmoidin-B	DRD1
73205	Sigmoidin-B	DRD3
73205	Sigmoidin-B	DRD4
73205	Sigmoidin-B	DRD5
73205	Sigmoidin-B	ESR1
73205	Sigmoidin-B	ESR2
73205	Sigmoidin-B	ESRRA
73205	Sigmoidin-B	ESRRB
73205	Sigmoidin-B	F3
73205	Sigmoidin-B	GCGR
73205	Sigmoidin-B	HDAC4
73205	Sigmoidin-B	HSD17B1
73205	Sigmoidin-B	HSD17B2
73205	Sigmoidin-B	HSP90AA1
73205	Sigmoidin-B	IGF1R
73205	Sigmoidin-B	INSR
73205	Sigmoidin-B	KLK2
73205	Sigmoidin-B	MAOB
73205	Sigmoidin-B	MAPK14
73205	Sigmoidin-B	MCL1
73205	Sigmoidin-B	MMP1
73205	Sigmoidin-B	MMP12
73205	Sigmoidin-B	MMP13
73205	Sigmoidin-B	NOX4
73205	Sigmoidin-B	ODC1
73205	Sigmoidin-B	PDK1
73205	Sigmoidin-B	PIM1
73205	Sigmoidin-B	PIM2
73205	Sigmoidin-B	PIM3
73205	Sigmoidin-B	PLA2G10
73205	Sigmoidin-B	PLA2G1B
73205	Sigmoidin-B	PLA2G5
73205	Sigmoidin-B	PLG
73205	Sigmoidin-B	POLB
73205	Sigmoidin-B	PPARG

73205	Sigmoidin-B	PTGS1
73205	Sigmoidin-B	PTPN1
73205	Sigmoidin-B	RET
73205	Sigmoidin-B	RXRA
73205	Sigmoidin-B	SERPINE1
73205	Sigmoidin-B	SGK1
73205	Sigmoidin-B	SHBG
73205	Sigmoidin-B	SLC5A2
73205	Sigmoidin-B	SRC
73205	Sigmoidin-B	TAS2R31
73205	Sigmoidin-B	TNF
73205	Sigmoidin-B	WEE1
928837	(2R)-7-hydroxy-2-(4-hydroxyphenyl)chroman-4-one	ABCC1
928837	(2R)-7-hydroxy-2-(4-hydroxyphenyl)chroman-4-one	ABCG2
928837	(2R)-7-hydroxy-2-(4-hydroxyphenyl)chroman-4-one	ABL1
928837	(2R)-7-hydroxy-2-(4-hydroxyphenyl)chroman-4-one	ACHE
928837	(2R)-7-hydroxy-2-(4-hydroxyphenyl)chroman-4-one	ADORA1
928837	(2R)-7-hydroxy-2-(4-hydroxyphenyl)chroman-4-one	ADORA3
928837	(2R)-7-hydroxy-2-(4-hydroxyphenyl)chroman-4-one	AKR1C3
928837	(2R)-7-hydroxy-2-(4-hydroxyphenyl)chroman-4-one	ALK
928837	(2R)-7-hydroxy-2-(4-hydroxyphenyl)chroman-4-one	ALPG
928837	(2R)-7-hydroxy-2-(4-hydroxyphenyl)chroman-4-one	ALPL
928837	(2R)-7-hydroxy-2-(4-hydroxyphenyl)chroman-4-one	APP
928837	(2R)-7-hydroxy-2-(4-hydroxyphenyl)chroman-4-one	AURKA
928837	(2R)-7-hydroxy-2-(4-hydroxyphenyl)chroman-4-one	AURKB
928837	(2R)-7-hydroxy-2-(4-hydroxyphenyl)chroman-4-one	BACE1
928837	(2R)-7-hydroxy-2-(4-hydroxyphenyl)chroman-4-one	BCHE
928837	(2R)-7-hydroxy-2-(4-hydroxyphenyl)chroman-4-one	CA1

928837	(2R)-7-hydroxy-2-(4-hydroxyphenyl)chroman-4-one	CA12
928837	(2R)-7-hydroxy-2-(4-hydroxyphenyl)chroman-4-one	CA13
928837	(2R)-7-hydroxy-2-(4-hydroxyphenyl)chroman-4-one	CA2
928837	(2R)-7-hydroxy-2-(4-hydroxyphenyl)chroman-4-one	CA3
928837	(2R)-7-hydroxy-2-(4-hydroxyphenyl)chroman-4-one	CA4
928837	(2R)-7-hydroxy-2-(4-hydroxyphenyl)chroman-4-one	CA5A
928837	(2R)-7-hydroxy-2-(4-hydroxyphenyl)chroman-4-one	CA5B
928837	(2R)-7-hydroxy-2-(4-hydroxyphenyl)chroman-4-one	CA6
928837	(2R)-7-hydroxy-2-(4-hydroxyphenyl)chroman-4-one	CA7
928837	(2R)-7-hydroxy-2-(4-hydroxyphenyl)chroman-4-one	CA9
928837	(2R)-7-hydroxy-2-(4-hydroxyphenyl)chroman-4-one	CBR1
928837	(2R)-7-hydroxy-2-(4-hydroxyphenyl)chroman-4-one	CCNB1
928837	(2R)-7-hydroxy-2-(4-hydroxyphenyl)chroman-4-one	CCNB2
928837	(2R)-7-hydroxy-2-(4-hydroxyphenyl)chroman-4-one	CCNB3
928837	(2R)-7-hydroxy-2-(4-hydroxyphenyl)chroman-4-one	CCNE1
928837	(2R)-7-hydroxy-2-(4-hydroxyphenyl)chroman-4-one	CCNE2
928837	(2R)-7-hydroxy-2-(4-hydroxyphenyl)chroman-4-one	CDK1
928837	(2R)-7-hydroxy-2-(4-hydroxyphenyl)chroman-4-one	CDK1
928837	(2R)-7-hydroxy-2-(4-hydroxyphenyl)chroman-4-one	CDK2
928837	(2R)-7-hydroxy-2-(4-hydroxyphenyl)chroman-4-one	CDK2
928837	(2R)-7-hydroxy-2-(4-hydroxyphenyl)chroman-4-one	CDK4
928837	(2R)-7-hydroxy-2-(4-hydroxyphenyl)chroman-4-one	CDK5

928837	(2R)-7-hydroxy-2-(4-hydroxyphenyl)chroman-4-one	CDK5R1
928837	(2R)-7-hydroxy-2-(4-hydroxyphenyl)chroman-4-one	CES1
928837	(2R)-7-hydroxy-2-(4-hydroxyphenyl)chroman-4-one	CES2
928837	(2R)-7-hydroxy-2-(4-hydroxyphenyl)chroman-4-one	CHEK1
928837	(2R)-7-hydroxy-2-(4-hydroxyphenyl)chroman-4-one	CISD1
928837	(2R)-7-hydroxy-2-(4-hydroxyphenyl)chroman-4-one	CYP19A1
928837	(2R)-7-hydroxy-2-(4-hydroxyphenyl)chroman-4-one	CYP1B1
928837	(2R)-7-hydroxy-2-(4-hydroxyphenyl)chroman-4-one	DAO
928837	(2R)-7-hydroxy-2-(4-hydroxyphenyl)chroman-4-one	DCTPP1
928837	(2R)-7-hydroxy-2-(4-hydroxyphenyl)chroman-4-one	EDNRA
928837	(2R)-7-hydroxy-2-(4-hydroxyphenyl)chroman-4-one	EEF2K
928837	(2R)-7-hydroxy-2-(4-hydroxyphenyl)chroman-4-one	ERN1
928837	(2R)-7-hydroxy-2-(4-hydroxyphenyl)chroman-4-one	ESR1
928837	(2R)-7-hydroxy-2-(4-hydroxyphenyl)chroman-4-one	ESR2
928837	(2R)-7-hydroxy-2-(4-hydroxyphenyl)chroman-4-one	GCGR
928837	(2R)-7-hydroxy-2-(4-hydroxyphenyl)chroman-4-one	GRK2
928837	(2R)-7-hydroxy-2-(4-hydroxyphenyl)chroman-4-one	GRM2
928837	(2R)-7-hydroxy-2-(4-hydroxyphenyl)chroman-4-one	GRM4
928837	(2R)-7-hydroxy-2-(4-hydroxyphenyl)chroman-4-one	GRM5
928837	(2R)-7-hydroxy-2-(4-hydroxyphenyl)chroman-4-one	GSK3B
928837	(2R)-7-hydroxy-2-(4-hydroxyphenyl)chroman-4-one	HNF4A
928837	(2R)-7-hydroxy-2-(4-hydroxyphenyl)chroman-4-one	HSD17B1

928837	(2R)-7-hydroxy-2-(4-hydroxyphenyl)chroman-4-one	HSD17B3
928837	(2R)-7-hydroxy-2-(4-hydroxyphenyl)chroman-4-one	IGF1R
928837	(2R)-7-hydroxy-2-(4-hydroxyphenyl)chroman-4-one	KLK1
928837	(2R)-7-hydroxy-2-(4-hydroxyphenyl)chroman-4-one	KLK2
928837	(2R)-7-hydroxy-2-(4-hydroxyphenyl)chroman-4-one	LNPEP
928837	(2R)-7-hydroxy-2-(4-hydroxyphenyl)chroman-4-one	MAOA
928837	(2R)-7-hydroxy-2-(4-hydroxyphenyl)chroman-4-one	MAOB
928837	(2R)-7-hydroxy-2-(4-hydroxyphenyl)chroman-4-one	MET
928837	(2R)-7-hydroxy-2-(4-hydroxyphenyl)chroman-4-one	MME
928837	(2R)-7-hydroxy-2-(4-hydroxyphenyl)chroman-4-one	MMP12
928837	(2R)-7-hydroxy-2-(4-hydroxyphenyl)chroman-4-one	MMP13
928837	(2R)-7-hydroxy-2-(4-hydroxyphenyl)chroman-4-one	MMP2
928837	(2R)-7-hydroxy-2-(4-hydroxyphenyl)chroman-4-one	MMP3
928837	(2R)-7-hydroxy-2-(4-hydroxyphenyl)chroman-4-one	PDPK1
928837	(2R)-7-hydroxy-2-(4-hydroxyphenyl)chroman-4-one	PGF
928837	(2R)-7-hydroxy-2-(4-hydroxyphenyl)chroman-4-one	PIK3CG
928837	(2R)-7-hydroxy-2-(4-hydroxyphenyl)chroman-4-one	PLA2G10
928837	(2R)-7-hydroxy-2-(4-hydroxyphenyl)chroman-4-one	PLA2G1B
928837	(2R)-7-hydroxy-2-(4-hydroxyphenyl)chroman-4-one	PLA2G2A
928837	(2R)-7-hydroxy-2-(4-hydroxyphenyl)chroman-4-one	PLA2G5
928837	(2R)-7-hydroxy-2-(4-hydroxyphenyl)chroman-4-one	PNMT
928837	(2R)-7-hydroxy-2-(4-hydroxyphenyl)chroman-4-one	POLB

928837	(2R)-7-hydroxy-2-(4-hydroxyphenyl)chroman-4-one	PPARG
928837	(2R)-7-hydroxy-2-(4-hydroxyphenyl)chroman-4-one	PRKACA
928837	(2R)-7-hydroxy-2-(4-hydroxyphenyl)chroman-4-one	PTGER1
928837	(2R)-7-hydroxy-2-(4-hydroxyphenyl)chroman-4-one	PTGER3
928837	(2R)-7-hydroxy-2-(4-hydroxyphenyl)chroman-4-one	PTGS1
928837	(2R)-7-hydroxy-2-(4-hydroxyphenyl)chroman-4-one	QDPR
928837	(2R)-7-hydroxy-2-(4-hydroxyphenyl)chroman-4-one	ROCK1
928837	(2R)-7-hydroxy-2-(4-hydroxyphenyl)chroman-4-one	RPS6KA3
928837	(2R)-7-hydroxy-2-(4-hydroxyphenyl)chroman-4-one	RPS6KA5
928837	(2R)-7-hydroxy-2-(4-hydroxyphenyl)chroman-4-one	RPS6KB1
928837	(2R)-7-hydroxy-2-(4-hydroxyphenyl)chroman-4-one	RXRA
928837	(2R)-7-hydroxy-2-(4-hydroxyphenyl)chroman-4-one	SERPINE1
928837	(2R)-7-hydroxy-2-(4-hydroxyphenyl)chroman-4-one	SHBG
928837	(2R)-7-hydroxy-2-(4-hydroxyphenyl)chroman-4-one	SLC5A2
928837	(2R)-7-hydroxy-2-(4-hydroxyphenyl)chroman-4-one	SRC
928837	(2R)-7-hydroxy-2-(4-hydroxyphenyl)chroman-4-one	TAS2R31
928837	(2R)-7-hydroxy-2-(4-hydroxyphenyl)chroman-4-one	VEGFA
928837	(2R)-7-hydroxy-2-(4-hydroxyphenyl)chroman-4-one	WEE1
193679	(2S)-7-hydroxy-2-(4-hydroxyphenyl)-8-(3-methylbut-2-enyl)chroman-4-one	ABCC1
193679	(2S)-7-hydroxy-2-(4-hydroxyphenyl)-8-(3-methylbut-2-enyl)chroman-4-one	ABCG2

193679	(2S)-7-hydroxy-2-(4-hydroxyphenyl)-8-(3-methylbut-2-enyl)chroman-4-one	ABL1
193679	(2S)-7-hydroxy-2-(4-hydroxyphenyl)-8-(3-methylbut-2-enyl)chroman-4-one	ACHE
193679	(2S)-7-hydroxy-2-(4-hydroxyphenyl)-8-(3-methylbut-2-enyl)chroman-4-one	ADAM17
193679	(2S)-7-hydroxy-2-(4-hydroxyphenyl)-8-(3-methylbut-2-enyl)chroman-4-one	ADORA1
193679	(2S)-7-hydroxy-2-(4-hydroxyphenyl)-8-(3-methylbut-2-enyl)chroman-4-one	ADORA2A
193679	(2S)-7-hydroxy-2-(4-hydroxyphenyl)-8-(3-methylbut-2-enyl)chroman-4-one	ADORA3
193679	(2S)-7-hydroxy-2-(4-hydroxyphenyl)-8-(3-methylbut-2-enyl)chroman-4-one	ADRA2A
193679	(2S)-7-hydroxy-2-(4-hydroxyphenyl)-8-(3-methylbut-2-enyl)chroman-4-one	ADRA2B
193679	(2S)-7-hydroxy-2-(4-hydroxyphenyl)-8-(3-methylbut-2-enyl)chroman-4-one	ADRA2C
193679	(2S)-7-hydroxy-2-(4-hydroxyphenyl)-8-(3-methylbut-2-enyl)chroman-4-one	AGTR1
193679	(2S)-7-hydroxy-2-(4-hydroxyphenyl)-8-(3-methylbut-2-enyl)chroman-4-one	AKR1B10
193679	(2S)-7-hydroxy-2-(4-hydroxyphenyl)-8-(3-methylbut-2-enyl)chroman-4-one	ANPEP
193679	(2S)-7-hydroxy-2-(4-hydroxyphenyl)-8-(3-methylbut-2-enyl)chroman-4-one	BACE1
193679	(2S)-7-hydroxy-2-(4-hydroxyphenyl)-8-(3-methylbut-2-enyl)chroman-4-one	BCHE

193679	(2S)-7-hydroxy-2-(4-hydroxyphenyl)-8-(3-methylbut-2-enyl)chroman-4-one	BCL2L1
193679	(2S)-7-hydroxy-2-(4-hydroxyphenyl)-8-(3-methylbut-2-enyl)chroman-4-one	BRAF
193679	(2S)-7-hydroxy-2-(4-hydroxyphenyl)-8-(3-methylbut-2-enyl)chroman-4-one	CA12
193679	(2S)-7-hydroxy-2-(4-hydroxyphenyl)-8-(3-methylbut-2-enyl)chroman-4-one	CA4
193679	(2S)-7-hydroxy-2-(4-hydroxyphenyl)-8-(3-methylbut-2-enyl)chroman-4-one	CA7
193679	(2S)-7-hydroxy-2-(4-hydroxyphenyl)-8-(3-methylbut-2-enyl)chroman-4-one	CBR1
193679	(2S)-7-hydroxy-2-(4-hydroxyphenyl)-8-(3-methylbut-2-enyl)chroman-4-one	CCNE1
193679	(2S)-7-hydroxy-2-(4-hydroxyphenyl)-8-(3-methylbut-2-enyl)chroman-4-one	CCNE1
193679	(2S)-7-hydroxy-2-(4-hydroxyphenyl)-8-(3-methylbut-2-enyl)chroman-4-one	CDK2
193679	(2S)-7-hydroxy-2-(4-hydroxyphenyl)-8-(3-methylbut-2-enyl)chroman-4-one	CDK3
193679	(2S)-7-hydroxy-2-(4-hydroxyphenyl)-8-(3-methylbut-2-enyl)chroman-4-one	CES1
193679	(2S)-7-hydroxy-2-(4-hydroxyphenyl)-8-(3-methylbut-2-enyl)chroman-4-one	CES2
193679	(2S)-7-hydroxy-2-(4-hydroxyphenyl)-8-(3-methylbut-2-enyl)chroman-4-one	CHEK1
193679	(2S)-7-hydroxy-2-(4-hydroxyphenyl)-8-(3-methylbut-2-enyl)chroman-4-one	CHRNA7

193679	(2S)-7-hydroxy-2-(4-hydroxyphenyl)-8-(3-methylbut-2-enyl)chroman-4-one	CLK1
193679	(2S)-7-hydroxy-2-(4-hydroxyphenyl)-8-(3-methylbut-2-enyl)chroman-4-one	CMA1
193679	(2S)-7-hydroxy-2-(4-hydroxyphenyl)-8-(3-methylbut-2-enyl)chroman-4-one	CTSD
193679	(2S)-7-hydroxy-2-(4-hydroxyphenyl)-8-(3-methylbut-2-enyl)chroman-4-one	CTSL
193679	(2S)-7-hydroxy-2-(4-hydroxyphenyl)-8-(3-methylbut-2-enyl)chroman-4-one	CYP19A1
193679	(2S)-7-hydroxy-2-(4-hydroxyphenyl)-8-(3-methylbut-2-enyl)chroman-4-one	CYP1B1
193679	(2S)-7-hydroxy-2-(4-hydroxyphenyl)-8-(3-methylbut-2-enyl)chroman-4-one	DYRK1B
193679	(2S)-7-hydroxy-2-(4-hydroxyphenyl)-8-(3-methylbut-2-enyl)chroman-4-one	EPHA1
193679	(2S)-7-hydroxy-2-(4-hydroxyphenyl)-8-(3-methylbut-2-enyl)chroman-4-one	EPHA2
193679	(2S)-7-hydroxy-2-(4-hydroxyphenyl)-8-(3-methylbut-2-enyl)chroman-4-one	EPHA3
193679	(2S)-7-hydroxy-2-(4-hydroxyphenyl)-8-(3-methylbut-2-enyl)chroman-4-one	EPHA4
193679	(2S)-7-hydroxy-2-(4-hydroxyphenyl)-8-(3-methylbut-2-enyl)chroman-4-one	EPHA5
193679	(2S)-7-hydroxy-2-(4-hydroxyphenyl)-8-(3-methylbut-2-enyl)chroman-4-one	EPHA7
193679	(2S)-7-hydroxy-2-(4-hydroxyphenyl)-8-(3-methylbut-2-enyl)chroman-4-one	EPHA8

193679	(2S)-7-hydroxy-2-(4-hydroxyphenyl)-8-(3-methylbut-2-enyl)chroman-4-one	EPHB1
193679	(2S)-7-hydroxy-2-(4-hydroxyphenyl)-8-(3-methylbut-2-enyl)chroman-4-one	EPHB2
193679	(2S)-7-hydroxy-2-(4-hydroxyphenyl)-8-(3-methylbut-2-enyl)chroman-4-one	EPHB3
193679	(2S)-7-hydroxy-2-(4-hydroxyphenyl)-8-(3-methylbut-2-enyl)chroman-4-one	EPHB4
193679	(2S)-7-hydroxy-2-(4-hydroxyphenyl)-8-(3-methylbut-2-enyl)chroman-4-one	ERBB2
193679	(2S)-7-hydroxy-2-(4-hydroxyphenyl)-8-(3-methylbut-2-enyl)chroman-4-one	ESR1
193679	(2S)-7-hydroxy-2-(4-hydroxyphenyl)-8-(3-methylbut-2-enyl)chroman-4-one	ESR2
193679	(2S)-7-hydroxy-2-(4-hydroxyphenyl)-8-(3-methylbut-2-enyl)chroman-4-one	GCGR
193679	(2S)-7-hydroxy-2-(4-hydroxyphenyl)-8-(3-methylbut-2-enyl)chroman-4-one	GSK3B
193679	(2S)-7-hydroxy-2-(4-hydroxyphenyl)-8-(3-methylbut-2-enyl)chroman-4-one	HSD17B1
193679	(2S)-7-hydroxy-2-(4-hydroxyphenyl)-8-(3-methylbut-2-enyl)chroman-4-one	HSD17B2
193679	(2S)-7-hydroxy-2-(4-hydroxyphenyl)-8-(3-methylbut-2-enyl)chroman-4-one	HSP90AA1
193679	(2S)-7-hydroxy-2-(4-hydroxyphenyl)-8-(3-methylbut-2-enyl)chroman-4-one	JAK1
193679	(2S)-7-hydroxy-2-(4-hydroxyphenyl)-8-(3-methylbut-2-enyl)chroman-4-one	JAK2

193679	(2S)-7-hydroxy-2-(4-hydroxyphenyl)-8-(3-methylbut-2-enyl)chroman-4-one	KCNA5
193679	(2S)-7-hydroxy-2-(4-hydroxyphenyl)-8-(3-methylbut-2-enyl)chroman-4-one	KDM1A
193679	(2S)-7-hydroxy-2-(4-hydroxyphenyl)-8-(3-methylbut-2-enyl)chroman-4-one	KDR
193679	(2S)-7-hydroxy-2-(4-hydroxyphenyl)-8-(3-methylbut-2-enyl)chroman-4-one	KLK1
193679	(2S)-7-hydroxy-2-(4-hydroxyphenyl)-8-(3-methylbut-2-enyl)chroman-4-one	KLK2
193679	(2S)-7-hydroxy-2-(4-hydroxyphenyl)-8-(3-methylbut-2-enyl)chroman-4-one	LCK
193679	(2S)-7-hydroxy-2-(4-hydroxyphenyl)-8-(3-methylbut-2-enyl)chroman-4-one	MAOB
193679	(2S)-7-hydroxy-2-(4-hydroxyphenyl)-8-(3-methylbut-2-enyl)chroman-4-one	MET
193679	(2S)-7-hydroxy-2-(4-hydroxyphenyl)-8-(3-methylbut-2-enyl)chroman-4-one	MMP12
193679	(2S)-7-hydroxy-2-(4-hydroxyphenyl)-8-(3-methylbut-2-enyl)chroman-4-one	MMP13
193679	(2S)-7-hydroxy-2-(4-hydroxyphenyl)-8-(3-methylbut-2-enyl)chroman-4-one	MMP15
193679	(2S)-7-hydroxy-2-(4-hydroxyphenyl)-8-(3-methylbut-2-enyl)chroman-4-one	MMP16
193679	(2S)-7-hydroxy-2-(4-hydroxyphenyl)-8-(3-methylbut-2-enyl)chroman-4-one	MMP26
193679	(2S)-7-hydroxy-2-(4-hydroxyphenyl)-8-(3-methylbut-2-enyl)chroman-4-one	MMP3

193679	(2S)-7-hydroxy-2-(4-hydroxyphenyl)-8-(3-methylbut-2-enyl)chroman-4-one	MMP8
193679	(2S)-7-hydroxy-2-(4-hydroxyphenyl)-8-(3-methylbut-2-enyl)chroman-4-one	NAAA
193679	(2S)-7-hydroxy-2-(4-hydroxyphenyl)-8-(3-methylbut-2-enyl)chroman-4-one	NEK1
193679	(2S)-7-hydroxy-2-(4-hydroxyphenyl)-8-(3-methylbut-2-enyl)chroman-4-one	NR1H4
193679	(2S)-7-hydroxy-2-(4-hydroxyphenyl)-8-(3-methylbut-2-enyl)chroman-4-one	ODC1
193679	(2S)-7-hydroxy-2-(4-hydroxyphenyl)-8-(3-methylbut-2-enyl)chroman-4-one	OPRD1
193679	(2S)-7-hydroxy-2-(4-hydroxyphenyl)-8-(3-methylbut-2-enyl)chroman-4-one	PDE7A
193679	(2S)-7-hydroxy-2-(4-hydroxyphenyl)-8-(3-methylbut-2-enyl)chroman-4-one	PITRM1
193679	(2S)-7-hydroxy-2-(4-hydroxyphenyl)-8-(3-methylbut-2-enyl)chroman-4-one	PLA2G1B
193679	(2S)-7-hydroxy-2-(4-hydroxyphenyl)-8-(3-methylbut-2-enyl)chroman-4-one	PLA2G2A
193679	(2S)-7-hydroxy-2-(4-hydroxyphenyl)-8-(3-methylbut-2-enyl)chroman-4-one	PLA2G5
193679	(2S)-7-hydroxy-2-(4-hydroxyphenyl)-8-(3-methylbut-2-enyl)chroman-4-one	PLA2G7
193679	(2S)-7-hydroxy-2-(4-hydroxyphenyl)-8-(3-methylbut-2-enyl)chroman-4-one	POLB
193679	(2S)-7-hydroxy-2-(4-hydroxyphenyl)-8-(3-methylbut-2-enyl)chroman-4-one	PPARG

193679	(2S)-7-hydroxy-2-(4-hydroxyphenyl)-8-(3-methylbut-2-enyl)chroman-4-one	PRKCZ
193679	(2S)-7-hydroxy-2-(4-hydroxyphenyl)-8-(3-methylbut-2-enyl)chroman-4-one	PTGS1
193679	(2S)-7-hydroxy-2-(4-hydroxyphenyl)-8-(3-methylbut-2-enyl)chroman-4-one	PTGS2
193679	(2S)-7-hydroxy-2-(4-hydroxyphenyl)-8-(3-methylbut-2-enyl)chroman-4-one	PTPN1
193679	(2S)-7-hydroxy-2-(4-hydroxyphenyl)-8-(3-methylbut-2-enyl)chroman-4-one	RET
193679	(2S)-7-hydroxy-2-(4-hydroxyphenyl)-8-(3-methylbut-2-enyl)chroman-4-one	RPS6KA3
193679	(2S)-7-hydroxy-2-(4-hydroxyphenyl)-8-(3-methylbut-2-enyl)chroman-4-one	RXRA
193679	(2S)-7-hydroxy-2-(4-hydroxyphenyl)-8-(3-methylbut-2-enyl)chroman-4-one	SHBG
193679	(2S)-7-hydroxy-2-(4-hydroxyphenyl)-8-(3-methylbut-2-enyl)chroman-4-one	SLC5A2
193679	(2S)-7-hydroxy-2-(4-hydroxyphenyl)-8-(3-methylbut-2-enyl)chroman-4-one	TAOK1
193679	(2S)-7-hydroxy-2-(4-hydroxyphenyl)-8-(3-methylbut-2-enyl)chroman-4-one	TAOK3
193679	(2S)-7-hydroxy-2-(4-hydroxyphenyl)-8-(3-methylbut-2-enyl)chroman-4-one	TAS2R31
193679	(2S)-7-hydroxy-2-(4-hydroxyphenyl)-8-(3-methylbut-2-enyl)chroman-4-one	TBXA2R
193679	(2S)-7-hydroxy-2-(4-hydroxyphenyl)-8-(3-methylbut-2-enyl)chroman-4-one	TNKS

193679	(2S)-7-hydroxy-2-(4-hydroxyphenyl)-8-(3-methylbut-2-enyl)chroman-4-one	TNKS2
193679	(2S)-7-hydroxy-2-(4-hydroxyphenyl)-8-(3-methylbut-2-enyl)chroman-4-one	WEE1
124050	Isoglycyrol	AGTR1
124050	Isoglycyrol	APP
124050	Isoglycyrol	CA1
124050	Isoglycyrol	CA2
124050	Isoglycyrol	CCNB1
124050	Isoglycyrol	CCNB2
124050	Isoglycyrol	CCNB3
124050	Isoglycyrol	CDK1
124050	Isoglycyrol	CES1
124050	Isoglycyrol	CES2
124050	Isoglycyrol	CHEK1
124050	Isoglycyrol	CHRNA7
124050	Isoglycyrol	COMT
124050	Isoglycyrol	EGFR
124050	Isoglycyrol	ERN1
124050	Isoglycyrol	FLT3
124050	Isoglycyrol	HDAC10
124050	Isoglycyrol	HDAC11
124050	Isoglycyrol	HDAC2
124050	Isoglycyrol	HDAC3
124050	Isoglycyrol	HDAC4
124050	Isoglycyrol	HDAC5
124050	Isoglycyrol	HDAC7
124050	Isoglycyrol	HDAC9
124050	Isoglycyrol	HPGDS
124050	Isoglycyrol	HSD17B1
124050	Isoglycyrol	HTR3A
124050	Isoglycyrol	IMPDH2
124050	Isoglycyrol	KCNA3
124050	Isoglycyrol	KDR
124050	Isoglycyrol	MET
124050	Isoglycyrol	MMP1
124050	Isoglycyrol	MMP13
124050	Isoglycyrol	MMP2
124050	Isoglycyrol	MMP8
124050	Isoglycyrol	MMP9
124050	Isoglycyrol	ODC1
124050	Isoglycyrol	P2RX3

124050	Isoglycyrol	PDE4A
124050	Isoglycyrol	PDE4B
124050	Isoglycyrol	PDE4C
124050	Isoglycyrol	PDE4D
124050	Isoglycyrol	PLK1
124050	Isoglycyrol	PPARG
124050	Isoglycyrol	PTPN1
124050	Isoglycyrol	ROCK2
124050	Isoglycyrol	SCN9A
124050	Isoglycyrol	SRC
5318585	Isolicoflavonol	ABCB1
5318585	Isolicoflavonol	ABCC1
5318585	Isolicoflavonol	ABCG2
5318585	Isolicoflavonol	ACHE
5318585	Isolicoflavonol	ADORA1
5318585	Isolicoflavonol	ADORA2A
5318585	Isolicoflavonol	ADORA3
5318585	Isolicoflavonol	AHR
5318585	Isolicoflavonol	AKR1A1
5318585	Isolicoflavonol	AKR1B1
5318585	Isolicoflavonol	AKR1B10
5318585	Isolicoflavonol	AKR1C1
5318585	Isolicoflavonol	AKR1C2
5318585	Isolicoflavonol	AKR1C3
5318585	Isolicoflavonol	AKR1C4
5318585	Isolicoflavonol	AKT1
5318585	Isolicoflavonol	ALK
5318585	Isolicoflavonol	ALOX12
5318585	Isolicoflavonol	ALOX15
5318585	Isolicoflavonol	ALOX5
5318585	Isolicoflavonol	AMY1A
5318585	Isolicoflavonol	APEX1
5318585	Isolicoflavonol	AR
5318585	Isolicoflavonol	ARG1
5318585	Isolicoflavonol	AURKB
5318585	Isolicoflavonol	AVPR2
5318585	Isolicoflavonol	AXL
5318585	Isolicoflavonol	BACE1
5318585	Isolicoflavonol	BCHE
5318585	Isolicoflavonol	BCL2
5318585	Isolicoflavonol	BCL2L1
5318585	Isolicoflavonol	CA1
5318585	Isolicoflavonol	CA12
5318585	Isolicoflavonol	CA13

5318585	Isolicoflavonol	CA2
5318585	Isolicoflavonol	CA3
5318585	Isolicoflavonol	CA4
5318585	Isolicoflavonol	CA5A
5318585	Isolicoflavonol	CA7
5318585	Isolicoflavonol	CA9
5318585	Isolicoflavonol	CAMK2B
5318585	Isolicoflavonol	CCNB1
5318585	Isolicoflavonol	CCNB2
5318585	Isolicoflavonol	CCNB3
5318585	Isolicoflavonol	CDK1
5318585	Isolicoflavonol	CDK1
5318585	Isolicoflavonol	CDK2
5318585	Isolicoflavonol	CDK5
5318585	Isolicoflavonol	CDK5R1
5318585	Isolicoflavonol	CDK6
5318585	Isolicoflavonol	CFTR
5318585	Isolicoflavonol	CSNK2A1
5318585	Isolicoflavonol	CYP19A1
5318585	Isolicoflavonol	CYP1B1
5318585	Isolicoflavonol	DAPK1
5318585	Isolicoflavonol	DRD4
5318585	Isolicoflavonol	EGFR
5318585	Isolicoflavonol	ESR1
5318585	Isolicoflavonol	ESR2
5318585	Isolicoflavonol	ESRRA
5318585	Isolicoflavonol	F2
5318585	Isolicoflavonol	FLT3
5318585	Isolicoflavonol	GCGR
5318585	Isolicoflavonol	GLO1
5318585	Isolicoflavonol	GSK3B
5318585	Isolicoflavonol	HSD17B1
5318585	Isolicoflavonol	HSD17B2
5318585	Isolicoflavonol	HSP90AA1
5318585	Isolicoflavonol	HSP90AB1
5318585	Isolicoflavonol	IGF1R
5318585	Isolicoflavonol	INSR
5318585	Isolicoflavonol	KDM4E
5318585	Isolicoflavonol	KDR
5318585	Isolicoflavonol	MAOA
5318585	Isolicoflavonol	MAPT
5318585	Isolicoflavonol	MPG
5318585	Isolicoflavonol	MPO
5318585	Isolicoflavonol	MYLK

5318585	Isolicoflavonol	NAE1
5318585	Isolicoflavonol	NEK2
5318585	Isolicoflavonol	NEK6
5318585	Isolicoflavonol	NOX4
5318585	Isolicoflavonol	NUAK1
5318585	Isolicoflavonol	OPRD1
5318585	Isolicoflavonol	PDE5A
5318585	Isolicoflavonol	PFKFB3
5318585	Isolicoflavonol	PIK3R1
5318585	Isolicoflavonol	PIM1
5318585	Isolicoflavonol	PKN1
5318585	Isolicoflavonol	PLA2G1B
5318585	Isolicoflavonol	PLK1
5318585	Isolicoflavonol	PTK2
5318585	Isolicoflavonol	PTPN1
5318585	Isolicoflavonol	PYGL
5318585	Isolicoflavonol	RAF1
5318585	Isolicoflavonol	SLC22A12
5318585	Isolicoflavonol	SRC
5318585	Isolicoflavonol	SYK
5318585	Isolicoflavonol	TNKS
5318585	Isolicoflavonol	TNKS2
5318585	Isolicoflavonol	TOP2A
5318585	Isolicoflavonol	TTR
5318585	Isolicoflavonol	TYR
5318585	Isolicoflavonol	XDH
5281654	isorhamnetin	ABCB1
5281654	isorhamnetin	ABCC1
5281654	isorhamnetin	ABCG2
5281654	isorhamnetin	ACHE
5281654	isorhamnetin	ADORA1
5281654	isorhamnetin	ADORA2A
5281654	isorhamnetin	AHR
5281654	isorhamnetin	AKR1A1
5281654	isorhamnetin	AKR1B1
5281654	isorhamnetin	AKR1B10
5281654	isorhamnetin	AKR1C1
5281654	isorhamnetin	AKR1C2
5281654	isorhamnetin	AKR1C3
5281654	isorhamnetin	AKR1C4
5281654	isorhamnetin	AKT1
5281654	isorhamnetin	ALK
5281654	isorhamnetin	ALOX12
5281654	isorhamnetin	ALOX15

5281654	isorhamnetin	ALOX5
5281654	isorhamnetin	APEX1
5281654	isorhamnetin	APP
5281654	isorhamnetin	ARG1
5281654	isorhamnetin	AURKB
5281654	isorhamnetin	AVPR2
5281654	isorhamnetin	AXL
5281654	isorhamnetin	BACE1
5281654	isorhamnetin	CA1
5281654	isorhamnetin	CA12
5281654	isorhamnetin	CA13
5281654	isorhamnetin	CA14
5281654	isorhamnetin	CA2
5281654	isorhamnetin	CA3
5281654	isorhamnetin	CA4
5281654	isorhamnetin	CA5A
5281654	isorhamnetin	CA6
5281654	isorhamnetin	CA7
5281654	isorhamnetin	CA9
5281654	isorhamnetin	CAMK2B
5281654	isorhamnetin	CCNB1
5281654	isorhamnetin	CCNB2
5281654	isorhamnetin	CCNB3
5281654	isorhamnetin	CD38
5281654	isorhamnetin	CDK1
5281654	isorhamnetin	CDK1
5281654	isorhamnetin	CDK2
5281654	isorhamnetin	CDK5
5281654	isorhamnetin	CDK5R1
5281654	isorhamnetin	CDK6
5281654	isorhamnetin	CSNK2A1
5281654	isorhamnetin	CXCR1
5281654	isorhamnetin	CYP19A1
5281654	isorhamnetin	CYP1B1
5281654	isorhamnetin	DAPK1
5281654	isorhamnetin	DRD4
5281654	isorhamnetin	EGFR
5281654	isorhamnetin	ESR2
5281654	isorhamnetin	ESRRA
5281654	isorhamnetin	F2
5281654	isorhamnetin	FLT3
5281654	isorhamnetin	GLO1
5281654	isorhamnetin	GPR35
5281654	isorhamnetin	GSK3B

5281654	isorhamnetin	HSD17B1
5281654	isorhamnetin	HSD17B2
5281654	isorhamnetin	IGF1R
5281654	isorhamnetin	INSR
5281654	isorhamnetin	KDM4E
5281654	isorhamnetin	KDR
5281654	isorhamnetin	MAOA
5281654	isorhamnetin	MAPT
5281654	isorhamnetin	MCL1
5281654	isorhamnetin	MET
5281654	isorhamnetin	MMP12
5281654	isorhamnetin	MMP13
5281654	isorhamnetin	MMP2
5281654	isorhamnetin	MMP3
5281654	isorhamnetin	MMP9
5281654	isorhamnetin	MPG
5281654	isorhamnetin	MPO
5281654	isorhamnetin	MYLK
5281654	isorhamnetin	NEK2
5281654	isorhamnetin	NEK6
5281654	isorhamnetin	NOX4
5281654	isorhamnetin	NUAK1
5281654	isorhamnetin	PARP1
5281654	isorhamnetin	PIK3CG
5281654	isorhamnetin	PIK3R1
5281654	isorhamnetin	PIM1
5281654	isorhamnetin	PKN1
5281654	isorhamnetin	PLA2G1B
5281654	isorhamnetin	PLG
5281654	isorhamnetin	PLK1
5281654	isorhamnetin	PTK2
5281654	isorhamnetin	PTPRS
5281654	isorhamnetin	PYGL
5281654	isorhamnetin	SLC22A12
5281654	isorhamnetin	SRC
5281654	isorhamnetin	SYK
5281654	isorhamnetin	TERT
5281654	isorhamnetin	TNKS2
5281654	isorhamnetin	TOP2A
5281654	isorhamnetin	TTR
5281654	isorhamnetin	TYR
5281654	isorhamnetin	XDH
3764	HMO	ABCB1
3764	HMO	ABCG2

3764	HMO	ABL1
3764	HMO	ACHE
3764	HMO	ADAM17
3764	HMO	ADORA1
3764	HMO	ADORA2A
3764	HMO	ALDH2
3764	HMO	ALOX12
3764	HMO	ALOX15
3764	HMO	ALPG
3764	HMO	ALPL
3764	HMO	AURKA
3764	HMO	BACE1
3764	HMO	BMP1
3764	HMO	CA1
3764	HMO	CA12
3764	HMO	CA13
3764	HMO	CA14
3764	HMO	CA2
3764	HMO	CA3
3764	HMO	CA4
3764	HMO	CA5A
3764	HMO	CA5B
3764	HMO	CA6
3764	HMO	CA7
3764	HMO	CA9
3764	HMO	CBR1
3764	HMO	CCNA1
3764	HMO	CCNA2
3764	HMO	CDC25A
3764	HMO	CDC25B
3764	HMO	CDK2
3764	HMO	CDK4
3764	HMO	CHEK1
3764	HMO	CHRNA7
3764	HMO	CYP19A1
3764	HMO	DRD1
3764	HMO	EGFR
3764	HMO	EPHB2
3764	HMO	EPHB4
3764	HMO	ESR1
3764	HMO	ESR2
3764	HMO	ESRRA
3764	HMO	ESRRB
3764	HMO	EZR

3764	HMO	FGFR1
3764	HMO	FLT1
3764	HMO	GRIA1
3764	HMO	GRM4
3764	HMO	GSK3A
3764	HMO	HCK
3764	HMO	HDAC1
3764	HMO	HDAC10
3764	HMO	HDAC11
3764	HMO	HDAC3
3764	HMO	HDAC6
3764	HMO	HSD17B1
3764	HMO	HSD17B2
3764	HMO	HTR2A
3764	HMO	HTR2C
3764	HMO	IL2
3764	HMO	KDM1A
3764	HMO	MAOA
3764	HMO	MAOB
3764	HMO	MAP2K1
3764	HMO	MAPKAPK2
3764	HMO	MARK1
3764	HMO	MGAM
3764	HMO	MIF
3764	HMO	MMP1
3764	HMO	MMP14
3764	HMO	MMP16
3764	HMO	MMP25
3764	HMO	MMP8
3764	HMO	NCOR2
3764	HMO	NOX4
3764	HMO	PDE4A
3764	HMO	PDE4B
3764	HMO	PDE4C
3764	HMO	PDPK1
3764	HMO	PFKFB3
3764	HMO	PI4KB
3764	HMO	PIM2
3764	HMO	PLAA
3764	HMO	PON1
3764	HMO	PPARA
3764	HMO	PTGS1
3764	HMO	PTK6
3764	HMO	PTPN1

3764	HMO	PTPRS
3764	HMO	RET
3764	HMO	ROCK1
3764	HMO	RPS6KA2
3764	HMO	RPS6KB1
3764	HMO	SLC6A2
3764	HMO	SLC9A1
3764	HMO	TBXAS1
3764	HMO	TLR9
3764	HMO	TRPM8
3764	HMO	TYR
3764	HMO	WEE1
3764	HMO	XDH
480873	1-Methoxyphaseollidin	ABL1
480873	1-Methoxyphaseollidin	ADAM17
480873	1-Methoxyphaseollidin	ADORA2B
480873	1-Methoxyphaseollidin	AGTR1
480873	1-Methoxyphaseollidin	AKR1B10
480873	1-Methoxyphaseollidin	AR
480873	1-Methoxyphaseollidin	AURKA
480873	1-Methoxyphaseollidin	AURKB
480873	1-Methoxyphaseollidin	AVPR1A
480873	1-Methoxyphaseollidin	BACE1
480873	1-Methoxyphaseollidin	BRAF
480873	1-Methoxyphaseollidin	CA14
480873	1-Methoxyphaseollidin	CCNB1
480873	1-Methoxyphaseollidin	CCND1
480873	1-Methoxyphaseollidin	CCNE1
480873	1-Methoxyphaseollidin	CCNE1
480873	1-Methoxyphaseollidin	CCNE1
480873	1-Methoxyphaseollidin	CCNE2
480873	1-Methoxyphaseollidin	CDK1
480873	1-Methoxyphaseollidin	CDK2
480873	1-Methoxyphaseollidin	CDK2
480873	1-Methoxyphaseollidin	CDK3
480873	1-Methoxyphaseollidin	CDK4
480873	1-Methoxyphaseollidin	CFD
480873	1-Methoxyphaseollidin	CHEK1
480873	1-Methoxyphaseollidin	CLK1
480873	1-Methoxyphaseollidin	CMA1
480873	1-Methoxyphaseollidin	CSF1R
480873	1-Methoxyphaseollidin	CSNK1G1
480873	1-Methoxyphaseollidin	CTSD
480873	1-Methoxyphaseollidin	CTSK

480873	1-Methoxyphaseollidin	CYP19A1
480873	1-Methoxyphaseollidin	DNM1
480873	1-Methoxyphaseollidin	DRD1
480873	1-Methoxyphaseollidin	DYRK1B
480873	1-Methoxyphaseollidin	EPHA1
480873	1-Methoxyphaseollidin	EPHA2
480873	1-Methoxyphaseollidin	EPHA3
480873	1-Methoxyphaseollidin	EPHA4
480873	1-Methoxyphaseollidin	EPHA5
480873	1-Methoxyphaseollidin	EPHA7
480873	1-Methoxyphaseollidin	EPHA8
480873	1-Methoxyphaseollidin	EPHB1
480873	1-Methoxyphaseollidin	EPHB2
480873	1-Methoxyphaseollidin	EPHB3
480873	1-Methoxyphaseollidin	EPHB4
480873	1-Methoxyphaseollidin	ESR1
480873	1-Methoxyphaseollidin	ESR2
480873	1-Methoxyphaseollidin	GCGR
480873	1-Methoxyphaseollidin	GRK2
480873	1-Methoxyphaseollidin	HDAC1
480873	1-Methoxyphaseollidin	HDAC2
480873	1-Methoxyphaseollidin	HDAC3
480873	1-Methoxyphaseollidin	HDAC4
480873	1-Methoxyphaseollidin	HDAC5
480873	1-Methoxyphaseollidin	HDAC6
480873	1-Methoxyphaseollidin	HDAC7
480873	1-Methoxyphaseollidin	HDAC8
480873	1-Methoxyphaseollidin	HSD11B1
480873	1-Methoxyphaseollidin	HSD17B2
480873	1-Methoxyphaseollidin	HSP90AA1
480873	1-Methoxyphaseollidin	HTR2C
480873	1-Methoxyphaseollidin	HTR7
480873	1-Methoxyphaseollidin	IMPDH1
480873	1-Methoxyphaseollidin	IMPDH2
480873	1-Methoxyphaseollidin	KDM1A
480873	1-Methoxyphaseollidin	LCK
480873	1-Methoxyphaseollidin	MAPK1
480873	1-Methoxyphaseollidin	MAPK8
480873	1-Methoxyphaseollidin	MAPKAPK2
480873	1-Methoxyphaseollidin	MMP1
480873	1-Methoxyphaseollidin	MMP10
480873	1-Methoxyphaseollidin	MMP3
480873	1-Methoxyphaseollidin	MMP8
480873	1-Methoxyphaseollidin	OPRK1

480873	1-Methoxyphaseollidin	P2RX3
480873	1-Methoxyphaseollidin	P2RX7
480873	1-Methoxyphaseollidin	PARP1
480873	1-Methoxyphaseollidin	PDPK1
480873	1-Methoxyphaseollidin	PIK3CA
480873	1-Methoxyphaseollidin	PIK3CD
480873	1-Methoxyphaseollidin	PIM1
480873	1-Methoxyphaseollidin	PIM2
480873	1-Methoxyphaseollidin	PITRM1
480873	1-Methoxyphaseollidin	PLK1
480873	1-Methoxyphaseollidin	PTGS1
480873	1-Methoxyphaseollidin	PTPN1
480873	1-Methoxyphaseollidin	RAF1
480873	1-Methoxyphaseollidin	RET
480873	1-Methoxyphaseollidin	ROCK1
480873	1-Methoxyphaseollidin	RPS6KA1
480873	1-Methoxyphaseollidin	RPS6KB1
480873	1-Methoxyphaseollidin	SGK1
480873	1-Methoxyphaseollidin	SIGMAR1
480873	1-Methoxyphaseollidin	SLC29A1
480873	1-Methoxyphaseollidin	SPHK1
480873	1-Methoxyphaseollidin	SPHK2
480873	1-Methoxyphaseollidin	SYK
480873	1-Methoxyphaseollidin	TGM2
480873	1-Methoxyphaseollidin	TKT
480873	1-Methoxyphaseollidin	TNF
480873	1-Methoxyphaseollidin	TRPM8
480873	1-Methoxyphaseollidin	TUBB1
480873	1-Methoxyphaseollidin	TYMS
480873	1-Methoxyphaseollidin	TYR
480873	1-Methoxyphaseollidin	WEE1
5316900	Quercetin der	ABCB1
5316900	Quercetin der	ABCC1
5316900	Quercetin der	ABCG2
5316900	Quercetin der	ACHE
5316900	Quercetin der	ADORA1
5316900	Quercetin der	ADORA2A
5316900	Quercetin der	ADORA3
5316900	Quercetin der	AHR
5316900	Quercetin der	AKR1A1
5316900	Quercetin der	AKR1B1
5316900	Quercetin der	AKR1C1
5316900	Quercetin der	AKR1C2
5316900	Quercetin der	AKR1C3

5316900	Quercetin der	AKR1C4
5316900	Quercetin der	AKT1
5316900	Quercetin der	ALK
5316900	Quercetin der	ALOX12
5316900	Quercetin der	ALOX15
5316900	Quercetin der	ALOX5
5316900	Quercetin der	APEX1
5316900	Quercetin der	APP
5316900	Quercetin der	ARG1
5316900	Quercetin der	AURKB
5316900	Quercetin der	AVPR2
5316900	Quercetin der	AXL
5316900	Quercetin der	BACE1
5316900	Quercetin der	CA1
5316900	Quercetin der	CA12
5316900	Quercetin der	CA13
5316900	Quercetin der	CA14
5316900	Quercetin der	CA2
5316900	Quercetin der	CA3
5316900	Quercetin der	CA4
5316900	Quercetin der	CA5A
5316900	Quercetin der	CA6
5316900	Quercetin der	CA7
5316900	Quercetin der	CA9
5316900	Quercetin der	CAMK2B
5316900	Quercetin der	CCNB1
5316900	Quercetin der	CCNB2
5316900	Quercetin der	CCNB3
5316900	Quercetin der	CDK1
5316900	Quercetin der	CDK1
5316900	Quercetin der	CDK2
5316900	Quercetin der	CDK5
5316900	Quercetin der	CDK5R1
5316900	Quercetin der	CDK6
5316900	Quercetin der	CSNK2A1
5316900	Quercetin der	CXCR1
5316900	Quercetin der	CYP19A1
5316900	Quercetin der	CYP1B1
5316900	Quercetin der	DAPK1
5316900	Quercetin der	DRD4
5316900	Quercetin der	EGFR
5316900	Quercetin der	ESR1
5316900	Quercetin der	ESR2
5316900	Quercetin der	ESRRA

5316900	Quercetin der	F2
5316900	Quercetin der	FLT3
5316900	Quercetin der	GLO1
5316900	Quercetin der	GPR35
5316900	Quercetin der	GSK3B
5316900	Quercetin der	HSD17B1
5316900	Quercetin der	HSD17B2
5316900	Quercetin der	IGF1R
5316900	Quercetin der	INSR
5316900	Quercetin der	KDM4E
5316900	Quercetin der	KDR
5316900	Quercetin der	KIT
5316900	Quercetin der	MAOA
5316900	Quercetin der	MAPT
5316900	Quercetin der	MCL1
5316900	Quercetin der	MET
5316900	Quercetin der	MMP13
5316900	Quercetin der	MMP2
5316900	Quercetin der	MMP3
5316900	Quercetin der	MMP9
5316900	Quercetin der	MPO
5316900	Quercetin der	MYLK
5316900	Quercetin der	NEK2
5316900	Quercetin der	NEK6
5316900	Quercetin der	NOS2
5316900	Quercetin der	NOX4
5316900	Quercetin der	NUAK1
5316900	Quercetin der	OPRD1
5316900	Quercetin der	OPRM1
5316900	Quercetin der	PIK3CG
5316900	Quercetin der	PIK3R1
5316900	Quercetin der	PIM1
5316900	Quercetin der	PKN1
5316900	Quercetin der	PLA2G1B
5316900	Quercetin der	PLA2G2A
5316900	Quercetin der	PLG
5316900	Quercetin der	PLK1
5316900	Quercetin der	PTK2
5316900	Quercetin der	PYGL
5316900	Quercetin der	SLC22A12
5316900	Quercetin der	SRC
5316900	Quercetin der	ST6GAL1
5316900	Quercetin der	SYK
5316900	Quercetin der	TERT

5316900	Quercetin der	TOP2A
5316900	Quercetin der	TYR
5316900	Quercetin der	XDH
15228662	3'-Hydroxy-4'-O-Methylglabridin	ABL1
15228662	3'-Hydroxy-4'-O-Methylglabridin	ADORA1
15228662	3'-Hydroxy-4'-O-Methylglabridin	ADORA2A
15228662	3'-Hydroxy-4'-O-Methylglabridin	ADORA2B
15228662	3'-Hydroxy-4'-O-Methylglabridin	ADORA3
15228662	3'-Hydroxy-4'-O-Methylglabridin	AHCY
15228662	3'-Hydroxy-4'-O-Methylglabridin	ALPG
15228662	3'-Hydroxy-4'-O-Methylglabridin	ALPL
15228662	3'-Hydroxy-4'-O-Methylglabridin	AR
15228662	3'-Hydroxy-4'-O-Methylglabridin	ATM
15228662	3'-Hydroxy-4'-O-Methylglabridin	ATR
15228662	3'-Hydroxy-4'-O-Methylglabridin	BMP1
15228662	3'-Hydroxy-4'-O-Methylglabridin	BRAF
15228662	3'-Hydroxy-4'-O-Methylglabridin	CCND1
15228662	3'-Hydroxy-4'-O-Methylglabridin	CCNE1
15228662	3'-Hydroxy-4'-O-Methylglabridin	CCNE2
15228662	3'-Hydroxy-4'-O-Methylglabridin	CDC25A
15228662	3'-Hydroxy-4'-O-Methylglabridin	CDC25B
15228662	3'-Hydroxy-4'-O-Methylglabridin	CDC25C
15228662	3'-Hydroxy-4'-O-Methylglabridin	CDK1

15228662	3'-Hydroxy-4'-O-Methylglabridin	CDK2
15228662	3'-Hydroxy-4'-O-Methylglabridin	CDK2
15228662	3'-Hydroxy-4'-O-Methylglabridin	CDK4
15228662	3'-Hydroxy-4'-O-Methylglabridin	CDK4
15228662	3'-Hydroxy-4'-O-Methylglabridin	CHEK1
15228662	3'-Hydroxy-4'-O-Methylglabridin	CRHR1
15228662	3'-Hydroxy-4'-O-Methylglabridin	DNM1
15228662	3'-Hydroxy-4'-O-Methylglabridin	DRD1
15228662	3'-Hydroxy-4'-O-Methylglabridin	DRD3
15228662	3'-Hydroxy-4'-O-Methylglabridin	ELANE
15228662	3'-Hydroxy-4'-O-Methylglabridin	ENPP1
15228662	3'-Hydroxy-4'-O-Methylglabridin	EPHB2
15228662	3'-Hydroxy-4'-O-Methylglabridin	EPHB4
15228662	3'-Hydroxy-4'-O-Methylglabridin	ERN1
15228662	3'-Hydroxy-4'-O-Methylglabridin	ESR1
15228662	3'-Hydroxy-4'-O-Methylglabridin	ESR2
15228662	3'-Hydroxy-4'-O-Methylglabridin	ESRRG
15228662	3'-Hydroxy-4'-O-Methylglabridin	F3
15228662	3'-Hydroxy-4'-O-Methylglabridin	FGFR1
15228662	3'-Hydroxy-4'-O-Methylglabridin	FLT3
15228662	3'-Hydroxy-4'-O-Methylglabridin	GCGR
15228662	3'-Hydroxy-4'-O-Methylglabridin	GCK

15228662	3'-Hydroxy-4'-O-Methylglabridin	GRIA1
15228662	3'-Hydroxy-4'-O-Methylglabridin	GRM1
15228662	3'-Hydroxy-4'-O-Methylglabridin	GSK3A
15228662	3'-Hydroxy-4'-O-Methylglabridin	HCK
15228662	3'-Hydroxy-4'-O-Methylglabridin	HDAC1
15228662	3'-Hydroxy-4'-O-Methylglabridin	HDAC8
15228662	3'-Hydroxy-4'-O-Methylglabridin	HPGDS
15228662	3'-Hydroxy-4'-O-Methylglabridin	HSD17B1
15228662	3'-Hydroxy-4'-O-Methylglabridin	HSD17B2
15228662	3'-Hydroxy-4'-O-Methylglabridin	HSD17B3
15228662	3'-Hydroxy-4'-O-Methylglabridin	HSP90AB1
15228662	3'-Hydroxy-4'-O-Methylglabridin	HTR1A
15228662	3'-Hydroxy-4'-O-Methylglabridin	HTR2C
15228662	3'-Hydroxy-4'-O-Methylglabridin	KDM1A
15228662	3'-Hydroxy-4'-O-Methylglabridin	KDR
15228662	3'-Hydroxy-4'-O-Methylglabridin	KIT
15228662	3'-Hydroxy-4'-O-Methylglabridin	LCK
15228662	3'-Hydroxy-4'-O-Methylglabridin	LNPEP
15228662	3'-Hydroxy-4'-O-Methylglabridin	MAOB
15228662	3'-Hydroxy-4'-O-Methylglabridin	MAPT
15228662	3'-Hydroxy-4'-O-Methylglabridin	MARK1
15228662	3'-Hydroxy-4'-O-Methylglabridin	MELK

15228662	3'-Hydroxy-4'-O-Methylglabridin	MET
15228662	3'-Hydroxy-4'-O-Methylglabridin	MTOR
15228662	3'-Hydroxy-4'-O-Methylglabridin	MYLK
15228662	3'-Hydroxy-4'-O-Methylglabridin	ODC1
15228662	3'-Hydroxy-4'-O-Methylglabridin	PARP1
15228662	3'-Hydroxy-4'-O-Methylglabridin	PDE10A
15228662	3'-Hydroxy-4'-O-Methylglabridin	PDE3A
15228662	3'-Hydroxy-4'-O-Methylglabridin	PDE5A
15228662	3'-Hydroxy-4'-O-Methylglabridin	PDE7A
15228662	3'-Hydroxy-4'-O-Methylglabridin	PDK1
15228662	3'-Hydroxy-4'-O-Methylglabridin	PI4KB
15228662	3'-Hydroxy-4'-O-Methylglabridin	PIK3CB
15228662	3'-Hydroxy-4'-O-Methylglabridin	PIK3CD
15228662	3'-Hydroxy-4'-O-Methylglabridin	PIK3CG
15228662	3'-Hydroxy-4'-O-Methylglabridin	PIK3R1
15228662	3'-Hydroxy-4'-O-Methylglabridin	PIM1
15228662	3'-Hydroxy-4'-O-Methylglabridin	PIM2
15228662	3'-Hydroxy-4'-O-Methylglabridin	PIM3
15228662	3'-Hydroxy-4'-O-Methylglabridin	PLAA
15228662	3'-Hydroxy-4'-O-Methylglabridin	PLK1
15228662	3'-Hydroxy-4'-O-Methylglabridin	PRKDC
15228662	3'-Hydroxy-4'-O-Methylglabridin	PTK6

15228662	3'-Hydroxy-4'-O-Methylglabridin	PTPN1
15228662	3'-Hydroxy-4'-O-Methylglabridin	RAF1
15228662	3'-Hydroxy-4'-O-Methylglabridin	RET
15228662	3'-Hydroxy-4'-O-Methylglabridin	ROCK1
15228662	3'-Hydroxy-4'-O-Methylglabridin	RORC
15228662	3'-Hydroxy-4'-O-Methylglabridin	RPS6KA3
15228662	3'-Hydroxy-4'-O-Methylglabridin	S1PR1
15228662	3'-Hydroxy-4'-O-Methylglabridin	S1PR3
15228662	3'-Hydroxy-4'-O-Methylglabridin	SHBG
15228662	3'-Hydroxy-4'-O-Methylglabridin	SNCA
15228662	3'-Hydroxy-4'-O-Methylglabridin	SRC
15228662	3'-Hydroxy-4'-O-Methylglabridin	TBXA2R
15228662	3'-Hydroxy-4'-O-Methylglabridin	TGFBR1
15228662	3'-Hydroxy-4'-O-Methylglabridin	TRPM8
15228662	3'-Hydroxy-4'-O-Methylglabridin	VCP
15228662	3'-Hydroxy-4'-O-Methylglabridin	WEE1
15228662	3'-Hydroxy-4'-O-Methylglabridin	XDH
5318998	licochalcone a	ABCB1
5318998	licochalcone a	ABCG2
5318998	licochalcone a	ABL1
5318998	licochalcone a	ACHE
5318998	licochalcone a	ADAM10
5318998	licochalcone a	ADAM17
5318998	licochalcone a	ADORA2B
5318998	licochalcone a	ADRA1B
5318998	licochalcone a	AKR1B1
5318998	licochalcone a	ALOX5

5318998	licochalcone a	APH1A
5318998	licochalcone a	APH1B
5318998	licochalcone a	APP
5318998	licochalcone a	BACE1
5318998	licochalcone a	BCHE
5318998	licochalcone a	BMP1
5318998	licochalcone a	BRAF
5318998	licochalcone a	CAPN1
5318998	licochalcone a	CDC25A
5318998	licochalcone a	CDC25B
5318998	licochalcone a	CHRNA4
5318998	licochalcone a	CHRNA7
5318998	licochalcone a	CHRNB2
5318998	licochalcone a	CTSD
5318998	licochalcone a	CYP11B1
5318998	licochalcone a	CYP11B2
5318998	licochalcone a	CYP19A1
5318998	licochalcone a	EGFR
5318998	licochalcone a	ERN1
5318998	licochalcone a	ESRRB
5318998	licochalcone a	F3
5318998	licochalcone a	FASN
5318998	licochalcone a	FLT3
5318998	licochalcone a	GNRHR
5318998	licochalcone a	HCRTR2
5318998	licochalcone a	HDAC1
5318998	licochalcone a	HDAC10
5318998	licochalcone a	HDAC11
5318998	licochalcone a	HDAC3
5318998	licochalcone a	HDAC3
5318998	licochalcone a	HDAC4
5318998	licochalcone a	HDAC5
5318998	licochalcone a	HDAC6
5318998	licochalcone a	HDAC7
5318998	licochalcone a	HDAC8
5318998	licochalcone a	HDAC9
5318998	licochalcone a	HPGDS
5318998	licochalcone a	HTR1A
5318998	licochalcone a	ICAM1
5318998	licochalcone a	IMPDH1
5318998	licochalcone a	ITGAL
5318998	licochalcone a	ITGB2
5318998	licochalcone a	ITK
5318998	licochalcone a	JAK2

5318998	licochalcone a	KCNA3
5318998	licochalcone a	MAOA
5318998	licochalcone a	MAOB
5318998	licochalcone a	MAPK1
5318998	licochalcone a	MAPK10
5318998	licochalcone a	MAPK14
5318998	licochalcone a	MAPKAPK2
5318998	licochalcone a	MDM2
5318998	licochalcone a	MDM4
5318998	licochalcone a	MIF
5318998	licochalcone a	MMP1
5318998	licochalcone a	MMP13
5318998	licochalcone a	MMP8
5318998	licochalcone a	MMP9
5318998	licochalcone a	NAAA
5318998	licochalcone a	NCOR2
5318998	licochalcone a	NCSTN
5318998	licochalcone a	NOS2
5318998	licochalcone a	NPY5R
5318998	licochalcone a	NR1H4
5318998	licochalcone a	ODC1
5318998	licochalcone a	P2RX3
5318998	licochalcone a	PDE10A
5318998	licochalcone a	PDE4B
5318998	licochalcone a	PDGFRB
5318998	licochalcone a	PDK1
5318998	licochalcone a	PDPK1
5318998	licochalcone a	PIM1
5318998	licochalcone a	PIM2
5318998	licochalcone a	PIM3
5318998	licochalcone a	PLA2G1B
5318998	licochalcone a	PLA2G2A
5318998	licochalcone a	PRKCB
5318998	licochalcone a	PSEN1
5318998	licochalcone a	PSEN2
5318998	licochalcone a	PSENEN
5318998	licochalcone a	PTGS1
5318998	licochalcone a	PTGS2
5318998	licochalcone a	PTPN1
5318998	licochalcone a	PTPN6
5318998	licochalcone a	RAF1
5318998	licochalcone a	RET
5318998	licochalcone a	ROCK1
5318998	licochalcone a	ROCK2

5318998	licochalcone a	RPS6KB1
5318998	licochalcone a	S1PR1
5318998	licochalcone a	S1PR3
5318998	licochalcone a	SLC29A1
5318998	licochalcone a	SLC5A1
5318998	licochalcone a	SLC5A2
5318998	licochalcone a	TACR3
5318998	licochalcone a	THRA
5318998	licochalcone a	THRB
5318998	licochalcone a	TRPM8
5318998	licochalcone a	TYR
5318998	licochalcone a	VCP
15228663	3'-Methoxyglabridin	ABL1
15228663	3'-Methoxyglabridin	ADORA1
15228663	3'-Methoxyglabridin	ADORA2A
15228663	3'-Methoxyglabridin	ADORA2B
15228663	3'-Methoxyglabridin	ADORA3
15228663	3'-Methoxyglabridin	AHCY
15228663	3'-Methoxyglabridin	ALPG
15228663	3'-Methoxyglabridin	ALPL
15228663	3'-Methoxyglabridin	AR
15228663	3'-Methoxyglabridin	AURKA
15228663	3'-Methoxyglabridin	AURKB
15228663	3'-Methoxyglabridin	BCL2
15228663	3'-Methoxyglabridin	BMP1
15228663	3'-Methoxyglabridin	BRAF
15228663	3'-Methoxyglabridin	CBFB
15228663	3'-Methoxyglabridin	CCNB1
15228663	3'-Methoxyglabridin	CCNB2
15228663	3'-Methoxyglabridin	CCNB3
15228663	3'-Methoxyglabridin	CCND1
15228663	3'-Methoxyglabridin	CCND1
15228663	3'-Methoxyglabridin	CCND2
15228663	3'-Methoxyglabridin	CCND3
15228663	3'-Methoxyglabridin	CCNE1
15228663	3'-Methoxyglabridin	CCNE2
15228663	3'-Methoxyglabridin	CDC25A
15228663	3'-Methoxyglabridin	CDC25B
15228663	3'-Methoxyglabridin	CDC25C
15228663	3'-Methoxyglabridin	CDK1
15228663	3'-Methoxyglabridin	CDK1
15228663	3'-Methoxyglabridin	CDK2
15228663	3'-Methoxyglabridin	CDK2
15228663	3'-Methoxyglabridin	CDK4

15228663	3'-Methoxyglabridin	CDK4
15228663	3'-Methoxyglabridin	CDK4
15228663	3'-Methoxyglabridin	CDK5
15228663	3'-Methoxyglabridin	CDK5R1
15228663	3'-Methoxyglabridin	CHEK1
15228663	3'-Methoxyglabridin	DRD1
15228663	3'-Methoxyglabridin	DRD3
15228663	3'-Methoxyglabridin	ELANE
15228663	3'-Methoxyglabridin	ENPP1
15228663	3'-Methoxyglabridin	EP300
15228663	3'-Methoxyglabridin	EPHB2
15228663	3'-Methoxyglabridin	ERN1
15228663	3'-Methoxyglabridin	ESR2
15228663	3'-Methoxyglabridin	ESRRG
15228663	3'-Methoxyglabridin	EZR
15228663	3'-Methoxyglabridin	FGFR1
15228663	3'-Methoxyglabridin	FGFR2
15228663	3'-Methoxyglabridin	FLT3
15228663	3'-Methoxyglabridin	GRIA1
15228663	3'-Methoxyglabridin	GRM1
15228663	3'-Methoxyglabridin	HDAC1
15228663	3'-Methoxyglabridin	HDAC8
15228663	3'-Methoxyglabridin	HPGDS
15228663	3'-Methoxyglabridin	HSD17B1
15228663	3'-Methoxyglabridin	HSD17B2
15228663	3'-Methoxyglabridin	HSD17B3
15228663	3'-Methoxyglabridin	HTR1A
15228663	3'-Methoxyglabridin	KDR
15228663	3'-Methoxyglabridin	KIT
15228663	3'-Methoxyglabridin	LCK
15228663	3'-Methoxyglabridin	LNPEP
15228663	3'-Methoxyglabridin	LTA4H
15228663	3'-Methoxyglabridin	MAOB
15228663	3'-Methoxyglabridin	MAPK14
15228663	3'-Methoxyglabridin	MAPT
15228663	3'-Methoxyglabridin	MCL1
15228663	3'-Methoxyglabridin	MELK
15228663	3'-Methoxyglabridin	MET
15228663	3'-Methoxyglabridin	MKNK1
15228663	3'-Methoxyglabridin	MMP8
15228663	3'-Methoxyglabridin	MTOR
15228663	3'-Methoxyglabridin	NOX1
15228663	3'-Methoxyglabridin	NOX4
15228663	3'-Methoxyglabridin	ODC1

15228663	3'-Methoxyglabridin	PDE10A
15228663	3'-Methoxyglabridin	PDE5A
15228663	3'-Methoxyglabridin	PDE7A
15228663	3'-Methoxyglabridin	PDGFRA
15228663	3'-Methoxyglabridin	PDGFRB
15228663	3'-Methoxyglabridin	PDK1
15228663	3'-Methoxyglabridin	PIK3CD
15228663	3'-Methoxyglabridin	PIK3CG
15228663	3'-Methoxyglabridin	PIK3R1
15228663	3'-Methoxyglabridin	PIM1
15228663	3'-Methoxyglabridin	PIM2
15228663	3'-Methoxyglabridin	PIM3
15228663	3'-Methoxyglabridin	PLAA
15228663	3'-Methoxyglabridin	PLK1
15228663	3'-Methoxyglabridin	PRF1
15228663	3'-Methoxyglabridin	PRKACA
15228663	3'-Methoxyglabridin	PRKCA
15228663	3'-Methoxyglabridin	PTK2
15228663	3'-Methoxyglabridin	PTK6
15228663	3'-Methoxyglabridin	PTPN1
15228663	3'-Methoxyglabridin	RAF1
15228663	3'-Methoxyglabridin	RET
15228663	3'-Methoxyglabridin	ROCK1
15228663	3'-Methoxyglabridin	S1PR1
15228663	3'-Methoxyglabridin	S1PR3
15228663	3'-Methoxyglabridin	SCD
15228663	3'-Methoxyglabridin	SHBG
15228663	3'-Methoxyglabridin	SNCA
15228663	3'-Methoxyglabridin	SRC
15228663	3'-Methoxyglabridin	SYK
15228663	3'-Methoxyglabridin	TBXA2R
15228663	3'-Methoxyglabridin	TRPM8
15228663	3'-Methoxyglabridin	VCP
15228663	3'-Methoxyglabridin	WEE1
15228663	3'-Methoxyglabridin	XDH
9927807	2-[(3R)-8,8-dimethyl-3,4-dihydro-2H-pyrano[6,5-f]chromen-3-yl]-5-methoxyphenol	ABL1
9927807	2-[(3R)-8,8-dimethyl-3,4-dihydro-2H-pyrano[6,5-f]chromen-3-yl]-5-methoxyphenol	ADAMTS5

9927807	2-[(3R)-8,8-dimethyl-3,4-dihydro-2H-pyrano[6,5-f]chromen-3-yl]-5-methoxyphenol	ADORA1
9927807	2-[(3R)-8,8-dimethyl-3,4-dihydro-2H-pyrano[6,5-f]chromen-3-yl]-5-methoxyphenol	ADORA2A
9927807	2-[(3R)-8,8-dimethyl-3,4-dihydro-2H-pyrano[6,5-f]chromen-3-yl]-5-methoxyphenol	ADORA2B
9927807	2-[(3R)-8,8-dimethyl-3,4-dihydro-2H-pyrano[6,5-f]chromen-3-yl]-5-methoxyphenol	ADORA3
9927807	2-[(3R)-8,8-dimethyl-3,4-dihydro-2H-pyrano[6,5-f]chromen-3-yl]-5-methoxyphenol	ALPL
9927807	2-[(3R)-8,8-dimethyl-3,4-dihydro-2H-pyrano[6,5-f]chromen-3-yl]-5-methoxyphenol	AR
9927807	2-[(3R)-8,8-dimethyl-3,4-dihydro-2H-pyrano[6,5-f]chromen-3-yl]-5-methoxyphenol	ATM
9927807	2-[(3R)-8,8-dimethyl-3,4-dihydro-2H-pyrano[6,5-f]chromen-3-yl]-5-methoxyphenol	ATP4A
9927807	2-[(3R)-8,8-dimethyl-3,4-dihydro-2H-pyrano[6,5-f]chromen-3-yl]-5-methoxyphenol	ATP4B
9927807	2-[(3R)-8,8-dimethyl-3,4-dihydro-2H-pyrano[6,5-f]chromen-3-yl]-5-methoxyphenol	ATR
9927807	2-[(3R)-8,8-dimethyl-3,4-dihydro-2H-pyrano[6,5-f]chromen-3-yl]-5-methoxyphenol	AVPR1A

9927807	2-[(3R)-8,8-dimethyl-3,4-dihydro-2H-pyrano[6,5-f]chromen-3-yl]-5-methoxyphenol	BRAF
9927807	2-[(3R)-8,8-dimethyl-3,4-dihydro-2H-pyrano[6,5-f]chromen-3-yl]-5-methoxyphenol	CCNB1
9927807	2-[(3R)-8,8-dimethyl-3,4-dihydro-2H-pyrano[6,5-f]chromen-3-yl]-5-methoxyphenol	CCNB2
9927807	2-[(3R)-8,8-dimethyl-3,4-dihydro-2H-pyrano[6,5-f]chromen-3-yl]-5-methoxyphenol	CCNB3
9927807	2-[(3R)-8,8-dimethyl-3,4-dihydro-2H-pyrano[6,5-f]chromen-3-yl]-5-methoxyphenol	CCND1
9927807	2-[(3R)-8,8-dimethyl-3,4-dihydro-2H-pyrano[6,5-f]chromen-3-yl]-5-methoxyphenol	CCNE1
9927807	2-[(3R)-8,8-dimethyl-3,4-dihydro-2H-pyrano[6,5-f]chromen-3-yl]-5-methoxyphenol	CCNE2
9927807	2-[(3R)-8,8-dimethyl-3,4-dihydro-2H-pyrano[6,5-f]chromen-3-yl]-5-methoxyphenol	CDK1
9927807	2-[(3R)-8,8-dimethyl-3,4-dihydro-2H-pyrano[6,5-f]chromen-3-yl]-5-methoxyphenol	CDK2
9927807	2-[(3R)-8,8-dimethyl-3,4-dihydro-2H-pyrano[6,5-f]chromen-3-yl]-5-methoxyphenol	CDK4
9927807	2-[(3R)-8,8-dimethyl-3,4-dihydro-2H-pyrano[6,5-f]chromen-3-yl]-5-methoxyphenol	CDK5

9927807	2-[(3R)-8,8-dimethyl-3,4-dihydro-2H-pyrano[6,5-f]chromen-3-yl]-5-methoxyphenol	CDK5R1
9927807	2-[(3R)-8,8-dimethyl-3,4-dihydro-2H-pyrano[6,5-f]chromen-3-yl]-5-methoxyphenol	CHEK1
9927807	2-[(3R)-8,8-dimethyl-3,4-dihydro-2H-pyrano[6,5-f]chromen-3-yl]-5-methoxyphenol	CHRNA7
9927807	2-[(3R)-8,8-dimethyl-3,4-dihydro-2H-pyrano[6,5-f]chromen-3-yl]-5-methoxyphenol	CRHR1
9927807	2-[(3R)-8,8-dimethyl-3,4-dihydro-2H-pyrano[6,5-f]chromen-3-yl]-5-methoxyphenol	CTSD
9927807	2-[(3R)-8,8-dimethyl-3,4-dihydro-2H-pyrano[6,5-f]chromen-3-yl]-5-methoxyphenol	DAGLA
9927807	2-[(3R)-8,8-dimethyl-3,4-dihydro-2H-pyrano[6,5-f]chromen-3-yl]-5-methoxyphenol	DRD1
9927807	2-[(3R)-8,8-dimethyl-3,4-dihydro-2H-pyrano[6,5-f]chromen-3-yl]-5-methoxyphenol	DRD2
9927807	2-[(3R)-8,8-dimethyl-3,4-dihydro-2H-pyrano[6,5-f]chromen-3-yl]-5-methoxyphenol	DRD3
9927807	2-[(3R)-8,8-dimethyl-3,4-dihydro-2H-pyrano[6,5-f]chromen-3-yl]-5-methoxyphenol	EGFR
9927807	2-[(3R)-8,8-dimethyl-3,4-dihydro-2H-pyrano[6,5-f]chromen-3-yl]-5-methoxyphenol	ELANE

9927807	2-[(3R)-8,8-dimethyl-3,4-dihydro-2H-pyrano[6,5-f]chromen-3-yl]-5-methoxyphenol	EP300
9927807	2-[(3R)-8,8-dimethyl-3,4-dihydro-2H-pyrano[6,5-f]chromen-3-yl]-5-methoxyphenol	EPHB2
9927807	2-[(3R)-8,8-dimethyl-3,4-dihydro-2H-pyrano[6,5-f]chromen-3-yl]-5-methoxyphenol	ERBB2
9927807	2-[(3R)-8,8-dimethyl-3,4-dihydro-2H-pyrano[6,5-f]chromen-3-yl]-5-methoxyphenol	ERN1
9927807	2-[(3R)-8,8-dimethyl-3,4-dihydro-2H-pyrano[6,5-f]chromen-3-yl]-5-methoxyphenol	ESR1
9927807	2-[(3R)-8,8-dimethyl-3,4-dihydro-2H-pyrano[6,5-f]chromen-3-yl]-5-methoxyphenol	ESR2
9927807	2-[(3R)-8,8-dimethyl-3,4-dihydro-2H-pyrano[6,5-f]chromen-3-yl]-5-methoxyphenol	ESRRG
9927807	2-[(3R)-8,8-dimethyl-3,4-dihydro-2H-pyrano[6,5-f]chromen-3-yl]-5-methoxyphenol	F3
9927807	2-[(3R)-8,8-dimethyl-3,4-dihydro-2H-pyrano[6,5-f]chromen-3-yl]-5-methoxyphenol	FASN
9927807	2-[(3R)-8,8-dimethyl-3,4-dihydro-2H-pyrano[6,5-f]chromen-3-yl]-5-methoxyphenol	FGFR1
9927807	2-[(3R)-8,8-dimethyl-3,4-dihydro-2H-pyrano[6,5-f]chromen-3-yl]-5-methoxyphenol	FLT1

9927807	2-[(3R)-8,8-dimethyl-3,4-dihydro-2H-pyrano[6,5-f]chromen-3-yl]-5-methoxyphenol	FLT3
9927807	2-[(3R)-8,8-dimethyl-3,4-dihydro-2H-pyrano[6,5-f]chromen-3-yl]-5-methoxyphenol	FNTA
9927807	2-[(3R)-8,8-dimethyl-3,4-dihydro-2H-pyrano[6,5-f]chromen-3-yl]-5-methoxyphenol	FNTB
9927807	2-[(3R)-8,8-dimethyl-3,4-dihydro-2H-pyrano[6,5-f]chromen-3-yl]-5-methoxyphenol	GCGR
9927807	2-[(3R)-8,8-dimethyl-3,4-dihydro-2H-pyrano[6,5-f]chromen-3-yl]-5-methoxyphenol	GCK
9927807	2-[(3R)-8,8-dimethyl-3,4-dihydro-2H-pyrano[6,5-f]chromen-3-yl]-5-methoxyphenol	GRIA1
9927807	2-[(3R)-8,8-dimethyl-3,4-dihydro-2H-pyrano[6,5-f]chromen-3-yl]-5-methoxyphenol	GRM4
9927807	2-[(3R)-8,8-dimethyl-3,4-dihydro-2H-pyrano[6,5-f]chromen-3-yl]-5-methoxyphenol	GSK3A
9927807	2-[(3R)-8,8-dimethyl-3,4-dihydro-2H-pyrano[6,5-f]chromen-3-yl]-5-methoxyphenol	HPGDS
9927807	2-[(3R)-8,8-dimethyl-3,4-dihydro-2H-pyrano[6,5-f]chromen-3-yl]-5-methoxyphenol	HSD17B1
9927807	2-[(3R)-8,8-dimethyl-3,4-dihydro-2H-pyrano[6,5-f]chromen-3-yl]-5-methoxyphenol	HSD17B2

9927807	2-[(3R)-8,8-dimethyl-3,4-dihydro-2H-pyrano[6,5-f]chromen-3-yl]-5-methoxyphenol	HSD17B3
9927807	2-[(3R)-8,8-dimethyl-3,4-dihydro-2H-pyrano[6,5-f]chromen-3-yl]-5-methoxyphenol	HSP90AB1
9927807	2-[(3R)-8,8-dimethyl-3,4-dihydro-2H-pyrano[6,5-f]chromen-3-yl]-5-methoxyphenol	HTR1A
9927807	2-[(3R)-8,8-dimethyl-3,4-dihydro-2H-pyrano[6,5-f]chromen-3-yl]-5-methoxyphenol	KDM1A
9927807	2-[(3R)-8,8-dimethyl-3,4-dihydro-2H-pyrano[6,5-f]chromen-3-yl]-5-methoxyphenol	KDR
9927807	2-[(3R)-8,8-dimethyl-3,4-dihydro-2H-pyrano[6,5-f]chromen-3-yl]-5-methoxyphenol	KIF11
9927807	2-[(3R)-8,8-dimethyl-3,4-dihydro-2H-pyrano[6,5-f]chromen-3-yl]-5-methoxyphenol	LCK
9927807	2-[(3R)-8,8-dimethyl-3,4-dihydro-2H-pyrano[6,5-f]chromen-3-yl]-5-methoxyphenol	LNPEP
9927807	2-[(3R)-8,8-dimethyl-3,4-dihydro-2H-pyrano[6,5-f]chromen-3-yl]-5-methoxyphenol	LTA4H
9927807	2-[(3R)-8,8-dimethyl-3,4-dihydro-2H-pyrano[6,5-f]chromen-3-yl]-5-methoxyphenol	MAOB
9927807	2-[(3R)-8,8-dimethyl-3,4-dihydro-2H-pyrano[6,5-f]chromen-3-yl]-5-methoxyphenol	MAPK14

9927807	2-[(3R)-8,8-dimethyl-3,4-dihydro-2H-pyrano[6,5-f]chromen-3-yl]-5-methoxyphenol	MAPT
9927807	2-[(3R)-8,8-dimethyl-3,4-dihydro-2H-pyrano[6,5-f]chromen-3-yl]-5-methoxyphenol	MARK1
9927807	2-[(3R)-8,8-dimethyl-3,4-dihydro-2H-pyrano[6,5-f]chromen-3-yl]-5-methoxyphenol	MET
9927807	2-[(3R)-8,8-dimethyl-3,4-dihydro-2H-pyrano[6,5-f]chromen-3-yl]-5-methoxyphenol	MMP1
9927807	2-[(3R)-8,8-dimethyl-3,4-dihydro-2H-pyrano[6,5-f]chromen-3-yl]-5-methoxyphenol	MTOR
9927807	2-[(3R)-8,8-dimethyl-3,4-dihydro-2H-pyrano[6,5-f]chromen-3-yl]-5-methoxyphenol	MYLK
9927807	2-[(3R)-8,8-dimethyl-3,4-dihydro-2H-pyrano[6,5-f]chromen-3-yl]-5-methoxyphenol	NEK1
9927807	2-[(3R)-8,8-dimethyl-3,4-dihydro-2H-pyrano[6,5-f]chromen-3-yl]-5-methoxyphenol	NOX1
9927807	2-[(3R)-8,8-dimethyl-3,4-dihydro-2H-pyrano[6,5-f]chromen-3-yl]-5-methoxyphenol	NOX4
9927807	2-[(3R)-8,8-dimethyl-3,4-dihydro-2H-pyrano[6,5-f]chromen-3-yl]-5-methoxyphenol	NR4A1
9927807	2-[(3R)-8,8-dimethyl-3,4-dihydro-2H-pyrano[6,5-f]chromen-3-yl]-5-methoxyphenol	ODC1

9927807	2-[(3R)-8,8-dimethyl-3,4-dihydro-2H-pyrano[6,5-f]chromen-3-yl]-5-methoxyphenol	PARP1
9927807	2-[(3R)-8,8-dimethyl-3,4-dihydro-2H-pyrano[6,5-f]chromen-3-yl]-5-methoxyphenol	PDE3A
9927807	2-[(3R)-8,8-dimethyl-3,4-dihydro-2H-pyrano[6,5-f]chromen-3-yl]-5-methoxyphenol	PDE4A
9927807	2-[(3R)-8,8-dimethyl-3,4-dihydro-2H-pyrano[6,5-f]chromen-3-yl]-5-methoxyphenol	PDE4B
9927807	2-[(3R)-8,8-dimethyl-3,4-dihydro-2H-pyrano[6,5-f]chromen-3-yl]-5-methoxyphenol	PDE5A
9927807	2-[(3R)-8,8-dimethyl-3,4-dihydro-2H-pyrano[6,5-f]chromen-3-yl]-5-methoxyphenol	PDE7A
9927807	2-[(3R)-8,8-dimethyl-3,4-dihydro-2H-pyrano[6,5-f]chromen-3-yl]-5-methoxyphenol	PDK1
9927807	2-[(3R)-8,8-dimethyl-3,4-dihydro-2H-pyrano[6,5-f]chromen-3-yl]-5-methoxyphenol	PIK3CG
9927807	2-[(3R)-8,8-dimethyl-3,4-dihydro-2H-pyrano[6,5-f]chromen-3-yl]-5-methoxyphenol	PIK3R1
9927807	2-[(3R)-8,8-dimethyl-3,4-dihydro-2H-pyrano[6,5-f]chromen-3-yl]-5-methoxyphenol	PIM1
9927807	2-[(3R)-8,8-dimethyl-3,4-dihydro-2H-pyrano[6,5-f]chromen-3-yl]-5-methoxyphenol	PIM2

9927807	2-[(3R)-8,8-dimethyl-3,4-dihydro-2H-pyrano[6,5-f]chromen-3-yl]-5-methoxyphenol	PLK1
9927807	2-[(3R)-8,8-dimethyl-3,4-dihydro-2H-pyrano[6,5-f]chromen-3-yl]-5-methoxyphenol	PRKDC
9927807	2-[(3R)-8,8-dimethyl-3,4-dihydro-2H-pyrano[6,5-f]chromen-3-yl]-5-methoxyphenol	PTPN1
9927807	2-[(3R)-8,8-dimethyl-3,4-dihydro-2H-pyrano[6,5-f]chromen-3-yl]-5-methoxyphenol	RET
9927807	2-[(3R)-8,8-dimethyl-3,4-dihydro-2H-pyrano[6,5-f]chromen-3-yl]-5-methoxyphenol	RORC
9927807	2-[(3R)-8,8-dimethyl-3,4-dihydro-2H-pyrano[6,5-f]chromen-3-yl]-5-methoxyphenol	S1PR1
9927807	2-[(3R)-8,8-dimethyl-3,4-dihydro-2H-pyrano[6,5-f]chromen-3-yl]-5-methoxyphenol	S1PR3
9927807	2-[(3R)-8,8-dimethyl-3,4-dihydro-2H-pyrano[6,5-f]chromen-3-yl]-5-methoxyphenol	SIGMAR1
9927807	2-[(3R)-8,8-dimethyl-3,4-dihydro-2H-pyrano[6,5-f]chromen-3-yl]-5-methoxyphenol	SMARCA2
9927807	2-[(3R)-8,8-dimethyl-3,4-dihydro-2H-pyrano[6,5-f]chromen-3-yl]-5-methoxyphenol	SNCA
9927807	2-[(3R)-8,8-dimethyl-3,4-dihydro-2H-pyrano[6,5-f]chromen-3-yl]-5-methoxyphenol	SRC

9927807	2-[(3R)-8,8-dimethyl-3,4-dihydro-2H-pyrano[6,5-f]chromen-3-yl]-5-methoxyphenol	SREBF2
9927807	2-[(3R)-8,8-dimethyl-3,4-dihydro-2H-pyrano[6,5-f]chromen-3-yl]-5-methoxyphenol	STAT6
9927807	2-[(3R)-8,8-dimethyl-3,4-dihydro-2H-pyrano[6,5-f]chromen-3-yl]-5-methoxyphenol	TBXA2R
9927807	2-[(3R)-8,8-dimethyl-3,4-dihydro-2H-pyrano[6,5-f]chromen-3-yl]-5-methoxyphenol	TGFBR1
9927807	2-[(3R)-8,8-dimethyl-3,4-dihydro-2H-pyrano[6,5-f]chromen-3-yl]-5-methoxyphenol	THRA
9927807	2-[(3R)-8,8-dimethyl-3,4-dihydro-2H-pyrano[6,5-f]chromen-3-yl]-5-methoxyphenol	TRPM8
9927807	2-[(3R)-8,8-dimethyl-3,4-dihydro-2H-pyrano[6,5-f]chromen-3-yl]-5-methoxyphenol	TRPV1
9927807	2-[(3R)-8,8-dimethyl-3,4-dihydro-2H-pyrano[6,5-f]chromen-3-yl]-5-methoxyphenol	WEE1
5318437	Inflacoumarin A	ABL1
5318437	Inflacoumarin A	ACVR1
5318437	Inflacoumarin A	ADAM17
5318437	Inflacoumarin A	ADRA1A
5318437	Inflacoumarin A	ADRA2A
5318437	Inflacoumarin A	ADRA2B
5318437	Inflacoumarin A	ADRA2C
5318437	Inflacoumarin A	AGTR1
5318437	Inflacoumarin A	AKR1B1
5318437	Inflacoumarin A	AURKC
5318437	Inflacoumarin A	BACE2
5318437	Inflacoumarin A	CA1

5318437	Inflacoumarin A	CA12
5318437	Inflacoumarin A	CA7
5318437	Inflacoumarin A	CA9
5318437	Inflacoumarin A	CAPN1
5318437	Inflacoumarin A	CCNE1
5318437	Inflacoumarin A	CCNE1
5318437	Inflacoumarin A	CCNE2
5318437	Inflacoumarin A	CDK2
5318437	Inflacoumarin A	CDK2
5318437	Inflacoumarin A	CDK4
5318437	Inflacoumarin A	CDK5
5318437	Inflacoumarin A	CHEK1
5318437	Inflacoumarin A	CHRNA4
5318437	Inflacoumarin A	CHRNB2
5318437	Inflacoumarin A	CLK1
5318437	Inflacoumarin A	COMT
5318437	Inflacoumarin A	CTSD
5318437	Inflacoumarin A	CYP19A1
5318437	Inflacoumarin A	DRD1
5318437	Inflacoumarin A	DRD2
5318437	Inflacoumarin A	DRD3
5318437	Inflacoumarin A	DYRK1A
5318437	Inflacoumarin A	DYRK1B
5318437	Inflacoumarin A	ELANE
5318437	Inflacoumarin A	ESR1
5318437	Inflacoumarin A	ESR2
5318437	Inflacoumarin A	GSK3A
5318437	Inflacoumarin A	HSD17B3
5318437	Inflacoumarin A	HSP90AA1
5318437	Inflacoumarin A	HSP90AB1
5318437	Inflacoumarin A	JAK2
5318437	Inflacoumarin A	KDM1A
5318437	Inflacoumarin A	KIF11
5318437	Inflacoumarin A	LDLR
5318437	Inflacoumarin A	LTB4R
5318437	Inflacoumarin A	MAP2K2
5318437	Inflacoumarin A	MAPK8
5318437	Inflacoumarin A	MDM2
5318437	Inflacoumarin A	MDM4
5318437	Inflacoumarin A	MMP1
5318437	Inflacoumarin A	MMP7
5318437	Inflacoumarin A	MMP8
5318437	Inflacoumarin A	NEK1
5318437	Inflacoumarin A	PAK4

5318437	Inflacoumarin A	PDGFRA
5318437	Inflacoumarin A	PDK1
5318437	Inflacoumarin A	PIK3CD
5318437	Inflacoumarin A	PITRM1
5318437	Inflacoumarin A	PLA2G2A
5318437	Inflacoumarin A	PLA2G7
5318437	Inflacoumarin A	PRKCZ
5318437	Inflacoumarin A	PTK6
5318437	Inflacoumarin A	RAF1
5318437	Inflacoumarin A	RET
5318437	Inflacoumarin A	ROS1
5318437	Inflacoumarin A	SHBG
5318437	Inflacoumarin A	SIGMAR1
5318437	Inflacoumarin A	TYMS
101666840	Kanzonol F	ABL1
101666840	Kanzonol F	ADAMTS4
101666840	Kanzonol F	ADAMTS5
101666840	Kanzonol F	ADORA1
101666840	Kanzonol F	ADORA2A
101666840	Kanzonol F	ADORA2B
101666840	Kanzonol F	ADORA3
101666840	Kanzonol F	AURKA
101666840	Kanzonol F	BRAF
101666840	Kanzonol F	CCKAR
101666840	Kanzonol F	CCKBR
101666840	Kanzonol F	CCNA1
101666840	Kanzonol F	CCNA2
101666840	Kanzonol F	CCNA2
101666840	Kanzonol F	CDK2
101666840	Kanzonol F	CDK2
101666840	Kanzonol F	CDK5
101666840	Kanzonol F	CDK5R1
101666840	Kanzonol F	CHEK1
101666840	Kanzonol F	CPT1A
101666840	Kanzonol F	CSF1R
101666840	Kanzonol F	CTSB
101666840	Kanzonol F	CXCR2
101666840	Kanzonol F	DDX3X
101666840	Kanzonol F	EGFR
101666840	Kanzonol F	ELANE
101666840	Kanzonol F	ESR1
101666840	Kanzonol F	ESR2
101666840	Kanzonol F	F10
101666840	Kanzonol F	FLT3

101666840	Kanzonol F	FNTA
101666840	Kanzonol F	FNTB
101666840	Kanzonol F	GCK
101666840	Kanzonol F	GPR119
101666840	Kanzonol F	GPR55
101666840	Kanzonol F	GRK2
101666840	Kanzonol F	GRK3
101666840	Kanzonol F	GRK5
101666840	Kanzonol F	GRM2
101666840	Kanzonol F	GSK3B
101666840	Kanzonol F	HSP90AB1
101666840	Kanzonol F	ITK
101666840	Kanzonol F	KIF11
101666840	Kanzonol F	LDLR
101666840	Kanzonol F	MGAM
101666840	Kanzonol F	NOS1
101666840	Kanzonol F	NOS3
101666840	Kanzonol F	NR1H4
101666840	Kanzonol F	NR4A1
101666840	Kanzonol F	ODC1
101666840	Kanzonol F	PDE10A
101666840	Kanzonol F	PDE5A
101666840	Kanzonol F	PDGFRB
101666840	Kanzonol F	PDK1
101666840	Kanzonol F	PTPN1
101666840	Kanzonol F	QPCTL
101666840	Kanzonol F	RAF1
101666840	Kanzonol F	SIGMAR1
101666840	Kanzonol F	SLC2A1
101666840	Kanzonol F	SLC2A2
101666840	Kanzonol F	SLC2A3
101666840	Kanzonol F	SLC8A1
101666840	Kanzonol F	SYK
25015742	7,2',4'-trihydroxy − 5-methoxy-3 − arylcoumarin	ABCB1
25015742	7,2',4'-trihydroxy − 5-methoxy-3 − arylcoumarin	ABCG2
25015742	7,2',4'-trihydroxy − 5-methoxy-3 − arylcoumarin	ACHE
25015742	7,2',4'-trihydroxy − 5-methoxy-3 − arylcoumarin	ADAM17
25015742	7,2',4'-trihydroxy − 5-methoxy-3 − arylcoumarin	ADORA1

25015742	7,2',4'-trihydroxy − 5-methoxy-3 − arylcoumarin	ADORA2A
25015742	7,2',4'-trihydroxy − 5-methoxy-3 − arylcoumarin	ALDH2
25015742	7,2',4'-trihydroxy − 5-methoxy-3 − arylcoumarin	ALOX12
25015742	7,2',4'-trihydroxy − 5-methoxy-3 − arylcoumarin	ALOX5
25015742	7,2',4'-trihydroxy − 5-methoxy-3 − arylcoumarin	AURKA
25015742	7,2',4'-trihydroxy − 5-methoxy-3 − arylcoumarin	BACE1
25015742	7,2',4'-trihydroxy − 5-methoxy-3 − arylcoumarin	BCL2L1
25015742	7,2',4'-trihydroxy − 5-methoxy-3 − arylcoumarin	CA1
25015742	7,2',4'-trihydroxy − 5-methoxy-3 − arylcoumarin	CA12
25015742	7,2',4'-trihydroxy − 5-methoxy-3 − arylcoumarin	CA2
25015742	7,2',4'-trihydroxy − 5-methoxy-3 − arylcoumarin	CA4
25015742	7,2',4'-trihydroxy − 5-methoxy-3 − arylcoumarin	CA7
25015742	7,2',4'-trihydroxy − 5-methoxy-3 − arylcoumarin	CAPN1
25015742	7,2',4'-trihydroxy − 5-methoxy-3 − arylcoumarin	CBR1
25015742	7,2',4'-trihydroxy − 5-methoxy-3 − arylcoumarin	CCNB1
25015742	7,2',4'-trihydroxy − 5-methoxy-3 − arylcoumarin	CCNE1
25015742	7,2',4'-trihydroxy − 5-methoxy-3 − arylcoumarin	CCNE1
25015742	7,2',4'-trihydroxy − 5-methoxy-3 − arylcoumarin	CDC7
25015742	7,2',4'-trihydroxy − 5-methoxy-3 − arylcoumarin	CDK1
25015742	7,2',4'-trihydroxy − 5-methoxy-3 − arylcoumarin	CDK2
25015742	7,2',4'-trihydroxy − 5-methoxy-3 − arylcoumarin	CDK2
25015742	7,2',4'-trihydroxy − 5-methoxy-3 − arylcoumarin	CDK3

25015742	7,2',4'-trihydroxy − 5-methoxy-3 − arylcoumarin	CHEK1
25015742	7,2',4'-trihydroxy − 5-methoxy-3 − arylcoumarin	CHRNA7
25015742	7,2',4'-trihydroxy − 5-methoxy-3 − arylcoumarin	CLK1
25015742	7,2',4'-trihydroxy − 5-methoxy-3 − arylcoumarin	CYP19A1
25015742	7,2',4'-trihydroxy − 5-methoxy-3 − arylcoumarin	DRD1
25015742	7,2',4'-trihydroxy − 5-methoxy-3 − arylcoumarin	DYRK1B
25015742	7,2',4'-trihydroxy − 5-methoxy-3 − arylcoumarin	EGFR
25015742	7,2',4'-trihydroxy − 5-methoxy-3 − arylcoumarin	ERBB2
25015742	7,2',4'-trihydroxy − 5-methoxy-3 − arylcoumarin	ESR1
25015742	7,2',4'-trihydroxy − 5-methoxy-3 − arylcoumarin	ESR2
25015742	7,2',4'-trihydroxy − 5-methoxy-3 − arylcoumarin	ESRRA
25015742	7,2',4'-trihydroxy − 5-methoxy-3 − arylcoumarin	ESRRB
25015742	7,2',4'-trihydroxy − 5-methoxy-3 − arylcoumarin	FGFR1
25015742	7,2',4'-trihydroxy − 5-methoxy-3 − arylcoumarin	GRK2
25015742	7,2',4'-trihydroxy − 5-methoxy-3 − arylcoumarin	HPGDS
25015742	7,2',4'-trihydroxy − 5-methoxy-3 − arylcoumarin	HSD17B1
25015742	7,2',4'-trihydroxy − 5-methoxy-3 − arylcoumarin	HSD17B14
25015742	7,2',4'-trihydroxy − 5-methoxy-3 − arylcoumarin	HSD17B2
25015742	7,2',4'-trihydroxy − 5-methoxy-3 − arylcoumarin	HTR2A
25015742	7,2',4'-trihydroxy − 5-methoxy-3 − arylcoumarin	HTR2B
25015742	7,2',4'-trihydroxy − 5-methoxy-3 − arylcoumarin	IGF1R
25015742	7,2',4'-trihydroxy − 5-methoxy-3 − arylcoumarin	IGFBP3

25015742	7,2',4'-trihydroxy − 5-methoxy-3 − arylcoumarin	IL2
25015742	7,2',4'-trihydroxy − 5-methoxy-3 − arylcoumarin	INSR
25015742	7,2',4'-trihydroxy − 5-methoxy-3 − arylcoumarin	KDR
25015742	7,2',4'-trihydroxy − 5-methoxy-3 − arylcoumarin	MAOA
25015742	7,2',4'-trihydroxy − 5-methoxy-3 − arylcoumarin	MAOB
25015742	7,2',4'-trihydroxy − 5-methoxy-3 − arylcoumarin	MAPT
25015742	7,2',4'-trihydroxy − 5-methoxy-3 − arylcoumarin	MET
25015742	7,2',4'-trihydroxy − 5-methoxy-3 − arylcoumarin	MGAM
25015742	7,2',4'-trihydroxy − 5-methoxy-3 − arylcoumarin	MIF
25015742	7,2',4'-trihydroxy − 5-methoxy-3 − arylcoumarin	MMP1
25015742	7,2',4'-trihydroxy − 5-methoxy-3 − arylcoumarin	MMP2
25015742	7,2',4'-trihydroxy − 5-methoxy-3 − arylcoumarin	MMP3
25015742	7,2',4'-trihydroxy − 5-methoxy-3 − arylcoumarin	MMP9
25015742	7,2',4'-trihydroxy − 5-methoxy-3 − arylcoumarin	NOX4
25015742	7,2',4'-trihydroxy − 5-methoxy-3 − arylcoumarin	NQO2
25015742	7,2',4'-trihydroxy − 5-methoxy-3 − arylcoumarin	PON1
25015742	7,2',4'-trihydroxy − 5-methoxy-3 − arylcoumarin	PPARA
25015742	7,2',4'-trihydroxy − 5-methoxy-3 − arylcoumarin	PTGS1
25015742	7,2',4'-trihydroxy − 5-methoxy-3 − arylcoumarin	PTPRS
25015742	7,2',4'-trihydroxy − 5-methoxy-3 − arylcoumarin	SERPINE1
25015742	7,2',4'-trihydroxy − 5-methoxy-3 − arylcoumarin	SIGMAR1
25015742	7,2',4'-trihydroxy − 5-methoxy-3 − arylcoumarin	SLC6A2

25015742	7,2',4'-trihydroxy − 5-methoxy-3 − arylcoumarin	SNCA
25015742	7,2',4'-trihydroxy − 5-methoxy-3 − arylcoumarin	SRC
25015742	7,2',4'-trihydroxy − 5-methoxy-3 − arylcoumarin	TLR9
25015742	7,2',4'-trihydroxy − 5-methoxy-3 − arylcoumarin	TRPM8
25015742	7,2',4'-trihydroxy − 5-methoxy-3 − arylcoumarin	TYR
25015742	7,2',4'-trihydroxy − 5-methoxy-3 − arylcoumarin	WEE1
25015742	7,2',4'-trihydroxy − 5-methoxy-3 − arylcoumarin	XDH
25015742	7,2',4'-trihydroxy − 5-methoxy-3 − arylcoumarin	YWHAG
5317480	Lupiwighteone	ABCB1
5317480	Lupiwighteone	ABCG2
5317480	Lupiwighteone	ACHE
5317480	Lupiwighteone	AKT1
5317480	Lupiwighteone	ALDH2
5317480	Lupiwighteone	ALOX12
5317480	Lupiwighteone	ALOX15
5317480	Lupiwighteone	BACE1
5317480	Lupiwighteone	BCL2
5317480	Lupiwighteone	CA12
5317480	Lupiwighteone	CA4
5317480	Lupiwighteone	CCR4
5317480	Lupiwighteone	CYP19A1
5317480	Lupiwighteone	EGFR
5317480	Lupiwighteone	ESR1
5317480	Lupiwighteone	ESR2
5317480	Lupiwighteone	ESRRA
5317480	Lupiwighteone	ESRRB
5317480	Lupiwighteone	FNTA
5317480	Lupiwighteone	FNTB
5317480	Lupiwighteone	GCGR
5317480	Lupiwighteone	GPR84
5317480	Lupiwighteone	HSD17B1
5317480	Lupiwighteone	HSD17B2
5317480	Lupiwighteone	HSP90AA1
5317480	Lupiwighteone	HSP90AB1
5317480	Lupiwighteone	HSP90B1
5317480	Lupiwighteone	HTR2A

5317480	Lupiwighteone	HTR2C
5317480	Lupiwighteone	IL2
5317480	Lupiwighteone	MAOA
5317480	Lupiwighteone	MGAM
5317480	Lupiwighteone	MIF
5317480	Lupiwighteone	PDE10A
5317480	Lupiwighteone	PDE5A
5317480	Lupiwighteone	PFKFB3
5317480	Lupiwighteone	PTPN1
5317480	Lupiwighteone	RELA
5317480	Lupiwighteone	SIRT1
5317480	Lupiwighteone	TBXAS1
5317480	Lupiwighteone	TYR
5317480	Lupiwighteone	XDH
268208	7-Acetoxy-2-methylisoflavone	ABCB1
268208	7-Acetoxy-2-methylisoflavone	ABCG2
268208	7-Acetoxy-2-methylisoflavone	ADAMTS5
268208	7-Acetoxy-2-methylisoflavone	ADORA1
268208	7-Acetoxy-2-methylisoflavone	ADORA2A
268208	7-Acetoxy-2-methylisoflavone	ADORA2B
268208	7-Acetoxy-2-methylisoflavone	AGPAT2
268208	7-Acetoxy-2-methylisoflavone	ALDH2
268208	7-Acetoxy-2-methylisoflavone	APH1A
268208	7-Acetoxy-2-methylisoflavone	APH1B
268208	7-Acetoxy-2-methylisoflavone	AURKA
268208	7-Acetoxy-2-methylisoflavone	CA1
268208	7-Acetoxy-2-methylisoflavone	CA12
268208	7-Acetoxy-2-methylisoflavone	CA2
268208	7-Acetoxy-2-methylisoflavone	CA4
268208	7-Acetoxy-2-methylisoflavone	CA7
268208	7-Acetoxy-2-methylisoflavone	CBR1
268208	7-Acetoxy-2-methylisoflavone	CCNB1
268208	7-Acetoxy-2-methylisoflavone	CCND1
268208	7-Acetoxy-2-methylisoflavone	CCNE1
268208	7-Acetoxy-2-methylisoflavone	CCNE2
268208	7-Acetoxy-2-methylisoflavone	CCNT1
268208	7-Acetoxy-2-methylisoflavone	CDC7
268208	7-Acetoxy-2-methylisoflavone	CDC7
268208	7-Acetoxy-2-methylisoflavone	CDK1
268208	7-Acetoxy-2-methylisoflavone	CDK2
268208	7-Acetoxy-2-methylisoflavone	CDK4
268208	7-Acetoxy-2-methylisoflavone	CDK9
268208	7-Acetoxy-2-methylisoflavone	CDK9
268208	7-Acetoxy-2-methylisoflavone	CHEK1

268208	7-Acetoxy-2-methylisoflavone	CRHR1
268208	7-Acetoxy-2-methylisoflavone	CSF1R
268208	7-Acetoxy-2-methylisoflavone	CYP17A1
268208	7-Acetoxy-2-methylisoflavone	CYP19A1
268208	7-Acetoxy-2-methylisoflavone	DBF4
268208	7-Acetoxy-2-methylisoflavone	DHFR
268208	7-Acetoxy-2-methylisoflavone	DHODH
268208	7-Acetoxy-2-methylisoflavone	DYRK1A
268208	7-Acetoxy-2-methylisoflavone	EGFR
268208	7-Acetoxy-2-methylisoflavone	EPHX1
268208	7-Acetoxy-2-methylisoflavone	EPHX2
268208	7-Acetoxy-2-methylisoflavone	ESR2
268208	7-Acetoxy-2-methylisoflavone	ESRRB
268208	7-Acetoxy-2-methylisoflavone	FNTA
268208	7-Acetoxy-2-methylisoflavone	FNTB
268208	7-Acetoxy-2-methylisoflavone	GABRA1
268208	7-Acetoxy-2-methylisoflavone	GABRA2
268208	7-Acetoxy-2-methylisoflavone	GABRA3
268208	7-Acetoxy-2-methylisoflavone	GABRA5
268208	7-Acetoxy-2-methylisoflavone	GABRB3
268208	7-Acetoxy-2-methylisoflavone	GABRB3
268208	7-Acetoxy-2-methylisoflavone	GABRB3
268208	7-Acetoxy-2-methylisoflavone	GABRB3
268208	7-Acetoxy-2-methylisoflavone	GABRG2
268208	7-Acetoxy-2-methylisoflavone	GABRG2
268208	7-Acetoxy-2-methylisoflavone	GABRG2
268208	7-Acetoxy-2-methylisoflavone	GABRG2
268208	7-Acetoxy-2-methylisoflavone	GCK
268208	7-Acetoxy-2-methylisoflavone	GRIA2
268208	7-Acetoxy-2-methylisoflavone	GRIN1
268208	7-Acetoxy-2-methylisoflavone	GRIN2A
268208	7-Acetoxy-2-methylisoflavone	GRM1
268208	7-Acetoxy-2-methylisoflavone	GRM5
268208	7-Acetoxy-2-methylisoflavone	HSD17B1
268208	7-Acetoxy-2-methylisoflavone	HSD17B2
268208	7-Acetoxy-2-methylisoflavone	HTR2A
268208	7-Acetoxy-2-methylisoflavone	HTR2C
268208	7-Acetoxy-2-methylisoflavone	IL2
268208	7-Acetoxy-2-methylisoflavone	IRAK4
268208	7-Acetoxy-2-methylisoflavone	JAK2
268208	7-Acetoxy-2-methylisoflavone	JAK3
268208	7-Acetoxy-2-methylisoflavone	KAT2B
268208	7-Acetoxy-2-methylisoflavone	KCNH2
268208	7-Acetoxy-2-methylisoflavone	KCNK3

268208	7-Acetoxy-2-methylisoflavone	KCNK9
268208	7-Acetoxy-2-methylisoflavone	KDM4A
268208	7-Acetoxy-2-methylisoflavone	KDM4C
268208	7-Acetoxy-2-methylisoflavone	KDM4D
268208	7-Acetoxy-2-methylisoflavone	KDM5C
268208	7-Acetoxy-2-methylisoflavone	LRRK2
268208	7-Acetoxy-2-methylisoflavone	MAOA
268208	7-Acetoxy-2-methylisoflavone	MAOB
268208	7-Acetoxy-2-methylisoflavone	MAPK10
268208	7-Acetoxy-2-methylisoflavone	MAPK8
268208	7-Acetoxy-2-methylisoflavone	MAPKAPK2
268208	7-Acetoxy-2-methylisoflavone	MGAM
268208	7-Acetoxy-2-methylisoflavone	NAAA
268208	7-Acetoxy-2-methylisoflavone	NCSTN
268208	7-Acetoxy-2-methylisoflavone	NR1H4
268208	7-Acetoxy-2-methylisoflavone	NR3C1
268208	7-Acetoxy-2-methylisoflavone	NR3C2
268208	7-Acetoxy-2-methylisoflavone	NTRK1
268208	7-Acetoxy-2-methylisoflavone	PDE10A
268208	7-Acetoxy-2-methylisoflavone	PDGFRA
268208	7-Acetoxy-2-methylisoflavone	PDGFRB
268208	7-Acetoxy-2-methylisoflavone	PFKFB3
268208	7-Acetoxy-2-methylisoflavone	PGK1
268208	7-Acetoxy-2-methylisoflavone	PGR
268208	7-Acetoxy-2-methylisoflavone	PIK3CA
268208	7-Acetoxy-2-methylisoflavone	PIK3CB
268208	7-Acetoxy-2-methylisoflavone	PIK3CD
268208	7-Acetoxy-2-methylisoflavone	PIK3CD
268208	7-Acetoxy-2-methylisoflavone	PIK3R1
268208	7-Acetoxy-2-methylisoflavone	PRF1
268208	7-Acetoxy-2-methylisoflavone	PRKCQ
268208	7-Acetoxy-2-methylisoflavone	PROKR1
268208	7-Acetoxy-2-methylisoflavone	PSEN1
268208	7-Acetoxy-2-methylisoflavone	PSEN2
268208	7-Acetoxy-2-methylisoflavone	PSENEN
268208	7-Acetoxy-2-methylisoflavone	PTGES
268208	7-Acetoxy-2-methylisoflavone	PTGS1
268208	7-Acetoxy-2-methylisoflavone	QPCT
268208	7-Acetoxy-2-methylisoflavone	RET
268208	7-Acetoxy-2-methylisoflavone	SCD
268208	7-Acetoxy-2-methylisoflavone	SHH
268208	7-Acetoxy-2-methylisoflavone	SIRT2
268208	7-Acetoxy-2-methylisoflavone	SLC6A2
268208	7-Acetoxy-2-methylisoflavone	TBXAS1

268208	7-Acetoxy-2-methylisoflavone	TGFB1
268208	7-Acetoxy-2-methylisoflavone	TGFBR1
268208	7-Acetoxy-2-methylisoflavone	TRPA1
268208	7-Acetoxy-2-methylisoflavone	TRPM8
268208	7-Acetoxy-2-methylisoflavone	TYMS
177149	Vestitol	ABL1
177149	Vestitol	ADAM17
177149	Vestitol	ADCY5
177149	Vestitol	ADORA2A
177149	Vestitol	ADORA2B
177149	Vestitol	AKR1B10
177149	Vestitol	ALOX12
177149	Vestitol	ALOX15
177149	Vestitol	ALOX15B
177149	Vestitol	ALPL
177149	Vestitol	AURKA
177149	Vestitol	AURKB
177149	Vestitol	BCHE
177149	Vestitol	BRAF
177149	Vestitol	CA14
177149	Vestitol	CASP3
177149	Vestitol	CASP7
177149	Vestitol	CCNB1
177149	Vestitol	CCNB2
177149	Vestitol	CCNB3
177149	Vestitol	CCNE1
177149	Vestitol	CCNE1
177149	Vestitol	CDK1
177149	Vestitol	CDK1
177149	Vestitol	CDK2
177149	Vestitol	CDK2
177149	Vestitol	CDK3
177149	Vestitol	CDK4
177149	Vestitol	CDK5
177149	Vestitol	CDK5R1
177149	Vestitol	CHEK1
177149	Vestitol	CHRNA7
177149	Vestitol	CLK1
177149	Vestitol	COMT
177149	Vestitol	CTSS
177149	Vestitol	CYP19A1
177149	Vestitol	CYP24A1
177149	Vestitol	DRD2
177149	Vestitol	DUSP3

177149	Vestitol	DYRK1B
177149	Vestitol	EIF2AK2
177149	Vestitol	ESR1
177149	Vestitol	ESR2
177149	Vestitol	ESRRA
177149	Vestitol	ESRRB
177149	Vestitol	EZR
177149	Vestitol	F3
177149	Vestitol	GRIA1
177149	Vestitol	GRK6
177149	Vestitol	GSK3B
177149	Vestitol	GSTM2
177149	Vestitol	GSTP1
177149	Vestitol	HDAC10
177149	Vestitol	HDAC11
177149	Vestitol	HDAC2
177149	Vestitol	HDAC3
177149	Vestitol	HDAC9
177149	Vestitol	HSD17B14
177149	Vestitol	HSD17B2
177149	Vestitol	HSD17B3
177149	Vestitol	HSP90AA1
177149	Vestitol	HSP90AB1
177149	Vestitol	HTR2A
177149	Vestitol	HTT
177149	Vestitol	IGF1R
177149	Vestitol	INSR
177149	Vestitol	KIF11
177149	Vestitol	LCK
177149	Vestitol	LIMK1
177149	Vestitol	LNPEP
177149	Vestitol	MAOB
177149	Vestitol	MAP2K1
177149	Vestitol	MAPKAPK2
177149	Vestitol	MCL1
177149	Vestitol	MIF
177149	Vestitol	NOX1
177149	Vestitol	NOX4
177149	Vestitol	PDE7A
177149	Vestitol	PDK1
177149	Vestitol	PFKFB3
177149	Vestitol	PIK3CA
177149	Vestitol	PIK3CD
177149	Vestitol	PIK3CG

177149	Vestitol	PIM1
177149	Vestitol	PIM2
177149	Vestitol	PIM3
177149	Vestitol	PLK1
177149	Vestitol	PRF1
177149	Vestitol	PRKACA
177149	Vestitol	PRKCA
177149	Vestitol	PTGS1
177149	Vestitol	PTGS2
177149	Vestitol	PTPN1
177149	Vestitol	RAF1
177149	Vestitol	RET
177149	Vestitol	ROCK1
177149	Vestitol	RPS6KB1
177149	Vestitol	SIGMAR1
177149	Vestitol	SNCA
177149	Vestitol	TAAR1
177149	Vestitol	TNKS
177149	Vestitol	TNKS2
177149	Vestitol	TRPM8
177149	Vestitol	TYR
177149	Vestitol	VCP
177149	Vestitol	WEE1
480780	Gancaonin G	ABCG2
480780	Gancaonin G	ACHE
480780	Gancaonin G	ADAM17
480780	Gancaonin G	ADORA1
480780	Gancaonin G	ADORA2A
480780	Gancaonin G	ADORA2B
480780	Gancaonin G	ALDH2
480780	Gancaonin G	APP
480780	Gancaonin G	AURKA
480780	Gancaonin G	BLK
480780	Gancaonin G	BMP1
480780	Gancaonin G	BMX
480780	Gancaonin G	BTK
480780	Gancaonin G	CA12
480780	Gancaonin G	CA4
480780	Gancaonin G	CA7
480780	Gancaonin G	CBR1
480780	Gancaonin G	CCNB1
480780	Gancaonin G	CCNE1
480780	Gancaonin G	CCNE1
480780	Gancaonin G	CDK1

480780	Gancaonin G	CDK2
480780	Gancaonin G	CDK2
480780	Gancaonin G	CDK3
480780	Gancaonin G	CES1
480780	Gancaonin G	CES2
480780	Gancaonin G	CHEK1
480780	Gancaonin G	CMA1
480780	Gancaonin G	COMT
480780	Gancaonin G	COQ8B
480780	Gancaonin G	CPT1A
480780	Gancaonin G	CSK
480780	Gancaonin G	CTSK
480780	Gancaonin G	CYP19A1
480780	Gancaonin G	EGFR
480780	Gancaonin G	EPHA1
480780	Gancaonin G	EPHA2
480780	Gancaonin G	EPHA3
480780	Gancaonin G	EPHA4
480780	Gancaonin G	EPHA5
480780	Gancaonin G	EPHA6
480780	Gancaonin G	EPHB2
480780	Gancaonin G	EPHB3
480780	Gancaonin G	ESR1
480780	Gancaonin G	ESR2
480780	Gancaonin G	ESRRA
480780	Gancaonin G	ESRRB
480780	Gancaonin G	FCER2
480780	Gancaonin G	FGR
480780	Gancaonin G	FOLH1
480780	Gancaonin G	FYN
480780	Gancaonin G	HDAC1
480780	Gancaonin G	HDAC10
480780	Gancaonin G	HDAC2
480780	Gancaonin G	HDAC3
480780	Gancaonin G	HDAC4
480780	Gancaonin G	HDAC6
480780	Gancaonin G	HDAC8
480780	Gancaonin G	HPGDS
480780	Gancaonin G	HSD17B1
480780	Gancaonin G	HSD17B2
480780	Gancaonin G	HTR2A
480780	Gancaonin G	LCK
480780	Gancaonin G	LYN
480780	Gancaonin G	MAOA

480780	Gancaonin G	MAP2K1
480780	Gancaonin G	MAPK8
480780	Gancaonin G	ME1
480780	Gancaonin G	MERTK
480780	Gancaonin G	MGAM
480780	Gancaonin G	MIF
480780	Gancaonin G	MMP1
480780	Gancaonin G	MMP13
480780	Gancaonin G	MMP2
480780	Gancaonin G	MMP3
480780	Gancaonin G	MMP7
480780	Gancaonin G	MMP9
480780	Gancaonin G	MTOR
480780	Gancaonin G	NAMPT
480780	Gancaonin G	NFKB1
480780	Gancaonin G	ODC1
480780	Gancaonin G	PAK4
480780	Gancaonin G	PDE10A
480780	Gancaonin G	PDE4A
480780	Gancaonin G	PDE4B
480780	Gancaonin G	PDE4C
480780	Gancaonin G	PDE4D
480780	Gancaonin G	PDE7A
480780	Gancaonin G	PDGFRB
480780	Gancaonin G	PIK3CA
480780	Gancaonin G	PPARG
480780	Gancaonin G	PTGS1
480780	Gancaonin G	PTK6
480780	Gancaonin G	PTPN1
480780	Gancaonin G	ROCK1
480780	Gancaonin G	ROCK2
480780	Gancaonin G	RPS6KB1
480780	Gancaonin G	SCD
480780	Gancaonin G	SLC6A2
480780	Gancaonin G	TACR3
480780	Gancaonin G	TXK
480780	Gancaonin G	TYRO3
480780	Gancaonin G	YES1
5481949	Gancaonin H	ACHE
5481949	Gancaonin H	AGTR1
5481949	Gancaonin H	CHEK1
5481949	Gancaonin H	F10
5481949	Gancaonin H	F2
5481949	Gancaonin H	GCGR

5481949	Gancaonin H	HSP90AA1
5481949	Gancaonin H	KISS1R
5481949	Gancaonin H	MELK
5481949	Gancaonin H	MTOR
5481949	Gancaonin H	PDK1
5481949	Gancaonin H	PIK3CA
5481949	Gancaonin H	PRKCA
5481949	Gancaonin H	PRKCB
5481949	Gancaonin H	PRKCD
5481949	Gancaonin H	PRKCE
5481949	Gancaonin H	PTPN1
5481949	Gancaonin H	RARA
5481949	Gancaonin H	WEE1
15840593	Licoagrocarpin	ABCG2
15840593	Licoagrocarpin	ABL1
15840593	Licoagrocarpin	ADAM17
15840593	Licoagrocarpin	ADAM33
15840593	Licoagrocarpin	ADAMTS5
15840593	Licoagrocarpin	AKR1B10
15840593	Licoagrocarpin	ALK
15840593	Licoagrocarpin	APH1A
15840593	Licoagrocarpin	APH1B
15840593	Licoagrocarpin	AURKA
15840593	Licoagrocarpin	AURKB
15840593	Licoagrocarpin	BMP1
15840593	Licoagrocarpin	BRAF
15840593	Licoagrocarpin	BRD4
15840593	Licoagrocarpin	CAPN1
15840593	Licoagrocarpin	CCNB1
15840593	Licoagrocarpin	CCND1
15840593	Licoagrocarpin	CCNE1
15840593	Licoagrocarpin	CCNE1
15840593	Licoagrocarpin	CCNE1
15840593	Licoagrocarpin	CCNE2
15840593	Licoagrocarpin	CDK1
15840593	Licoagrocarpin	CDK1
15840593	Licoagrocarpin	CDK2
15840593	Licoagrocarpin	CDK2
15840593	Licoagrocarpin	CDK2
15840593	Licoagrocarpin	CDK3
15840593	Licoagrocarpin	CDK4
15840593	Licoagrocarpin	CDK4
15840593	Licoagrocarpin	CFD
15840593	Licoagrocarpin	CHEK1

15840593	Licoagrocarpin	CLK1
15840593	Licoagrocarpin	CRHR1
15840593	Licoagrocarpin	CTSD
15840593	Licoagrocarpin	DHFR
15840593	Licoagrocarpin	DPP4
15840593	Licoagrocarpin	DRD1
15840593	Licoagrocarpin	DYRK1B
15840593	Licoagrocarpin	EPHA1
15840593	Licoagrocarpin	EPHA2
15840593	Licoagrocarpin	EPHA3
15840593	Licoagrocarpin	EPHA4
15840593	Licoagrocarpin	EPHA5
15840593	Licoagrocarpin	EPHA7
15840593	Licoagrocarpin	EPHA8
15840593	Licoagrocarpin	EPHB1
15840593	Licoagrocarpin	EPHB2
15840593	Licoagrocarpin	EPHB3
15840593	Licoagrocarpin	EPHB4
15840593	Licoagrocarpin	ERN1
15840593	Licoagrocarpin	EZR
15840593	Licoagrocarpin	FLT3
15840593	Licoagrocarpin	FLT4
15840593	Licoagrocarpin	GCGR
15840593	Licoagrocarpin	GSK3B
15840593	Licoagrocarpin	HCRTR1
15840593	Licoagrocarpin	HCRTR2
15840593	Licoagrocarpin	HDAC1
15840593	Licoagrocarpin	HSD11B1
15840593	Licoagrocarpin	HSD17B2
15840593	Licoagrocarpin	HSP90AB1
15840593	Licoagrocarpin	LCK
15840593	Licoagrocarpin	LDHA
15840593	Licoagrocarpin	MAP2K2
15840593	Licoagrocarpin	MAPK8
15840593	Licoagrocarpin	MARS
15840593	Licoagrocarpin	MMP1
15840593	Licoagrocarpin	MMP3
15840593	Licoagrocarpin	MMP7
15840593	Licoagrocarpin	MYLK
15840593	Licoagrocarpin	NCSTN
15840593	Licoagrocarpin	NR3C2
15840593	Licoagrocarpin	OPRK1
15840593	Licoagrocarpin	OXTR
15840593	Licoagrocarpin	PARP1

15840593	Licoagrocarpin	PDE10A
15840593	Licoagrocarpin	PDE5A
15840593	Licoagrocarpin	PDE6C
15840593	Licoagrocarpin	PDF
15840593	Licoagrocarpin	PDGFRA
15840593	Licoagrocarpin	PDGFRB
15840593	Licoagrocarpin	PDK1
15840593	Licoagrocarpin	PRKCZ
15840593	Licoagrocarpin	PSEN1
15840593	Licoagrocarpin	PSEN2
15840593	Licoagrocarpin	PSENEN
15840593	Licoagrocarpin	PTK2B
15840593	Licoagrocarpin	PTPN1
15840593	Licoagrocarpin	RAF1
15840593	Licoagrocarpin	RET
15840593	Licoagrocarpin	RORC
15840593	Licoagrocarpin	ROS1
15840593	Licoagrocarpin	RPS6KB1
15840593	Licoagrocarpin	S1PR1
15840593	Licoagrocarpin	S1PR3
15840593	Licoagrocarpin	SIGMAR1
15840593	Licoagrocarpin	SLC29A1
15840593	Licoagrocarpin	SMARCA2
15840593	Licoagrocarpin	STAT3
15840593	Licoagrocarpin	STAT6
15840593	Licoagrocarpin	SYK
15840593	Licoagrocarpin	TGFBR1
15840593	Licoagrocarpin	TGM2
15840593	Licoagrocarpin	THRA
15840593	Licoagrocarpin	TLR9
15840593	Licoagrocarpin	TNKS
15840593	Licoagrocarpin	TNKS2
15840593	Licoagrocarpin	TRPM8
15840593	Licoagrocarpin	TUBB1
15840593	Licoagrocarpin	TYR
15840593	Licoagrocarpin	WEE1
5317765	Glycyrrhiza flavonol A	ADRB1
5317765	Glycyrrhiza flavonol A	ADRB2
5317765	Glycyrrhiza flavonol A	AHR
5317765	Glycyrrhiza flavonol A	BACE1
5317765	Glycyrrhiza flavonol A	CHEK1
5317765	Glycyrrhiza flavonol A	CHEK2
5317765	Glycyrrhiza flavonol A	CYP19A1
5317765	Glycyrrhiza flavonol A	ESRRA

5317765	Glycyrrhiza flavonol A	GCGR
5317765	Glycyrrhiza flavonol A	HIF1A
5317765	Glycyrrhiza flavonol A	HSP90AA1
5317765	Glycyrrhiza flavonol A	HSP90AB1
5317765	Glycyrrhiza flavonol A	RPS6KA3
5317765	Glycyrrhiza flavonol A	TYR
5317765	Glycyrrhiza flavonol A	WEE1
5317765	Glycyrrhiza flavonol A	XDH
636883	Licoagroisoflavone	ACACB
636883	Licoagroisoflavone	ACHE
636883	Licoagroisoflavone	ACVR1
636883	Licoagroisoflavone	ADAM17
636883	Licoagroisoflavone	ADAMTS4
636883	Licoagroisoflavone	ADAMTS5
636883	Licoagroisoflavone	AKR1B10
636883	Licoagroisoflavone	AKT2
636883	Licoagroisoflavone	ALDH2
636883	Licoagroisoflavone	ALPL
636883	Licoagroisoflavone	APP
636883	Licoagroisoflavone	AURKA
636883	Licoagroisoflavone	AURKB
636883	Licoagroisoflavone	BLK
636883	Licoagroisoflavone	BMP4
636883	Licoagroisoflavone	BMX
636883	Licoagroisoflavone	BRAF
636883	Licoagroisoflavone	BTK
636883	Licoagroisoflavone	CCNA1
636883	Licoagroisoflavone	CCNA2
636883	Licoagroisoflavone	CDC25A
636883	Licoagroisoflavone	CDC25B
636883	Licoagroisoflavone	CDK1
636883	Licoagroisoflavone	CDK2
636883	Licoagroisoflavone	CDK2
636883	Licoagroisoflavone	CDK4
636883	Licoagroisoflavone	CES1
636883	Licoagroisoflavone	CES2
636883	Licoagroisoflavone	CHEK1
636883	Licoagroisoflavone	COMT
636883	Licoagroisoflavone	COQ8B
636883	Licoagroisoflavone	CSF1R
636883	Licoagroisoflavone	CSK
636883	Licoagroisoflavone	DNM1
636883	Licoagroisoflavone	EPHA1
636883	Licoagroisoflavone	EPHA2

636883	Licoagroisoflavone	EPHA3
636883	Licoagroisoflavone	EPHA4
636883	Licoagroisoflavone	EPHA5
636883	Licoagroisoflavone	EPHA6
636883	Licoagroisoflavone	EPHB2
636883	Licoagroisoflavone	EPHB3
636883	Licoagroisoflavone	EPHB4
636883	Licoagroisoflavone	ERBB2
636883	Licoagroisoflavone	ERN1
636883	Licoagroisoflavone	F10
636883	Licoagroisoflavone	FGR
636883	Licoagroisoflavone	FYN
636883	Licoagroisoflavone	GCGR
636883	Licoagroisoflavone	GSK3A
636883	Licoagroisoflavone	GSK3B
636883	Licoagroisoflavone	HDAC3
636883	Licoagroisoflavone	HDAC4
636883	Licoagroisoflavone	HPGDS
636883	Licoagroisoflavone	HSD11B1
636883	Licoagroisoflavone	HSD17B2
636883	Licoagroisoflavone	HSD17B3
636883	Licoagroisoflavone	IRAK4
636883	Licoagroisoflavone	KDR
636883	Licoagroisoflavone	KIT
636883	Licoagroisoflavone	LCK
636883	Licoagroisoflavone	LYN
636883	Licoagroisoflavone	MAPK14
636883	Licoagroisoflavone	MAPT
636883	Licoagroisoflavone	ME1
636883	Licoagroisoflavone	MET
636883	Licoagroisoflavone	MMP1
636883	Licoagroisoflavone	MMP13
636883	Licoagroisoflavone	MMP14
636883	Licoagroisoflavone	MMP2
636883	Licoagroisoflavone	MMP9
636883	Licoagroisoflavone	NAMPT
636883	Licoagroisoflavone	NOX1
636883	Licoagroisoflavone	NR1H4
636883	Licoagroisoflavone	ODC1
636883	Licoagroisoflavone	PARP1
636883	Licoagroisoflavone	PDE4A
636883	Licoagroisoflavone	PDE4B
636883	Licoagroisoflavone	PDE4C
636883	Licoagroisoflavone	PDGFRA

636883	Licoagroisoflavone	PDGFRB
636883	Licoagroisoflavone	PDK1
636883	Licoagroisoflavone	PIK3CA
636883	Licoagroisoflavone	PLAA
636883	Licoagroisoflavone	PTGES
636883	Licoagroisoflavone	PTK6
636883	Licoagroisoflavone	RET
636883	Licoagroisoflavone	ROCK1
636883	Licoagroisoflavone	ROCK2
636883	Licoagroisoflavone	SCD
636883	Licoagroisoflavone	SNCA
636883	Licoagroisoflavone	SRC
636883	Licoagroisoflavone	STK17B
636883	Licoagroisoflavone	SYK
636883	Licoagroisoflavone	TAS2R31
636883	Licoagroisoflavone	TGFBR1
636883	Licoagroisoflavone	THRA
636883	Licoagroisoflavone	TRPV1
636883	Licoagroisoflavone	TXK
636883	Licoagroisoflavone	TYRO3
636883	Licoagroisoflavone	WEE1
636883	Licoagroisoflavone	YES1
13965473	Odoratin	ABCB1
13965473	Odoratin	ABCG2
13965473	Odoratin	ACHE
13965473	Odoratin	ADORA1
13965473	Odoratin	ADORA2A
13965473	Odoratin	AKR1B1
13965473	Odoratin	ALDH2
13965473	Odoratin	ALOX12
13965473	Odoratin	ALOX15
13965473	Odoratin	ALPL
13965473	Odoratin	BACE1
13965473	Odoratin	CA1
13965473	Odoratin	CA12
13965473	Odoratin	CA2
13965473	Odoratin	CA4
13965473	Odoratin	CA7
13965473	Odoratin	CBR1
13965473	Odoratin	CDC25B
13965473	Odoratin	CHEK1
13965473	Odoratin	CYP19A1
13965473	Odoratin	DUSP3
13965473	Odoratin	EGFR

13965473	Odoratin	ESR1
13965473	Odoratin	ESR2
13965473	Odoratin	ESRRA
13965473	Odoratin	ESRRB
13965473	Odoratin	GCGR
13965473	Odoratin	HSD17B1
13965473	Odoratin	HSD17B2
13965473	Odoratin	HSP90AA1
13965473	Odoratin	HSP90AB1
13965473	Odoratin	HSP90B1
13965473	Odoratin	HTR2A
13965473	Odoratin	HTR2C
13965473	Odoratin	IGFBP1
13965473	Odoratin	IGFBP2
13965473	Odoratin	IGFBP3
13965473	Odoratin	IGFBP4
13965473	Odoratin	IGFBP5
13965473	Odoratin	IGFBP6
13965473	Odoratin	IL2
13965473	Odoratin	KIT
13965473	Odoratin	MAOA
13965473	Odoratin	MAOB
13965473	Odoratin	MGAM
13965473	Odoratin	MIF
13965473	Odoratin	NOX4
13965473	Odoratin	OPRD1
13965473	Odoratin	PDE10A
13965473	Odoratin	PFKFB3
13965473	Odoratin	PLAU
13965473	Odoratin	PON1
13965473	Odoratin	PPARA
13965473	Odoratin	PTGS1
13965473	Odoratin	PTPN1
13965473	Odoratin	PTPRS
13965473	Odoratin	SLC6A2
13965473	Odoratin	STS
13965473	Odoratin	TBXAS1
13965473	Odoratin	TLR9
13965473	Odoratin	TNNT2
13965473	Odoratin	TYR
13965473	Odoratin	WEE1
13965473	Odoratin	XDH
14769500	Xambioona	CTSK
14769500	Xambioona	ODC1

354368	7-Methoxy-2-methyl isoflavone	ABAT
354368	7-Methoxy-2-methyl isoflavone	ABCB1
354368	7-Methoxy-2-methyl isoflavone	ABCG2
354368	7-Methoxy-2-methyl isoflavone	ACHE
354368	7-Methoxy-2-methyl isoflavone	ADORA1
354368	7-Methoxy-2-methyl isoflavone	ADORA2A
354368	7-Methoxy-2-methyl isoflavone	ALDH2
354368	7-Methoxy-2-methyl isoflavone	ALOX12
354368	7-Methoxy-2-methyl isoflavone	ALOX5
354368	7-Methoxy-2-methyl isoflavone	APH1A
354368	7-Methoxy-2-methyl isoflavone	APH1B
354368	7-Methoxy-2-methyl isoflavone	AURKA
354368	7-Methoxy-2-methyl isoflavone	CA1
354368	7-Methoxy-2-methyl isoflavone	CA12
354368	7-Methoxy-2-methyl isoflavone	CA2
354368	7-Methoxy-2-methyl isoflavone	CA4
354368	7-Methoxy-2-methyl isoflavone	CA7
354368	7-Methoxy-2-methyl isoflavone	CACNA2D1
354368	7-Methoxy-2-methyl isoflavone	CBR1
354368	7-Methoxy-2-methyl isoflavone	CHRNA7
354368	7-Methoxy-2-methyl isoflavone	CRHR1
354368	7-Methoxy-2-methyl isoflavone	CXCR2
354368	7-Methoxy-2-methyl isoflavone	CYP17A1
354368	7-Methoxy-2-methyl isoflavone	CYP19A1
354368	7-Methoxy-2-methyl isoflavone	CYP1B1
354368	7-Methoxy-2-methyl isoflavone	CYP27A1
354368	7-Methoxy-2-methyl isoflavone	EGFR
354368	7-Methoxy-2-methyl isoflavone	ESR1
354368	7-Methoxy-2-methyl isoflavone	ESR2
354368	7-Methoxy-2-methyl isoflavone	ESRRA
354368	7-Methoxy-2-methyl isoflavone	ESRRB
354368	7-Methoxy-2-methyl isoflavone	FLT1
354368	7-Methoxy-2-methyl isoflavone	FLT3
354368	7-Methoxy-2-methyl isoflavone	FNTA
354368	7-Methoxy-2-methyl isoflavone	FNTA
354368	7-Methoxy-2-methyl isoflavone	FNTB
354368	7-Methoxy-2-methyl isoflavone	FPR2
354368	7-Methoxy-2-methyl isoflavone	GPBAR1
354368	7-Methoxy-2-methyl isoflavone	GRM1
354368	7-Methoxy-2-methyl isoflavone	GRM2
354368	7-Methoxy-2-methyl isoflavone	GRM5
354368	7-Methoxy-2-methyl isoflavone	HCRTR1
354368	7-Methoxy-2-methyl isoflavone	HCRTR2
354368	7-Methoxy-2-methyl isoflavone	HDAC1

354368	7-Methoxy-2-methyl isoflavone	HDAC6
354368	7-Methoxy-2-methyl isoflavone	HSD17B1
354368	7-Methoxy-2-methyl isoflavone	HSD17B2
354368	7-Methoxy-2-methyl isoflavone	HTR2A
354368	7-Methoxy-2-methyl isoflavone	HTR2C
354368	7-Methoxy-2-methyl isoflavone	HTR7
354368	7-Methoxy-2-methyl isoflavone	IDO1
354368	7-Methoxy-2-methyl isoflavone	IL2
354368	7-Methoxy-2-methyl isoflavone	JAK2
354368	7-Methoxy-2-methyl isoflavone	JAK3
354368	7-Methoxy-2-methyl isoflavone	KCNA5
354368	7-Methoxy-2-methyl isoflavone	KDM1A
354368	7-Methoxy-2-methyl isoflavone	LRRK2
354368	7-Methoxy-2-methyl isoflavone	MAOA
354368	7-Methoxy-2-methyl isoflavone	MAOB
354368	7-Methoxy-2-methyl isoflavone	MCL1
354368	7-Methoxy-2-methyl isoflavone	MGAM
354368	7-Methoxy-2-methyl isoflavone	MGLL
354368	7-Methoxy-2-methyl isoflavone	MMP1
354368	7-Methoxy-2-methyl isoflavone	MTNR1A
354368	7-Methoxy-2-methyl isoflavone	MTNR1B
354368	7-Methoxy-2-methyl isoflavone	NAAA
354368	7-Methoxy-2-methyl isoflavone	NCSTN
354368	7-Methoxy-2-methyl isoflavone	NR3C1
354368	7-Methoxy-2-methyl isoflavone	NR3C2
354368	7-Methoxy-2-methyl isoflavone	NTRK1
354368	7-Methoxy-2-methyl isoflavone	P2RX7
354368	7-Methoxy-2-methyl isoflavone	PARP2
354368	7-Methoxy-2-methyl isoflavone	PDE10A
354368	7-Methoxy-2-methyl isoflavone	PDE1B
354368	7-Methoxy-2-methyl isoflavone	PDE4A
354368	7-Methoxy-2-methyl isoflavone	PDE4C
354368	7-Methoxy-2-methyl isoflavone	PDE7A
354368	7-Methoxy-2-methyl isoflavone	PDGFRA
354368	7-Methoxy-2-methyl isoflavone	PDGFRB
354368	7-Methoxy-2-methyl isoflavone	PFKFB3
354368	7-Methoxy-2-methyl isoflavone	PGGT1B
354368	7-Methoxy-2-methyl isoflavone	PGK1
354368	7-Methoxy-2-methyl isoflavone	PGR
354368	7-Methoxy-2-methyl isoflavone	PIK3CA
354368	7-Methoxy-2-methyl isoflavone	PON1
354368	7-Methoxy-2-methyl isoflavone	PPARG
354368	7-Methoxy-2-methyl isoflavone	PRKCA
354368	7-Methoxy-2-methyl isoflavone	PRKCG

354368	7-Methoxy-2-methyl isoflavone	PSEN1
354368	7-Methoxy-2-methyl isoflavone	PSEN2
354368	7-Methoxy-2-methyl isoflavone	PSENEN
354368	7-Methoxy-2-methyl isoflavone	PTGS1
354368	7-Methoxy-2-methyl isoflavone	PTPRS
354368	7-Methoxy-2-methyl isoflavone	QPCT
354368	7-Methoxy-2-methyl isoflavone	ROCK2
354368	7-Methoxy-2-methyl isoflavone	RPS6KB1
354368	7-Methoxy-2-methyl isoflavone	SIRT2
354368	7-Methoxy-2-methyl isoflavone	SIRT3
354368	7-Methoxy-2-methyl isoflavone	SLC5A7
354368	7-Methoxy-2-methyl isoflavone	SLC6A2
354368	7-Methoxy-2-methyl isoflavone	TACR1
354368	7-Methoxy-2-methyl isoflavone	TBXAS1
354368	7-Methoxy-2-methyl isoflavone	TGFBR1
354368	7-Methoxy-2-methyl isoflavone	TGM2
354368	7-Methoxy-2-methyl isoflavone	TNKS
354368	7-Methoxy-2-methyl isoflavone	TRPM8
354368	7-Methoxy-2-methyl isoflavone	TYR
354368	7-Methoxy-2-methyl isoflavone	UTS2R
5280378	formononetin	ABCB1
5280378	formononetin	ABCG2
5280378	formononetin	ACHE
5280378	formononetin	ADORA1
5280378	formononetin	ADORA2A
5280378	formononetin	ALDH2
5280378	formononetin	ALOX12
5280378	formononetin	ALOX15
5280378	formononetin	BACE1
5280378	formononetin	CA1
5280378	formononetin	CA12
5280378	formononetin	CA13
5280378	formononetin	CA14
5280378	formononetin	CA2
5280378	formononetin	CA3
5280378	formononetin	CA4
5280378	formononetin	CA5A
5280378	formononetin	CA5B
5280378	formononetin	CA6
5280378	formononetin	CA7
5280378	formononetin	CA9
5280378	formononetin	CBR1
5280378	formononetin	CYP19A1
5280378	formononetin	DHODH

5280378	formononetin	EGFR
5280378	formononetin	ERCC5
5280378	formononetin	ESR1
5280378	formononetin	ESR2
5280378	formononetin	ESRRA
5280378	formononetin	ESRRB
5280378	formononetin	FEN1
5280378	formononetin	HSD17B1
5280378	formononetin	HSD17B2
5280378	formononetin	HTR2A
5280378	formononetin	HTR2C
5280378	formononetin	IL2
5280378	formononetin	MAOA
5280378	formononetin	MAOB
5280378	formononetin	MGAM
5280378	formononetin	MIF
5280378	formononetin	NOX4
5280378	formononetin	PFKFB3
5280378	formononetin	PON1
5280378	formononetin	PPARA
5280378	formononetin	PTGS1
5280378	formononetin	PTPN1
5280378	formononetin	PTPRS
5280378	formononetin	RAF1
5280378	formononetin	SLC6A2
5280378	formononetin	STS
5280378	formononetin	TBXAS1
5280378	formononetin	TLR9
5280378	formononetin	TNKS
5280378	formononetin	TNKS2
5280378	formononetin	TYR
5280378	formononetin	XDH
323	Coumarin	ACHE
323	Coumarin	CA1
323	Coumarin	CA12
323	Coumarin	CA13
323	Coumarin	CA14
323	Coumarin	CA2
323	Coumarin	CA3
323	Coumarin	CA4
323	Coumarin	CA5A
323	Coumarin	CA5B
323	Coumarin	CA6
323	Coumarin	CA7

323	Coumarin	CA9
323	Coumarin	DAO
323	Coumarin	EGFR
323	Coumarin	MAOA
323	Coumarin	NFKB1
323	Coumarin	NQO1
323	Coumarin	XDH
11604108	(-)-Syringaresinol	ABCG2
11604108	(-)-Syringaresinol	ABL1
11604108	(-)-Syringaresinol	ADAM33
11604108	(-)-Syringaresinol	ADRA2A
11604108	(-)-Syringaresinol	ALOX12
11604108	(-)-Syringaresinol	ALOX5
11604108	(-)-Syringaresinol	BDKRB1
11604108	(-)-Syringaresinol	BMP1
11604108	(-)-Syringaresinol	CASP1
11604108	(-)-Syringaresinol	CCKAR
11604108	(-)-Syringaresinol	CCKBR
11604108	(-)-Syringaresinol	CCNB3
11604108	(-)-Syringaresinol	CCR4
11604108	(-)-Syringaresinol	CDK1
11604108	(-)-Syringaresinol	CDK2
11604108	(-)-Syringaresinol	CHEK1
11604108	(-)-Syringaresinol	CSF1R
11604108	(-)-Syringaresinol	CXCR1
11604108	(-)-Syringaresinol	CXCR2
11604108	(-)-Syringaresinol	EIF2AK3
11604108	(-)-Syringaresinol	FCER2
11604108	(-)-Syringaresinol	GSK3B
11604108	(-)-Syringaresinol	HCK
11604108	(-)-Syringaresinol	HIF1A
11604108	(-)-Syringaresinol	IMPDH2
11604108	(-)-Syringaresinol	JAK3
11604108	(-)-Syringaresinol	LRRK2
11604108	(-)-Syringaresinol	MAP3K12
11604108	(-)-Syringaresinol	MAPK9
11604108	(-)-Syringaresinol	MCL1
11604108	(-)-Syringaresinol	MMP1
11604108	(-)-Syringaresinol	MMP7
11604108	(-)-Syringaresinol	MMP8
11604108	(-)-Syringaresinol	MPI
11604108	(-)-Syringaresinol	MTOR
11604108	(-)-Syringaresinol	OPRM1
11604108	(-)-Syringaresinol	P2RX3

11604108	(-)-Syringaresinol	PDK1
11604108	(-)-Syringaresinol	PI4KB
11604108	(-)-Syringaresinol	PIK3CA
11604108	(-)-Syringaresinol	PIK3CB
11604108	(-)-Syringaresinol	PIK3CD
11604108	(-)-Syringaresinol	PIK3CG
11604108	(-)-Syringaresinol	PLA2G7
11604108	(-)-Syringaresinol	PRKDC
11604108	(-)-Syringaresinol	PTAFR
11604108	(-)-Syringaresinol	RPS6KA3
11604108	(-)-Syringaresinol	SHBG
11604108	(-)-Syringaresinol	SLC5A2
11604108	(-)-Syringaresinol	SLC6A2
11604108	(-)-Syringaresinol	SLC6A4
11604108	(-)-Syringaresinol	SOAT1
11604108	(-)-Syringaresinol	SOAT2
11604108	(-)-Syringaresinol	SYK
11604108	(-)-Syringaresinol	TOP1
11604108	(-)-Syringaresinol	WEE1
614467	5-(2-Methyl-1,3-thiazol-4-yl)thiophene-2-carboxylic acid	AKR1B1
614467	5-(2-Methyl-1,3-thiazol-4-yl)thiophene-2-carboxylic acid	APEX1
614467	5-(2-Methyl-1,3-thiazol-4-yl)thiophene-2-carboxylic acid	CA1
614467	5-(2-Methyl-1,3-thiazol-4-yl)thiophene-2-carboxylic acid	CA12
614467	5-(2-Methyl-1,3-thiazol-4-yl)thiophene-2-carboxylic acid	CA13
614467	5-(2-Methyl-1,3-thiazol-4-yl)thiophene-2-carboxylic acid	CA14
614467	5-(2-Methyl-1,3-thiazol-4-yl)thiophene-2-carboxylic acid	CA3
614467	5-(2-Methyl-1,3-thiazol-4-yl)thiophene-2-carboxylic acid	CA5A
614467	5-(2-Methyl-1,3-thiazol-4-yl)thiophene-2-carboxylic acid	CA5B
614467	5-(2-Methyl-1,3-thiazol-4-yl)thiophene-2-carboxylic acid	CA9
614467	5-(2-Methyl-1,3-thiazol-4-yl)thiophene-2-carboxylic acid	CCNC
614467	5-(2-Methyl-1,3-thiazol-4-yl)thiophene-2-carboxylic acid	CHRNA4

614467	5-(2-Methyl-1,3-thiazol-4-yl)thiophene-2-carboxylic acid	CSNK2A1
614467	5-(2-Methyl-1,3-thiazol-4-yl)thiophene-2-carboxylic acid	ERCC5
614467	5-(2-Methyl-1,3-thiazol-4-yl)thiophene-2-carboxylic acid	ESR2
614467	5-(2-Methyl-1,3-thiazol-4-yl)thiophene-2-carboxylic acid	FEN1
614467	5-(2-Methyl-1,3-thiazol-4-yl)thiophene-2-carboxylic acid	GRK2
614467	5-(2-Methyl-1,3-thiazol-4-yl)thiophene-2-carboxylic acid	HCAR2
614467	5-(2-Methyl-1,3-thiazol-4-yl)thiophene-2-carboxylic acid	ITGAL
614467	5-(2-Methyl-1,3-thiazol-4-yl)thiophene-2-carboxylic acid	KDM2A
614467	5-(2-Methyl-1,3-thiazol-4-yl)thiophene-2-carboxylic acid	KDM3A
614467	5-(2-Methyl-1,3-thiazol-4-yl)thiophene-2-carboxylic acid	KDM4A
614467	5-(2-Methyl-1,3-thiazol-4-yl)thiophene-2-carboxylic acid	KDM4B
614467	5-(2-Methyl-1,3-thiazol-4-yl)thiophene-2-carboxylic acid	KDM4C
614467	5-(2-Methyl-1,3-thiazol-4-yl)thiophene-2-carboxylic acid	KDM4D
614467	5-(2-Methyl-1,3-thiazol-4-yl)thiophene-2-carboxylic acid	KDM4E
614467	5-(2-Methyl-1,3-thiazol-4-yl)thiophene-2-carboxylic acid	KDM5B
614467	5-(2-Methyl-1,3-thiazol-4-yl)thiophene-2-carboxylic acid	KDM5C
614467	5-(2-Methyl-1,3-thiazol-4-yl)thiophene-2-carboxylic acid	KDM6B
614467	5-(2-Methyl-1,3-thiazol-4-yl)thiophene-2-carboxylic acid	KMO
614467	5-(2-Methyl-1,3-thiazol-4-yl)thiophene-2-carboxylic acid	MAP3K8
614467	5-(2-Methyl-1,3-thiazol-4-yl)thiophene-2-carboxylic acid	NOTUM
614467	5-(2-Methyl-1,3-thiazol-4-yl)thiophene-2-carboxylic acid	PAM
614467	5-(2-Methyl-1,3-thiazol-4-yl)thiophene-2-carboxylic acid	PARP1

614467	5-(2-Methyl-1,3-thiazol-4-yl)thiophene-2-carboxylic acid	PIM1
614467	5-(2-Methyl-1,3-thiazol-4-yl)thiophene-2-carboxylic acid	SLC16A1
614467	5-(2-Methyl-1,3-thiazol-4-yl)thiophene-2-carboxylic acid	SLC16A3
614467	5-(2-Methyl-1,3-thiazol-4-yl)thiophene-2-carboxylic acid	SLCO1B1
614467	5-(2-Methyl-1,3-thiazol-4-yl)thiophene-2-carboxylic acid	SORT1
614467	5-(2-Methyl-1,3-thiazol-4-yl)thiophene-2-carboxylic acid	TAS1R3
614467	5-(2-Methyl-1,3-thiazol-4-yl)thiophene-2-carboxylic acid	TBXAS1
2794766	2-[4-(Trifluoromethyl)phenyl]-1,3-thiazole-4-carboxylic Acid	HTR2B
2794766	2-[4-(Trifluoromethyl)phenyl]-1,3-thiazole-4-carboxylic Acid	KMO
2794766	2-[4-(Trifluoromethyl)phenyl]-1,3-thiazole-4-carboxylic Acid	PTPN1
135567045	2-(4-((Pyridin-4-Yl)methyl)piperazin-1-Yl)-3,4,5,6,7,8-Hexahydroquinazolin-4-One	ABL1
135567045	2-(4-((Pyridin-4-Yl)methyl)piperazin-1-Yl)-3,4,5,6,7,8-Hexahydroquinazolin-4-One	ACACA
135567045	2-(4-((Pyridin-4-Yl)methyl)piperazin-1-Yl)-3,4,5,6,7,8-Hexahydroquinazolin-4-One	ACACB
135567045	2-(4-((Pyridin-4-Yl)methyl)piperazin-1-Yl)-3,4,5,6,7,8-Hexahydroquinazolin-4-One	ADAM17
135567045	2-(4-((Pyridin-4-Yl)methyl)piperazin-1-Yl)-3,4,5,6,7,8-Hexahydroquinazolin-4-One	ADRA1A
135567045	2-(4-((Pyridin-4-Yl)methyl)piperazin-1-Yl)-3,4,5,6,7,8-Hexahydroquinazolin-4-One	ADRA1D

135567045	2-(4-((Pyridin-4-Yl)methyl)piperazin-1-Yl)-3,4,5,6,7,8-Hexahydroquinazolin-4-One	ALK
135567045	2-(4-((Pyridin-4-Yl)methyl)piperazin-1-Yl)-3,4,5,6,7,8-Hexahydroquinazolin-4-One	ASAH1
135567045	2-(4-((Pyridin-4-Yl)methyl)piperazin-1-Yl)-3,4,5,6,7,8-Hexahydroquinazolin-4-One	AURKA
135567045	2-(4-((Pyridin-4-Yl)methyl)piperazin-1-Yl)-3,4,5,6,7,8-Hexahydroquinazolin-4-One	BRD4
135567045	2-(4-((Pyridin-4-Yl)methyl)piperazin-1-Yl)-3,4,5,6,7,8-Hexahydroquinazolin-4-One	CALCRL
135567045	2-(4-((Pyridin-4-Yl)methyl)piperazin-1-Yl)-3,4,5,6,7,8-Hexahydroquinazolin-4-One	CDC7
135567045	2-(4-((Pyridin-4-Yl)methyl)piperazin-1-Yl)-3,4,5,6,7,8-Hexahydroquinazolin-4-One	CDK1
135567045	2-(4-((Pyridin-4-Yl)methyl)piperazin-1-Yl)-3,4,5,6,7,8-Hexahydroquinazolin-4-One	CDK1
135567045	2-(4-((Pyridin-4-Yl)methyl)piperazin-1-Yl)-3,4,5,6,7,8-Hexahydroquinazolin-4-One	CDK2
135567045	2-(4-((Pyridin-4-Yl)methyl)piperazin-1-Yl)-3,4,5,6,7,8-Hexahydroquinazolin-4-One	CDK2
135567045	2-(4-((Pyridin-4-Yl)methyl)piperazin-1-Yl)-3,4,5,6,7,8-Hexahydroquinazolin-4-One	CDK9

135567045	2-(4-((Pyridin-4-Yl)methyl)piperazin-1-Yl)-3,4,5,6,7,8-Hexahydroquinazolin-4-One	CFTR
135567045	2-(4-((Pyridin-4-Yl)methyl)piperazin-1-Yl)-3,4,5,6,7,8-Hexahydroquinazolin-4-One	CLK4
135567045	2-(4-((Pyridin-4-Yl)methyl)piperazin-1-Yl)-3,4,5,6,7,8-Hexahydroquinazolin-4-One	CNR1
135567045	2-(4-((Pyridin-4-Yl)methyl)piperazin-1-Yl)-3,4,5,6,7,8-Hexahydroquinazolin-4-One	CNR2
135567045	2-(4-((Pyridin-4-Yl)methyl)piperazin-1-Yl)-3,4,5,6,7,8-Hexahydroquinazolin-4-One	CTSK
135567045	2-(4-((Pyridin-4-Yl)methyl)piperazin-1-Yl)-3,4,5,6,7,8-Hexahydroquinazolin-4-One	CTSL
135567045	2-(4-((Pyridin-4-Yl)methyl)piperazin-1-Yl)-3,4,5,6,7,8-Hexahydroquinazolin-4-One	CTSS
135567045	2-(4-((Pyridin-4-Yl)methyl)piperazin-1-Yl)-3,4,5,6,7,8-Hexahydroquinazolin-4-One	CTSV
135567045	2-(4-((Pyridin-4-Yl)methyl)piperazin-1-Yl)-3,4,5,6,7,8-Hexahydroquinazolin-4-One	CYP19A1
135567045	2-(4-((Pyridin-4-Yl)methyl)piperazin-1-Yl)-3,4,5,6,7,8-Hexahydroquinazolin-4-One	DGAT1
135567045	2-(4-((Pyridin-4-Yl)methyl)piperazin-1-Yl)-3,4,5,6,7,8-Hexahydroquinazolin-4-One	DRD4

135567045	2-(4-((Pyridin-4-Yl)methyl)piperazin-1-Yl)-3,4,5,6,7,8-Hexahydroquinazolin-4-One	ELANE
135567045	2-(4-((Pyridin-4-Yl)methyl)piperazin-1-Yl)-3,4,5,6,7,8-Hexahydroquinazolin-4-One	EPHX2
135567045	2-(4-((Pyridin-4-Yl)methyl)piperazin-1-Yl)-3,4,5,6,7,8-Hexahydroquinazolin-4-One	EZR
135567045	2-(4-((Pyridin-4-Yl)methyl)piperazin-1-Yl)-3,4,5,6,7,8-Hexahydroquinazolin-4-One	F10
135567045	2-(4-((Pyridin-4-Yl)methyl)piperazin-1-Yl)-3,4,5,6,7,8-Hexahydroquinazolin-4-One	F13A1
135567045	2-(4-((Pyridin-4-Yl)methyl)piperazin-1-Yl)-3,4,5,6,7,8-Hexahydroquinazolin-4-One	F2
135567045	2-(4-((Pyridin-4-Yl)methyl)piperazin-1-Yl)-3,4,5,6,7,8-Hexahydroquinazolin-4-One	FAAH
135567045	2-(4-((Pyridin-4-Yl)methyl)piperazin-1-Yl)-3,4,5,6,7,8-Hexahydroquinazolin-4-One	FASN
135567045	2-(4-((Pyridin-4-Yl)methyl)piperazin-1-Yl)-3,4,5,6,7,8-Hexahydroquinazolin-4-One	FLT1
135567045	2-(4-((Pyridin-4-Yl)methyl)piperazin-1-Yl)-3,4,5,6,7,8-Hexahydroquinazolin-4-One	GHSR
135567045	2-(4-((Pyridin-4-Yl)methyl)piperazin-1-Yl)-3,4,5,6,7,8-Hexahydroquinazolin-4-One	GPBAR1

135567045	2-(4-((Pyridin-4-Yl)methyl)piperazin-1-Yl)-3,4,5,6,7,8-Hexahydroquinazolin-4-One	GRM1
135567045	2-(4-((Pyridin-4-Yl)methyl)piperazin-1-Yl)-3,4,5,6,7,8-Hexahydroquinazolin-4-One	GSK3A
135567045	2-(4-((Pyridin-4-Yl)methyl)piperazin-1-Yl)-3,4,5,6,7,8-Hexahydroquinazolin-4-One	GSK3B
135567045	2-(4-((Pyridin-4-Yl)methyl)piperazin-1-Yl)-3,4,5,6,7,8-Hexahydroquinazolin-4-One	HDAC1
135567045	2-(4-((Pyridin-4-Yl)methyl)piperazin-1-Yl)-3,4,5,6,7,8-Hexahydroquinazolin-4-One	HDAC10
135567045	2-(4-((Pyridin-4-Yl)methyl)piperazin-1-Yl)-3,4,5,6,7,8-Hexahydroquinazolin-4-One	HDAC11
135567045	2-(4-((Pyridin-4-Yl)methyl)piperazin-1-Yl)-3,4,5,6,7,8-Hexahydroquinazolin-4-One	HDAC2
135567045	2-(4-((Pyridin-4-Yl)methyl)piperazin-1-Yl)-3,4,5,6,7,8-Hexahydroquinazolin-4-One	HDAC3
135567045	2-(4-((Pyridin-4-Yl)methyl)piperazin-1-Yl)-3,4,5,6,7,8-Hexahydroquinazolin-4-One	HDAC6
135567045	2-(4-((Pyridin-4-Yl)methyl)piperazin-1-Yl)-3,4,5,6,7,8-Hexahydroquinazolin-4-One	HDAC8
135567045	2-(4-((Pyridin-4-Yl)methyl)piperazin-1-Yl)-3,4,5,6,7,8-Hexahydroquinazolin-4-One	IMPDH2

135567045	2-(4-((Pyridin-4-Yl)methyl)piperazin-1-Yl)-3,4,5,6,7,8-Hexahydroquinazolin-4-One	ITK
135567045	2-(4-((Pyridin-4-Yl)methyl)piperazin-1-Yl)-3,4,5,6,7,8-Hexahydroquinazolin-4-One	KDR
135567045	2-(4-((Pyridin-4-Yl)methyl)piperazin-1-Yl)-3,4,5,6,7,8-Hexahydroquinazolin-4-One	LCK
135567045	2-(4-((Pyridin-4-Yl)methyl)piperazin-1-Yl)-3,4,5,6,7,8-Hexahydroquinazolin-4-One	LRRK2
135567045	2-(4-((Pyridin-4-Yl)methyl)piperazin-1-Yl)-3,4,5,6,7,8-Hexahydroquinazolin-4-One	MAPK1
135567045	2-(4-((Pyridin-4-Yl)methyl)piperazin-1-Yl)-3,4,5,6,7,8-Hexahydroquinazolin-4-One	MAPK10
135567045	2-(4-((Pyridin-4-Yl)methyl)piperazin-1-Yl)-3,4,5,6,7,8-Hexahydroquinazolin-4-One	MAPK14
135567045	2-(4-((Pyridin-4-Yl)methyl)piperazin-1-Yl)-3,4,5,6,7,8-Hexahydroquinazolin-4-One	MAPK8
135567045	2-(4-((Pyridin-4-Yl)methyl)piperazin-1-Yl)-3,4,5,6,7,8-Hexahydroquinazolin-4-One	MAPK9
135567045	2-(4-((Pyridin-4-Yl)methyl)piperazin-1-Yl)-3,4,5,6,7,8-Hexahydroquinazolin-4-One	MAPKAPK2
135567045	2-(4-((Pyridin-4-Yl)methyl)piperazin-1-Yl)-3,4,5,6,7,8-Hexahydroquinazolin-4-One	MCHR1

135567045	2-(4-((Pyridin-4-Yl)methyl)piperazin-1-Yl)-3,4,5,6,7,8-Hexahydroquinazolin-4-One	MET
135567045	2-(4-((Pyridin-4-Yl)methyl)piperazin-1-Yl)-3,4,5,6,7,8-Hexahydroquinazolin-4-One	MGLL
135567045	2-(4-((Pyridin-4-Yl)methyl)piperazin-1-Yl)-3,4,5,6,7,8-Hexahydroquinazolin-4-One	MMP1
135567045	2-(4-((Pyridin-4-Yl)methyl)piperazin-1-Yl)-3,4,5,6,7,8-Hexahydroquinazolin-4-One	MTNR1A
135567045	2-(4-((Pyridin-4-Yl)methyl)piperazin-1-Yl)-3,4,5,6,7,8-Hexahydroquinazolin-4-One	MTNR1B
135567045	2-(4-((Pyridin-4-Yl)methyl)piperazin-1-Yl)-3,4,5,6,7,8-Hexahydroquinazolin-4-One	NAMPT
135567045	2-(4-((Pyridin-4-Yl)methyl)piperazin-1-Yl)-3,4,5,6,7,8-Hexahydroquinazolin-4-One	NPY5R
135567045	2-(4-((Pyridin-4-Yl)methyl)piperazin-1-Yl)-3,4,5,6,7,8-Hexahydroquinazolin-4-One	NR4A2
135567045	2-(4-((Pyridin-4-Yl)methyl)piperazin-1-Yl)-3,4,5,6,7,8-Hexahydroquinazolin-4-One	P2RX7
135567045	2-(4-((Pyridin-4-Yl)methyl)piperazin-1-Yl)-3,4,5,6,7,8-Hexahydroquinazolin-4-One	PARP1
135567045	2-(4-((Pyridin-4-Yl)methyl)piperazin-1-Yl)-3,4,5,6,7,8-Hexahydroquinazolin-4-One	PARP10

135567045	2-(4-((Pyridin-4-Yl)methyl)piperazin-1-Yl)-3,4,5,6,7,8-Hexahydroquinazolin-4-One	PARP2
135567045	2-(4-((Pyridin-4-Yl)methyl)piperazin-1-Yl)-3,4,5,6,7,8-Hexahydroquinazolin-4-One	PDE10A
135567045	2-(4-((Pyridin-4-Yl)methyl)piperazin-1-Yl)-3,4,5,6,7,8-Hexahydroquinazolin-4-One	PDE3A
135567045	2-(4-((Pyridin-4-Yl)methyl)piperazin-1-Yl)-3,4,5,6,7,8-Hexahydroquinazolin-4-One	PDE3B
135567045	2-(4-((Pyridin-4-Yl)methyl)piperazin-1-Yl)-3,4,5,6,7,8-Hexahydroquinazolin-4-One	PDE4B
135567045	2-(4-((Pyridin-4-Yl)methyl)piperazin-1-Yl)-3,4,5,6,7,8-Hexahydroquinazolin-4-One	PDE7A
135567045	2-(4-((Pyridin-4-Yl)methyl)piperazin-1-Yl)-3,4,5,6,7,8-Hexahydroquinazolin-4-One	PDGFRA
135567045	2-(4-((Pyridin-4-Yl)methyl)piperazin-1-Yl)-3,4,5,6,7,8-Hexahydroquinazolin-4-One	PLAU
135567045	2-(4-((Pyridin-4-Yl)methyl)piperazin-1-Yl)-3,4,5,6,7,8-Hexahydroquinazolin-4-One	PTGS2
135567045	2-(4-((Pyridin-4-Yl)methyl)piperazin-1-Yl)-3,4,5,6,7,8-Hexahydroquinazolin-4-One	RBBP9
135567045	2-(4-((Pyridin-4-Yl)methyl)piperazin-1-Yl)-3,4,5,6,7,8-Hexahydroquinazolin-4-One	RET

135567045	2-(4-((Pyridin-4-Yl)methyl)piperazin-1-Yl)-3,4,5,6,7,8-Hexahydroquinazolin-4-One	ROCK2
135567045	2-(4-((Pyridin-4-Yl)methyl)piperazin-1-Yl)-3,4,5,6,7,8-Hexahydroquinazolin-4-One	RORC
135567045	2-(4-((Pyridin-4-Yl)methyl)piperazin-1-Yl)-3,4,5,6,7,8-Hexahydroquinazolin-4-One	RPS6KA2
135567045	2-(4-((Pyridin-4-Yl)methyl)piperazin-1-Yl)-3,4,5,6,7,8-Hexahydroquinazolin-4-One	SCARB1
135567045	2-(4-((Pyridin-4-Yl)methyl)piperazin-1-Yl)-3,4,5,6,7,8-Hexahydroquinazolin-4-One	SERPINE1
135567045	2-(4-((Pyridin-4-Yl)methyl)piperazin-1-Yl)-3,4,5,6,7,8-Hexahydroquinazolin-4-One	SLC27A1
135567045	2-(4-((Pyridin-4-Yl)methyl)piperazin-1-Yl)-3,4,5,6,7,8-Hexahydroquinazolin-4-One	SLC6A9
135567045	2-(4-((Pyridin-4-Yl)methyl)piperazin-1-Yl)-3,4,5,6,7,8-Hexahydroquinazolin-4-One	SLC9A1
135567045	2-(4-((Pyridin-4-Yl)methyl)piperazin-1-Yl)-3,4,5,6,7,8-Hexahydroquinazolin-4-One	SRC
135567045	2-(4-((Pyridin-4-Yl)methyl)piperazin-1-Yl)-3,4,5,6,7,8-Hexahydroquinazolin-4-One	TEK
135567045	2-(4-((Pyridin-4-Yl)methyl)piperazin-1-Yl)-3,4,5,6,7,8-Hexahydroquinazolin-4-One	TGM1

135567045	2-(4-((Pyridin-4-Yl)methyl)piperazin-1-Yl)-3,4,5,6,7,8-Hexahydroquinazolin-4-One	TGM2
135567045	2-(4-((Pyridin-4-Yl)methyl)piperazin-1-Yl)-3,4,5,6,7,8-Hexahydroquinazolin-4-One	TNKS
135567045	2-(4-((Pyridin-4-Yl)methyl)piperazin-1-Yl)-3,4,5,6,7,8-Hexahydroquinazolin-4-One	TNKS2
135567045	2-(4-((Pyridin-4-Yl)methyl)piperazin-1-Yl)-3,4,5,6,7,8-Hexahydroquinazolin-4-One	TRPA1
135567045	2-(4-((Pyridin-4-Yl)methyl)piperazin-1-Yl)-3,4,5,6,7,8-Hexahydroquinazolin-4-One	TRPV1
135567045	2-(4-((Pyridin-4-Yl)methyl)piperazin-1-Yl)-3,4,5,6,7,8-Hexahydroquinazolin-4-One	TRPV4
21594250	Macharinic acid lactone	ADORA2A
21594250	Macharinic acid lactone	AOC1
21594250	Macharinic acid lactone	ASF1A
21594250	Macharinic acid lactone	BACE2
21594250	Macharinic acid lactone	BRAF
21594250	Macharinic acid lactone	CASP6
21594250	Macharinic acid lactone	CASR
21594250	Macharinic acid lactone	CDC7
21594250	Macharinic acid lactone	CDK1
21594250	Macharinic acid lactone	CDK2
21594250	Macharinic acid lactone	CDK5R1
21594250	Macharinic acid lactone	CHEK1
21594250	Macharinic acid lactone	CXCR2
21594250	Macharinic acid lactone	CYP11B1
21594250	Macharinic acid lactone	CYP11B2
21594250	Macharinic acid lactone	DAO
21594250	Macharinic acid lactone	DHFR
21594250	Macharinic acid lactone	DRD4
21594250	Macharinic acid lactone	EGLN1
21594250	Macharinic acid lactone	ERN1

21594250	Macharinic acid lactone	FAP
21594250	Macharinic acid lactone	FGFR1
21594250	Macharinic acid lactone	FKBP1A
21594250	Macharinic acid lactone	FLT3
21594250	Macharinic acid lactone	FPR1
21594250	Macharinic acid lactone	GABRA1
21594250	Macharinic acid lactone	GABRA2
21594250	Macharinic acid lactone	GABRA5
21594250	Macharinic acid lactone	GABRB3
21594250	Macharinic acid lactone	GABRB3
21594250	Macharinic acid lactone	GABRB3
21594250	Macharinic acid lactone	GCGR
21594250	Macharinic acid lactone	GRK6
21594250	Macharinic acid lactone	GSK3B
21594250	Macharinic acid lactone	GSTA1
21594250	Macharinic acid lactone	HCRTR1
21594250	Macharinic acid lactone	HCRTR2
21594250	Macharinic acid lactone	HDAC4
21594250	Macharinic acid lactone	HDAC5
21594250	Macharinic acid lactone	HDAC7
21594250	Macharinic acid lactone	HSP90AA1
21594250	Macharinic acid lactone	HTR1A
21594250	Macharinic acid lactone	HTR6
21594250	Macharinic acid lactone	HTR7
21594250	Macharinic acid lactone	IMPDH1
21594250	Macharinic acid lactone	IMPDH2
21594250	Macharinic acid lactone	JAK2
21594250	Macharinic acid lactone	JAK3
21594250	Macharinic acid lactone	KCNMA1
21594250	Macharinic acid lactone	KDM1A
21594250	Macharinic acid lactone	KDR
21594250	Macharinic acid lactone	KIF11
21594250	Macharinic acid lactone	KMT5A
21594250	Macharinic acid lactone	LDHA
21594250	Macharinic acid lactone	LNPEP
21594250	Macharinic acid lactone	MAOA
21594250	Macharinic acid lactone	MAOB
21594250	Macharinic acid lactone	MAPK8
21594250	Macharinic acid lactone	MAPKAPK2
21594250	Macharinic acid lactone	MIF
21594250	Macharinic acid lactone	MMP1
21594250	Macharinic acid lactone	MMP3
21594250	Macharinic acid lactone	MMP7
21594250	Macharinic acid lactone	MSR1

21594250	Macharinic acid lactone	MTNR1A
21594250	Macharinic acid lactone	MTNR1B
21594250	Macharinic acid lactone	MTOR
21594250	Macharinic acid lactone	MYLK
21594250	Macharinic acid lactone	NEK1
21594250	Macharinic acid lactone	NOD1
21594250	Macharinic acid lactone	NOD2
21594250	Macharinic acid lactone	NOS1
21594250	Macharinic acid lactone	NOS2
21594250	Macharinic acid lactone	NOX4
21594250	Macharinic acid lactone	NPY5R
21594250	Macharinic acid lactone	NQO1
21594250	Macharinic acid lactone	NQO2
21594250	Macharinic acid lactone	P2RX3
21594250	Macharinic acid lactone	PARP1
21594250	Macharinic acid lactone	PDE10A
21594250	Macharinic acid lactone	PDE2A
21594250	Macharinic acid lactone	PDE4A
21594250	Macharinic acid lactone	PDE4B
21594250	Macharinic acid lactone	PDE4C
21594250	Macharinic acid lactone	PDGFRA
21594250	Macharinic acid lactone	PDPK1
21594250	Macharinic acid lactone	PIK3CA
21594250	Macharinic acid lactone	PLA2G7
21594250	Macharinic acid lactone	PPIA
21594250	Macharinic acid lactone	PSEN2
21594250	Macharinic acid lactone	QPCTL
21594250	Macharinic acid lactone	RORC
21594250	Macharinic acid lactone	SIRT1
21594250	Macharinic acid lactone	TNKS
21594250	Macharinic acid lactone	TNKS2
21594250	Macharinic acid lactone	TUBB1
21594250	Macharinic acid lactone	TUBB3
21594250	Macharinic acid lactone	TYMS
21594250	Macharinic acid lactone	VCAM1
21594250	Macharinic acid lactone	XIAP
5281646	Macluraxanthone	PTPN1
69997336	Norarmepavine	ACHE
69997336	Norarmepavine	BCHE
69997336	Norarmepavine	CACNA1C
69997336	Norarmepavine	CHRM2
69997336	Norarmepavine	CHRM3
69997336	Norarmepavine	CHRNA2
69997336	Norarmepavine	DRD2

69997336	Norarmepavine	HTR3A
69997336	Norarmepavine	PDE4A
69997336	Norarmepavine	SLC18A2
54670067	Vitamin C	ALKBH2
54670067	Vitamin C	ALKBH3
54670067	Vitamin C	BBOX1
54670067	Vitamin C	CACNA1G
54670067	Vitamin C	CRTAP
54670067	Vitamin C	DBH
54670067	Vitamin C	EGLN1
54670067	Vitamin C	EGLN2
54670067	Vitamin C	EGLN3
54670067	Vitamin C	FTO
54670067	Vitamin C	GBA3
54670067	Vitamin C	JMJD6
54670067	Vitamin C	KDM5D
54670067	Vitamin C	LCT
54670067	Vitamin C	MAPK7
54670067	Vitamin C	MC3R
54670067	Vitamin C	OGFOD1
54670067	Vitamin C	OGFOD2
54670067	Vitamin C	P3H1
54670067	Vitamin C	P3H2
54670067	Vitamin C	P3H3
54670067	Vitamin C	P4HA1
54670067	Vitamin C	P4HTM
54670067	Vitamin C	PAM
54670067	Vitamin C	PGLYRP2
54670067	Vitamin C	PHYH
54670067	Vitamin C	PLOD1
54670067	Vitamin C	PLOD2
54670067	Vitamin C	PLOD3
54670067	Vitamin C	SLC23A1
54670067	Vitamin C	SLC23A2
54670067	Vitamin C	SLC27A2
54670067	Vitamin C	TMLHE
12305894	acacic acid	ACP1
12305894	acacic acid	AGTR1
12305894	acacic acid	AKR1B10
12305894	acacic acid	AKR1C4
12305894	acacic acid	ALDH2
12305894	acacic acid	ALOX5
12305894	acacic acid	AR
12305894	acacic acid	BACE1

12305894	acacic acid	CASP3
12305894	acacic acid	CD81
12305894	acacic acid	CDC25A
12305894	acacic acid	CDC25B
12305894	acacic acid	CES2
12305894	acacic acid	CTSA
12305894	acacic acid	CYP17A1
12305894	acacic acid	CYP51A1
12305894	acacic acid	ESR1
12305894	acacic acid	ESR2
12305894	acacic acid	FAAH
12305894	acacic acid	FABP1
12305894	acacic acid	FABP3
12305894	acacic acid	FABP4
12305894	acacic acid	FABP5
12305894	acacic acid	FNTA
12305894	acacic acid	G6PD
12305894	acacic acid	GPBAR1
12305894	acacic acid	HCAR2
12305894	acacic acid	HDAC1
12305894	acacic acid	HDAC8
12305894	acacic acid	HMGCR
12305894	acacic acid	HSD11B1
12305894	acacic acid	HSD11B2
12305894	acacic acid	ITGAV
12305894	acacic acid	LTB4R
12305894	acacic acid	MCL1
12305894	acacic acid	NOS2
12305894	acacic acid	NPC1L1
12305894	acacic acid	NR1H3
12305894	acacic acid	NR3C1
12305894	acacic acid	NR3C2
12305894	acacic acid	PDE4D
12305894	acacic acid	PLA2G1B
12305894	acacic acid	PLA2G4A
12305894	acacic acid	POLB
12305894	acacic acid	PPARA
12305894	acacic acid	PPARD
12305894	acacic acid	PPARG
12305894	acacic acid	PREP
12305894	acacic acid	PRKCH
12305894	acacic acid	PTGDR
12305894	acacic acid	PTGDR2
12305894	acacic acid	PTGER1

12305894	acacic acid	PTGER2
12305894	acacic acid	PTGER3
12305894	acacic acid	PTGER4
12305894	acacic acid	PTGES
12305894	acacic acid	PTGFR
12305894	acacic acid	PTGIR
12305894	acacic acid	PTGS1
12305894	acacic acid	PTGS2
12305894	acacic acid	PTPN1
12305894	acacic acid	PTPN11
12305894	acacic acid	PTPN2
12305894	acacic acid	PTPN6
12305894	acacic acid	PTPRF
12305894	acacic acid	RORC
12305894	acacic acid	SCD
12305894	acacic acid	SERPINA6
12305894	acacic acid	SHBG
12305894	acacic acid	SIGMAR1
12305894	acacic acid	SLC10A1
12305894	acacic acid	SLC10A2
12305894	acacic acid	STS
12305894	acacic acid	TERT
12305894	acacic acid	THRA
12305894	acacic acid	THRB
12305894	acacic acid	TOP1
12305894	acacic acid	TOP2A
6712546	acacic acid lactone	AADACL2
6712546	acacic acid lactone	ABL1
6712546	acacic acid lactone	AR
6712546	acacic acid lactone	AURKA
6712546	acacic acid lactone	AVPR1A
6712546	acacic acid lactone	BCL2L1
6712546	acacic acid lactone	CCR1
6712546	acacic acid lactone	CDK2
6712546	acacic acid lactone	CES2
6712546	acacic acid lactone	CSF1R
6712546	acacic acid lactone	CTRC
6712546	acacic acid lactone	CXCR3
6712546	acacic acid lactone	CYP17A1
6712546	acacic acid lactone	CYP19A1
6712546	acacic acid lactone	EPAS1
6712546	acacic acid lactone	F2
6712546	acacic acid lactone	F2RL1
6712546	acacic acid lactone	FNTA

6712546	acacic acid lactone	FNTB
6712546	acacic acid lactone	GPR18
6712546	acacic acid lactone	HMGCR
6712546	acacic acid lactone	HPGDS
6712546	acacic acid lactone	HSD11B1
6712546	acacic acid lactone	HSD11B2
6712546	acacic acid lactone	IKBKB
6712546	acacic acid lactone	ITGAL
6712546	acacic acid lactone	ITGB2
6712546	acacic acid lactone	KCNH2
6712546	acacic acid lactone	KIT
6712546	acacic acid lactone	LIMK2
6712546	acacic acid lactone	MAPK14
6712546	acacic acid lactone	MAPK8
6712546	acacic acid lactone	MDM2
6712546	acacic acid lactone	MTNR1A
6712546	acacic acid lactone	MTNR1B
6712546	acacic acid lactone	NPPB
6712546	acacic acid lactone	NR1H4
6712546	acacic acid lactone	NR3C1
6712546	acacic acid lactone	NR3C2
6712546	acacic acid lactone	PDE10A
6712546	acacic acid lactone	PGR
6712546	acacic acid lactone	PREP
6712546	acacic acid lactone	PRKCA
6712546	acacic acid lactone	PRSS1
6712546	acacic acid lactone	PTGES
6712546	acacic acid lactone	PTGS2
6712546	acacic acid lactone	PTPN1
6712546	acacic acid lactone	PTPN2
6712546	acacic acid lactone	SLC6A2
6712546	acacic acid lactone	SLC6A3
6712546	acacic acid lactone	TACR1
6712546	acacic acid lactone	TACR2
6712546	acacic acid lactone	TBXAS1
6712546	acacic acid lactone	TERT
21119850	AP1	ADORA2A
21119850	AP1	ADRB1
21119850	AP1	ADRB2
21119850	AP1	ADRB3
21119850	AP1	AKT1
21119850	AP1	CDK4
21119850	AP1	CDK6
21119850	AP1	DDOST

21119850	AP1	DRD2
21119850	AP1	DRD3
21119850	AP1	FUCA1
21119850	AP1	GAA
21119850	AP1	GBA
21119850	AP1	GPBAR1
21119850	AP1	HRH3
21119850	AP1	HTR1B
21119850	AP1	HTR2A
21119850	AP1	ITGA2B
21119850	AP1	JAK1
21119850	AP1	JAK2
21119850	AP1	JAK3
21119850	AP1	KCNH2
21119850	AP1	MAPK1
21119850	AP1	MAPKAPK2
21119850	AP1	MET
21119850	AP1	MGAM
21119850	AP1	MTOR
21119850	AP1	NR1H4
21119850	AP1	PDE4B
21119850	AP1	PDE7A
21119850	AP1	PDGFRB
21119850	AP1	PIK3CA
21119850	AP1	PIK3CB
21119850	AP1	PIK3CD
21119850	AP1	PIM1
21119850	AP1	PIM2
21119850	AP1	PIM3
21119850	AP1	PRKDC
21119850	AP1	S1PR1
21119850	AP1	SCN9A
21119850	AP1	SI
21119850	AP1	SLC16A1
21119850	AP1	SPHK1
21119850	AP1	SPHK2
21119850	AP1	SYK
21119850	AP1	TOP1
21119850	AP1	TYK2
444664	AP3	ADORA3
444664	AP3	GCLC
444664	AP3	GRIA1
444664	AP3	GRIA4
444664	AP3	GRIK1

444664	AP3	GRIK2
444664	AP3	GRIK3
444664	AP3	GRIK5
444664	AP3	GRM2
444664	AP3	GRM3
444664	AP3	GRM6
444664	AP3	GRM8
444664	AP3	LAP3
444664	AP3	SLC1A1
73309	Echinocystic acid	ACP1
73309	Echinocystic acid	AGTR1
73309	Echinocystic acid	AKR1B10
73309	Echinocystic acid	ALOX5
73309	Echinocystic acid	ALOX5AP
73309	Echinocystic acid	AR
73309	Echinocystic acid	CD81
73309	Echinocystic acid	CDC25A
73309	Echinocystic acid	CDC25B
73309	Echinocystic acid	CES2
73309	Echinocystic acid	CYP17A1
73309	Echinocystic acid	CYP19A1
73309	Echinocystic acid	CYP51A1
73309	Echinocystic acid	EDNRA
73309	Echinocystic acid	ESR1
73309	Echinocystic acid	ESR2
73309	Echinocystic acid	FAAH
73309	Echinocystic acid	FABP1
73309	Echinocystic acid	FABP3
73309	Echinocystic acid	FABP4
73309	Echinocystic acid	FABP5
73309	Echinocystic acid	FFAR1
73309	Echinocystic acid	FNTA
73309	Echinocystic acid	G6PD
73309	Echinocystic acid	GPBAR1
73309	Echinocystic acid	GRIK1
73309	Echinocystic acid	GRIK2
73309	Echinocystic acid	HMGCR
73309	Echinocystic acid	HSD11B1
73309	Echinocystic acid	HSD11B2
73309	Echinocystic acid	LTB4R
73309	Echinocystic acid	MDM2
73309	Echinocystic acid	MMP1
73309	Echinocystic acid	MMP2
73309	Echinocystic acid	MMP3

73309	Echinocystic acid	NOS2
73309	Echinocystic acid	NPC1L1
73309	Echinocystic acid	NR1H3
73309	Echinocystic acid	NR1H4
73309	Echinocystic acid	NR3C1
73309	Echinocystic acid	NR3C2
73309	Echinocystic acid	PDE4D
73309	Echinocystic acid	PGR
73309	Echinocystic acid	PLA2G1B
73309	Echinocystic acid	POLA1
73309	Echinocystic acid	POLB
73309	Echinocystic acid	PPARA
73309	Echinocystic acid	PPARD
73309	Echinocystic acid	PPARG
73309	Echinocystic acid	PREP
73309	Echinocystic acid	PRKCH
73309	Echinocystic acid	PTGDR
73309	Echinocystic acid	PTGDR2
73309	Echinocystic acid	PTGER1
73309	Echinocystic acid	PTGER2
73309	Echinocystic acid	PTGER4
73309	Echinocystic acid	PTGES
73309	Echinocystic acid	PTGIR
73309	Echinocystic acid	PTGS1
73309	Echinocystic acid	PTGS2
73309	Echinocystic acid	PTPN1
73309	Echinocystic acid	PTPN11
73309	Echinocystic acid	PTPN2
73309	Echinocystic acid	PTPN6
73309	Echinocystic acid	PTPRF
73309	Echinocystic acid	RORC
73309	Echinocystic acid	SCD
73309	Echinocystic acid	SERPINA6
73309	Echinocystic acid	SHBG
73309	Echinocystic acid	SIGMAR1
73309	Echinocystic acid	SLC10A1
73309	Echinocystic acid	SLC10A2
73309	Echinocystic acid	SRD5A2
73309	Echinocystic acid	TERT
73309	Echinocystic acid	THRA
73309	Echinocystic acid	THRB
73309	Echinocystic acid	TLR9
73309	Echinocystic acid	TOP1
73309	Echinocystic acid	TOP2A

73309	Echinocystic acid	TRPM8
725031	2-[(4-Methylphenyl)thio]nicotinic acid	ABCC1
725031	2-[(4-Methylphenyl)thio]nicotinic acid	ACE
725031	2-[(4-Methylphenyl)thio]nicotinic acid	ACE2
725031	2-[(4-Methylphenyl)thio]nicotinic acid	AKR1B1
725031	2-[(4-Methylphenyl)thio]nicotinic acid	AKR1B10
725031	2-[(4-Methylphenyl)thio]nicotinic acid	AKR1C1
725031	2-[(4-Methylphenyl)thio]nicotinic acid	AKR1C2
725031	2-[(4-Methylphenyl)thio]nicotinic acid	AKR1C3
725031	2-[(4-Methylphenyl)thio]nicotinic acid	ALOX5
725031	2-[(4-Methylphenyl)thio]nicotinic acid	APEX1
725031	2-[(4-Methylphenyl)thio]nicotinic acid	CA1
725031	2-[(4-Methylphenyl)thio]nicotinic acid	CA12
725031	2-[(4-Methylphenyl)thio]nicotinic acid	CA9
725031	2-[(4-Methylphenyl)thio]nicotinic acid	CASP1
725031	2-[(4-Methylphenyl)thio]nicotinic acid	CCNC
725031	2-[(4-Methylphenyl)thio]nicotinic acid	CCR2
725031	2-[(4-Methylphenyl)thio]nicotinic acid	CDK9
725031	2-[(4-Methylphenyl)thio]nicotinic acid	CES1
725031	2-[(4-Methylphenyl)thio]nicotinic acid	CES2
725031	2-[(4-Methylphenyl)thio]nicotinic acid	CHRM1
725031	2-[(4-Methylphenyl)thio]nicotinic acid	CHRNA4

725031	2-[(4-Methylphenyl)thio]nicotinic acid	CISD1
725031	2-[(4-Methylphenyl)thio]nicotinic acid	CLK1
725031	2-[(4-Methylphenyl)thio]nicotinic acid	CSNK2A1
725031	2-[(4-Methylphenyl)thio]nicotinic acid	CSNK2A2
725031	2-[(4-Methylphenyl)thio]nicotinic acid	CXCL8
725031	2-[(4-Methylphenyl)thio]nicotinic acid	DAO
725031	2-[(4-Methylphenyl)thio]nicotinic acid	DBF4
725031	2-[(4-Methylphenyl)thio]nicotinic acid	DHODH
725031	2-[(4-Methylphenyl)thio]nicotinic acid	DYRK1A
725031	2-[(4-Methylphenyl)thio]nicotinic acid	ECE1
725031	2-[(4-Methylphenyl)thio]nicotinic acid	EDNRA
725031	2-[(4-Methylphenyl)thio]nicotinic acid	EP300
725031	2-[(4-Methylphenyl)thio]nicotinic acid	EPRS
725031	2-[(4-Methylphenyl)thio]nicotinic acid	ERCC5
725031	2-[(4-Methylphenyl)thio]nicotinic acid	ESR2
725031	2-[(4-Methylphenyl)thio]nicotinic acid	FABP1
725031	2-[(4-Methylphenyl)thio]nicotinic acid	FABP2
725031	2-[(4-Methylphenyl)thio]nicotinic acid	FABP3
725031	2-[(4-Methylphenyl)thio]nicotinic acid	FABP4
725031	2-[(4-Methylphenyl)thio]nicotinic acid	FABP5
725031	2-[(4-Methylphenyl)thio]nicotinic acid	FEN1
725031	2-[(4-Methylphenyl)thio]nicotinic acid	FNTA

725031	2-[(4-Methylphenyl)thio]nicotinic acid	FOLH1
725031	2-[(4-Methylphenyl)thio]nicotinic acid	GABRA2
725031	2-[(4-Methylphenyl)thio]nicotinic acid	GSTA1
725031	2-[(4-Methylphenyl)thio]nicotinic acid	GSTP1
725031	2-[(4-Methylphenyl)thio]nicotinic acid	HAO1
725031	2-[(4-Methylphenyl)thio]nicotinic acid	HSP90AA1
725031	2-[(4-Methylphenyl)thio]nicotinic acid	HSPA1A
725031	2-[(4-Methylphenyl)thio]nicotinic acid	ICAM1
725031	2-[(4-Methylphenyl)thio]nicotinic acid	ITGAL
725031	2-[(4-Methylphenyl)thio]nicotinic acid	KDM3A
725031	2-[(4-Methylphenyl)thio]nicotinic acid	KDM4A
725031	2-[(4-Methylphenyl)thio]nicotinic acid	KDM4B
725031	2-[(4-Methylphenyl)thio]nicotinic acid	KDM4C
725031	2-[(4-Methylphenyl)thio]nicotinic acid	KDM4D
725031	2-[(4-Methylphenyl)thio]nicotinic acid	KDM4E
725031	2-[(4-Methylphenyl)thio]nicotinic acid	KDM5A
725031	2-[(4-Methylphenyl)thio]nicotinic acid	KDM5B
725031	2-[(4-Methylphenyl)thio]nicotinic acid	KDM5C
725031	2-[(4-Methylphenyl)thio]nicotinic acid	KDM6B
725031	2-[(4-Methylphenyl)thio]nicotinic acid	KMO
725031	2-[(4-Methylphenyl)thio]nicotinic acid	MAOA
725031	2-[(4-Methylphenyl)thio]nicotinic acid	MAOB

725031	2-[(4-Methylphenyl)thio]nicotinic acid	MAP3K8
725031	2-[(4-Methylphenyl)thio]nicotinic acid	MAPK1
725031	2-[(4-Methylphenyl)thio]nicotinic acid	MCL1
725031	2-[(4-Methylphenyl)thio]nicotinic acid	METAP2
725031	2-[(4-Methylphenyl)thio]nicotinic acid	MKNK1
725031	2-[(4-Methylphenyl)thio]nicotinic acid	MKNK2
725031	2-[(4-Methylphenyl)thio]nicotinic acid	MME
725031	2-[(4-Methylphenyl)thio]nicotinic acid	MMP12
725031	2-[(4-Methylphenyl)thio]nicotinic acid	MMP13
725031	2-[(4-Methylphenyl)thio]nicotinic acid	MMP3
725031	2-[(4-Methylphenyl)thio]nicotinic acid	MMP8
725031	2-[(4-Methylphenyl)thio]nicotinic acid	NOTUM
725031	2-[(4-Methylphenyl)thio]nicotinic acid	P4HTM
725031	2-[(4-Methylphenyl)thio]nicotinic acid	PDE10A
725031	2-[(4-Methylphenyl)thio]nicotinic acid	PIK3CA
725031	2-[(4-Methylphenyl)thio]nicotinic acid	PLEC
725031	2-[(4-Methylphenyl)thio]nicotinic acid	PPARA
725031	2-[(4-Methylphenyl)thio]nicotinic acid	PPARG
725031	2-[(4-Methylphenyl)thio]nicotinic acid	PPP1CA
725031	2-[(4-Methylphenyl)thio]nicotinic acid	PTGDR2
725031	2-[(4-Methylphenyl)thio]nicotinic acid	PTGER1
725031	2-[(4-Methylphenyl)thio]nicotinic acid	PTGER4

725031	2-[(4-Methylphenyl)thio]nicotinic acid	PTGES
725031	2-[(4-Methylphenyl)thio]nicotinic acid	PTGS1
725031	2-[(4-Methylphenyl)thio]nicotinic acid	PTPRG
725031	2-[(4-Methylphenyl)thio]nicotinic acid	PYGL
725031	2-[(4-Methylphenyl)thio]nicotinic acid	PYGM
725031	2-[(4-Methylphenyl)thio]nicotinic acid	RPA1
725031	2-[(4-Methylphenyl)thio]nicotinic acid	SELE
725031	2-[(4-Methylphenyl)thio]nicotinic acid	SLC22A12
725031	2-[(4-Methylphenyl)thio]nicotinic acid	SLCO1B1
725031	2-[(4-Methylphenyl)thio]nicotinic acid	SORT1
725031	2-[(4-Methylphenyl)thio]nicotinic acid	SRD5A2
725031	2-[(4-Methylphenyl)thio]nicotinic acid	TBXAS1
725031	2-[(4-Methylphenyl)thio]nicotinic acid	TTR
11453158	Keratinocyte Differentiation Inducer	ALDH2
11453158	Keratinocyte Differentiation Inducer	ALOX5AP
11453158	Keratinocyte Differentiation Inducer	APP
11453158	Keratinocyte Differentiation Inducer	ATR
11453158	Keratinocyte Differentiation Inducer	BACE1
11453158	Keratinocyte Differentiation Inducer	BAD
11453158	Keratinocyte Differentiation Inducer	BDKRB1
11453158	Keratinocyte Differentiation Inducer	BLM
11453158	Keratinocyte Differentiation Inducer	C1R

11453158	Keratinocyte Differentiation Inducer	CA1
11453158	Keratinocyte Differentiation Inducer	CA2
11453158	Keratinocyte Differentiation Inducer	CASP3
11453158	Keratinocyte Differentiation Inducer	CDC7
11453158	Keratinocyte Differentiation Inducer	CDK5R1
11453158	Keratinocyte Differentiation Inducer	CDKL1
11453158	Keratinocyte Differentiation Inducer	CFD
11453158	Keratinocyte Differentiation Inducer	CFTR
11453158	Keratinocyte Differentiation Inducer	CLK3
11453158	Keratinocyte Differentiation Inducer	CNR1
11453158	Keratinocyte Differentiation Inducer	CNR2
11453158	Keratinocyte Differentiation Inducer	CXCR2
11453158	Keratinocyte Differentiation Inducer	CYP19A1
11453158	Keratinocyte Differentiation Inducer	CYP2C9
11453158	Keratinocyte Differentiation Inducer	CYP3A4
11453158	Keratinocyte Differentiation Inducer	DHODH
11453158	Keratinocyte Differentiation Inducer	DUT
11453158	Keratinocyte Differentiation Inducer	DYRK2
11453158	Keratinocyte Differentiation Inducer	ELANE
11453158	Keratinocyte Differentiation Inducer	F2
11453158	Keratinocyte Differentiation Inducer	F3
11453158	Keratinocyte Differentiation Inducer	FGFR2

11453158	Keratinocyte Differentiation Inducer	FKBP1A
11453158	Keratinocyte Differentiation Inducer	FPR2
11453158	Keratinocyte Differentiation Inducer	GCK
11453158	Keratinocyte Differentiation Inducer	GRIN1
11453158	Keratinocyte Differentiation Inducer	GRIN2B
11453158	Keratinocyte Differentiation Inducer	GRK2
11453158	Keratinocyte Differentiation Inducer	GRK3
11453158	Keratinocyte Differentiation Inducer	GRK5
11453158	Keratinocyte Differentiation Inducer	GRM1
11453158	Keratinocyte Differentiation Inducer	HSD11B1
11453158	Keratinocyte Differentiation Inducer	HSD17B1
11453158	Keratinocyte Differentiation Inducer	HSD17B2
11453158	Keratinocyte Differentiation Inducer	IDH1
11453158	Keratinocyte Differentiation Inducer	JAK2
11453158	Keratinocyte Differentiation Inducer	KCNK3
11453158	Keratinocyte Differentiation Inducer	KCNK9
11453158	Keratinocyte Differentiation Inducer	KDR
11453158	Keratinocyte Differentiation Inducer	KHK
11453158	Keratinocyte Differentiation Inducer	LCK
11453158	Keratinocyte Differentiation Inducer	LIPG
11453158	Keratinocyte Differentiation Inducer	MAPK1
11453158	Keratinocyte Differentiation Inducer	MERTK

11453158	Keratinocyte Differentiation Inducer	METAP2
11453158	Keratinocyte Differentiation Inducer	MGLL
11453158	Keratinocyte Differentiation Inducer	MKNK2
11453158	Keratinocyte Differentiation Inducer	MTOR
11453158	Keratinocyte Differentiation Inducer	NAMPT
11453158	Keratinocyte Differentiation Inducer	NOS1
11453158	Keratinocyte Differentiation Inducer	NOS2
11453158	Keratinocyte Differentiation Inducer	PARP1
11453158	Keratinocyte Differentiation Inducer	PDE3B
11453158	Keratinocyte Differentiation Inducer	PDE4A
11453158	Keratinocyte Differentiation Inducer	PDE4C
11453158	Keratinocyte Differentiation Inducer	PDE4D
11453158	Keratinocyte Differentiation Inducer	PDE9A
11453158	Keratinocyte Differentiation Inducer	PDPK1
11453158	Keratinocyte Differentiation Inducer	PGK1
11453158	Keratinocyte Differentiation Inducer	PIK3CD
11453158	Keratinocyte Differentiation Inducer	PIK3R1
11453158	Keratinocyte Differentiation Inducer	PIM2
11453158	Keratinocyte Differentiation Inducer	PKM
11453158	Keratinocyte Differentiation Inducer	PLK3
11453158	Keratinocyte Differentiation Inducer	PORCN
11453158	Keratinocyte Differentiation Inducer	PPIA

11453158	Keratinocyte Differentiation Inducer	PRKACA
11453158	Keratinocyte Differentiation Inducer	PRKCA
11453158	Keratinocyte Differentiation Inducer	PRKCB
11453158	Keratinocyte Differentiation Inducer	PRKCQ
11453158	Keratinocyte Differentiation Inducer	PRKDC
11453158	Keratinocyte Differentiation Inducer	PTGS2
11453158	Keratinocyte Differentiation Inducer	PTK2
11453158	Keratinocyte Differentiation Inducer	QPCT
11453158	Keratinocyte Differentiation Inducer	RGS4
11453158	Keratinocyte Differentiation Inducer	ROCK1
11453158	Keratinocyte Differentiation Inducer	ROCK2
11453158	Keratinocyte Differentiation Inducer	RORC
11453158	Keratinocyte Differentiation Inducer	S1PR1
11453158	Keratinocyte Differentiation Inducer	S1PR3
11453158	Keratinocyte Differentiation Inducer	SCN10A
11453158	Keratinocyte Differentiation Inducer	SCN2A
11453158	Keratinocyte Differentiation Inducer	SFRP1
11453158	Keratinocyte Differentiation Inducer	SRC
11453158	Keratinocyte Differentiation Inducer	STAT3
11453158	Keratinocyte Differentiation Inducer	STK17A
11453158	Keratinocyte Differentiation Inducer	STK17B
11453158	Keratinocyte Differentiation Inducer	TBXAS1

11453158	Keratinocyte Differentiation Inducer	TGFBR1
11453158	Keratinocyte Differentiation Inducer	TNIK
11453158	Keratinocyte Differentiation Inducer	TRPV1
11453158	Keratinocyte Differentiation Inducer	TSPO
11453158	Keratinocyte Differentiation Inducer	TUBB1
131990	10-(4-Methylpiperazin-1-yl)pyrido(4,3-b)(1,4)benzothiazepine	ACHE
131990	10-(4-Methylpiperazin-1-yl)pyrido(4,3-b)(1,4)benzothiazepine	ADRA1A
131990	10-(4-Methylpiperazin-1-yl)pyrido(4,3-b)(1,4)benzothiazepine	ADRA1B
131990	10-(4-Methylpiperazin-1-yl)pyrido(4,3-b)(1,4)benzothiazepine	ADRA1D
131990	10-(4-Methylpiperazin-1-yl)pyrido(4,3-b)(1,4)benzothiazepine	ADRA2A
131990	10-(4-Methylpiperazin-1-yl)pyrido(4,3-b)(1,4)benzothiazepine	ADRA2B
131990	10-(4-Methylpiperazin-1-yl)pyrido(4,3-b)(1,4)benzothiazepine	ADRA2C
131990	10-(4-Methylpiperazin-1-yl)pyrido(4,3-b)(1,4)benzothiazepine	ALOX5
131990	10-(4-Methylpiperazin-1-yl)pyrido(4,3-b)(1,4)benzothiazepine	BACE1
131990	10-(4-Methylpiperazin-1-yl)pyrido(4,3-b)(1,4)benzothiazepine	BCHE
131990	10-(4-Methylpiperazin-1-yl)pyrido(4,3-b)(1,4)benzothiazepine	CHRM4

131990	10-(4-Methylpiperazin-1-yl)pyrido(4,3-b)(1,4)benzothiazepine	DRD1
131990	10-(4-Methylpiperazin-1-yl)pyrido(4,3-b)(1,4)benzothiazepine	DRD2
131990	10-(4-Methylpiperazin-1-yl)pyrido(4,3-b)(1,4)benzothiazepine	DRD3
131990	10-(4-Methylpiperazin-1-yl)pyrido(4,3-b)(1,4)benzothiazepine	DRD4
131990	10-(4-Methylpiperazin-1-yl)pyrido(4,3-b)(1,4)benzothiazepine	H1F0
131990	10-(4-Methylpiperazin-1-yl)pyrido(4,3-b)(1,4)benzothiazepine	HDAC1
131990	10-(4-Methylpiperazin-1-yl)pyrido(4,3-b)(1,4)benzothiazepine	HDAC7
131990	10-(4-Methylpiperazin-1-yl)pyrido(4,3-b)(1,4)benzothiazepine	HRH1
131990	10-(4-Methylpiperazin-1-yl)pyrido(4,3-b)(1,4)benzothiazepine	HRH4
131990	10-(4-Methylpiperazin-1-yl)pyrido(4,3-b)(1,4)benzothiazepine	HSD11B1
131990	10-(4-Methylpiperazin-1-yl)pyrido(4,3-b)(1,4)benzothiazepine	HTR1A
131990	10-(4-Methylpiperazin-1-yl)pyrido(4,3-b)(1,4)benzothiazepine	HTR2A
131990	10-(4-Methylpiperazin-1-yl)pyrido(4,3-b)(1,4)benzothiazepine	HTR2B
131990	10-(4-Methylpiperazin-1-yl)pyrido(4,3-b)(1,4)benzothiazepine	HTR2C

131990	10-(4-Methylpiperazin-1-yl)pyrido(4,3-b)(1,4)benzothiazepine	HTR6
131990	10-(4-Methylpiperazin-1-yl)pyrido(4,3-b)(1,4)benzothiazepine	HTR7
131990	10-(4-Methylpiperazin-1-yl)pyrido(4,3-b)(1,4)benzothiazepine	JAK1
131990	10-(4-Methylpiperazin-1-yl)pyrido(4,3-b)(1,4)benzothiazepine	JAK2
131990	10-(4-Methylpiperazin-1-yl)pyrido(4,3-b)(1,4)benzothiazepine	JAK3
131990	10-(4-Methylpiperazin-1-yl)pyrido(4,3-b)(1,4)benzothiazepine	MAP4K3
131990	10-(4-Methylpiperazin-1-yl)pyrido(4,3-b)(1,4)benzothiazepine	MAP4K5
131990	10-(4-Methylpiperazin-1-yl)pyrido(4,3-b)(1,4)benzothiazepine	NEK1
131990	10-(4-Methylpiperazin-1-yl)pyrido(4,3-b)(1,4)benzothiazepine	NPY5R
131990	10-(4-Methylpiperazin-1-yl)pyrido(4,3-b)(1,4)benzothiazepine	OPRK1
131990	10-(4-Methylpiperazin-1-yl)pyrido(4,3-b)(1,4)benzothiazepine	PABPC1
131990	10-(4-Methylpiperazin-1-yl)pyrido(4,3-b)(1,4)benzothiazepine	PARP1
131990	10-(4-Methylpiperazin-1-yl)pyrido(4,3-b)(1,4)benzothiazepine	PRKCZ
131990	10-(4-Methylpiperazin-1-yl)pyrido(4,3-b)(1,4)benzothiazepine	PTPN1

131990	10-(4-Methylpiperazin-1-yl)pyrido(4,3-b)(1,4)benzothiazepine	SIGMAR1
131990	10-(4-Methylpiperazin-1-yl)pyrido(4,3-b)(1,4)benzothiazepine	SRD5A2
33032	L-glutamic acid	ADORA3
33032	L-glutamic acid	BBOX1
33032	L-glutamic acid	GABRA1
33032	L-glutamic acid	GABRA2
33032	L-glutamic acid	GABRA3
33032	L-glutamic acid	GABRR1
33032	L-glutamic acid	GRIA1
33032	L-glutamic acid	GRIA2
33032	L-glutamic acid	GRIA4
33032	L-glutamic acid	GRIK1
33032	L-glutamic acid	GRIK2
33032	L-glutamic acid	GRIK3
33032	L-glutamic acid	GRIK5
33032	L-glutamic acid	GRM1
33032	L-glutamic acid	GRM2
33032	L-glutamic acid	GRM3
33032	L-glutamic acid	GRM4
33032	L-glutamic acid	GRM5
33032	L-glutamic acid	GRM6
33032	L-glutamic acid	GRM7
33032	L-glutamic acid	GRM8
33032	L-glutamic acid	SLC1A1
33032	L-glutamic acid	SLC1A2
33032	L-glutamic acid	SLC22A6
33032	L-glutamic acid	SLC6A1
33032	L-glutamic acid	SLC6A11
33032	L-glutamic acid	SLC6A12
33032	L-glutamic acid	SLC6A13
5960	l-aspartic acid	EGLN1
5962	lysine	AR
5962	lysine	CACNA2D1
5962	lysine	CPB2
5962	lysine	GRIA1
5962	lysine	GRIA2
5962	lysine	GRIA4
5962	lysine	GRIK1
5962	lysine	GRIK2
5962	lysine	GRIK3

5962	lysine	GRIK5
5962	lysine	GRM1
5962	lysine	GRM2
5962	lysine	GRM3
5962	lysine	GRM4
5962	lysine	GRM5
5962	lysine	GRM6
5962	lysine	GRM7
5962	lysine	GRM8
5962	lysine	LTA4H
5962	lysine	ODC1
5962	lysine	PEPD
5962	lysine	PLG
5962	lysine	SHBG
5962	lysine	SLC1A1
5962	lysine	SLC22A6
205	DL-Threonine	ABAT
205	DL-Threonine	GABBR2
14729078	sanjoinenine	ACACB
14729078	sanjoinenine	ADRA1A
14729078	sanjoinenine	ADRA1B
14729078	sanjoinenine	ADRA1D
14729078	sanjoinenine	ADRA2A
14729078	sanjoinenine	AGTR1
14729078	sanjoinenine	AVPR1A
14729078	sanjoinenine	AVPR2
14729078	sanjoinenine	BCL2
14729078	sanjoinenine	BDKRB1
14729078	sanjoinenine	BDKRB2
14729078	sanjoinenine	BRD2
14729078	sanjoinenine	BRD4
14729078	sanjoinenine	C5AR1
14729078	sanjoinenine	CALCRL
14729078	sanjoinenine	CAPN1
14729078	sanjoinenine	CDK2
14729078	sanjoinenine	CFD
14729078	sanjoinenine	CHRM1
14729078	sanjoinenine	CHRM3
14729078	sanjoinenine	CPT1A
14729078	sanjoinenine	CPT2
14729078	sanjoinenine	CTRC
14729078	sanjoinenine	CTSV
14729078	sanjoinenine	CYP19A1
14729078	sanjoinenine	DRD1

14729078	sanjoinenine	EPHX2
14729078	sanjoinenine	EZH2
14729078	sanjoinenine	F10
14729078	sanjoinenine	F2RL1
14729078	sanjoinenine	F9
14729078	sanjoinenine	FLT3
14729078	sanjoinenine	FPR1
14729078	sanjoinenine	FPR2
14729078	sanjoinenine	GALR1
14729078	sanjoinenine	GALR2
14729078	sanjoinenine	GRK2
14729078	sanjoinenine	HCRTR1
14729078	sanjoinenine	HCRTR2
14729078	sanjoinenine	HLA-DRB1
14729078	sanjoinenine	HRH1
14729078	sanjoinenine	HTR1A
14729078	sanjoinenine	HTR2B
14729078	sanjoinenine	HTR6
14729078	sanjoinenine	HTR7
14729078	sanjoinenine	IDH1
14729078	sanjoinenine	JAK2
14729078	sanjoinenine	KCNA5
14729078	sanjoinenine	KCNJ11
14729078	sanjoinenine	KCNJ5
14729078	sanjoinenine	KCNJ6
14729078	sanjoinenine	KIF11
14729078	sanjoinenine	LGMN
14729078	sanjoinenine	MAPK11
14729078	sanjoinenine	MAPK14
14729078	sanjoinenine	MAPK8
14729078	sanjoinenine	MMP8
14729078	sanjoinenine	NCOR1
14729078	sanjoinenine	NOS2
14729078	sanjoinenine	NPY1R
14729078	sanjoinenine	NPY5R
14729078	sanjoinenine	NR1H4
14729078	sanjoinenine	OPRD1
14729078	sanjoinenine	OPRK1
14729078	sanjoinenine	OPRM1
14729078	sanjoinenine	OXTR
14729078	sanjoinenine	P2RX7
14729078	sanjoinenine	PDE10A
14729078	sanjoinenine	PDE11A
14729078	sanjoinenine	PDE2A

14729078	sanjoinenine	PDE5A
14729078	sanjoinenine	PDE9A
14729078	sanjoinenine	PDPK1
14729078	sanjoinenine	PGGT1B
14729078	sanjoinenine	PLA2G7
14729078	sanjoinenine	PLAT
14729078	sanjoinenine	PPIA
14729078	sanjoinenine	PRCP
14729078	sanjoinenine	PRLHR
14729078	sanjoinenine	PSMB5
14729078	sanjoinenine	PTGER1
14729078	sanjoinenine	S1PR3
14729078	sanjoinenine	SCARB1
14729078	sanjoinenine	SCN9A
14729078	sanjoinenine	SFRP1
14729078	sanjoinenine	SHH
14729078	sanjoinenine	SLC10A2
14729078	sanjoinenine	SLC6A4
14729078	sanjoinenine	SLC6A9
14729078	sanjoinenine	SMO
14729078	sanjoinenine	SOAT1
14729078	sanjoinenine	SYK
14729078	sanjoinenine	TACR1
14729078	sanjoinenine	TACR3
14729078	sanjoinenine	TBK1
14729078	sanjoinenine	TBXA2R
14729078	sanjoinenine	TERT
14729078	sanjoinenine	TGFBR1
14729078	sanjoinenine	TRPV1
14729078	sanjoinenine	TSPO
102063083	zizyphusine	ABCB1
102063083	zizyphusine	ADAM17
102063083	zizyphusine	ADORA1
102063083	zizyphusine	ADORA2A
102063083	zizyphusine	ADORA3
102063083	zizyphusine	ADRA1A
102063083	zizyphusine	ADRA1B
102063083	zizyphusine	ADRA1D
102063083	zizyphusine	ADRA2A
102063083	zizyphusine	ADRA2B
102063083	zizyphusine	ADRA2C
102063083	zizyphusine	ADRB1
102063083	zizyphusine	ADRB2
102063083	zizyphusine	ALK

102063083	zizyphusine	ALOX12
102063083	zizyphusine	ALOX15
102063083	zizyphusine	ANPEP
102063083	zizyphusine	AURKA
102063083	zizyphusine	AURKB
102063083	zizyphusine	CDC25B
102063083	zizyphusine	CDK2
102063083	zizyphusine	CDK5R1
102063083	zizyphusine	CHRNA4
102063083	zizyphusine	CLK1
102063083	zizyphusine	CLK3
102063083	zizyphusine	CMA1
102063083	zizyphusine	CSF1R
102063083	zizyphusine	CTSB
102063083	zizyphusine	CXCR2
102063083	zizyphusine	DBF4
102063083	zizyphusine	DRD1
102063083	zizyphusine	DRD2
102063083	zizyphusine	DRD3
102063083	zizyphusine	DRD4
102063083	zizyphusine	DRD5
102063083	zizyphusine	DUSP3
102063083	zizyphusine	DYRK1A
102063083	zizyphusine	DYRK2
102063083	zizyphusine	ERN1
102063083	zizyphusine	F10
102063083	zizyphusine	FLT3
102063083	zizyphusine	FLT4
102063083	zizyphusine	GCGR
102063083	zizyphusine	GSK3A
102063083	zizyphusine	GSK3B
102063083	zizyphusine	GUSB
102063083	zizyphusine	HRH2
102063083	zizyphusine	HSD17B1
102063083	zizyphusine	HSD17B2
102063083	zizyphusine	HSP90AB1
102063083	zizyphusine	HTR1A
102063083	zizyphusine	HTR1B
102063083	zizyphusine	HTR1D
102063083	zizyphusine	HTR2A
102063083	zizyphusine	HTR2B
102063083	zizyphusine	HTR2C
102063083	zizyphusine	HTR5A
102063083	zizyphusine	HTR6

102063083	zizyphusine	HTR7
102063083	zizyphusine	KCNH2
102063083	zizyphusine	KDR
102063083	zizyphusine	KIT
102063083	zizyphusine	LRRK2
102063083	zizyphusine	MAP2K2
102063083	zizyphusine	MET
102063083	zizyphusine	MIF
102063083	zizyphusine	MMP1
102063083	zizyphusine	MMP12
102063083	zizyphusine	MMP13
102063083	zizyphusine	MMP2
102063083	zizyphusine	MMP3
102063083	zizyphusine	MMP7
102063083	zizyphusine	MMP8
102063083	zizyphusine	MMP9
102063083	zizyphusine	NOS2
102063083	zizyphusine	OPRM1
102063083	zizyphusine	PDE5A
102063083	zizyphusine	PDF
102063083	zizyphusine	PDGFRA
102063083	zizyphusine	PDGFRB
102063083	zizyphusine	PIK3CA
102063083	zizyphusine	PIK3CA
102063083	zizyphusine	PIM1
102063083	zizyphusine	PLA2G7
102063083	zizyphusine	PRKCA
102063083	zizyphusine	PRKCB
102063083	zizyphusine	PSEN2
102063083	zizyphusine	PTPRCAP
102063083	zizyphusine	ROCK2
102063083	zizyphusine	RPS6KB1
102063083	zizyphusine	SIGMAR1
102063083	zizyphusine	SLC6A3
102063083	zizyphusine	SLC6A4
102063083	zizyphusine	SYK
102063083	zizyphusine	TGFBR1
102063083	zizyphusine	TH
102063083	zizyphusine	TNKS
102063083	zizyphusine	TNKS2
102063083	zizyphusine	TRPV1
102063083	zizyphusine	TSPO
12305768	Alphitolic Acid	ACP1
12305768	Alphitolic Acid	AGTR1

12305768	Alphitolic Acid	AKR1B10
12305768	Alphitolic Acid	ALOX5AP
12305768	Alphitolic Acid	AMPD2
12305768	Alphitolic Acid	AR
12305768	Alphitolic Acid	CDC25A
12305768	Alphitolic Acid	CDC25B
12305768	Alphitolic Acid	CDC25C
12305768	Alphitolic Acid	CES2
12305768	Alphitolic Acid	CYP17A1
12305768	Alphitolic Acid	CYP19A1
12305768	Alphitolic Acid	CYP51A1
12305768	Alphitolic Acid	FABP1
12305768	Alphitolic Acid	FNTA
12305768	Alphitolic Acid	G6PD
12305768	Alphitolic Acid	GABBR1
12305768	Alphitolic Acid	GPBAR1
12305768	Alphitolic Acid	HMGCR
12305768	Alphitolic Acid	HSD11B1
12305768	Alphitolic Acid	HSD17B3
12305768	Alphitolic Acid	ITGB1
12305768	Alphitolic Acid	MMP1
12305768	Alphitolic Acid	MMP2
12305768	Alphitolic Acid	MMP3
12305768	Alphitolic Acid	NPC1L1
12305768	Alphitolic Acid	NR1H4
12305768	Alphitolic Acid	PDE4D
12305768	Alphitolic Acid	PLA2G1B
12305768	Alphitolic Acid	POLB
12305768	Alphitolic Acid	PPARA
12305768	Alphitolic Acid	PPARD
12305768	Alphitolic Acid	PPARG
12305768	Alphitolic Acid	PTGER2
12305768	Alphitolic Acid	PTGES
12305768	Alphitolic Acid	PTGS2
12305768	Alphitolic Acid	PTPN1
12305768	Alphitolic Acid	PTPN11
12305768	Alphitolic Acid	PTPN2
12305768	Alphitolic Acid	PTPRF
12305768	Alphitolic Acid	RORC
12305768	Alphitolic Acid	SAE1
12305768	Alphitolic Acid	SCD
12305768	Alphitolic Acid	SIGMAR1
12305768	Alphitolic Acid	TERT
12305768	Alphitolic Acid	TOP1

12305768	Alphitolic Acid	TOP2A
12305768	Alphitolic Acid	UGT2B7
12305768	Alphitolic Acid	VDR
197017	N-Methylasimilobine	CYP2D6
197017	N-Methylasimilobine	ABCB1
197017	N-Methylasimilobine	ADCY5
197017	N-Methylasimilobine	ADORA3
197017	N-Methylasimilobine	ADRA1A
197017	N-Methylasimilobine	ADRA1B
197017	N-Methylasimilobine	ADRA1D
197017	N-Methylasimilobine	ADRA2A
197017	N-Methylasimilobine	ADRA2B
197017	N-Methylasimilobine	ADRA2C
197017	N-Methylasimilobine	ADRB1
197017	N-Methylasimilobine	ADRB2
197017	N-Methylasimilobine	ADRB3
197017	N-Methylasimilobine	ALOX12
197017	N-Methylasimilobine	ALOX15
197017	N-Methylasimilobine	AOC3
197017	N-Methylasimilobine	BIRC2
197017	N-Methylasimilobine	CCL2
197017	N-Methylasimilobine	CDK9
197017	N-Methylasimilobine	CHRM1
197017	N-Methylasimilobine	CHRM2
197017	N-Methylasimilobine	CHRM3
197017	N-Methylasimilobine	CHRM4
197017	N-Methylasimilobine	CHRNA3
197017	N-Methylasimilobine	CHRNA4
197017	N-Methylasimilobine	CHRNA4
197017	N-Methylasimilobine	CHRNA7
197017	N-Methylasimilobine	CHRNB1
197017	N-Methylasimilobine	CHRNB3
197017	N-Methylasimilobine	CTSC
197017	N-Methylasimilobine	CYP1A2
197017	N-Methylasimilobine	DPP4
197017	N-Methylasimilobine	DPP9
197017	N-Methylasimilobine	DRD1
197017	N-Methylasimilobine	DRD2
197017	N-Methylasimilobine	DRD3
197017	N-Methylasimilobine	DRD4
197017	N-Methylasimilobine	DRD5
197017	N-Methylasimilobine	DYRK2
197017	N-Methylasimilobine	EBP
197017	N-Methylasimilobine	F3

197017	N-Methylasimilobine	FAP
197017	N-Methylasimilobine	FDFT1
197017	N-Methylasimilobine	GRIN2A
197017	N-Methylasimilobine	HASPIN
197017	N-Methylasimilobine	HPGDS
197017	N-Methylasimilobine	HRH2
197017	N-Methylasimilobine	HRH4
197017	N-Methylasimilobine	HTR1A
197017	N-Methylasimilobine	HTR1B
197017	N-Methylasimilobine	HTR1D
197017	N-Methylasimilobine	HTR1E
197017	N-Methylasimilobine	HTR1F
197017	N-Methylasimilobine	HTR2A
197017	N-Methylasimilobine	HTR2B
197017	N-Methylasimilobine	HTR2C
197017	N-Methylasimilobine	HTR3A
197017	N-Methylasimilobine	HTR4
197017	N-Methylasimilobine	HTR5A
197017	N-Methylasimilobine	HTR6
197017	N-Methylasimilobine	HTR7
197017	N-Methylasimilobine	IDO1
197017	N-Methylasimilobine	KCNH2
197017	N-Methylasimilobine	L3MBTL3
197017	N-Methylasimilobine	LTA4H
197017	N-Methylasimilobine	MAOA
197017	N-Methylasimilobine	MAPK8
197017	N-Methylasimilobine	MTNR1B
197017	N-Methylasimilobine	MYLK
197017	N-Methylasimilobine	NOS2
197017	N-Methylasimilobine	NR4A1
197017	N-Methylasimilobine	OPRD1
197017	N-Methylasimilobine	OPRK1
197017	N-Methylasimilobine	OPRM1
197017	N-Methylasimilobine	PHLPP2
197017	N-Methylasimilobine	PKN1
197017	N-Methylasimilobine	PKN2
197017	N-Methylasimilobine	PRKCE
197017	N-Methylasimilobine	PRKX
197017	N-Methylasimilobine	PTPRCAP
197017	N-Methylasimilobine	ROCK1
197017	N-Methylasimilobine	ROCK2
197017	N-Methylasimilobine	ROCK2
197017	N-Methylasimilobine	RPS6KA5
197017	N-Methylasimilobine	SCN4A

197017	N-Methylasimilobine	SIGMAR1
197017	N-Methylasimilobine	SLC6A3
197017	N-Methylasimilobine	SLC6A4
197017	N-Methylasimilobine	TBXA2R
197017	N-Methylasimilobine	TH
197017	N-Methylasimilobine	TNKS2
197017	N-Methylasimilobine	TSPO
197017	N-Methylasimilobine	XIAP
23335	Caaverine	CYP2D6
23335	Caaverine	ABCB1
23335	Caaverine	ADCY5
23335	Caaverine	ADRA1A
23335	Caaverine	ADRA1B
23335	Caaverine	ADRA1D
23335	Caaverine	ADRA2A
23335	Caaverine	ADRA2B
23335	Caaverine	ADRA2C
23335	Caaverine	ADRB1
23335	Caaverine	ADRB2
23335	Caaverine	ADRB3
23335	Caaverine	ALOX12
23335	Caaverine	ALOX15
23335	Caaverine	AOC3
23335	Caaverine	CCL2
23335	Caaverine	CDK9
23335	Caaverine	CHRM1
23335	Caaverine	CHRM2
23335	Caaverine	CHRM3
23335	Caaverine	CHRNA3
23335	Caaverine	CHRNA4
23335	Caaverine	CHRNA4
23335	Caaverine	CHRNB1
23335	Caaverine	CHRNB3
23335	Caaverine	CTSC
23335	Caaverine	CYP1A2
23335	Caaverine	DPP4
23335	Caaverine	DPP9
23335	Caaverine	DRD1
23335	Caaverine	DRD2
23335	Caaverine	DRD3
23335	Caaverine	DRD4
23335	Caaverine	DRD5
23335	Caaverine	DYRK2
23335	Caaverine	F3

23335	Caaverine	FAP
23335	Caaverine	FDFT1
23335	Caaverine	HASPIN
23335	Caaverine	HRH2
23335	Caaverine	HRH4
23335	Caaverine	HTR1A
23335	Caaverine	HTR1B
23335	Caaverine	HTR1D
23335	Caaverine	HTR1E
23335	Caaverine	HTR1F
23335	Caaverine	HTR2A
23335	Caaverine	HTR2B
23335	Caaverine	HTR2C
23335	Caaverine	HTR3A
23335	Caaverine	HTR4
23335	Caaverine	HTR5A
23335	Caaverine	HTR6
23335	Caaverine	HTR7
23335	Caaverine	IDO1
23335	Caaverine	IKBKB
23335	Caaverine	KCNH2
23335	Caaverine	KISS1R
23335	Caaverine	LTA4H
23335	Caaverine	MAOA
23335	Caaverine	MAPK8
23335	Caaverine	MYLK
23335	Caaverine	NR4A1
23335	Caaverine	OPRD1
23335	Caaverine	OPRK1
23335	Caaverine	OPRM1
23335	Caaverine	PHLPP2
23335	Caaverine	PIM1
23335	Caaverine	PIM2
23335	Caaverine	PKN1
23335	Caaverine	PKN2
23335	Caaverine	PLA2G2A
23335	Caaverine	PRKCE
23335	Caaverine	PRKX
23335	Caaverine	PTPRCAP
23335	Caaverine	QDPR
23335	Caaverine	ROCK1
23335	Caaverine	ROCK2
23335	Caaverine	ROCK2
23335	Caaverine	RPS6KA5

23335	Caaverine	SCN4A
23335	Caaverine	SIGMAR1
23335	Caaverine	SLC6A3
23335	Caaverine	SLC6A4
23335	Caaverine	TBXA2R
23335	Caaverine	TH
23335	Caaverine	TNKS2
23335	Caaverine	TSPO
15515703	Jujubogenin	CYP2D6
15515703	Jujubogenin	ABCB1
15515703	Jujubogenin	ADCY5
15515703	Jujubogenin	ADORA3
15515703	Jujubogenin	ADRA1A
15515703	Jujubogenin	ADRA1B
15515703	Jujubogenin	ADRA1D
15515703	Jujubogenin	ADRA2A
15515703	Jujubogenin	ADRA2B
15515703	Jujubogenin	ADRA2C
15515703	Jujubogenin	ADRB1
15515703	Jujubogenin	ADRB2
15515703	Jujubogenin	ADRB3
15515703	Jujubogenin	ALOX12
15515703	Jujubogenin	ALOX15
15515703	Jujubogenin	AOC3
15515703	Jujubogenin	BIRC2
15515703	Jujubogenin	CCL2
15515703	Jujubogenin	CDK9
15515703	Jujubogenin	CHRM1
15515703	Jujubogenin	CHRM2
15515703	Jujubogenin	CHRM3
15515703	Jujubogenin	CHRM4
15515703	Jujubogenin	CHRNA3
15515703	Jujubogenin	CHRNA4
15515703	Jujubogenin	CHRNA4
15515703	Jujubogenin	CHRNA7
15515703	Jujubogenin	CHRNB1
15515703	Jujubogenin	CHRNB3
15515703	Jujubogenin	CTSC
15515703	Jujubogenin	CYP1A2
15515703	Jujubogenin	DPP4
15515703	Jujubogenin	DPP9
15515703	Jujubogenin	DRD1
15515703	Jujubogenin	DRD2
15515703	Jujubogenin	DRD3

15515703	Jujubogenin	DRD4
15515703	Jujubogenin	DRD5
15515703	Jujubogenin	DYRK2
15515703	Jujubogenin	EBP
15515703	Jujubogenin	F3
15515703	Jujubogenin	FAP
15515703	Jujubogenin	FDFT1
15515703	Jujubogenin	GRIN2A
15515703	Jujubogenin	HASPIN
15515703	Jujubogenin	HPGDS
15515703	Jujubogenin	HRH2
15515703	Jujubogenin	HRH4
15515703	Jujubogenin	HTR1A
15515703	Jujubogenin	HTR1B
15515703	Jujubogenin	HTR1D
15515703	Jujubogenin	HTR1E
15515703	Jujubogenin	HTR1F
15515703	Jujubogenin	HTR2A
15515703	Jujubogenin	HTR2B
15515703	Jujubogenin	HTR2C
15515703	Jujubogenin	HTR3A
15515703	Jujubogenin	HTR4
15515703	Jujubogenin	HTR5A
15515703	Jujubogenin	HTR6
15515703	Jujubogenin	HTR7
15515703	Jujubogenin	IDO1
15515703	Jujubogenin	KCNH2
15515703	Jujubogenin	L3MBTL3
15515703	Jujubogenin	LTA4H
15515703	Jujubogenin	MAOA
15515703	Jujubogenin	MAPK8
15515703	Jujubogenin	MTNR1B
15515703	Jujubogenin	MYLK
15515703	Jujubogenin	NOS2
15515703	Jujubogenin	NR4A1
15515703	Jujubogenin	OPRD1
15515703	Jujubogenin	OPRK1
15515703	Jujubogenin	OPRM1
15515703	Jujubogenin	PHLPP2
15515703	Jujubogenin	PKN1
15515703	Jujubogenin	PKN2
15515703	Jujubogenin	PRKCE
15515703	Jujubogenin	PRKX
15515703	Jujubogenin	PTPRCAP

15515703	Jujubogenin	ROCK1
15515703	Jujubogenin	ROCK2
15515703	Jujubogenin	ROCK2
15515703	Jujubogenin	RPS6KA5
15515703	Jujubogenin	SCN4A
15515703	Jujubogenin	SIGMAR1
15515703	Jujubogenin	SLC6A3
15515703	Jujubogenin	SLC6A4
15515703	Jujubogenin	TBXA2R
15515703	Jujubogenin	TH
15515703	Jujubogenin	TNKS2
15515703	Jujubogenin	TSPO
15515703	Jujubogenin	XIAP
3085285	Juzirine	ABCG2
3085285	Juzirine	ABL1
3085285	Juzirine	ACHE
3085285	Juzirine	ADORA2B
3085285	Juzirine	ADORA3
3085285	Juzirine	ADRA1A
3085285	Juzirine	ADRA1B
3085285	Juzirine	ADRA1D
3085285	Juzirine	AGPAT2
3085285	Juzirine	AHR
3085285	Juzirine	ALOX12
3085285	Juzirine	ALOX15
3085285	Juzirine	ALPL
3085285	Juzirine	BACE1
3085285	Juzirine	CA1
3085285	Juzirine	CA14
3085285	Juzirine	CA5A
3085285	Juzirine	CA5B
3085285	Juzirine	CA6
3085285	Juzirine	CA7
3085285	Juzirine	CA9
3085285	Juzirine	CAPN1
3085285	Juzirine	CASP3
3085285	Juzirine	CASP7
3085285	Juzirine	CDK2
3085285	Juzirine	CDK4
3085285	Juzirine	CHEK1
3085285	Juzirine	CLK1
3085285	Juzirine	CNR2
3085285	Juzirine	CTSD
3085285	Juzirine	CTSS

3085285	Juzirine	CYP24A1
3085285	Juzirine	CYP2C19
3085285	Juzirine	DNM1
3085285	Juzirine	DRD1
3085285	Juzirine	DRD2
3085285	Juzirine	DRD3
3085285	Juzirine	DRD5
3085285	Juzirine	DYRK1A
3085285	Juzirine	DYRK1B
3085285	Juzirine	ELANE
3085285	Juzirine	EZR
3085285	Juzirine	F3
3085285	Juzirine	FLT3
3085285	Juzirine	GCGR
3085285	Juzirine	GSK3B
3085285	Juzirine	HDAC6
3085285	Juzirine	HSD17B3
3085285	Juzirine	HSP90AA1
3085285	Juzirine	HSP90AB1
3085285	Juzirine	HTT
3085285	Juzirine	IGFBP1
3085285	Juzirine	IGFBP2
3085285	Juzirine	IGFBP3
3085285	Juzirine	IGFBP4
3085285	Juzirine	IGFBP5
3085285	Juzirine	IGFBP6
3085285	Juzirine	ILK
3085285	Juzirine	KCNH2
3085285	Juzirine	KDM1A
3085285	Juzirine	LNPEP
3085285	Juzirine	MAPK8
3085285	Juzirine	MAPKAPK2
3085285	Juzirine	MMP1
3085285	Juzirine	MMP13
3085285	Juzirine	MMP2
3085285	Juzirine	MMP3
3085285	Juzirine	MMP8
3085285	Juzirine	MMP9
3085285	Juzirine	MTOR
3085285	Juzirine	NEK1
3085285	Juzirine	NOX4
3085285	Juzirine	OPRK1
3085285	Juzirine	PAK4
3085285	Juzirine	PDE10A

3085285	Juzirine	PDE2A
3085285	Juzirine	PDE3A
3085285	Juzirine	PDE3B
3085285	Juzirine	PDE5A
3085285	Juzirine	PDGFRA
3085285	Juzirine	PDGFRB
3085285	Juzirine	PDK1
3085285	Juzirine	PIK3CA
3085285	Juzirine	PIK3CD
3085285	Juzirine	PIK3CG
3085285	Juzirine	PITRM1
3085285	Juzirine	PLA2G7
3085285	Juzirine	PLK1
3085285	Juzirine	PRKCZ
3085285	Juzirine	PTPN1
3085285	Juzirine	RAF1
3085285	Juzirine	RET
3085285	Juzirine	RPS6KA5
3085285	Juzirine	SHBG
3085285	Juzirine	TLR9
3085285	Juzirine	TRPM8
3085285	Juzirine	TTR
3085285	Juzirine	TYMS
3085285	Juzirine	VCP
3085285	Juzirine	WEE1
161388	Virgaureagenin G	ACP1
161388	Virgaureagenin G	ADAMTS4
161388	Virgaureagenin G	AKR1B10
161388	Virgaureagenin G	ALOX5
161388	Virgaureagenin G	AMPD1
161388	Virgaureagenin G	AMPD2
161388	Virgaureagenin G	AMPD3
161388	Virgaureagenin G	CD81
161388	Virgaureagenin G	CDC25A
161388	Virgaureagenin G	CDC25B
161388	Virgaureagenin G	CES2
161388	Virgaureagenin G	CHEK1
161388	Virgaureagenin G	CTSA
161388	Virgaureagenin G	CYP17A1
161388	Virgaureagenin G	CYP19A1
161388	Virgaureagenin G	CYP51A1
161388	Virgaureagenin G	EDNRA
161388	Virgaureagenin G	EDNRB
161388	Virgaureagenin G	ESR1

161388	Virgaureagenin G	ESR2
161388	Virgaureagenin G	FAAH
161388	Virgaureagenin G	FABP1
161388	Virgaureagenin G	FABP3
161388	Virgaureagenin G	FABP4
161388	Virgaureagenin G	FABP5
161388	Virgaureagenin G	FNTA
161388	Virgaureagenin G	FNTB
161388	Virgaureagenin G	G6PD
161388	Virgaureagenin G	GPBAR1
161388	Virgaureagenin G	HMGCR
161388	Virgaureagenin G	HSD11B1
161388	Virgaureagenin G	HSD11B2
161388	Virgaureagenin G	ITGA4
161388	Virgaureagenin G	ITGB1
161388	Virgaureagenin G	LTB4R
161388	Virgaureagenin G	MME
161388	Virgaureagenin G	MMP1
161388	Virgaureagenin G	MMP2
161388	Virgaureagenin G	MMP3
161388	Virgaureagenin G	NOS2
161388	Virgaureagenin G	NPC1L1
161388	Virgaureagenin G	NR1H3
161388	Virgaureagenin G	NR3C1
161388	Virgaureagenin G	NR3C2
161388	Virgaureagenin G	PDE4D
161388	Virgaureagenin G	PDE5A
161388	Virgaureagenin G	PLA2G1B
161388	Virgaureagenin G	POLB
161388	Virgaureagenin G	PPARA
161388	Virgaureagenin G	PPARD
161388	Virgaureagenin G	PPARG
161388	Virgaureagenin G	PREP
161388	Virgaureagenin G	PTGDR2
161388	Virgaureagenin G	PTGER1
161388	Virgaureagenin G	PTGER2
161388	Virgaureagenin G	PTGER4
161388	Virgaureagenin G	PTGES
161388	Virgaureagenin G	PTGFR
161388	Virgaureagenin G	PTGS1
161388	Virgaureagenin G	PTGS2
161388	Virgaureagenin G	PTPN1
161388	Virgaureagenin G	PTPN2
161388	Virgaureagenin G	PTPN6

161388	Virgaureagenin G	PTPRF
161388	Virgaureagenin G	RORC
161388	Virgaureagenin G	SERPINA6
161388	Virgaureagenin G	SHBG
161388	Virgaureagenin G	SIGMAR1
161388	Virgaureagenin G	SLC6A3
161388	Virgaureagenin G	WEE1
