# Supplementary material for: Potential Mechanism of Dingji Fumai Decoction Against Atrial Fibrillation Based on Network Pharmacology, Molecular Docking, and Experimental Verification Integration Strategy
Source: Front Cardiovasc Med. 2021 Nov 11;8:712398. doi: 10.3389/fcvm.2021.712398 (PMC8631917; doi:10.3389/fcvm.2021.712398)
Supplement: Supplementary file 3 [file Table_3.pdf]

Table S3. Designations and topological parameter of hub genes in the PPI network

Symbol	Protein Name	Degree	Betweenness	Closeness
ALB	Albumin	45	1198.18	0.66
NOS3	Nitric oxide synthase, endothelial	38	594.76	0.60
IL6	Interleukin-6	37	389.74	0.60
VEGFA	Vascular endothelial growth factor A	34	494.72	0.57
REN	Renin	34	434.06	0.59
ACE	Angiotensin-converting enzyme	33	368.88	0.57
PTGS2	Prostaglandin G/H synthase 2	28	133.45	0.55
CASP3	Caspase-3	27	240.61	0.55
F2	Prothrombin	24	155.38	0.53
AGTR1	Type-1 angiotensin II receptor	23	113.89	0.53
CYP3A4	Cytochrome P450 3A4	22	353.20	0.54
PPARG	Peroxisome proliferator-activated receptor gamma	22	194.82	0.54
CCND1	G1/S-specific cyclin-D1	21	476.39	0.48
SIRT1	NAD-dependent protein deacetylase sirtuin-1	21	263.16	0.54
VCAM1	Vascular cell adhesion protein 1	19	32.51	0.49
KCNH2	Potassium voltage-gated channel subfamily H member 2	18	447.80	0.47
CYP2C9	Cytochrome P450 2C9	18	198.42	0.53
HIF1A	Hypoxia-inducible factor 1-alpha	17	32.26	0.50
NR3C1	Glucocorticoid receptor	17	94.37	0.52
TGFB1	Transforming growth factor beta-1 proprotein	17	49.20	0.49
CYP1A1	Cytochrome P450 1A1	16	92.96	0.49
CACNA1C	Voltage-dependent L-type calcium channel subunit alpha-1C	16	136.21	0.43
PLG	Plasminogen	16	13.41	0.48
KCNJ5	G protein-activated inward rectifier potassium channel 4	15	258.75	0.46
ADRB2	Beta-2 adrenergic receptor	15	175.15	0.51
F3	Tissue factor	15	19.93	0.48
SCN5A	Sodium channel protein type 5 subunit alpha	14	46.15	0.41
NR1I2	Nuclear receptor subfamily 1 group I member 2	14	55.81	0.46
SELE	E-selectin	14	8.59	0.48
SLCO1B1	Solute carrier organic anion transporter family member 1B1	13	170.67	0.45
MCHR1	Melanin-concentrating hormone receptor 1	13	94.81	0.45
CYP2C19	Cytochrome P450 2C19	13	71.69	0.51
IGF1R	Insulin-like growth factor 1 receptor	12	12.86	0.47
CYP2D6	Cytochrome P450 2D6	12	104.40	0.50
ESR2	Estrogen receptor beta	12	37.90	0.49
ADRB1	Beta-1 adrenergic receptor	12	148.71	0.48
PLAT	Tissue-type plasminogen activator	12	7.35	0.47
KCNA5	Potassium voltage-gated channel subfamily A member 5	11	115.65	0.43
CYP1A2	Cytochrome P450 1A2	11	51.77	0.47
PPARA	Peroxisome proliferator-activated receptor alpha	11	8.80	0.48

HTR1A	5-hydroxytryptamine receptor 1A	11	119.74	0.45
ADORA1	Adenosine receptor A1	11	74.88	0.48
CYP11B2	Cytochrome P450 11B2, mitochondrial	11	153.88	0.47
ADRA1B	Alpha-1B adrenergic receptor	11	21.52	0.45
ADRA2A	Alpha-2A adrenergic receptor	11	65.31	0.45
CACNA2D1	Voltage-dependent calcium channel subunit alpha-2/delta-1	10	21.48	0.39
HCN4	Potassium/sodium hyperpolarization-activated cyclic nucleotide-gated channel 4	10	15.57	0.38
SCN10A	Sodium channel protein type 10 subunit alpha	10	196.71	0.48
UGT2B7	UDP-glucuronosyltransferase 2B7	10	7.12	0.39
ADRA1A	Alpha-1A adrenergic receptor	10	32.43	0.45
EDNRA	Endothelin-1 receptor	10	9.06	0.45
HMGCR	3-hydroxy-3-methylglutaryl-coenzyme A reductase	10	22.90	0.48
KCNN3	Small conductance calcium-activated potassium channel protein 3	9	36.25	0.39
NR3C2	Mineralocorticoid receptor	9	4.23	0.46
NPPB	Natriuretic peptides B	9	96.75	0.48
ADRA2C	Alpha-2C adrenergic receptor	9	15.03	0.43
ADRA1D	Alpha-1D adrenergic receptor	9	1.81	0.42
ADRA2B	Alpha-2B adrenergic receptor	9	15.03	0.43
PTGS1	Prostaglandin G/H synthase 1	8	8.15	0.46
KCNJ11	ATP-sensitive inward rectifier potassium channel 11	8	118.09	0.44
SLC8A1	Sodium/calcium exchanger 1	8	25.10	0.36
CES1	Liver carboxylesterase 1	8	2.64	0.43
ADRB3	Beta-3 adrenergic receptor	8	29.63	0.44
IKBKB	Inhibitor of nuclear factor kappa-B kinase subunit beta	7	1.00	0.42
CASP1	Caspase-1	7	1.19	0.42
PTK2	Focal adhesion kinase 1	7	2.65	0.43
CAMK2D	Calcium/calmodulin-dependent protein kinase type II subunit delta	7	111.17	0.43
HTR1B	5-hydroxytryptamine receptor 1B	7	17.56	0.39
KCNN2	Small conductance calcium-activated potassium channel protein 2	6	0.00	0.37
TNNT2	Troponin T, cardiac muscle	6	22.94	0.38
MAPT	Microtubule-associated protein tau	6	63.54	0.45
F10	Coagulation factor X	6	1.93	0.42
FGFR1	Fibroblast growth factor receptor 1	6	53.54	0.45
CDK6	Cyclin-dependent kinase 6	5	0.00	0.40
SCN4A	Sodium channel protein type 4 subunit alpha	5	0.39	0.36
AKR1C1	Aldo-keto reductase family 1 member C1	5	12.02	0.39
SLC47A1	Multidrug and toxin extrusion protein 1	4	20.59	0.36
XPO1	Exportin-1	4	0.27	0.39
XDH	Xanthine dehydrogenase/oxidase	4	3.45	0.44

RPS6KA3	Ribosomal protein S6 kinase alpha-3	3	2.88	0.35
ATP1A1	Sodium/potassium-transporting ATPase subunit alpha-1	3	6.15	0.35
FGFR2	Fibroblast growth factor receptor 2	3	0.00	0.42
HTR4	5-hydroxytryptamine receptor 4	3	0.00	0.35
SLC22A6	Solute carrier family 22 member 6	2	0.00	0.31
THRB	Thyroid hormone receptor beta	2	0.00	0.33
THRA	Thyroid hormone receptor alpha	2	0.00	0.33
HPSE	Heparanase	1	0.00	0.37
