# Supplementary material for: Potential Mechanism of Dingji Fumai Decoction Against Atrial Fibrillation Based on Network Pharmacology, Molecular Docking, and Experimental Verification Integration Strategy
Source: Front Cardiovasc Med. 2021 Nov 11;8:712398. doi: 10.3389/fcvm.2021.712398 (PMC8631917; doi:10.3389/fcvm.2021.712398)
Supplement: Supplementary file 4 [file Table_4.pdf]

Table S4. The results of relevance analysis.

Symbol	Description	Score	Relationship
SCN5A	Sodium Voltage-Gated Channel Alpha Subunit 5	602.66	Directely
KCNA5	Potassium Voltage-Gated Channel Subfamily A Member 5	471.03	Directely
KCNH2	Potassium Voltage-Gated Channel Subfamily H Member 2	424.92	Directely
HCN4	Hyperpolarization Activated Cyclic Nucleotide Gated Potassium Channel 4	391.41	Directely
KCNJ5	Potassium Inwardly Rectifying Channel Subfamily J Member 5	391.41	Directely
CACNA1C	Calcium Voltage-Gated Channel Subunit Alpha1 C	391.41	Directely
CACNA2D1	Calcium Voltage-Gated Channel Auxiliary Subunit Alpha2delta 1	313.82	Directely
TNNT2	Troponin T2, Cardiac Type	276.77	Directely
F2	Coagulation Factor II, Thrombin	233.91	Directely
CYP2C9	Cytochrome P450 Family 2 Subfamily C Member 9	221.91	Directely
NOS3	Nitric Oxide Synthase 3	209.22	Directely
SCN10A	Sodium Voltage-Gated Channel Alpha Subunit 10	143.24	Directely
KCNJ11	Potassium Inwardly Rectifying Channel Subfamily J Member 11	143.24	Directely
ALB	Albumin	122.66	Directely
NPPB	Natriuretic Peptide B	122.66	Directely
REN	Renin	116.96	Directely
ACE	Angiotensin I Converting Enzyme	116.96	Directely
IL6	Interleukin 6	116.96	Directely
TGFB1	Transforming Growth Factor Beta 1	110.95	Directely
PLAT	Plasminogen Activator, Tissue Type	110.95	Directely
CYP11B2	Cytochrome P450 Family 11 Subfamily B Member 2	104.61	Directely
F3	Coagulation Factor III, Tissue Factor	104.61	Directely
PPARG	Peroxisome Proliferator Activated Receptor Gamma	104.61	Directely
AGTR1	Angiotensin II Receptor Type 1	104.61	Directely
F10	Coagulation Factor X	97.85	Directely
ADORA1	Adenosine A1 Receptor	97.85	Directely
ADRB1	Adrenoceptor Beta 1	97.85	Directely
NR3C2	Nuclear Receptor Subfamily 3 Group C Member 2	97.85	Directely
HMGCR	3-Hydroxy-3-Methylglutaryl-CoA Reductase	97.85	Directely
KCNN3	Potassium Calcium-Activated Channel Subfamily N Member 3	90.59	Directely
EDNRA	Endothelin Receptor Type A	64.06	Directely
PLG	Plasminogen	52.3	Directely
VEGFA	Vascular Endothelial Growth Factor A	52.3	Directely
CAMK2D	Calcium/Calmodulin Dependent Protein Kinase II Delta	36.98	Directely
ADRB3	Adrenoceptor Beta 3	36.98	Directely
PPARA	Peroxisome Proliferator Activated Receptor Alpha	36.98	Directely
CASP1	Caspase 1	36.98	Directely
ATP1A1	ATPase Na+/K+ Transporting Subunit Alpha 1	36.98	Directely
ADRA2C	Adrenoceptor Alpha 2C	36.98	Directely
ADRA1D	Adrenoceptor Alpha 1D	36.98	Directely
SIRT1	Sirtuin 1	36.98	Directely

SLC8A1	Solute Carrier Family 8 Member A1	36.98	Directely
MAPT	Microtubule Associated Protein Tau	36.98	Directely
CASP3	Caspase 3	36.98	Directely
VCAM1	Vascular Cell Adhesion Molecule 1	36.98	Directely
ADRB2	Adrenoceptor Beta 2	36.98	Directely
XDH	Xanthine Dehydrogenase	36.98	Directely

Notes: The score is an indication of the strength of the connection between the gene and the disorders.
