# Supplementary material for: Potential Mechanism of Dingji Fumai Decoction Against Atrial Fibrillation Based on Network Pharmacology, Molecular Docking, and Experimental Verification Integration Strategy
Source: Front Cardiovasc Med. 2021 Nov 11;8:712398. doi: 10.3389/fcvm.2021.712398 (PMC8631917; doi:10.3389/fcvm.2021.712398)
Supplement: Supplementary file 5 [file Table_5.pdf]

Table S5. The details of corresponding proteins in molecular docking.

Gene Symbol	Protein	PDB ID
NOS3	Nitric oxide synthase, endothelial	1M9J
ACE	Angiotensin-converting enzyme	1O86
SIRT1	NAD-dependent protein deacetylase sirtuin-1	4IG9
IL6	Interleukin-6	1ALU
VCAM1	Vascular cell adhesion protein 1	1IJ9
KCNH2	Potassium voltage-gated channel subfamily H member 2	1BYW
